# Radiation and Dose-densification of R-CHOP in Aggressive B-cell Lymphoma With Intermediate Prognosis: The UNFOLDER Study

**DOI:** 10.1097/HS9.0000000000000904

**Published:** 2023-07-05

**Authors:** Lorenz Thurner, Marita Ziepert, Christian Berdel, Christian Schmidt, Peter Borchmann, Dominic Kaddu-Mulindwa, Andreas Viardot, Mathias Witzens-Harig, Judith Dierlamm, Mathias Haenel, Bernd Metzner, Gerald Wulf, Eva Lengfelder, Ulrich B. Keller, Norbert Frickhofen, Maike Nickelsen, Tobias Gaska, Frank Griesinger, Rolf Mahlberg, Reinhard Marks, Ofer Shpilberg, Hans-Walter Lindemann, Martin Soekler, Ludwig Fischer von Weikersthal, Michael Kiehl, Eva Roemer, Martin Bentz, Beate Krammer-Steiner, Ralf Trappe, Peter de Nully Brown, Massimo Federico, Francesco Merli, Marianne Engelhard, Bertram Glass, Norbert Schmitz, Lorenz Truemper, Moritz Bewarder, Frank Hartmann, Niels Murawski, Stephan Stilgenbauer, Andreas Rosenwald, Bettina Altmann, Heinz Schmidberger, Jochen Fleckenstein, Markus Loeffler, Viola Poeschel, Gerhard Held

**Affiliations:** 1 Department of Internal Medicine 1 (Oncology, Hematology, Clinical Immunology and Rheumatology), Saarland University Medical School, Homburg/Saar, Germany; 2 Institute for Medical Informatics, Statistics and Epidemiology, University Leipzig, Germany; 3 Department of Radiotherapy and Radiation Oncology, Saarland University Medical School, Homburg/Saar, Germany; 4 Department of Medicine III, University Hospital, Munich, Germany; 5 Department of Hematology and Oncology, University Hospital of Cologne, Germany; 6 Department of Internal Medicine III, University Hospital Ulm, Germany; 7 Department of Internal Medicine V, University of Heidelberg, Germany; 8 Department of Internal Medicine II, University Hospital Eppendorf, Hamburg, Germany; 9 Department of Internal Medicine III, Küchwald Hospital Chemnitz, Germany; 10 Department of Hematology and Oncology, University Clinic, Klinikum Oldenburg, Germany; 11 Department of Hematology and Oncology, Georg August University of Goettingen, Germany; 12 Department of Internal Medicine III, University Hospital Mannheim, Germany; 13 Department of Internal Medicine III, Klinikum rechts der Isar der TU München, Munich, Germany; 14 Department of Internal Medicine III, Helios Dr. Horst-Schmidt-Kliniken, Wiesbaden, Germany; 15 Oncology Lerchenfeld, Hamburg, Germany; 16 Department of Hematology and Oncology, Brüderkrankenhaus St. Josef, Paderborn, Germany; 17 Department of Internal Oncology, Pius-Hospital, Oldenburg, Germany; 18 Department of Internal Medicine I, Klinikum Mutterhaus der Borromaerinnen, Trier, Germany; 19 Department of Hematology and Oncology, University Medical Center, Freiburg, Germany; 20 Department of Hematology, Rabin Medical Center, Beilinson Hospital, Petah-Tiqwa, Israel; 21 Department of Hematology and Internal Oncology, St.-Josefs-Hospital, Hagen, Germany; 22 Department of Internal Medicine II, University Hospital Tuebingen, Germany; 23 Medical Service Center, Klinikum St. Marien, Amberg, Germany; 24 Department of Internal Medicine, Klinikum Frankfurt (Oder), Germany; 25 Department of Gastroenterology, Nephrology, Diabeteology, Hematoloy, Internal Oncology and Internal Intensive Medical Care, Klinikum Idar-Oberstein, Germany; 26 Department of Hematology and Oncology, Städtisches Klinikum, Karlsruhe, Germany; 27 Department of Internal Medicine, Klinikum Rostock Südstadt, Germany; 28 Department of Internal Medicine II, Evang. Diakonie-Krankenhaus gGmbH, Bremen, Germany; 29 Department of Hematology, Rigshospitalet, Copenhagen, Denmark; 30 CHIMOMO Department, University of Modena and Reggio Emilia, Italy; 31 Hematology Azienda USL-IRCCS of Reggio Emilia, Italy; 32 Department of Radiotherapy, University Hospital Essen, Germany; 33 Department of Hematology and Stem Cell Transplantation, Helios Klinikum Berlin-Buch, Germany; 34 Department of Medicine A, Hematology, Oncology and Pneumology, University Hospital Münster, Germany; 35 Institute of Pathology, University of Wuerzburg, and Comprehensive Cancer Center Mainfranken, Germany; 36 Department of Radiooncology and Radiotherapy, University Medical Center, Mainz, Germany; 37 Department of Internal Medicine 1, Westpfalz-Klinikum, Kaiserslautern, Germany

## Abstract

UNFOLDER (Unfavorable Young Low-Risk Densification of R-Chemo Regimens) is an international phase-3 trial in patients 18–60 years with aggressive B-cell lymphoma and intermediate prognosis defined by age-adjusted International Prognostic Index (aaIPI) of 0 and bulky disease (≥7.5 cm) or aaIPI of 1. In a 2 × 2 factorial design patients were randomized to 6× R-CHOP-14 or 6× R-CHOP-21 (rituximab, cyclophosphamide, doxorubicin, vincristine, and prediso[lo]ne) and to consolidation radiotherapy to extralymphatic and bulky disease or observation. Response was assessed according to the standardized response criteria published in 1999, not including F-18 fluordesoxyglucose positron emission tomography/computed tomography (FDG-PET). Primary endpoint was event-free survival (EFS). A total of 695 of 700 patients were eligible for the intention-to-treat analysis. Totally 467 patients qualified for radiotherapy of whom 305 patients were randomized to receive radiotherapy (R-CHOP-21: 155; R-CHOP-14: 150) and 162 to observation (R-CHOP-21: 81, R-CHOP-14: 81). Two hundred twenty-eight patients not qualifying for radiotherapy were randomized for R-CHOP-14 versus R-CHOP-21. After a median observation of 66 months 3-year EFS was superior in the radiotherapy-arm versus observation-arm (84% versus 68%; *P* = 0.0012), due to a lower rate of partial responses (PR) (2% versus 11%). PR often triggered additional treatment, mostly radiotherapy. No significant difference was observed in progression-free survival (PFS) (89% versus 81%; *P* = 0.22) and overall survival (OS) (93% versus 93%; *P* = 0.51). Comparing R-CHOP-14 and R-CHOP-21 EFS, PFS and OS were not different. Patients randomized to radiotherapy had a superior EFS, largely due to a lower PR rate requiring less additional treatment (NCT00278408, EUDRACT 2005-005218-19).

## Graphical abstract

**Figure:**
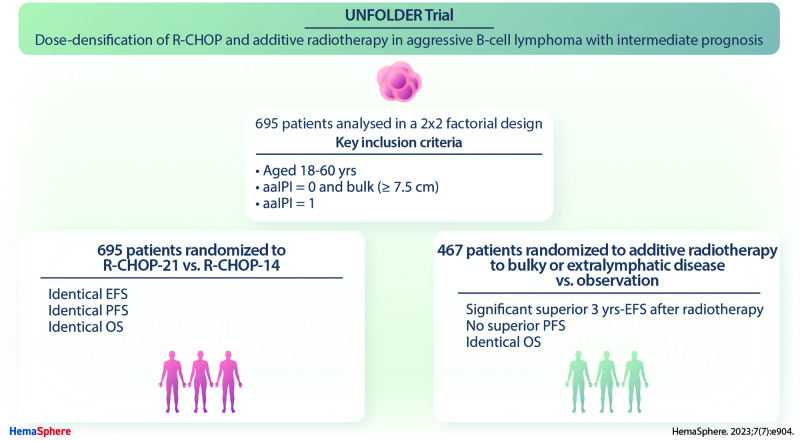

## INTRODUCTION

In aggressive B-cell non-Hodgkin lymphoma, CHOP chemotherapy (cyclophosphamide, doxorubicin, vincristine and prednisone) combined with rituximab is the standard of care.^1–3^ However, patients with risk factors as defined by the International Prognostic Index (IPI) still need further improvement of therapy.^4^ For patients aged ≤60 years, the age-adjusted IPI (aaIPI) including serum lactate dehydrogenase (LDH), stage, and performance status has been established.^4^ Bulky disease (maximal tumor diameter ≥7.5 cm) has been identified as an additional risk factor for patients in the MInT trial with a significant impact on event-free survival (EFS) and overall survival (OS).^5,6^ Patients without any of these risk factors can be safely de-escalated to 4 cycles of R-CHOP-21 plus 2 applications of rituximab as shown in the FLYER trial.^7^ In contrast, the unfavorable subgroup of young patients require a more effective therapy. A principle to improve efficacy of CHOP chemotherapy represents its dose-densification by reducing the intervals between cycles, which had been demonstrated in trials from the prerituximab era.^8,9^ Chemotherapy-intensification and -densification by rituximab, doxorubicin, cyclophosphamide vindesine, bleomycin, prednisone (R-ACVBP) improved EFS, PFS, and OS when compared with 8 cycles of R-CHOP-21 in the LNH03-2B trial.^10^ R-ACVBP is a dose-intensive, modified R-CHOP-like regimen given in 2-weekly intervals followed by subsequent consolidation. Another principle to improve efficacy of therapy might be consolidation radiotherapy, which might overcome the negative prognostic effect of bulky disease.^5,11^ Moreover, manifestations at extranodal sites showed improved outcome after consolidation radiotherapy in retrospective analyses.^12,13^

In the UNFOLDER (Unfavorable Young Low-Risk Densification of R-Chemo Regimens) trial, we investigated the impact of dose-densification of standard R-CHOP-21 to a 2-weekly R-CHOP-14 and the role of consolidation radiotherapy applied to initial bulky and extralymphatic disease in newly diagnosed adult patients aged ≤60 years with intermediate prognosis.

## METHODS

### Patients

UNFOLDER is a 2 × 2 factorial design, phase-III trial from 148 clinical sites in Denmark, Israel, Italy, and Germany (Suppl. Digital content; Suppl. Table S1). It was coordinated by the German High-grade Non-Hodgkin’s Lymphoma Study Group, now part of the German Lymphoma Alliance. It was conducted in accordance with the Helsinki declaration. Protocol and its amendments were approved by the ethics committee of each participating center. Additional information about trial oversight is provided in the Suppl. Appendix (p.9) and Suppl. Table S2. Patients aged between 18 and 60 years were eligible if they presented with untreated aggressive B-cell lymphoma according to the World Health Organization (WHO) classification (3rd edition, 2001 and 4th edition, 2008) and if they had 1 risk factor according to the aaIPI (LDH above the upper limit of normal [ULN], ECOG performance status 2 or 3, Ann Arbor stage III or IV), or no risk factor according to the aaIPI but bulky disease (diameter of single or conglomerate tumor ≥7.5 cm). Patients with central nervous system (CNS) involvement were excluded. More details are provided in the protocol in the appendix.

### Treatments

R-CHOP comprised rituximab (375 mg/m^2^), cyclophosphamide (750 mg/m^2^), doxorubicin (50 mg/m^2^), vincristine (1.4 mg/m^2^, maximum total dose of 2 mg) administered on day 1, and oral prednisone/prednisolone (100 mg) administered on days 1–5. R-CHOP-14 was repeated every 2 weeks with mandatory granulocyte colony-stimulating factor (G-CSF)-support and R-CHOP-21 every 3 weeks. Patients qualifying for radiotherapy had bulky disease (≥7.5 cm) not surgically removed and/or extralymphatic involvement amenable for radiotherapy. Involvements of bone marrow, lung, liver, kidney, small intestine, colon, ascites, pericardial, and pleural effusions were planned not to receive radiotherapy. Radiotherapy was administered at a total dose of 39.6 Gy involved-field with 1.8 Gy/fraction 5 times a week. Radiotherapy should be started 2–6 weeks after end of chemotherapy. Initial staging images and the radiotherapy-plan and verification-images were evaluated by reference radiotherapy panel. Prophylactic radiotherapy with 30.6 Gy to the contralateral testis in testicular involvement was mandatory since the second amendment.

### Response assessment and end points

Response was assessed according to the International Workshop to Standardize Response Criteria for Non-Hodgkin’s Lymphomas published in 1999.^14^ Responding patients with residual masses were assessed as unconfirmed complete response (CRu) (residual lymphoma regressed by >75% in the sum of the product of greatest diameters [SPDs]) or partial response (PR) (residual lymphoma regressed by ≥50% in SPD). PR also indicated the need for additional treatment by vital lymphoma in biopsy or by the judgement of the investigator. Final response was assessed 2 weeks after the sixth cycle of R-CHOP in the observation-arm. Patients with CRu/PR in restaging after completion of R-CHOP received confirmation of remission 4 weeks thereafter. Final response was assessed after end of radiotherapy simultaneously with first follow-up in the radiotherapy-arm. First follow-up examination was done 3 months after restaging after 6 cycles of R-CHOP.

EFS was the primary end point, defined as time from randomization until one of the following events had occurred: progression during therapy, no change, termination of therapy due to toxicity without CR/CRu, no CR/CRu at the end of study treatment, relapse after CR/CRu, death from any cause, or application of additional treatment, whichever came first.

Radiotherapy as additional treatment was not counted as an event in patients of the observation-arm who received radiotherapy due to the results of the interim analysis. In these patients, response assessment was performed after radiotherapy. Key secondary end points were PFS, defined as the date from randomization to disease progression, relapse, or any cause of death and OS defined as the time from randomization to death of any cause.

Other secondary end points were rate of CRs and progressive disease, relapse patterns (relapse in regions treated with radiotherapy, relapse in primarily involved regions and in not primarily involved regions), safety (adverse events, serious adverse events, rate of secondary neoplasia, selected laboratory parameters, including leucocytes, thrombocytes, and hemoglobin), adherence to protocol (duration of cycles, cumulative dose, and dose intensity), and health-economic aspects.

For patients qualifying for radiotherapy, an as-treated analysis was performed for PFS and OS. Patients who were randomized in observation-arms but received radiotherapy were analyzed in radiotherapy-arms.

### Statistical analysis

The UNFOLDER trial was planned for patients qualifying for radiotherapy in a 2 × 2 factorial design to show differences in comparison of chemotherapy dose-densification (6× R-CHOP-14 versus 6× R-CHOP-21) and in the impact of radiotherapy to bulky disease and/or extralymphatic involvement (6× R-CHOP-21/14 with radiotherapy versus 6× R-CHOP-21/14 observation).

Randomization was done before the start of R-CHOP using the Pocock minimization algorithm with a random component after stratification for centers, LDH (normal versus elevated), stage (I, II versus III, IV), ECOG performance status (0,1 versus 2,3), bulky disease (no versus yes), and extralymphatic sites (no versus yes). Randomization was performed at a ratio of 1:1:1:1 in the following treatment arms: 6× R-CHOP–21 + radiotherapy, or 6× R-CHOP-14 + radiotherapy, or only 6× R-CHOP–21 or 6× R-CHOP-14. In addition, patients not qualifying for consolidation radiotherapy were randomized at a ratio of 1:1 to receive either 6× R-CHOP–21 or 6× R-CHOP-14. It was powered to show a hazard ratio (HR) of 0.615 or an improvement of 10% in the primary end point of 3-year EFS (71%–81%) for dose-densification and radiotherapy. With 578 patients, a power of 80% can be achieved for a 2-sided log-rank test and a significance level of 5%. We expected that 60% of included patients would have a qualification for radiotherapy and therefore 964 patients had to be included. Plus 10% of patients should be randomized to carry out the per protocol analysis (patients with confirmed reference pathology and conformity to entry criteria) with sufficient power. This resulted in an intended sample size of 1072 patients.

A planned interim analysis was performed on July 1, 2012. A total of 443 patients were evaluable for analysis, of whom 285 were qualified and randomized to receive radiotherapy. In this analysis, the predefined formal criterion of discontinuation was fulfilled, because EFS of the 139 patients randomized to receive radiotherapy was significantly better compared with those in the observational arm, with a *P*-value of 0.004 favoring the radiotherapy-arm, thus meeting the alpha spending function of *P* = 0.008. The Data and Safety Monitoring Committee (DSMC) and recommended July 31st, 2012 to close the 2 treatment arms (R-CHOP-21 and R-CHOP-14) without radiotherapy and to continue both arms with radiotherapy (R-CHOP-21 with radiotherapy; R-CHOP-14 with radiotherapy) as planned. A second interim analysis was planned with 450 patients qualifying for radiotherapy, in a total of 682 patients. Due to slow recruitment, the trial was stopped earlier and the last patient was randomized on November 16, 2015. With 700 patients included and 695 analyzable patients, the power to detect the 3-year EFS difference of 10% for dose-densification within the pooled cohort (qualifying and not qualifying for radiotherapy) was 86%.

Characteristics of patients were compared by χ^2^ tests and, if necessary, by Fisher exact tests. Treatment duration and dose reduction were assessed using a Kaplan-Meier like estimator.^15^ Response and relapse rates were presented with 95% confidence intervals (CI). Dose-densification (14 versus 21 days) and radiotherapy (radiotherapy versus observation) were analyzed for EFS, PFS, and OS using Kaplan-Meier plots and log-rank tests. Multivariable Cox regression models adjusted for strata were performed (LDH, stage, bulk, extralymphatic involvement). HR with 95% CI were presented. The significance level was 2-sided at 0.05. Statistical analyses were done with SPSS (version 24/25/26/28).

## RESULTS

**Figure 1.**
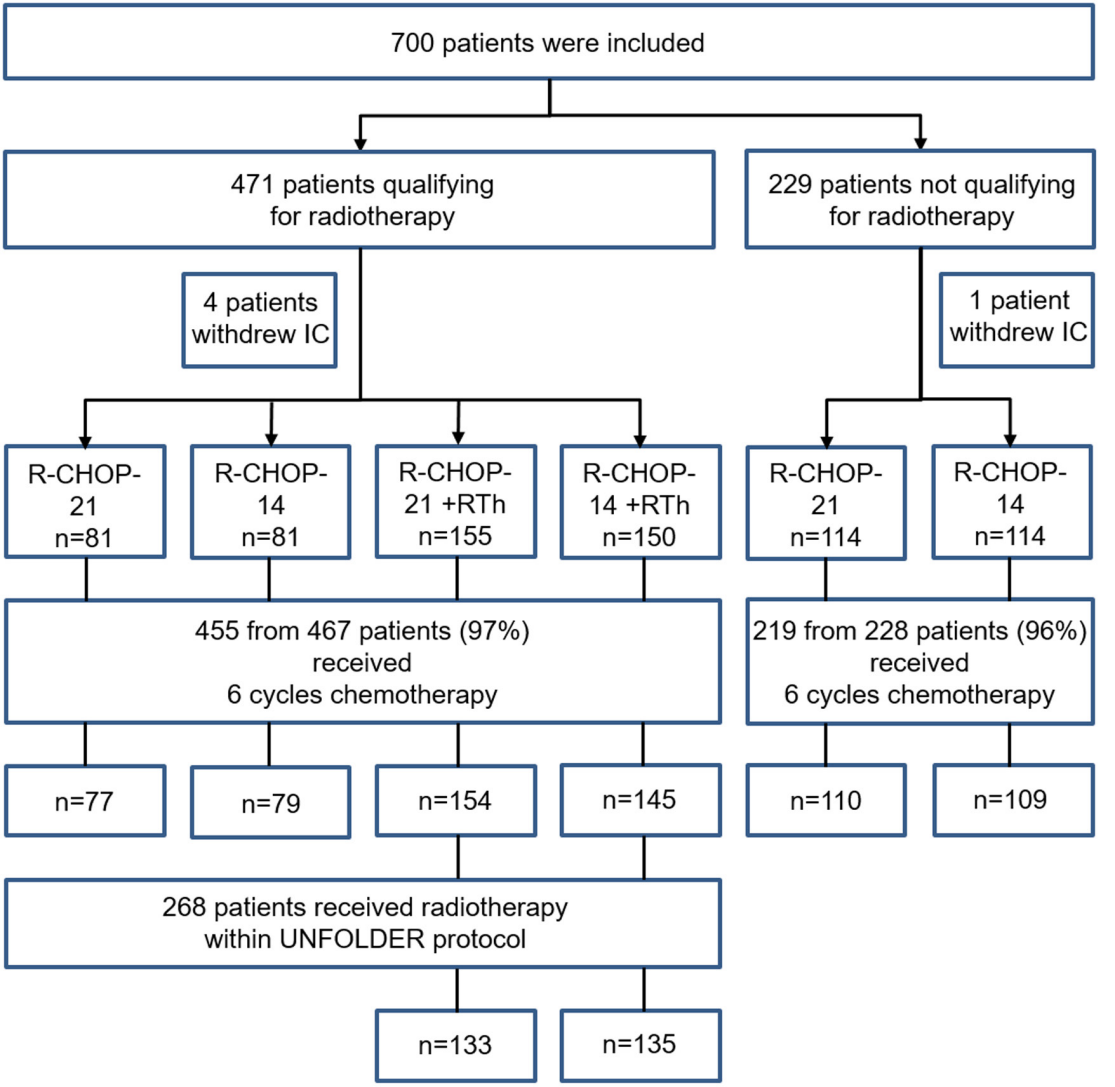
**Trial profile.** Qualification for radiotherapy was defined as the presence of bulky disease (maximal tumor diameter ≥7.5 cm) or extralymphatic involvement.

From January 2, 2006 to November 16, 2015, totally 700 patients were enrolled at 148 sites. Five patients withdrew consent leaving 695 patients for the intention-to-treat analysis (Figure 1). Baseline characteristics were well balanced (Table 1; Suppl. Table S3). Median age was 47 years, 58% were male, 42% had LDH > ULN, 16% had aaIPI 0, 83% had aaIPI 1, 1% had aaIPI 2, 57% had bulky disease, 47% had extralymphatic involvement, and 16% extralymphatic involvement > 1 site. Two hundred and twenty-eight patients (median age, 50 years; male, 62%; aaIPI 1, 91%) did not qualify for radiotherapy. Four hundred and sixty-seven patients (median age, 44 years; male, 56%; aaIPI 1, 79%; 131) with primary mediastinal B-cell lymphoma (PMBCL) were qualifying for radiotherapy. Suppl. Table S4 provides the details on the extralymphatic localizations.

**Table 1:** Baseline Demographic and Disease Characteristics (Intention-to-Treat Population)

	Qualifying for Radiotherapy (n = 467)				Not Qualifying for Radiotherapy (n = 228)	
	R-CHOP-21 (n = 81)	R-CHOP-14 (n = 81)	R-CHOP-21 + Radiotherapy (n = 155)	R-CHOP-14 + Radiotherapy (n = 150)	R-CHOP-21 (n=114)	R-CHOP-14 (n=114)
Male	49 (60%)	43 (53%)	86 (55%)	83 (55%)	71 (62%)	71 (62%)
Female	32 (40%)	38 (47%)	69 (45%)	67 (45%)	43 (38%)	43 (38%)
Age, median (range)	43 (20–60)	45 (20–60)	46 (18–60)	44 (18–60)	50 (18–60)	49 (20–60)
LDH > ULN	36 (44%)	37 (46%)	71 (46%)	67 (45%)	40 (35%)	42 (37%)
ECOG > 1	1 (1%)	1 (1%)	0 (0%)	0 (0%)	0 (0%)	1 (1%)
Stage III/ IV	30 (37%)	28 (35%)	55 (35%)	52 (35%)	64 (56%)	67 (59%)
aaIPI						
0^*a*^	14 (17%)	16 (20%)	33 (21%)	31 (21%)	12 (11%)	5 (4%)
1	67 (83%)	64 (79%)	118 (76%)	119 (79%)	100 (88%)	108 (95%)
2	0 (0%)	1 (1%)	4 (3%)	0 (0%)	2 (2%)	1 (1%)
Stage						
I	28 (35%)	20 (25%)	43 (28%)	31 (21%)	18 (16%)	19 (17%)
II	23 (28%)	33 (41%)	57 (37%)	67 (45%)	32 (28%)	28 (25%)
III	9 (11%)	7 (9%)	15 (10%)	16 (11%)	36 (32%)	41 (36%)
IV	21 (26%)	21 (26%)	40 (26%)	36 (24%)	28 (25%)	26 (23%)
Extralymph. involv.	45 (56%)	37 (46%)	80 (52%)	80 (53%)	43 (38%)	39 (34%)
Extralymph. involv. > 1	14 (17%)	18 (22%)	33 (21%)	23 (15%)	14 (12%)	11 (10%)
Bulk ≥ 7.5 cm	59 (73%)	60 (74%)	121 (78%)	117 (78%)	22 (19%)	17 (15%)
B symptoms^*b*^	23 (28%)	19 (23%)	35 (23%)	41 (28%)	22 (19%)	12 (11%)
BM involvement	3 (4%)	5 (6%)	8 (5%)	6 (4%)	13 (11%)	6 (5%)
Reference pathology available	79 (98%)	74 (91%)	150 (97%)	146 (97%)	105 (92%)	101 (89%)
DLBCL	69 (87%)	62 (84%)	133 (89%)	132 (90%)	80 (76%)	81 (80%)
PMBCL^*c*^	27 (34%)	22 (30%)	43 (29%)	39 (27%)	2 (2%)	3 (3%)
Follicular lymphoma III°b	3 (4%)	2 (3%)	3 (2%)	1 (1%)	7 (7%)	3 (3%)
Follicular lymphoma III°+DLBCL	2 (2%)	3 (4%)	3 (2%)	6 (4%)	11 (10%)	11 (11%)
Burkitt‘s lymphoma	2 (2%)	0 (0%)	1 (1%)	0 (0%)	2 (2%)	1 (1%)
Burkitt-like	0 (0%)	1 (1%)	2 (1%)	0 (0%)	0 (0%)	0 (0%)
Aggressive marginal zone lymphoma	1 (1%)	0 (0%)	0 (0%)	0 (0%)	3 (3%)	1 (1%)
Grey zone lymphoma	0 (0%)	1 (1%)	1 (1%)	1 (1%)	0 (0%)	0 (0%)
B-cell, NOS	0 (0%)	1 (1%)	0 (0%)	0 (0%)	1 (1%)	1 (1%)
B-cell, unclassified (techn. insufficient mat.)	0 (0%)	1 (1%)	3 (2%)	3 (2%)	0 (0%)	0 (0%)
Other, not B-cell	2 (2%)	3 (4%)	4 (3%)	3 (2%)	1 (1%)	3 (3%)

Bone marrow is counted as extralymphatic; spleen and waldeyers ring are counted as lymphathic DLBCL.

^*a*^10 (0/2/1/1/4/2) IPI = 0 without bulk.

^*b*^4 (0/0/2/1/0/1) missing values.

^*c*^Subtype of DLBCL.

aaIPI = age-adjusted International Prognostic Index; BM = bone marrow; DLBCL = diffuse large B-cell lymphoma; ECOG = Eastern Cooperative Oncology Group; LDH = lactate dehydrogenase; NOS = not otherwise specified; PMBCL = primary mediastinal B-cell lymphoma; R-CHOP = rituximab, cyclophosphamide, doxorubicin, vincristine, and prednisone; ULN = upper limit of normal.

According to the primary pathology report, 621 of 695 (89%) patients had a diffuse large B-cell lymphoma (DLBCL) or one of its subtypes (Suppl. Table S5). Randomization and treatment allocation was based on the diagnosis of the primary pathology. Reference pathology review was performed in 655 from 695 (94%) of patients (Table 1; Suppl. Table S3).

No relevant differences in protocol adherence and dose-achievement were observed between R-CHOP-21 and R-CHOP-14 (Suppl. Figure S1). A total of 268 of 305 (88%) patients randomized in the radiotherapy-arm received radiotherapy according to the protocol. Eleven patients did not receive radiotherapy as planned due to insufficient response. The reasons for not giving radiotherapy are depicted in Table 2; 152 of 162 (94%) patients allocated to the observation-arm did not receive radiotherapy, but 7 patients received radiotherapy due to the results of the interim analysis and 3 patients due to protocol violation. Ten patients received radiotherapy after achieving a PR at final restaging, resulting in 20 of 162 (12%) patients who received radiotherapy in the observation-arm. Two hundred and nineteen of 228 (96%) patients not qualifying for radiotherapy did not receive radiotherapy according to the protocol.

**Table 2:** Application of Radiotherapy for Patients Qualifying for Radiotherapy (n = 467)

	R-CHOP-21 (n = 81)	R-CHOP-14 (n = 81)	R-CHOP-21 + Radiotherapy (n = 155)	R-CHOP-14 + Radiotherapy (n = 150)^*a*^
Radiotherapy given				
According to protocol	-	-	133 (86%)	135 (90%)
Protocol violation^*b*^	2 (2%)	1 (1%)	-	-
Due to interim analysis^*c*^	4 (5%)	3 (4%)	-	-
Radiotherapy not given				
According to protocol	75 (93%)	77 (95%)	-	-
Insufficient response	-	-	4 (3%)	7 (5%)
Toxicity^*d*^	-	-	3 (2%)	-
Protocol violation	-	-	5 (3%)	4 (3%)
Patients decision	-	-	6 (4%)	1 (1%)
Concomitant disease	-	-	1 (1%)	-
Other reason	-	-	3 (2%)	3 (2%)
Radiotherapy given after R-CHOP, due to final restaging with result partial response	5/81 (6%)	5/81 (6%)	-	-
Radiotherapy given	11/81 (14%)	9/81 (11%)	133/155 (86%)	135/150 (90%)

^*a*^For 1 patient, qualification for radiotherapy was no longer further present after randomization.

^*b*^Radiotherapy after final restaging with result CR/CRu.

^*c*^After interim analysis, the observation-arms were closed and radiotherapy was performed also in observation-arms.

^*d*^Toxicity occurred during the course of R-CHOP chemotherapy.

CR/CRu = complete response/unconfirmed complete response; R-CHOP = rituximab, cyclophosphamide, doxorubicin, vincristine, and prednisone.

**Figure 2.**
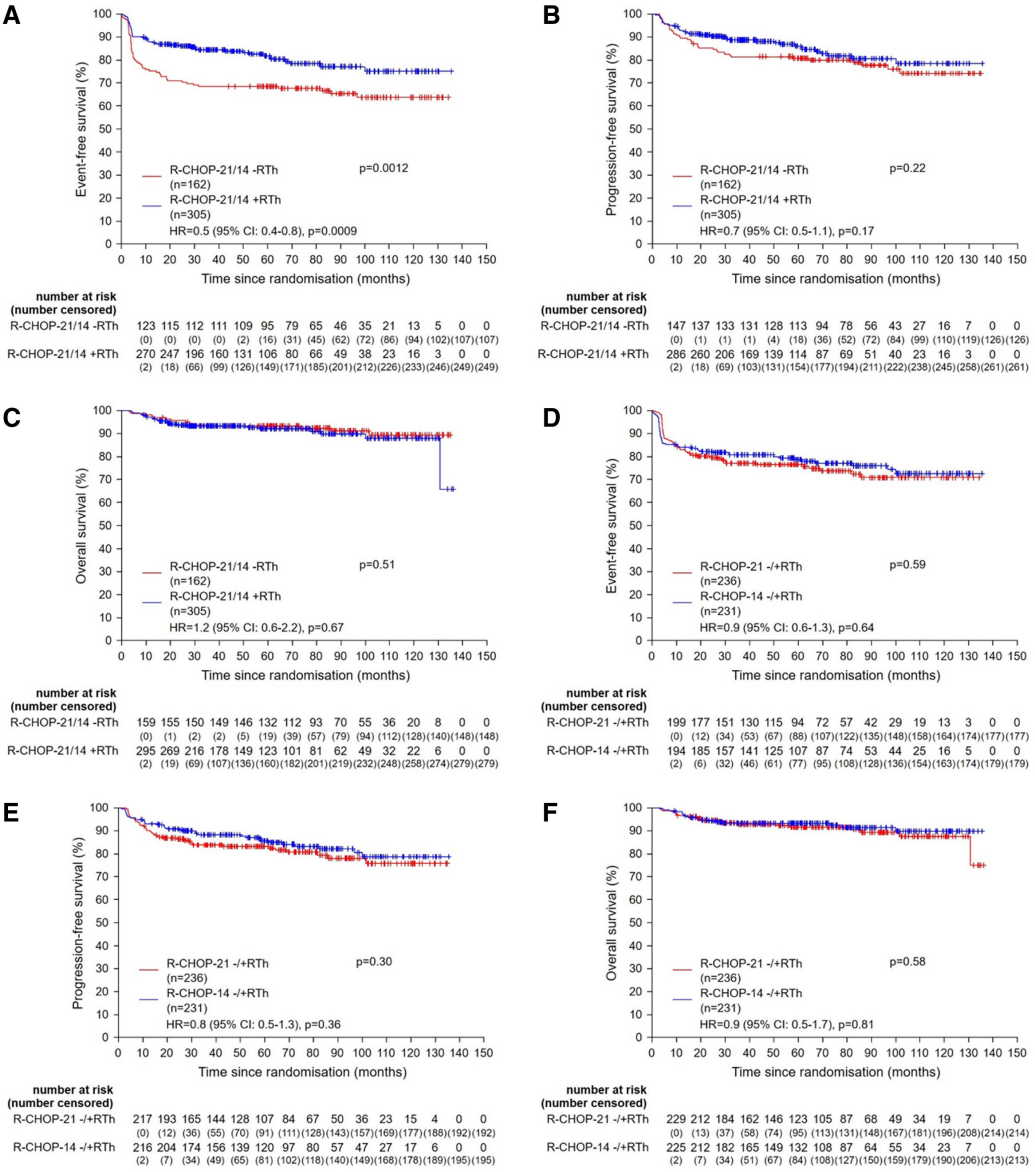
**Event-free, progression-free, and overall survival according to the therapy arm for patients qualifying for radiotherapy.** Graphs show event-free (A), progression-free (B), and overall survival (C) for patients qualifying for radiotherapy treated in radiotherapy-arm or observation-arm and event-free (D), progression-free (E), and overall survival (F) for patients qualifying for radiotherapy treated either with R-CHOP-14 or R-CHOP-21. Hazard ratios for treatment effect adjusted for strata are presented for event-free, progression-free, and overall survival. CI = confidence interval; HR = hazard ratio; PMBCL = primary mediastinal B-cell lymphoma; R-CHOP = rituximab, cyclophosphamide, doxorubicin, vincristine and prednisone.

In 467 patients qualifying for radiotherapy, dose-densification and radiotherapy were analyzed by 2 × 2 factorial testing. A planned interim analysis of the first 285 patients had revealed a significantly better EFS for patients assigned to radiotherapy (*P* = 0.004), resulting in a predefined closing of the observation-arms, with 305 patients assigned to radiotherapy (R-CHOP-21: 155 and R-CHOP-14: 150) and 162 to observation (R-CHOP-21: 81 and R-CHOP-14: 81). In the final response assessment, CR/CRu rate was 90% (274/305) in the radiotherapy-arm versus 79% (128/162) in the observation-arm. In contrast, the rate of PRs was lower in the radiotherapy-arm versus the observation-arm, 2% (7/305) versus 11% (18/162), respectively (Table 3). After a median observation of 66 months, 3-years EFS was superior in the radiotherapy-arm versus the observation-arms, 84% (95% CI, 80-89) versus 68% (95% CI, 61-76); *P* = 0.0012 (Figure 2A; Table 4). This difference was predominantly caused by the lower rate of PRs (2% versus 11%) triggering additional treatment, mostly radiotherapy in the observation-arms (Tables 2 and 3). However, after 3 years, no statistical difference was detected in PFS between radiotherapy versus observation, 89% (95% CI, 85-92) versus 81% (95% CI, 75-87); *P* = 0.22 (Figure 2B; Table 4), respectively. Three-year OS was identical in both arms, 93% (95% CI, 90-96) versus 93% (95% CI, 89-97); *P* = 0.51 (Figure 2C; Table 4). These results were confirmed in multivariable Cox regression models for EFS, PFS, and OS adjusted for the strata (Figure 2A–2C). Analyzing post hoc EFS, PFS, and OS in all 4 arms separately revealed a very similar pattern (Suppl. Figure S2A-S2C). Similar results were obtained for EFS (*P* = 0.0030), PFS (*P* = 0.37), and OS (*P* = 0.21) when the analysis was restricted to patients with extralymphatic involvement (Suppl. Figure S3A-S3C). No statistical difference was observed in PFS (*P* = 0.23) and OS (*P* = 0.078) when the analysis was restricted to patients achieving a CR/CRu after R-CHOP chemotherapy (Suppl. Figure S4A and S4B). A post hoc as-treated analysis was performed for PFS and OS. In this analysis, patients who were irradiated in the observation-arm after achieving a PR or CR/Cru were analyzed within the radiotherapy-arm (Table 2; Suppl. Figure S5A and S5B). The PFS curves showed a similar pattern (*P* = 0.14) and an overlap for OS (*P* = 0.48).

**Table 3:** Response Rates in the Final Response Assessment

Response	Qualifying for Radiotherapy (n = 467)				Not Qualifying for Radiotherapy (n = 228)	
	R-CHOP-21 (n = 81)^*a*^	R-CHOP-14 (n = 81)^*a*^	R-CHOP-21 +radiotherapy (n = 155)^*b*^	R-CHOP-14 +radiotherapy (n = 150)^*b*^	R-CHOP-21 (n = 114)^*a*^	R-CHOP-14 (n = 114)^*a*^
Complete response/unconfirmed complete response 95% CI	64 (79%) (69-87)	64 (79%) (69-87)	141 (91%) (85-95)	133 (89%) (83-94)	109 (96%) (91-99)	105 (92%) (85-96)
Complete response/unconfirmed complete response and additional treatment	2 (2%)	1 (1%)	2 (1%)	1 (1%)	1 (1%)	2 (2%)
Partial response (received additional therapy)	8 (10%)	10 (12%)	4 (3%)	3 (2%)	0 (0%)	3 (3%)
No change	0 (0%)	1 (1%)	0 (0%)	2 (1%)	0 (0%)	0 (0%)
Progressive disease	4 (5%)	2 (2%)	4 (3%)	6 (4%)	1 (1%)	0 (0%)
Therapy associated death^*c*^	0 (0%)	1 (1%)	2 (1%)	0 (0%)	0 (0%)	2 (2%)
Unknown	3 (4%)	2 (2%)	2 (1%)	5 (3%)	3 (3%)	2 (2%)

Remark: Five (0/0/1/0/2/2) patients terminated CHOP earlier due to excessive toxicity. Four patients achieved a complete remission and 1 patient deceased after cycle 1.

^*a*^Final response was assessed 2 weeks after start of the sixth cycle of R-CHOP.

^*b*^Final response was assessed after end of radiotherapy simultaneously with first follow-up.

^*c*^All deaths related to study treatment were related to R-CHOP chemotherapy (sudden cardiac arrest, traffic accident, suicide, cardiogenic shock during percutaneous coronary intervention, liver failure).

CI = confidence interval; R-CHOP = rituximab, cyclophosphamide, doxorubicin, vincristine, and prednisone.

**Table 4:** EFS, PFS and OS Rates, EFS-events and Relapse Rates

	Qualifying for Radiotherapy (n = 467)				Not Qualifying for Radiotherapy (n = 228)	
	R-CHOP-21 (n = 81)	R-CHOP-14 (n = 81)	R-CHOP-21 + Radiotherapy (n = 155)	R-CHOP-14 + Radiotherapy (n = 150)	R-CHOP-21 (n = 114)	R-CHOP-14 (n = 114)
3-y EFS (%)	65	72	83	86	81	86
95% CI (%)	(55-76)	(62-81)	(77-89)	(80-91)	(74-88)	(79-92)
3-y PFS (%)	79	84	86	91	84	91
95% CI (%)	(70-88)	(76-92)	(81-92)	(86-96)	(78-91)	(85-96)
3-y OS (%)	94	92	93	94	94	95
95% CI (%)	(89-99)	(87-98)	(88-97)	(90-98)	(89-98)	(91-99)
EFS-events						
Without event (censored)	51 (63%)	56 (69%)	126 (81%)	123 (82%)	89 (78%)	92 (81%)
Complete response/unconfirmed complete response and additional treatment	2 (2%)	1 (1%)	2 (1%)	1 (1%)	1 (1%)	2 (2%)
Complete response/unconfirmed complete response and relapse	12 (15%)	8 (10%)	13 (8%)	9 (6%)	18 (16%)	7 (6%)
Complete response/unconfirmed complete response and death	1 (1%)	0 (0%)	2(1%)	1 (1%)	2 (2%)	6 (5%)
Partial response (received additional therapy)	8 (10%)	10 (12%)	4 (3%)	3 (2%)	0 (0%)	3 (3%)
No change	0 (0%)	1 (1%)	0 (0%)	2 (1%)	0 (0%)	0 (0%)
Progressive disease	4 (5%)	2 (2%)	4 (3%)	6 (4%)	1 (1%)	0 (0%)
Therapy associated death^*a*^	0 (0%)	1 (1%)	2 (1%)	0 (0%)	0 (0%)	2 (2%)
Unknown	3 (4%)	2 (2%)	2 (1%)	5 (3%)	3 (3%)	2 (2%)
Relapse rates	12/64 (19%)	8/64 (12%)	13/141 (9%)	9/133 (7%)	18/109 (16%)	7/105 (7%)
95% CI (%)	(10-31)	(5-23)	(5-15)	(3-13)	(10-24)	(3-14)

Remark: Five (0/0/1/0/2/2) patients terminated CHOP earlier due to excessive toxicity. Four patients achieved a CR and 1 patient deceased after cycle 1.

^*a*^Áll deaths related to study treatment were related to R-CHOP chemotherapy (sudden cardiac arrest, traffic accident, suicide, cardiogenic shock during percutaneous coronary intervention, and liver failure).

CI = confidence interval; EFS = event-free survival; OS= overall survival; PFS = progression-free survival; R-CHOP = rituximab, cyclophosphamide, doxorubicin, vincristine and prednisone.

**Figure 3.**
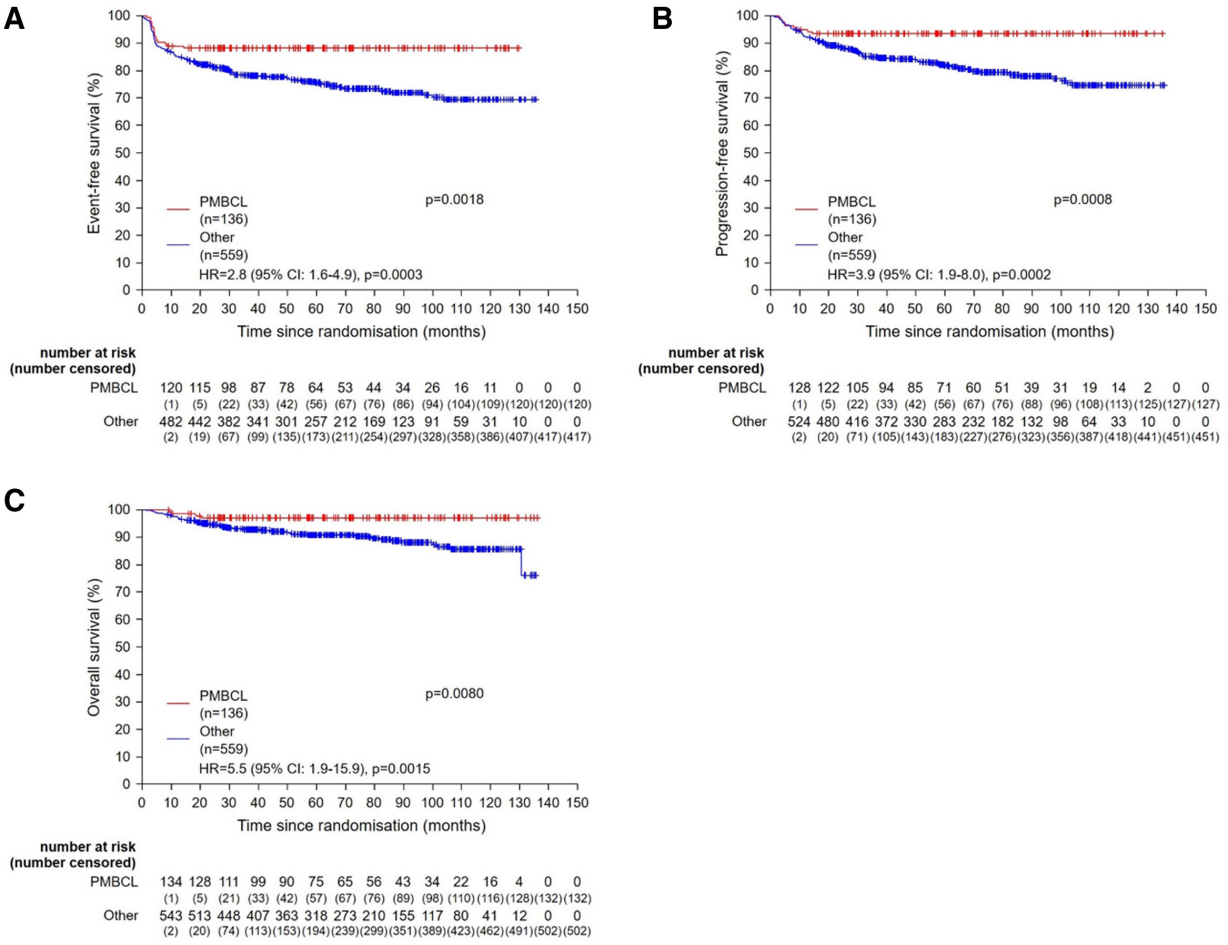
**Prognostic factors.** Graphs show event-free (A), progression-free (B), and overall survival (C) for patients with primary mediastinal B-cell lymphoma subgroup compared with all other. Hazard ratios for treatment effect adjusted for strata are presented for event-free, progression-free, and overall survival. CI = confidence interval; HR = hazard ratio; PMBCL = primary mediastinal B-cell lymphoma.

When comparing patients qualifying for radiotherapy with regard to dose-densification by a 2 × 2 factorial testing, no significant effect was observed for EFS (81% [95% CI, 76-86] versus 77% [95% CI, 72-82]; *P* = 0.59), PFS (88% [95% CI, 84-93] versus 84% [95% CI, 79-89]; *P* = 0.30), or OS (93% [95% CI, 90-97] versus 93% [95% CI, 90-96]; *P* = 0.58) after R-CHOP-14 versus R-CHOP-21 (Figure 2D–2F). These results were confirmed in multivariable Cox regression models for EFS, PFS, and OS adjusted for the strata (Figure 2D–2F). In 228 patients not qualifying for radiotherapy, there was also no difference for EFS, PFS, and OS. When comparing R-CHOP-14 with R-CHOP-21 in all 695 patients, which have been included in the trial, regardless of radiotherapy qualification, there was also no difference for EFS (82% [95% CI, 78-86] versus 78% [95% CI, 74-83]; *P* = 0.46), PFS (89% [95% CI, 86-93] versus 84% [95% CI, 80-88]; *P* = 0.17), and OS (94% [95% CI, 91-97] versus 93% [95% CI, 90-96]; *P* = 0.73). In an analysis of prognostic factors in the univariate and multivariable analysis, the subgroup of PMBCL 136 of 695 patients (20%) was associated with a distinct, very favorable outcome with a superior 3-year EFS 88% (95% CI, 83-94) versus 78% ([95% CI, 75-82]; *P* = 0.0018), PFS 93% (95% CI, 89-98) versus 85% ([95% CI, 82-88]; *P* = 0.0008), and OS 97% [95% CI, 94-100] versus 93% [95% CI, 90-95]; *P* = 0.0080) (Figure 3A–3C). We subsequently performed a sensitivity analysis restricted to patients qualifying for radiotherapy and excluding patients with PMBCL, defining a subgroup with a more unfavorable prognostic profile. Again, a significant superior 3-year EFS was observed in the radiotherapy-arms versus observation-arms, 81% (95% CI, 76-86) versus 65% (95% CI, 56-73); *P* = 0.010, but not in PFS (86% [95% CI, 82-91] versus 78% [95% CI, 70-85]; *P* = 0.27) and OS (92% [95% CI, 88-95] versus 92% [95% CI, 87-97]; *P* = 0.44) (Suppl. Figure S6A-S6C). No differences between R-CHOP-14 and R-CHOP-21 were observed for EFS, PFS, and OS (Suppl. Figure S6D-S6F).

The safety population included 467 patients qualifying for radiotherapy and 228 patients not qualifying for radiotherapy (Table 5). Regarding common toxicity classification (CTC) grade 3 of 4 hematological toxicity during immunochemotherapy, more patients in the R-CHOP-14 than in the R-CHOP-21 arms experienced anemia 24 of 316 (8%) versus 12 of 321 (4%) whereas leukocytopenia was less common in R-CHOP-14 arms 79 of 182 (43%) as compared with the R-CHOP-21 arms 97 of 168 (58%). With respect to nonhematological events, no relevant difference in polyneuropathy, infections, and cardiac toxicities between the arms were reported in >5% of patients.

**Table 5:** Toxicity of Chemoimmunotherapy per Patient

CTC Grade 3/4	Qualifying for Radiotherapy (n = 467)				Not Qualifying for Radiotherapy (n = 228)	
	R-CHOP-21 (n = 81)	R-CHOP-14 (n = 81)	R-CHOP-21 + Radiotherapy (n = 155)	R-CHOP-14 + Radiotherapy (n = 150)	R-CHOP-21 (n = 114)	R-CHOP-14 (n = 114)
Leukocytopenia^*a*^	31/43 72%	22/44 50%	45/76 59%	36/83 43%	21/49 43%	21/55 38%
Thrombocytopenia	3/76 4%	3/75 4%	3/142 2%	0/137 0%	2/99 2%	1/103 1%
Anemia	1/76 1%	3/76 4%	9/145 6%	10/136 7%	2/100 2%	11/104 11%
Arrhythmia	0/75 (0%)	0/74 (0%)	0/149 (0%)	2/141 (1%)	2/105 (2%)	0/112 (0%)
Cardiac functions	0/73 (0%)	0/72 (0%)	1/149 (1%)	2/138 (1%)	0/105 (0%)	0/112 (0%)
Sensory	3/75 (4%)	4/76 (5%)	10/150 (6%)	4/141 (3%)	3/103 (3%)	8/111 (7%)
Infection	6/79 (8%)	12/78 (15%)	13/152 (9%)	15/144 (10%)	8/105 (8%)	12/113 (11%)

Remark: Nonhematological toxicities are outlined if observed in 5% of patients or more. Cardiac toxicity is outlined additionally.

^*a*^Based on the blood counts within nadir interval day 11–14 (R-CHOP-21) and day 8–10 (R-CHOP-14).

CTC = common toxicity criteria; R-CHOP = rituximab, cyclophosphamide, doxorubicin, vincristine and prednisone.

Additional radiotherapy was generally very well tolerated. Depending on the irradiated region, only a few (1%–3%) CTC grade 3 of 4 acute toxicities in the form of mucositis and esophagitis occurred. Other severe side effects were even less common (Table 6).

**Table 6:** Acute Toxicity of Radiotherapy

CTC Grade ¾	R-CHOP-21/14 With Radiotherapy (n = 268)^*a*^
Hemoglobin	0/172 (0%)
Leucocytes	5/172 (3%)
Platelets	1/172 (1%)
Nausea	1/192 (0·5%)
Vomiting	0/198 (0%)
Diarrhea	1/198 (0·5%)
Esophagitis/dysphagia	7/209 (3%)
Constipation	0/196 (0%)
Mucous membranes/mucositis	3/201 (1%)
Salivary glands	0/200 (0%)
Arrhythmia	0/188 (0%)
Cardiac function	0/186 (0%)
Dyspnea	0/198 (0%)
Larynx	1/178 (1%)
Hematuria	0/172 (0%)
Sensory	0/167 (0%)
Mood	0/174 (0%)
Otitis	0/173 (0%)
Keratitis	0/173 (0%)
Nose/sense of smell	0/174 (0%)
Skin/subcutis local	1/189 (0·5%)
Infection	2/181 (1%)

^*a*^Patients who received radiotherapy.

CTC = common toxicity criteria; R-CHOP = rituximab, cyclophosphamide, doxorubicin, vincristine and prednisone.

In patients qualifying for radiotherapy, 3 treatment-related deaths occurred, 2 in the radiotherapy and 1 in the observation-arm, all occurring during chemotherapy. In patients not qualifying for radiotherapy, 1 treatment-related death occurred (Table S6). Sixteen (3%) of 467 patients qualifying for radiotherapy developed secondary neoplasia and 16 (7%) of 228 patients not qualifying for radiotherapy developed secondary neoplasia (Table S7).

In patients treated within the UNFOLDER trial, 130 restaging F-18 fluordesoxyglucose positron emission tomography/computed tomography (FDG/PET) were performed by local-physicians’ choice, raising questions of bias by PET-based. These FDG/PETs were mostly performed in patients randomized to the radiotherapy-arm (92/130; 70.1%), and most concerned (99/130; 76%) restaging after completed chemotherapy. The majority of these FDG-PETs were evaluated as negative (89/130; 68.5%). In particular, in case of PET-negativity, irradiation was still performed in the radiotherapy-arm in most of the cases, and even in case of PET-positivity, irradiation was mostly omitted in the observation-arm. Thirteen PET-adapted therapy changes were identified, 12 of them based on the positive PET results. Ten of these 13 (77%) PET-adapted therapeutic changes occurred in the observation-arm, and 3 in radiotherapy-arm, in which there was also the only PET-adapted omission of radiotherapy for reasons of metabolic CR and initially resected bulk.

## DISCUSSION

The UNFOLDER trial aimed to improve outcome in patients, ≤60 years of age, with aggressive B-cell Non-Hodgkin’s lymphoma and an intermediate risk profile defined by one risk factor according to the aaIPI or bulky/extralymphatic disease without any risk factor. To our knowledge, this is the only prospective, randomized study evaluating the efficacy of consolidation radiotherapy in this patient population. Radiotherapy to bulky or extralymphatic disease improved EFS, but had no significant impact on PFS and OS (Figure 2A–2C). The inferior EFS rate of the observation-arm was caused by more events due to a higher PR rate triggering additional treatment, mostly radiotherapy. This was also observed when the distinct pathogenetic subtype of PMBCL was excluded from the analysis, focusing on the subgroup of patients with an inferior prognosis (Suppl. Figure S6 and S7). Also, results were consistent in patients, who presented with extralymphatic involvement (Suppl. Figure S3). Dose-densification of R-CHOP-21 by reducing the treatment interval to R-CHOP-14 did neither improve EFS, PFS, nor OS (Figure 2D–2F).

The observation of an inferior EFS but identical and favorable OS in the observation-arm allows the conclusion that radiotherapy to bulky (Suppl. Figure S8) or extralymphatic disease might be spared in patients, who are responding to 6 cycles of R-CHOP with a CR/CRu. Indeed, no statistical difference was observed in PFS nor OS when the analysis was restricted to patients achieving a CR/CRu after R-CHOP (Suppl. Figure S4A and S4B). Also in the LYSA 02-03 trial, patients of non-bulky, stage I/II disease in CR documented by FDG-PET after 4–6 cycles of R-CHOP were randomized to consolidation radiation or observation and there was no difference in EFS or OS.^16^ However, the study represents a population with a very favorable prognosis, whose outcome can be hardly improved. In the intention-to-treat population of the UNFOLDER study, radiotherapy of patients achieving only a PR in the observation-arm resulted in an identical survival compared with those patients who had received radiotherapy obligatorily in the radiotherapy-arm, irrespective of response. Indeed, response to treatment in aggressive lymphoma is highly predictive of survival. Patients with PET-positive as well as PET-negative residual masses after immunochemotherapy have an inferior prognosis.^17^ Thus, radiotherapy of patients in PR may have leveled the negative prognostic impact of the residual masses.

Limitations of the UNFOLDER trial concern the response assessment. First, restaging was performed later (after radiation) in the radiotherapy-arms compared with observation-arms, what might has contributed to a time-based diagnostic assessment bias, as the effects of immunochemotherapy just had longer time to appear. However, the main limitation of this trial is that response assessment was done according to the International Workshop to Standardize Response Criteria for Non-Hodgkin’s Lymphomas published in 1999, which does not require a FDG-PET scan.^14^ Until very recently, FDG-PET scan has not been reimbursed for initial staging and/or response assessment of aggressive lymphoma in Germany, where most of the patients had been recruited. Meanwhile, response assessment was changed by the Lugano Classification making a FDG-PET scan mandatory to distinguish metabolically between CR and PR.^18^ Only an appropriately powered, large scale, randomized trial can provide the scientific evidence to support the hypothesis that radiotherapy to bulky or extralymphatic lymphoma manifestations in patients in PR after R-CHOP improves their outcome, when response is assessed by FDG-PET scan. So far, only indirect evidence exists. In a retrospective study 196 patients with advanced-stage DLBCL, who had residual abnormalities on CT scan following R-CHOP, received consolidative radiation to sites of FDG-PET positivity, when feasible.^19^ Patients thus treated with consolidative radiotherapy had similar outcomes compared with those with negative scans. These observations could already be supported by the interim analysis of the OPTIMAL>60 trial (EudraCT-No. 2010-019587-36).^20^ In this study, historic data are used for comparison from the very similar RICOVER-60 study, which found that individuals aged >60 years benefit from irradiating former lymphoma bulk after 6 cycles of R-CHOP-14.^11^ According to the OPTIMAL>60 interim analysis, consolidating radiotherapy in the case of PET-positive bulk seems to improve the prognosis while its avoidance in PET-negative bulks did not compromise outcome.

In the prospective UNFOLDER trial, radiotherapy improved EFS, but had no statistical significant impact on PFS and resulted in an identical OS also in the subgroup of 242 patients with extralymphatic involvement (Suppl. Figure S3), contrary to retrospective analysis. Particularly, a monocentric analysis of 469 patients with DLBCL reported a 23% higher 5-year PFS associated with consolidation radiotherapy.^21^ A further retrospective analysis in extranodal stage I DLBCL demonstrated better PFS and OS after consolidative radiotherapy.^13^ However, the benefit was no longer observed in patients, who presented with negative FDG-PET after immunochemotherapy. In both studies, patients with extranodal involvement have been included irrespective of the site, which limits conclusions with regard to the relevance of radiotherapy for a specific organ. A SEER database analysis of primary aggressive lymphoma of the breast demonstrated improved survival in patients, who have been treated with consolidative radiotherapy.^22^ A pooled retrospective study of patients with bone involvement from 9 consecutive trials of the German High-Grade Non-Hodgkin’s lymphoma Study Group revealed an improved EFS after consolidation radiotherapy without affecting OS.^12^ However, these retrospective analyses do not allow final conclusions concerning a response adapted application of radiotherapy to extralymphatic sites.

FDG/PETs were performed by local-physicians’ choice for patients treated within the UNFOLDER trial raised questions of a potential PET-based bias, but overall, the performed FDG-PETs had little impact on the choice of further therapy in the UNFOLDER trial.

The still relatively limited experience and data on PET-adapted therapy at the time the UNFOLDER trial was conducted might has contributed to this few number of more PET-adapted therapy decisions.

Dose-densification from R-CHOP-21 to R-CHOP-14 did not lead to a superior outcome. This result is in line with 3 previous randomized studies.^23–25^ Thus, both regimens are equally effective and equally toxic. The increased rate of leukocytopenia observed in patients treated with R-CHOP-21 can be explained by the less commonly use of G-CSF support in this regimen. The choice of regimen depends on the oncologist’s discretion and the patient’s preference, as R-CHOP-21 is more widely used and R-CHOP-14 allows a significant reduction of treatment duration and therefore an earlier return to an unrestricted private and professional life.

Since the introduction of rituximab, numerous randomized trials failed to improve the efficacy of the R-CHOP-21 regimen.^23,24,26–33^ In the POLARIX study, pola-R-CHP, in which vincristine was replaced with polatuzumab vedotin, improved PFS but not OS as compared with standard R-CHOP.^34^ Only 1 single study, the LNH03-2B trial, showed improved EFS, PFS, and OS in the R-ACVBP arm when compared with R-CHOP-21 in a very similar population as in the UNFOLDER trial of younger patients with 1 risk factor according to the IPI.^10^ This trial provides evidence that therapy of aggressive lymphoma can still be improved by modifying the chemotherapy backbone.

Other limitations of the UNFOLDER trial are that the treatment arms are not equally balanced due to the early stop of randomization for radiotherapy by the Data and Safety Monitoring Committee. Hence, the arms without radiotherapy were closed and radiotherapy was given to all bulky and extralymphatic sites for the remaining participants. This decision was based on the analysis of EFS as the primary end point. In this light, PFS has been proposed as the preferred end point in lymphoma clinical trials, at a time when the UNFOLDER study was already initiated.^35^ Also generalizability of the data is limited by the narrow inclusion criteria, which only targeted patients with aaIPI of 1 or bulky aaIPI 0.

Regarding the data of the UNFOLDER trial in light of new immunotherapies for relapsed/refractory DLBCL and PMBCL like CAR-T cells, bispecific antibodies or the combination of PD1-blockade combined with brentuximab for PMBCL, these improved salvage therapy options might argue for less intensive front-line therapies.^36–41^ However, for instance, in the ZUMA-7 trial, the 24-months EFS has very much improved compared with former SOC (HR, 0.4), but still reaches only 41%, what highlights the still very relevant point of an efficient first-line therapy.

In conclusion, treatment of young patients with aggressive B-cell lymphoma and an intermediate risk profile is not improved by dose-densification of R-CHOP-21 to R-CHOP-14.

Application of consolidation radiotherapy to bulky and extralymphatic disease is improving EFS without affecting PFS and OS. Although the EFS was improved in patients who received radiotherapy, this was largely due to the unplanned administration of radiotherapy in the observation-arm due to lack of CR but since this was assessed by CT with 1999 criteria, in the modern era, the role of radiotherapy in favorable/intermediate DLBCL patients with bulky/extranodal disease remains undefined.

## ACKNOWLEDGMENTS

The trial was supported by a grant from the German nonprofit foundation, Deutsche Krebshilfe (German Cancer Aid), reference number 106377 and Chugai Pharmaceuticals. We thank the patients, families, caregivers, and principal investigators of all countries who participated in the clinical trial including Peter de Nully Brown (Denmark); Massimo Federico and Francesco Merli (Italy); Ofer Shpilberg (Israel); and Michael Pfreundschuh (died in March 2018; Germany), who designed the study, wrote the protocol, was the principal investigator in Germany, and was chairman of the study. We thank the numerous research and trial groups, including the German High-Grade Non-Hodgkin’s Lymphoma Study Group, the Fondazione Italiana Linfomi, and the Nordic lymphoma group for their participation in the trial. We thank the international board of expert pathologists who provided histopathological review: Andreas Rosenwald (chairman; Wuerzburg, Germany); Alfred C Feller (Luebeck, Germany); Martin-Leo Hansmann (Frankfurt, Germany); Wolfram Klapper (Kiel, Germany); Peter Moeller (Ulm, Germany); Hans Konrad Mueller-Hermelink (Wuerzburg, Germany); Elisabeth Ralfkiaer (Copenhagen, Denmark); Harald Stein (Berlin, Germany); Philippe Trougouboff (Afula, Israel); and Hans-Heinrich Wacker (Kiel, Germany). We thank Christian Berdel, Jochen Fleckenstein, and Christian Ruebe for imaging review, and as independent expert for radiotherapy and radiation oncology at Homburg/Saar, Germany. We thank the data and safety monitoring committee that served as an independent expert advisory group to assess safety and efficacy data during the trial, including Günter Brittinger (Essen, Germany, died June, 2021); Volker Diehl (Koeln, Germany); and Klaus Havemann (Marburg, Germany; died May, 2016). We thank the study trial office Homburg/Saar, Germany, including the secretaries Waltraud Beck (died August, 2013) and Daniela Ehlert; the data management team Stephanie Angel, Elina Dick, Kerstin Höhn, Kirstin Monz, Tanja Rixecker, and Christian Schorpp; and the clinicians Josif Amam, Konstantinos Christofyllakis, Gerhard Held, Niels Murawski, Milena Pfeiffer, Viola Poeschel, Christoph Renner, Rudolf Schmits (died October, 2016), Jörg Schubert, Pia Sweet, Anne Wolf, and Carsten Zwick. We thank the data centre in Leipzig, Germany, including the database team Sigrid Haupt, Jürgen Hentschel, Martina Kunert, Beate Mann, Katja Rillich, Ulrike Schoenwiese, and Barbara Wicklein; and the biometry team Bettina Altmann, Markus Loeffler, and Marita Ziepert.

## AUTHOR CONTRIBUTIONS

GH and VP oversaw the study (originally designed by Michael Pfreundschuh) and contributed to study design, data monitoring, data interpretation, and writing and approval of the report. L. Thurner contributed to data interpretation and wrote the article. ML and MZ did the statistical analysis and contributed to study design, data interpretation, and writing and approval of the report. CB and JF coordinated and assessed the imaging review and radiation oncology review and contributed to data interpretation. ME and HS represented the reference radiotherapy panel. AR coordinated the reference pathology. OS was the principal investigator in Israel and recruited patients. MF and FM were the principal investigator in Italy and recruited patients. PdNB was the principal investigator in Denmark and recruited patients. NM contributed to study oversight and data monitoring. M. Bewarder and SS contributed to data interpretation and recruited patients. ME contributed to study oversight. MN, GW, BG, NS, FH, and L. Truemper contributed to study design and recruited patients. BA contributed to statistical analysis and data interpretation. HS contributed to data interpretation. GH, CS, PB, DK-M, AV, MW-H, JD, MH, BM, EL, UBK, NF, TG, FG, R. Mahlberg, R. Marks, H-WL, MS, LFvW, MK, ER, M. Bentz, BK-S, and RT recruited patients. All authors have reviewed and approved the final version of the report.

## DATA SHARING STATEMENT

Data of results reported in this article will be shared after de-identification. Individual, pseudonymised data as well as data dictionaries will be available upon request up to 5 years after publication after providing a data sharing agreement directed to dshnhl@uks.eu, which describes intended analyses and required data. Selected data will be also available on the Leipzig Health Atlas (www.health-atlas.de).

## DISCLOSURES

L. Thurner has received travel grants from Abbvie, Janssen and EUSA-Pharm, and has indicated consultancy for Takeda, Astra-Zeneca, Merck, EUSA-pharm. DK-M has received personal fees from Novartis, Astra-Zeneca, Gilead Sciences, GlaxoSmithKline, Janssen-Cilag, and nonfinancial support from Gilead Sciences, Janssen-Cilag, Takeda, Novartis. AV has received honoraria from Roche, Amgen, Kite, Gilead, Novartis, Bristol-Myers Squibb and has indicated a membership of the advisory board of Roche, Amgen, Kite, Gilead, Novartis, Bristol-Myers Squibb. UBK has received honoraria and advisory fees from Roche, Janssen-Cilag, Takeda, Bristol-Myers Squibb, Gilead, Hexal, Pfizer, Astra- Zeneca, Pentixapharm, Amgen, Novartis, MSD and has received clinical study support for Celgene, Takeda, BMS, Roche, Astra-Zeneca, Novartis, MSD, Janssen-Cilag, Pfizer. MN has received travel grants from Roche, Celgene and MSD and personal fees from Roche, Celgene, MSD, Janssen, Amgen, Incyte and Abbvie. FG participates in advisory board of Roche, Boehringer Ingelheim, Abbvie, Merck, Takeda, MSD, Sanofi, Pfizer, Novartis, Amgen and Janssen. RT has received grants from Atara and Roche and travel support from Roche, Atara, Celgene, Janssen and Abbvie; he has indicated a membership of the advisory board of Atara and Abbvie and has indicated consultancy for s Atara. PdNB has indicated consultancy for Roche, Incyte and Novartis. FM has received travel grants from Roche, Takeda, Janssen and Gilead and has indicated consultancy and advisory role for Roche, Gilead, Janssen, MSD, Takeda and Novartis. SS has received grants from Abbvie, Astra-Zeneca, Celgene, Gilead, Roche, Janssen, Novartis, Morphosys and has indicated consultancy for for Abbvie, Astra-Zeneca, Celgene, Gilead, Roche, Janssen, Novartis, Morphosys; he has received drug/equipment supplied by entity from Abbvie, Astra-Zeneca, Celgene, Gilead, Roche, Janssen, Novartis, Morphosys. VP has received grants from Deutsche Krebshilfe (German Cancer Aid), Chugai, Abbvie, Amgen, Roche and Bristol Myers Squibb. GH has received grants from Roche and Bristol Myers Squibb and personal fees from Bristol Myers Squibb, Roche, Amgen, Spectrum and MSD. All the other authors have no conflicts of interest to disclose.

## Supplementary Material


