# Supplementary material for: Radiation and Dose-densification of R-CHOP in Aggressive B-cell Lymphoma With Intermediate Prognosis: The UNFOLDER Study
Source: Hemasphere. 2023 Jul 5;7(7):e904. doi: 10.1097/HS9.0000000000000904 (PMC10325769; doi:10.1097/HS9.0000000000000904)
Supplement: Supplementary file 1 [file hs9-7-e904-s001.pdf]

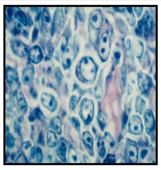

DEUTSCHE STUDIENGRUPPE HOCHMALIGNE NON-HODGKIN-LYMPHOME*
German High Grade Non-Hodgkin's Lymphoma Study Group
*(gefördert durch die Deutsche Krebshilfe)

Randomisierte Studie zum Vergleich einer kombinierten Immunochemotherapie mit 6 Zyklen des monoklonalen anti-CD20-Antikörpers Rituximab in Kombination mit 6 Zyklen einer Chemotherapie mit CHOP (Cyclophosphamid, Doxorubicin, Vincristin und Prednison)

in 21-tägigen Intervallen oder 14-tägigen Intervallen

jeweils mit und ohne konsolidierende Strahlentherapie von großen Tumormassen und/oder Extranodalbefällen

bei Patienten mit aggressiven $CD20^{+}$ B-Zell-Lymphomen

im Alter von 18-60 Jahren

mit altersadaptiertem IPI = 1 (alle) oder IPI = 0 mit großen Tumormassen (Durchmesser ≥ 7.5 cm)

Kurztitel: UNFOLDER 21/14-Studie

Studiennummer: DSHNHL 2004-3

Eudra CT-Nr: 2005-005218-19

Schriftführung: M. Pfreundschuh
mit Unterstützung von M. Ziepert, C. Rübe, N. Schmitz und F. Hartmann

Version 4.0 vom 22.11.12 positiv begutachtet durch die Ethikkommission
der Ärztekammer des Saarlandes am 25.02.2013

Version 3.2.2 vom 06.02.2006 positiv begutachtet durch die Ethikkommision
der Ärztekammer des Saarlandes am 02.03.2006

Version 3.2.1 positiv begutachtet durch die Ethikkommission
der Ärztekammer des Saarlandes am 07.10.2005

Version 3.2. mit Amendment positiv begutachtet durch die Ethikkommission
der Ärztekammer des Saarlandes mit Votum vom 18.08.2005

Version 3.1 positiv begutachtet durch die Ethikkommission
der Ärztekammer des Saarlandes mit Votum vom 30.09.2004

**Studienleitung DSHNHL 2004-3**	**Studiensekretariat der DSHNHL**	**Biometrie der DSHNHL**
Prof. Dr. M. Pfreundschuh	Prof. Dr. M. Pfreundschuh,<br>PD. Dr. G. Held,<br>Dr. N. Murawski<br>V. Pöschel	Prof. Dr. M. Löffler,<br>Dr. M. Ziepert
Universitätsklinikum des Saarlandes<br>Innere Medizin I<br>Kirrberger Strasse, Geb. 40<br>66424 Homburg/Saar	Universitätsklinikum des Saarlandes<br>Innere Medizin I<br>Kirrberger Straße, Geb. 40<br>66424 Homburg/Saar	Institut f. Medizinische Informatik, Statistik und Epidemiologie der Universität Leipzig (IMISE)<br>Härtelstr. 16-18<br>04107 Leipzig
Tel.:(06841) 16-23002<br>Fax: (06841) 16-23101<br>E-Mail: michael.pfreundschuh@uks.eu	Tel.: (06841) 16-23084 (-21306/-21303)<br>Fax: (06841) 16-23004<br>E-Mail: dshnhl@uks.eu	Tel.: (0341) 97-16120<br>Fax: (0341) 97-16119<br>E-Mail: marita.ziepert@imise.uni-leipzig.de

Inhaltsverzeichnis

0 Allgemeines ... 6

0.1 Verantwortliche Personen DSHNHL 2004-3 ... 6

0.2 Studienleitkommission der DSHNHL ... 7

0.2.1 Vorstand der DSHNHL ... 7

0.2.2 Weitere Mitglieder der Studienleitkommission ... 7

0.2.3 Scientific Advisory Board ... 7

0.3 Protokollkomitee und Daten- und Sicherheits- Monitoringkomitee (DSMK) ... 8

0.3.1 Protokollkomitee ... 8

0.3.2 DSMK ... 9

0.3.3 GCP-Konformität ... 9

0.4 Präambel zum Amendment ... 10

0.5 Protokoll-Synopse DSHNHL 2004-3 ... 11

0.6 Flussdiagramm der Studie 2004-3 ... 12

0.7 Therapielan und durchzuführende Untersuchungen ... 13

0.8 Checkliste für den Arzt ... 14

0.8.1 Was ist vor Therapiebeginn zu tun ? ... 14

0.8.2 Durchführung der Therapie ... 14

0.8.3 Was ist bzgl. der konsolidierenden Strahlentherapie zu beachten? ... 14

0.8.4 Was ist nach Therapieende zu tun? ... 15

0.8.5 Was ist bei Progress, NC oder Rezidiv zu tun? ... 15

0.8.6 Was ist bei vorzeitigem Therapieende zu tun? ... 15

0.8.7 Was ist bei einem Schwerwiegenden Unerwünschten Ereignis (SUE) zu tun? ... 15

0.8.8 Was ist bei einem Todesfall zu tun? ... 15

0.8.9 Was ist bei einem Arztwechsel/Klinikwechsel zu tun? ... 15

0.9 Änderungen im Vergleich zu dem Protokoll der zweiten Studiengeneration (High-CHOEP-Studie) ... 16

0.10 Änderungen im Amendment vom 22.11.12 ... 16

1 Ziele der Studie ... 17

1.1 Primäres Ziel der Studie ... 17

1.2 Sekundäre Ziele der Studie ... 17

2 Begründung und Rationale der Studie ... 19

2.1 Stand der Forschung ... 19

2.2 Begründung für die Patientenzielgruppe für DSHNHL 2004-3 ... 24

2.3 Standard-Regime/Kontrollarm ... 24

2.4 Begründung des Therapieoptimierungsversuchs ... 25

2.4.1 Intensivierung der Chemotherapie ... 25

2.4.2 Stellenwert der Strahlentherapie ... 27

2.4.3 Strahlentherapiedosis ... 29

2.4.4 Definition von Bulky Disease 29
2.4.5 Definition von qualifiziertem Extranodalbefall 30
2.4.6 Zusammenfassende Begründung der Studie 30

3 Studienplan 31

3.1 Studiendesign 31

3.2 Teilnehmende Institutionen und Zahl der Patienten 31

3.3 Dauer der Studie 31

3.4 Abbruch der Studie 31

3.4.1 Abbruch der Studie für einzelne Patienten 31
3.4.1.1 Nachträgliche Nicht-Qualifikation 31
3.4.1.2 Abbruch der Therapie 32
3.4.2 Abbruch der Studie bzw. einzelner Studienarme 32

4 Aufnahmekriterien 33

4.1 Einschlusskriterien 33

4.2 Ausschlusskriterien 34

5 Individueller Studienablauf 35

5.1 Durchführung der Staging-Untersuchungen 35

5.1.1 Obligate Untersuchungen 35
5.1.2 Fakultative Untersuchungen 35

5.2 Stadieneinteilung des Patienten/Risikozuordnung 36

5.3 Zentrale Beurteilung der prätherapeutischen bildgebenden Diagnostik ... 36

5.4 Aufklärung des Patienten über die Studie 36

5.5 Meldung des Patienten für die Studie 37

5.6 Aufnahme des Patienten in die Studie 38

5.7 Durchführung der Studientherapie 39

5.7.1 Vorphase-Therapie 40
5.7.2 Chemotherapie 40
5.7.2.1 Dosisreduktion 41
5.7.2.2 Rituximab-Applikation 42
5.7.2.3 ZNS-Prophylaxe/RX bei testikulärem Befall 42
5.7.2.4 G-CSF-Therapie 43
5.7.2.5 Nebenwirkungen der eingesetzten Therapiemodalitäten 43
5.7.2.6 Supportive Maßnahmen 44
5.7.2.7 Kontrolluntersuchungen unter Therapie 44
5.7.3 Konsolidierende Strahlentherapie 44
5.7.3.1 Indikation zur konsolidierenden Strahlentherapie 44
5.7.3.2 Beginn der konsolidierenden Strahlentherapie 45
5.7.3.3 Gerätetechnische Voraussetzungen für eine protokollgerechte konsolidierende Strahlentherapie 45
5.7.3.4 Bestrahlungsvolumina 46
5.7.3.5 Dokumentation der Strahlentherapie 47

5.8 Restaging- und Nachsorgeuntersuchungen 47

5.8.1 Zwischenstaging 47
5.8.2 Abschlussstaging nach Chemotherapie 47
5.8.3 Remissionsbestätigung 47
5.8.4 Zusatzstaging nach Strahlentherapie durch den Strahlentherapeuten (nicht obligat) 48

5.8.5 Restaging bei vorzeitiger Beendigung der Therapie 48
5.8.6 Nachsorgeuntersuchungen 48
5.9 Erhebung der Zielkriterien 48
5.9.1 Komplette Remission (CR) 49
5.9.2 Komplette Remission mit verbleibender Unsicherheit (CRu) 49
5.9.3 Partielle Remission (PR) 49
5.9.4 No Change (NC) 50
5.9.5 Progress (PRO) 50
5.9.6 Rezidiv 51
5.9.7 Beurteilung von nicht messbarem Tumor oder Knochenbefall 51

6 Feststellung der Sicherheit/Unerwünschte Ereignisse 52
6.1 Feststellung der Sicherheit 52
6.2 Unerwünschtes Ereignis (UE) 52
6.2.1 Definition erwartetes/unerwartetes Unerwünschtes Ereignis 52
6.2.2 Erfassung der erwarteten/unerwarteten Unerwünschten Ereignisse 53
6.3 Schwerwiegendes Unerwünschtes Ereignis (SUE) 53
6.3.1 Definition Schwerwiegendes Unerwünschtes Ereignis 53
6.3.2 Erfassung der Schwerwiegenden Unerwünschten Ereignisse 53

7 Biometrische Aspekte der Studie 54
7.1 Randomisationsalgorithmus 54
7.2 Endpunkte der Studie 55
7.2.1 Primärer Endpunkt – Hauptzielkriterium 55
7.2.2 Sekundäre Endpunkte – Nebenzielkriterien 55
7.2.2.1 Nebenzielkriterien bezüglich Wirksamkeit 55
7.2.2.2 Nebenzielkriterien bezüglich Sicherheit 56
7.2.2.3 Nebenzielkriterien zur Gesundheitsökonomie 56
7.2.2.4 Nebenzielkriterien zur Protokolladhärenz 56
7.2.2.5 Nebenzielkriterien zur konsolidierenden Strahlentherapie 56
7.3 Statistische Formulierung der Studienfrage und Fallzahlberechnung 56
7.4 Zwischenauswertungen und Abbruchkriterien 58
7.5 Geplante Analysemethoden 59
7.5.1 Definition des Auswertungskollektivs 59
7.5.2 Analyse zur Wirksamkeit und Sicherheit 60
7.5.2.1 Hauptzielkriterium 60
7.5.2.2 Nebenzielkriterien 60

8 Dokumentation und Monitoring 61
8.1 Aufbau der Dokumentationsmappe 61
8.2 Bearbeitung der ausgefüllten Dokumentationsbögen 61
8.3 Monitoring vor Ort 62

9 Referenzbeurteilungen 63
9.1 Referenzpathologische Beurteilung 63
9.2 Referenzstrahlentherapeutische Beurteilung 64

10 Wissenschaftliche Begleitprojekte und krebshilfe verbundprojekt „molekulare mechanismen bei malignen lymphomen“ 65

11 Ethische Grundlagen 67

12 Organisation 68

12.1 Datenverarbeitung und Archivierung 68

12.2 Nachträgliche Protokolländerungen..68

12.3 Finanzierung und Versicherungen 68

12.4 Publikationsvereinbarungen 69

13 Literaturverzeichnis 70

14 Anhang 73

14.1 Formular der Teilnahmeerklärung der Zentren 73

14.2 Patienteninformationen und Patientenaufklärung 75

14.2.1 Patienteninformation zur Studie DSHNHL 2004-3 (UNFOLDER) 75

14.2.2 Patienteninformation zum Datenschutzkonzept des Kompetenznetz Maligne Lymphome (KML) 84

14.2.3 Patienteninformation – Daten des aufklärenden Arztes 87

14.3 Protokoll der Patientenaufklärung 88

14.4 Patienten-Einverständniserklärung 89

14.5 Liste der teilnehmenden Zentren 93

14.6 Adressen der Mitglieder des Protokollkomitee und des DSMK 104

14.7 Voten der Ethikkommission 107

14.8 Versicherung 112

14.9 Definition – Stadium und Extranodalbefall 115

14.9.1 Stadieneinteilung 115

14.9.2 Extranodalbefall (E) 116

14.9.3 Definition Allgemeinsymptome 116

14.10 Definition - Bulk-Befall 117

14.11 Definition – Allgemeinzustand – ECOG 117

14.12 Internationaler Prognostischer Index 117

14.13 Liste der Referenzzentren für Lymphknotenpathologie zur Studie DSHNHL 2004-3 (UNFOLDER) 118

14.14 Definition – NCI-CTC Toxizitätskriterien 119

14.15 Dokumentationsbögen 126

14.15.1 Webbasiert 126

14.15.2 Konventionell 126

0 Allgemeines

0.1 Verantwortliche Personen DSHNHL 2004-3

Studienleiter	Prof. Dr. med. M. Pfreundschuh<br>Universitätsklinikum des Saarlandes<br>Innere Medizin I, 66421 Homburg/Saar<br>Tel: 06841/16-23002, Fax: 16-23101<br>E-Mail: michael.pfreundschuh@uks.eu
Studienkoordination	PD Dr. med. G. Held<br>Universitätsklinikum des Saarlandes<br>Innere Medizin I, 66421 Homburg/Saar<br>Tel: 06841/16-23084, Fax: 16-23004<br>E-Mail: gerhard.held@uks.eu
Medizinischer Konsiliardienst	PD Dr. med. G. Held, Dr. med. N. Murawski, Dr. med. C. Zwick<br>Universitätsklinikum des Saarlandes<br>Innere Medizin I, 66421 Homburg/Saar<br>Tel: 06841/16-23084, Fax: 16-23004<br>E-Mail: dshnhl@uks.eu
Zentrales Studiensekretariat	Frau V. Pöschel, Frau Dr. med. M. Pfeifer, Frau D. Ehlert<br>Universitätsklinikum des Saarlandes<br>Innere Medizin I, 66421 Homburg/Saar<br>Tel: 06841/16-23084 (-21306 oder -21303), Fax: 16-23004<br>E-Mail: dshnhl@uks.eu
Biometrie	Prof. Dr. M. Löffler, Frau Dr. M. Ziepert<br>IMISE/ NHL-Studiensekretariat<br>Universität Leipzig, Härtelstr. 16-18<br>04107 Leipzig<br>Tel: 0341/97-16120, Fax: 0341/97-16119<br>E-Mail: marita.ziepert@imise.uni-leipzig.de
Dokumentation & Monitoring	Frau Dipl.–Dok. T. Rixecker, Frau K. Monz<br>NHL-Studiensekretariat<br>Universitätsklinikum des Saarlandes<br>Innere Medizin I, 66421 Homburg/Saar<br>Tel: 06841/16-23084, Fax: 16-23004<br>E-Mail: dshnhl@uks.eu
Referenzpathologie	Prof. Dr. med. A. Rosenwald<br>Institut für Pathologie<br>Universität Würzburg, Josef-Schneider-Str. 2<br>97080 Würzburg<br>Tel.: 0931/31-81199, Fax: 31-81224<br>E-Mail: Rosenwald@mail.uni-wuerzburg.de
Referenzstrahlentherapie	Prof. Dr. med. Ch. Rübe<br>Klinik für Strahlentherapie und Radioonkologie<br>Universitätsklinikum des Saarlandes<br>66421 Homburg/Saar<br>Tel.: 06841/16-24606, Fax: 06841/16-24699<br>E-Mail: radonk@uks.eu

0.2 Studienleitkommission der DSHNHL

0.2.1 Vorstand der DSHNHL

- Prof. Dr. M. Pfreundschuh, Homburg
- Prof. Dr. N. Schmitz, Hamburg
- Prof. Dr. M. Löffler, Leipzig
- Prof. Dr. L. Trümper, Göttingen

0.2.2 Weitere Mitglieder der Studienleitkommission

- Prof. Dr. B. Glass, Hamburg
- PD. Dr. G. Held, Homburg
- Dr. M. Nickelsen, Hamburg
- V. Pöschel, Homburg
- Prof. Dr. G. Wulf, Göttingen
- Dr. S. Zeynalova, Leipzig
- Dr. M. Ziepert, Leipzig

0.2.3 Scientific Advisory Board

Das Scientific Advisory Board entscheidet über die Unterstützung wissenschaftlicher Begleitprojekte zur Studie. Ihm gehören an (Stand 02/2011):

• 1 Mitglied der Studienleitung	Prof. Dr. L. Trümper (Göttingen)
• 1 Pathologe	Prof. Dr. A. Rosenwald (Würzburg)
• 1 Biometriker	Dr. M. Ziepert (Leipzig)
• externe Experten	Prof Dr. M. Kneba (Kiel), Prof. Dr. R. Siebert (Kiel)

0.3 Protokollkomitee und Daten- und Sicherheits-Monitoringkomitee (DSMK)

0.3.1 Protokollkomitee

Dem Protokollkomitee gehören folgende Mitglieder an (Stand 02/2011):

- Prof. Dr. M. Bentz, Karlsruhe (7)
- PD Dr. P. Borchmann, Köln (7)
- PD Dr. A. Buck, München (4)
- Prof. Dr. A. Bücker, Homburg (5)
- Prof. Dr. M. Dreyling, München (7)
- Dr. M. Engelhard, Essen (3)
- Prof. Dr. N. Frickhofen, Wiesbaden (7)
- Prof. Dr. B. Glass, Hamburg (1)
- PD Dr. M. Hänel, Chemnitz (7)
- Prof. Dr. F. Hartmann, Lemgo (7)
- PD Dr. G. Held, Homburg (1)
- Prof. Dr. D. Hellwig, Homburg (4)
- Prof. Dr. U. Kaiser, Hildesheim (7)
- Prof. Dr. C.-M. Kirsch, Homburg (4)
- Prof. Dr. W. Knauf, Frankfurt/Main (6)
- Dr. P. Koch, Münster (7)
- PD Dr. E. Lengfelder, Mannheim (7)
- Dr. R. Liersch, Münster (7)
- Dr. W. Lindemann, Hagen (7)
- Prof. Dr. M. Löffler, Leipzig (1)
- Dr. B. Metzner, Oldenburg (7)
- Dr. M. Nickelsen, Hamburg (1)
- Dr. N. Peter, Cottbus (7)
- Prof. Dr. M. Pfreundschuh, Homburg (1)
- V. Pöschel, Homburg (1)
- Prof. Dr. A. Rosenwald, Würzburg (2)
- Dr. Ch. Rudolph, Berlin (7)
- Prof. Dr. Ch. Rübe, Homburg (3)
- PD Dr. H. Schmidberger, Mainz (3)
- PD Dr. R. Schmits, Saarbrücken (6)
- Prof. Dr. N. Schmitz, Hamburg (1)
- Prof. Dr. J. Schubert, Hamm (7)
- PD Dr. S. Stilgenbauer, Ulm (7)
- Prof. Dr. L. Trümper, Göttingen (1)
- PD Dr. U. Wedding, Jena (1)
- PD Dr. M. Witzens-Harig, Heidelberg (7)
- Prof. Dr. G. Wulf, Göttingen (1)
- Dr. S. Zeynalova, Leipzig (1)
- Dr. M. Ziepert, Leipzig (1)

(1) Vertreter der Studienleitkommission der DSHNHL
(2) Vertreter der Referenzpathologie
(3) Vertreter der Referenzstrahlentherapie
(4) Vetreter der Referenznuklearmedizin
(5) Vertreter der Referenzradiodiagnostik
(6) Vertreter der niedergelassenen Onkologen
(7) Gewählte Mitglieder sowie Leiter von Teilstudien innerhalb der DSHNHL

Die Adressen der Mitglieder des Protokollkomitees befinden sich in Anhang 14.6.

0.3.2 DSMK

Dem Daten- und Sicherheits- Monitoringkomitee gehören folgende unabhängige Experten an.

- Prof. Dr. G. Brittinger, Essen
- Prof. Dr. V. Diehl, Köln
- Prof. Dr. K. Havemann, Marburg
- Prof. Dr. R. Herrmann, Basel

Das DSMK erhält in regelmäßigen Abständen Informationen über den Fortgang der Studie und nimmt folgende Funktionen wahr:

- Beurteilung des Fortschrittes der Studie
- Beurteilung der Sicherheit
- Beurteilung der Schwerwiegenden Unerwünschten Ereignisse
- Beurteilung der Ergebnisse der Zwischenauswertungen

Das DSMK gibt Empfehlungen an die Studienleitung bezüglich der Fortführung, Modifizierung oder der vorzeitigen Beendigung der Studie.

Die Adressen der Mitglieder des DSMK befinden sich in Anhang 14.6.

0.3.3 GCP-Konformität

Im Januar 1997 wurde von der International Conference on Harmonization die „Note for Guidance on Good Clinical Practice" (ICH-GCP) verabschiedet. Die Studien der DSHNHL werden entsprechend den vorhandenen Kapazitäten unter Berücksichtigung der GCP-Grundsätze geplant, durchgeführt und ausgewertet. Die Deklaration von Helsinki liegt allen Studien zugrunde.

Unterschrift des Studienleiters

....

Prof. Dr. med. M. Pfreundschuh

0.4 Präambel zum Amendment

Die geplante Zwischenauswertung der UNFOLDER-Studie vom 01.07.2012 mit 443 auswertbaren Patienten, von denen 285 aufgrund „Bulky Disease“ und/oder bestrahlbarem extralymphatischen Befall in einen Therapiearm mit additiver Bestrahlung dieser Regionen oder Beobachtung randomisiert worden waren, ergab, dass ein vorbestimmtes Abbruchkriterium erfüllt war. Tatsächlich war das ereignisfreie Überleben der 139 Patienten, die in den Arm mit Bestrahlung randomisiert worden waren, hochsignifikant besser als das ereignisfreie Überleben der 146 Patienten, die in den Beobachtungsarm randomisiert worden waren. Der Unterschied der ereignisfreien Überlebenskurven der Patienten mit und ohne Bestrahlung erreichte einen p-Wert von 0.004 zugunsten des Therapiearmes mit Bestrahlung und lag damit unter der vordefinierten „alpha spending function“ von p = 0.008. Das daraufhin einberufene Daten- und Sicherheits-Monitoringkomitee (DSMK) empfahl daher auf seiner Sitzung am 31. Juli 2012, für Patienten mit „Bulky Disease“ und/oder bestrahlbarem extralymphatischem Befall die beiden Therapiearme ohne Bestrahlung (R-CHOP-21 ohne Bestrahlung; R-CHOP-14 ohne Bestrahlung) zu schließen, die beiden Therapiearme mit Bestrahlung (R-CHOP-21 mit Bestrahlung; R-CHOP-14 mit Bestrahlung) wie geplant fortzusetzen. Dieser Empfehlung schloss sich das Protokollkomitee auf seiner Sitzung am 31. August 2012 an.

Diese Entscheidung machte ein entsprechendes Amendment zum UNFOLDER-Studienprotokoll notwendig, das hiermit vorgelegt wird. Dabei beschränkt sich das Amendment auf solche Punkte, die die pratkische Durchführung der Behandlung innerhalb des UNFOLDER-Studienprotokolls betreffen. Auf eine Überarbeitung der wissenschaftlichen Grundlagen wurde verzichtet, weil seit der Abfassung des ursprünglichen UNFOLDER-Protokolls keine Erkenntnisse publiziert wurden, die für den weiteren Verlauf der UNFOLDER-Studie relevant wären.

Homburg, im November 2012

0.5 Protokoll-Synopse DSHNHL 2004-3

Studiennummer	DSHNHL 2004-3
Kurzbezeichnung der Studie	6 x Rituximab plus CHOP-21 vs. 6 x Rituximab plus CHOP-14 mit oder ohne konsolidierende Bestrahlung von großen Tumormassen und/oder Extranodalbefällen (Unfavourable young low-risk densification of R-chemo regimens = UNFOLDER 21/14)
Indikation	Aggressive $CD20^+$ B-NHL bei 18- bis 60-jährigen Patienten: alle Patienten mit IPI = 1 (nach altersadaptiertem IPI) und solche mit IPI = 0 nur bei gleichzeitigem Vorliegen großer Tumormassen (≥ 7.5cm)
Primäres Ziel der Studie	Verbesserung der Therapieergebnisse bzw. Reduktion der Nebenwirkungen einer kombinierten Immuno-Chemo-Therapie mit 6 Zyklen des monoklonalen anti-CD20-Antikörper Rituximab durch Verringerung der Therapieintervalle von 3 auf 2 Wochen bzw. Verbesserung durch konsolidierende Strahlentherapie (39,6 Gy) großer Tumormassen („Bulk“)
Sekundäre Ziele der Studie	Relevanz der konsolidierenden Bestrahlung von qualifizierten Extranodalbefällen; Vergleich der kurzfristigen und langfristigen Nebenwirkungen sowie der Kosten
Studiendesign	2 x 2-armige (in Analogie zum faktoriellen Design), offene, multizentrische, prospektive, randomisierte Phase-III-Studie (Therapieoptimierungsversuch)
Studienpopulation	Patienten mit unbehandeltem aggressiven $CD20^+$ Non-Hodgkin-Lymphom im Alter von 18 bis 60 Jahren ohne wesentliche Begleiterkrankungen mit IPI = 1 (alle) oder IPI = 0 mit großen Tumormassen ≥ 7,5 cm
Patientenzahl	578 Patienten mit Bulk, 1072 Patienten insgesamt
Therapie	Patienten erhalten randomisiert 6 Zyklen einer kombinierten Immuno-Chemotherapie mit dem monoklonalen anti-CD20-Antikörper Rituximab zusammen mit 6 Zyklen einer Chemotherapie mit Cyclophosphamid, Doxorubicin, Vincristin und Prednison in 21-tägigen Abständen (R-CHOP-21) oder 14-tägigen Abständen (R-CHOP-14). Patienten mit initialem Bulk und/oder qualifizierten Extranodalbefällen erhalten danach eine zusätzliche konsolidierende Strahlentherapie mit 39,6 Gy.
Primärer Endpunkt	Das Hauptzielkriterium ist die Zeit bis zum Therapieversagen (time to treatment failure = TTF) gerechnet ab der Randomisation
Sekundäre Endpunkte	Sekundäre Zielkriterien sind die CR-Rate, das Überleben, die Tumorkontrolle, das krankheitsfreie Überleben sowie Toxizität, Parameter zur Gesundheits-ökonomie und zur Protokolladhärenz sowie Analyse der Rezidive.
Auswertung	Die Zeit bis zum Therapieversagen wird mit dem Log-rank-Test zwischen den beiden Therapiestrategien verglichen.
Zeitplan	Beginn der Studie 10/2005, Ende der Rekrutierung ca. 9/2014; ausgehend von einer Rekrutierungszahl von 120 Patienten pro Jahr sollen 1072 Patienten randomisiert werden bei einer Rekrutierungsdauer von 9 Jahren. Die Nachbeobachtungsdauer im Rahmen der Studie beträgt 3 Jahre für den einzelnen Patienten, beginnend mit dem Abschluss der Therapie. Anschließend ist außerhalb der klinischen Prüfung eine lebenslange Kontrolle vorgesehen.
Förderer	Deutsche Krebshilfe / Dr. Mildred-Scheel-Stiftung

0.6 Flussdiagramm der Studie 2004-3

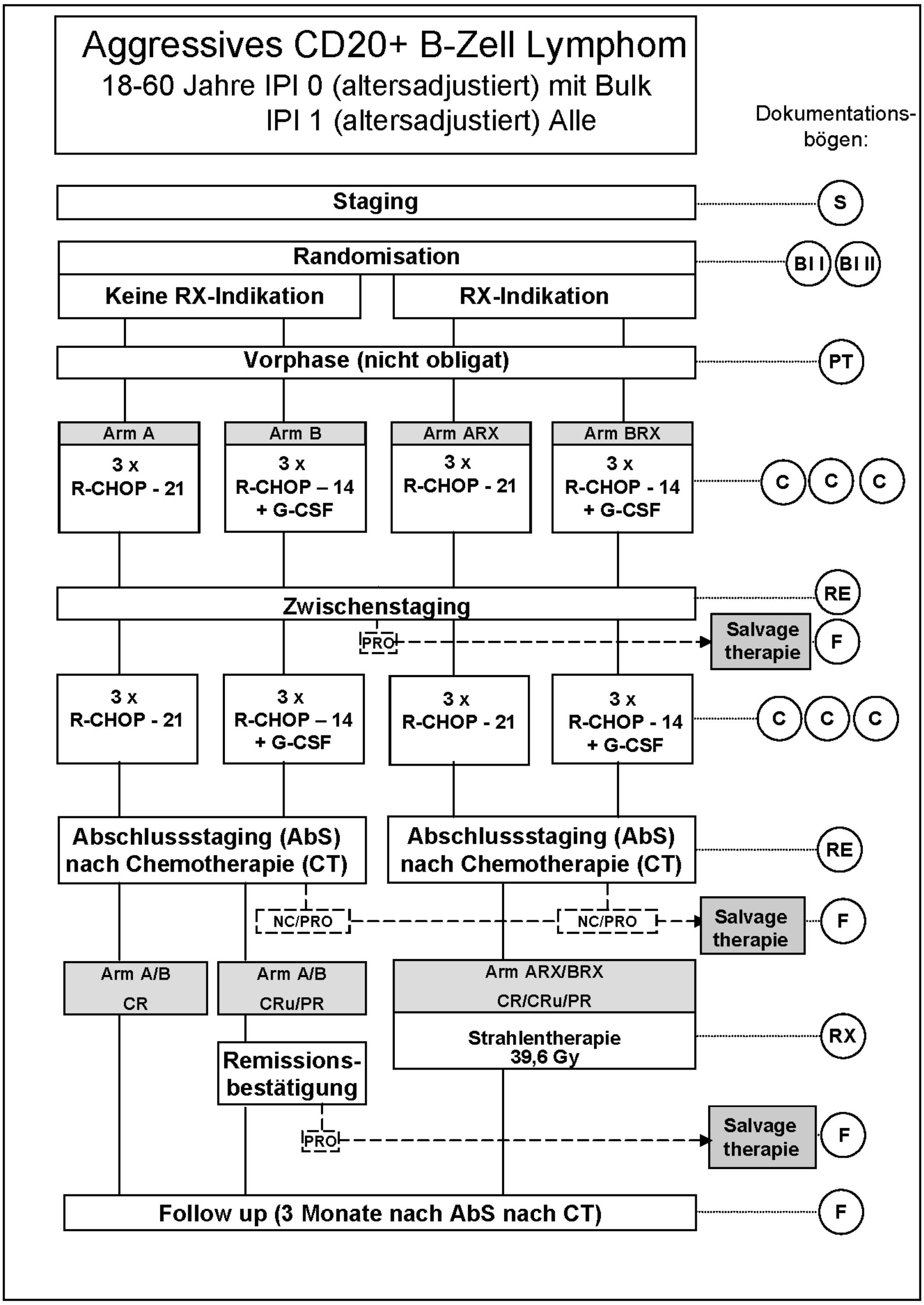

Meldung eines schwerwiegenden unerwünschten Ereignisses .... SAE

Abschlussbogen im Falle des Todes .... D

0.7 Therapielan und durchzuführende Untersuchungen

Phase	Staging	Vorphase	Therapie			Zwischen-staging	Therapie			Ab-schluss-staging nach Chemo-therapie	Remissions-bestätigung (nur Arme ohne RX-Indikation) in CRu/PR	Strahlen-therapie in ARX/BRX	Nachsorge
			Zyk-lus 1	Zyk-lus 2	Zyk-lus 3		Zyk-lus 4	Zyk-lus 5	Zyk-lus 6				
Tag		-6 bis 0	1	22/15	43/29	43/29 + ca. 14	64/43	85/57	106/71	106/71 + ca. 14	4 Wochen nach Abschluss-staging	2 bis 6 Wochen nach Beginn Zyklus 6	– 3 Mon. nach Abschlussstaging: 1. FU<br>– in Jahr 1 und 2: alle 3 Mon.<br>– in Jahr 3-5: alle 6 Mon.<br>– danach jährlich
Vorphasetherapie		X											
Arm A, ARX<br>Arm B, BRX			X<br>X	X<br>X	X<br>X		X<br>X	X<br>X	X<br>X				
Strahlentherapie (Arm ARX, BRX)												X[1]	
Anamnese	X		X	X	X	X	X	X	X	X	X	X	X
klinische Untersuchung	X		X	X	X	X	X	X	X	X	X	X	X
Allgemeinzustand (ECOG)	X[2a]	X[2b]	X[2c]	X[2c]	X[2c]	X	X[2c]	X[2c]	X[2c]	X	X	X	X
Labor	X[3a]		X[3b]	X[3b]	X[3b]	X[3a]	X[3b]	X[3b]	X[3b]	X[3a]	X[3a]		X[3c]
Blutbild während der Chemo			X[4]	X[4]	X[4]		X[4]	X[4]	X[4]				
Thorax-RX	X[8]												X[6]
Abdominal-Sono	X												X[6]
CT-Hals / Thorax / Abdomen	X[8]					X[5]				X	X[9]		X
Knochenmarkbiopsie	X									X[5]	X[5]		X[6]
Lumbalpunktion	X[7]												
CTC-Beurteilung			X	X	X		X	X	X			X	
Unerwünschte Ereignisse			X	X	X		X	X	X			X	X
Folgeerkrankungen													X

1	falls initial Bulk/E-Befall bzw. bei Hodenbefall (RX kontralateraler Hoden)
2	a) bei Patientenaufnahme<br>b) am Ende der Vorphase<br>c) minimaler Wert während der Chemotherapie
3	a) Leukoz., Lymphoz., Mono., Bas., Neut., Eos., Thromboz., Hb, BSG, LDH, GPT, AP, □GT, Bilirubin, Gesamteiweiß, Albumin, Paraprotein, IgG, IgA, IgM, □2-Mikrogl., Kreat., EF, Lungendiffusionskapazität<br>b) Leukoz., Thromboz., Hb, LDH, GPT, AP, Bilirubin, Kreatinin, Elektrolyte unmittelbar vor Beginn des Zyklus<br>c) Leukoz., Lymphoz., Mono., Bas., Neut., Eos., Thromboz., Hb, LDH, EF, Lungendiffusionskapazität

4	Leukoz., Thromboz., Hb vor Beginn des Zyklus und mindestens 2 Messungen im Nadirbereich
5	falls initial befallen
6	falls initial befallen, nach Therapieende überprüfen bis ohne Befund
7	Liquorpunktion mit Zytologie bei Befall der Testes
8	Versendung der kompletten bildgebenden Diagnostik an das Studiensekretariat in Homburg, möglichst in digitaler Form (CD)
9	nur in Arm A und B ohne RX-Indikation: Kontrolle der Restbefunde, falls im Abschlusstaging nach Chemotherapie Therapieerfolg CRu/PR

G-CSF: G-CSF soll bei Auftreten verlängerter Leukozytopenien (> 3d mit < 1 x 10^9/L) in den Armen A, ARX in den darauf folgenden Zyklen ab Tag 4 gegeben werden; in den Armen B, BRX ist die G-CSF-Gabe obligat!

0.7 Therapieplan und durchzuführende Untersuchungen
DSHNHL 2004-3 Version 4.0 vom 22.11.2012 13

0.8 Checkliste für den Arzt

0.8.1 Was ist vor Therapiebeginn zu tun ?

1) Voraussetzung: Teilnahmeerklärung der Klinik sowie Ethikvotum der zuständigen Ethikkommission liegen im Studiensekretariat vor?
2) Diagnose des Primärpathologen mit CD20-Immunhistologie liegt vor?
3) Risikofaktoren nach IPI bestimmt?
4) BULK Befall: vorhanden / nicht vorhanden?
5) Extranodalbefall: vorhanden / nicht vorhanden?
6) Prätherapeutische bildgebende Diagnostik (CT, MRT – möglichst auf CD/elektronisch) an das Studiensekretariat Homburg geschickt zur Erstellung einer Referenzbeurteilung **(alle Patienten, unabhänging von Bulk-und/oder E-Befall!)**?
7) Studienzuordnung geklärt?
8) Einschlusskriterien alle erfüllt?
9) Ausschlusskriterien alle ausgeschlossen?
10) Patient aufgeklärt, Zustimmung zur Randomisation erhalten und Einverständniserklärung an das Studiensekretariat geschickt?
11) Alle Staginguntersuchungen durchgeführt?
12) Stagingbogen, Meldebogen I, Meldebogen II und Histologiebefund an das Studiensekretariat geschickt? **Unterschrift Strahlentherapeut auf Stagingbogen und Meldebogen I eingeholt (für alle Patienten obligat)?**
13) Randomisation durch das Studiensekretariat durchgeführt?
14) Bei konsolidierender Bestrahlung von BULK- und/oder qualifizierten Extranodal-Befällen bzw. bei Hodenbefall (RX kontralateraler Hoden): Procedere zwischen Internist und Strahlentherapeut besprochen? Strahlentherapievorschlag bei der Referenzstrahlentherapie beantragt?
15) Serum-, Blut- und Knochenmarkproben eingesandt?

0.8.2 Durchführung der Therapie

1) Immuno-Chemotherapie Zyklus 1-3
2) Zwischenstaging
3) Immuno-Chemotherapie Zyklus 4-6
4) Abschlussstaging nach Chemotherapie: Restaging 14 Tage nach Beginn der letzten Rituximab-Gabe
5) In den Armen ohne Bestralungsindikation: Remissionsbestätigung 4 Wochen nach Abschlussstaging nach Chemotherapie, falls dort Therapieerfolg CRu/PR
6) 2 bis 6 Wochen nach Beginn Zyklus 6 Beginn konsolidierende Strahlentherapie bei Bulky Disease und/oder qualifizierten Extranodalbefällen bzw. bei Hodenbefall (Arm ARX, BRX).

0.8.3 Was ist bzgl. der konsolidierenden Strahlentherapie zu beachten?

Bei konsolidierender Bestrahlung von Bulky Disease und/oder qualifizierten Extranodal-Befällen bzw. bei Befall Testes (RX kontralateraler Hoden) (Arme ARX, BRX) (s. 5.7.3)

1) prätherapeutische Vorstellung mit Stagingbogen und Bildgebung beim lokalen Strahlentherapeuten

2) Feststellung des Bestrahlungskonzepts
3) Nach Abschluss der Strahlentherapie Ausfüllen des Radiotherapiebogens und Versand der Simulations- und Verifikationsaufnahmen sowie des Bestrahlungsplans durch den Radiotherapeuten an die Studienzentrale (Unterlagen werden nach Auswertung zurückgeschickt!)

0.8.4 Was ist nach Therapieende zu tun?

1) Restaging 14 Tage nach Beginn der letzten Rituximabapplikation
2) In den Armen ohne Bestrahlungsindikation: Remissionsbestätigung 4 Wochen nach Abschlussstaging nach Chemotherapie, falls dort Therapieerfolg CRu/PR
3) falls RX durchgeführt (Arm ARX/BRX): 1. Nachsorgeuntersuchung 3 Monate nach Abschlussstaging nach Chemotherapie!
4) Regelmäßige Nachsorgeuntersuchungen: 1. Follow up durch einen Internisten in allen Armen:
   3 Monate nach Abschlussstaging nach Chemotherapie , danach die ersten 2 Jahre alle 3 Monate, in Jahr 3-5 alle 6 Monate, danach jährlich)

0.8.5 Was ist bei Progress, NC oder Rezidiv zu tun?

Bitte in jedem Fall mit der Studienzentrale in Kontakt treten und prüfen, ob der Patient für ein geeignetes Therapieprotokoll qualifiziert ist.

0.8.6 Was ist bei vorzeitigem Therapieende zu tun?

Ursache des vorzeitigen Therapieabbruchs der Studienleitung mitteilen. Zum Zeitpunkt des Abbruches, wenn möglich, ein Abschlussstaging durchführen und Therapieergebnis zum Zeitpunkt des Abbruches auf dem Restagingbogen dokumentieren. Weitere durchgeführte Follow-up Untersuchungen dokumentieren.

0.8.7 Was ist bei einem Schwerwiegenden Unerwünschten Ereignis (SUE) zu tun?

Innerhalb eines Werktages Benachrichtigung der Studienleitung per SUE-Meldefax. Nach Therapieende innerhalb von 10 Tagen.

0.8.8 Was ist bei einem Todesfall zu tun?

Genaue Dokumentation des Zeitpunktes des Todes sowie der vermutlichen Todesursache auf dem Abschlussbogen und Einsendung des Sektionsbefundes (falls vorhanden) an das Studiensekretariat.

0.8.9 Was ist bei einem Arztwechsel/Klinikwechsel zu tun?

Mitteilung an das Studiensekretariat, wer die Behandlung, Nachsorge bzw. Dokumentation fortführt und bei Klinikwechsel Mitteilung, wo Behandlung fortgeführt wird.

0.9 Änderungen im Vergleich zu dem Protokoll der zweiten Studiengeneration (High-CHOEP-Studie)

In dieser dritten Studiengeneration der DSHNHL Studien haben sich einige Änderungen gegenüber der vorhergehenden Studiengeneration (DSHNHL-1999-2 „High-CHOEP") ergeben. Die Wichtigsten sind:

- Anstelle von 6 Zyklen Chemotherapie werden 6 Zyklen Immuno-Chemotherapie gegeben
- Etoposid wird nicht mehr gegeben (s. 2.3)
- Randomisation in Immuno-Chemotherapie alle 3 vs. alle 2 Wochen
- Strahlentherapiedosis beträgt 39.6 Gy (bisher 36 Gy)
- Zentraler Review der Primärdiagnostik **aller in die Studie eingebrachten Patienten** mit Überprüfung der Stadieneinteilung und ggf. des Vorliegens bzw. Ausschlusses eines Bulky Disease/Extranodalbefalls nach einheitlichen Kriterien durch die Studienzentrale in Homburg. Diese dient der Qualitätskontrolle der Entscheidung vor Ort und der Erstellung eines Bestrahlungsplanes bei entsprechend randomisierten Patienten. Daher ist die Unterschrift des Strahlentherapeuten auf dem Stagingbogen obligat.
- Patienten mit ZNS- Befall (intrazerebral, meningeal und intraspinal) werden nicht aufgenommen
- das erste Follow-up erfolgt durch einen Internisten **in allen Armen: 3 Monate nach Abschlussstaging nach Chemotherapie**
- ein Zusatzstaging nach konsolidierender Strahlentherapie durch den Strahlentherapeuten ist nicht obligat
- der Allgemeinzustand des Patienten wird in ECOG angegeben

Im Übrigen wird die GCP-konforme Durchführung der Studie beibehalten.

0.10 Änderungen im Amendment vom 22.11.12

- Die Remissionsbestätigung (4 Wochen nach Abschlussstaging nach Chemotherapie - falls dort Therapieerfolg CRu/PR) wird lediglich in den Armen ohne RX-Indikation durchgeführt. Eine Remissionsbestätigung in den Armen mit RX-Indikation entfällt
- konsolidierende Strahlentherapie Bulky Disease und/oder qualifizierte Extranodalbefälle wird auf jeden Fall durchgeführt und nicht mehr randomisiert; es wird nur noch in die zwei Therapiearme 6xR-CHOP-21 +RX und 6xR-CHOP-14 +RX randomisiert
- bei Befall eines Hodens wird der kontralaterale Hoden prophylaktisch bestrahlt

1 Ziele der Studie

1.1 Primäres Ziel der Studie

In der vorliegenden Studie sollen bei Patienten mit unbehandeltem aggressiven $CD20^+$ NHL mit günstigem Risikoprofil (d.h. mit keinem oder einem Risikofaktor entsprechend altersadaptiertem IPI[1]), die entweder 1 Risikofaktor und/oder Bulky Disease haben, in einer randomisierten multizentrischen Studie folgende Fragen geprüft werden:

1) Kann die Wirksamkeit einer Immuno-Chemotherapie mit 6 Zyklen Rituximab in Kombination mit CHOP (Cyclophosphamid, Doxorubicin, Vincristin und Prednison) verbessert werden, wenn die Therapieintervalle von 3 auf 2 Wochen reduziert werden (6 x R-CHOP-21 vs. 6 x R-CHOP-14)?
2) Führt die zusätzliche konsolidierende Bestrahlung von Bulky Disease im Anschluss an die Immuno-Chemotherapie zu einer Verbesserung der Therapieergebnisse?

Eingeschlossen in die Studie werden alle Patienten mit einem CD20-positiven aggressiven NHL im Alter von 18 bis 60 Jahren der Gruppe mit günstigem Risikoprofil (also IPI[1] = 0 (altersadjustiert) mit Bulk oder IPI = 1 mit oder ohne Bulk (mindestens ein Einzel- oder Konglomerat-Tumordurchmesser ≥7.5cm)). Hauptzielkriterium ist die Zeit bis zum Therapieversagen (time to treatment failure = TTF). Es soll mit einer Power von 80% ein Unterschied in der 3-Jahres – TTF-Rate von 10% (konstante Hazard Ratio von 0.615) mit einer Irrtumswahrscheinlichkeit von 5% (zweiseitig) aufgedeckt werden.

1.2 Sekundäre Ziele der Studie

Sekundäres Ziel der Studie ist es, die Relevanz der konsolidierenden Bestrahlung der qualifizierten Extranodal-Befälle zu untersuchen.
Weitere sekundäre Ziele der Studie sind die Erhebung weiterer Größen zur Bewertung.

1) der Nebenwirkungen:
   - Rate an Neutrozytopenien
   - Rate an Thrombozytopenien
   - Rate an Anämien
   - Rate an Infektionen
   - Rate an Antibiotikagaben
   - Rate an Erythrozytensubstitutionen
   - Rate an Thrombozytensubstitutionen
   - Rate an Sekundärneoplasien

2) der Wirksamkeit (s. 7.2.2.1):
   - Rate an kompletten Remissionen
   - Rate an Progressen unter Therapie
   - rezidivfreies Überleben
   - krankheitsfreies Überleben

---

[1] Internationaler Prognostischer Index

- Überleben
- Tumorkontrolle
- Rate an lokaler Kontrolle bei Bulky Disease und Extranodalbefällen mit oder ohne konsolidierende Bestrahlung

3) der Rezidivmuster :
   - Rezidive in bestrahlten Lokalisationen
   - Rezidive in primär befallenen Lokalisationen
   - Rezidive in primär nicht befallenen Lokalisationen

4) der Sicherheit (s. 7.2.2.2)

5) von gesundheitsökonomischen Aspekten (s.7.2.2.3)

6) der Protokolladhärenz (s. 7.2.2.4)

2 Begründung und Rationale der Studie

2.1 Stand der Forschung

Die aggressiven Non-Hodgkin-Lymphome nach der klinischen Gruppierung der R.E.A.L.-Klassifikation[2;3] sind aggressive bösartige Erkrankungen des lymphatischen Systems, die unbehandelt rasch zum Tode führen. Eine Strahlentherapie ist nur bei lokal begrenztem Lymphombefall kurativ, wobei die Heilungsraten bei Patienten > 60 Jahren deutlich abfallen[4]. Erst nach der Einführung von Polychemotherapieschemata wie dem CHOP-Schema, das als wirksamstes Zytostatikum neben Cyclophosphamid, Vincristin und Prednison das Anthrazyklin Doxorubicin enthält, gelang es, auch bei Patienten in fortgeschrittenen Stadien, komplette Remissionen und Heilungen zu erzielen[5]. Allerdings konnten nach der Einführung des CHOP-Protokolls vor fast 30 Jahren[5] die Ergebnisse der Chemotherapie bei hochmalignen Non-Hodgkin-Lymphomen lange Zeit nicht mehr weiter verbessert werden, trotz der Dosissteigerung einzelner und der Hinzunahme neuer Zytostatika in die Therapieschemata der zweiten und dritten Generation[6-11]. Dies wurde durch die Ergebnisse der Intergroup-Studie der SWOG und ECOG belegt, die erstmals in einer großen randomisierten Studie (über 1000 Patienten) CHOP mit m-BACOD, ProMACE-CytaBOM und MACOP-B verglich. Dabei zeigten die unterschiedlichen Schemata der sog. ersten, zweiten und dritten Generation eine vergleichbare Wirksamkeit: das krankheitsfreie Überleben nach 4 Jahren war nicht signifikant unterschiedlich und betrug ungefähr 40%[12]. Ebenso wenig ergaben sich Unterschiede im Gesamtüberleben. Zu gleichen Ergebnissen kommt man, wenn man die Ergebnisse der Patienten mit unterschiedlichem Risikoprofil entsprechend des "International Prognostic Index" (IPI)[13] betrachtet. Allerdings wurden Unterschiede in der Toxizität der einzelnen Schemata beobachtet: die therapieabhängigen Todesfälle betrugen nach CHOP 1%, nach m-BACOD 5%, nach ProMACE-CytaBOM 4% und nach MACOP-B 6%[12].

International anerkannte Prognosekriterien sind die 5 im "International Prognostic Index" berücksichtigten prätherapeutischen Risikofaktoren, die sich in einer Metaanalyse, in die 16 internationale Arbeitsgruppen ihre Ergebnisse von 3373 Patienten eingebracht hatten, als prognostisch relevant erwiesen hatten: Alter > 60 Jahre, Stadium III + IV, > 1 extranodaler Befall, schlechter Allgemeinzustand (ECOG 2,3,4) und erhöhter LDH-Wert[14]. Je nach der Zahl der vorhandenen Risikofaktoren (bis 1; 2; 3; > 3) können demnach 4 Risikogruppen unterschieden werden; in der besten Gruppe betragen die CR-Rate 87%, die Rezidivfreiheit nach 2 Jahren 79% und das 5-Jahres-Überleben 73%, bei der schlechtesten Gruppe sind die entsprechenden Zahlen 44%, 58% und 26%. Dieser Index ist mittlerweile auch an unabhängigen Patientenkollektiven, z.B. den Patienten der "National High Priority Study"[12] überprüft worden, wo er eine ähnlich gute diskriminierende Funktion im Hinblick auf die Unterteilung von Patientenkollektiven mit unterschiedlicher Prognose zeigte. Die wissenschaftliche Bedeutung des IPI liegt vor allem darin, dass er einen Vergleich der Ergebnisse von Studien von Patienten mit aggressiven Lymphomen erlaubt, wenn die Daten von Subgruppenanalysen entsprechend der Risikofaktoren verfügbar sind.

Erst durch die Hinzunahme von Etoposid in moderater Dosierung (CHOEP-Schema) gelang es, die Ergebnisse bei jungen Patienten mit günstigem Risikoprofil, zu verbessern. Dies konnte in der durch die Deutsche Krebshilfe / Mildred-Scheel-Stiftung geförderten NHL-B1-Studie gezeigt werden (Abb. 1)[15].

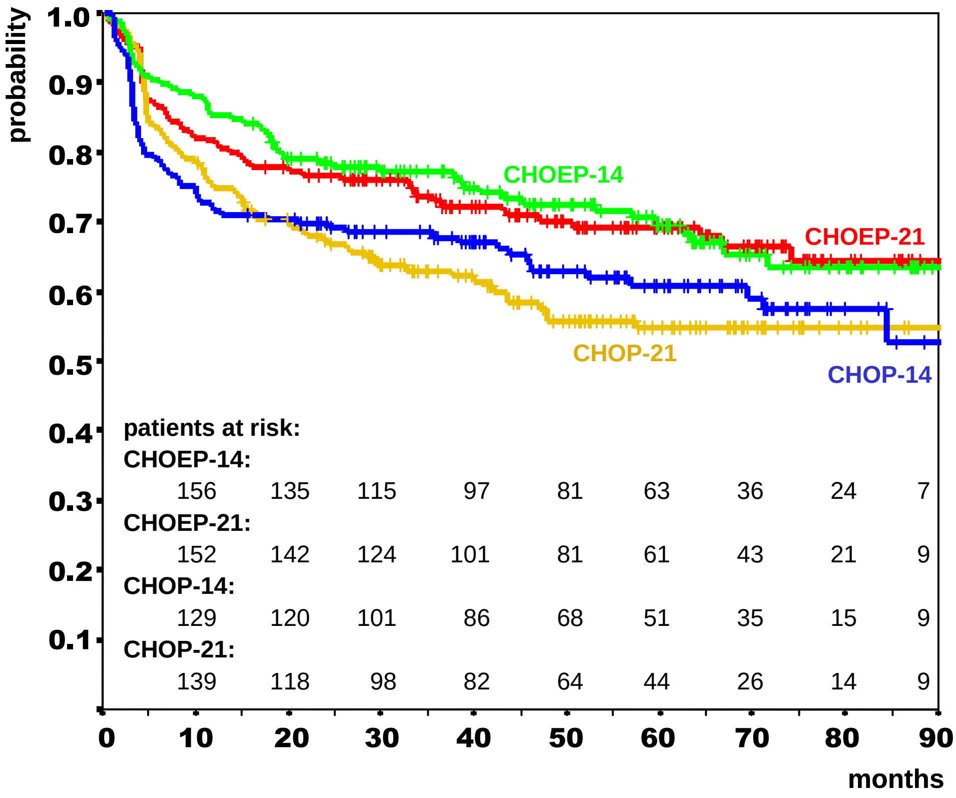

Figure 1A

Abbildung 1: Time-to-treatment-failure (TTF) bei jungen Patienten mit günstigem Risikoprofil (normaler LDH) aggressiven Lymphomen innerhalb der NHL-B1-Studie[16]. CHOEP-21 verbesserte im Vergleich zu CHOP-21 signifikant TTF, CHOEP-14 gegenüber CHOP-21 signifikant die Rate kompletter Remissionen, TTF und Gesamtüberleben.

Trotz dieses Fortschritts erschienen die Ergebnisse für die Gruppe der jungen Patienten mit günstigem Risikoprofil mit aggressiven Lymphomen immer noch verbesserungswürdig. Unter dem Eindruck des Konzeptes der „Effective Dose“[14], das eine weitere Verbesserung der TTF-Rate (time to treatment failure) nach 3 Jahren um 8% vorhersagte, wurde in der 2. Studiengeneration der DSHNHL in der DSHNHL 1999-2 („High-CHOEP“) Studie geprüft, ob eine signifikante Steigerung der Dosen von Cyclophosphamid, Doxorubicin und Etoposid tatsächlich zu einer Verbesserung der Therapieergebnisse bei jungen Patienten mit günstigem Risikoprofil führt. Die erste geplante Zwischenauswertung der DSHNHL 1999-2 Studie mit mehr als 300 Patienten und einer mittleren Beobachtungsdauer von 16 Monaten ergab jedoch, dass beide Therapiearme zu einer deckungsgleichen TTF-Rate (Zeit bis zum Therapieversagen) und Überlebensrate führen (Abb. 2). Statistische Berechnungen ergaben, dass die Wahrscheinlichkeit, dass bei einer Fortführung der Studie doch noch Unterschiede zwischen dem Standard-CHOEP- und dem High-CHOEP-Arm aufgedeckt werden könnten, 2.1% war.

Da bei gleicher Wirksamkeit die Nebenwirkungen von High-CHOEP ungleich höher als die des Standardarmes CHOEP waren, wurde auf dem Studientreffen der DSHNHL im Juni 2004 in Hamburg einstimmig beschlossen, den High-CHOEP-Arm der DSHNHL-Studie 1999-2 zu schließen und die Studie vorzeitig zu beenden.

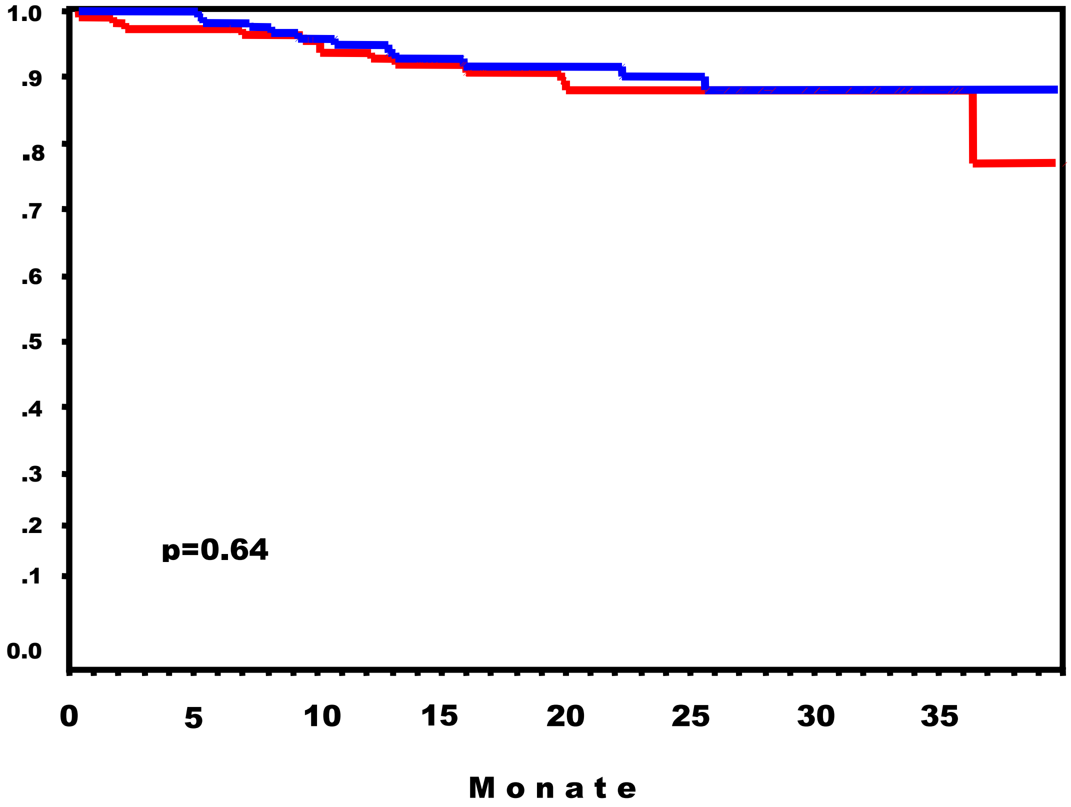

Abbildung 2: Gesamtüberleben in der DSHNHL 1999-2 Studie. Es fand sich kein Unterschied nach CHO[illegible] Standard-Dosis und eskalierter Dosis (High-CHOEP) in Bezug auf Gesamtüberleben (hier dargestellt). Ebenso waren die beiden Kurven für TTF deckungsgleich.

Parallel zur DSHNHL 1999-2 Studie beteiligten sich einige Zentren der DSHNHL an der internationalen „MInT"-Studie. Die Einschlusskriterien für diese Studie der Mabthera International Group, die 6 Zyklen einer CHOP-ähnlichen Chemotherapie mit 6 Zyklen der jeweils gleichen Chemotherapie zusammen mit 6 Gaben des monoklonalen anti-CD20-Antikörpers Rituximab verglich, entsprachen weitgehend denen der DSHNHL 1999-2 Studie, d.h. es wurden jungen Patienten mit günstigem Risikoprofil nach dem IPI rekrutiert. Im Gegensatz zu DSHNHL 1999-2 wurden in der MInT-Studie jedoch junge Patienten mit günstigem Risikoprofil im Stadium I ohne große Tumormassen (Bulky Disease) ausgeschlossen. Ebenso wie in der High-CHOEP-Studie erhielten Patienten mit Bulky Disease und/oder extranodalem Befall in der MInT-Studie eine Strahlentherapie auf die entsprechenden Regionen. Die erste geplante Zwischenauswertung von 326 Patienten der MInT-Studie, die alle Einschlusskriterien erfüllten und für die die Ergebnissse der ersten Nachsorgeuntersuchung 3 Monate nach Abschluss des definitiven Restaging vorlagen, ergab einen hochsignifikanten Vorteil der kombinierten Chemo-Immuntherapie gegenüber einer alleinigen Chemotherapie: das Signifikanzniveau zugunsten des kombinierten Ansatzes betrug $p = 0.0000308$ und lag damit mehr als 2 log-Stufen unter dem kritischen α-Wert von 0.000105, der bei Anwendung einer Alpha-Spending-Funktion für einen vorzeitigen Studienabbruch vorgesehen war (Abb. 3)[15]. Daher beschloss das Internationale Data Safety Monitoring Board, dem Prof. G. Brittinger, B. Coiffier und P. Carde angehörten, die Studie im Dezember 2003 vorzeitig zu schließen, als sich noch 45 Patienten unter Therapie befanden. Eine Follow-up-Analyse der Patienten, die in die erste Zwischenauswertung eingegangen waren, im Mai 2004 zeigte ebenso wie eine zum gleichen Zeitpunkt durchgeführte Analyse der entsprechenden Intention-to-Treat-Population, dass die Ergebnisse der ersten Interimanalyse nach einer mittleren Beobachtungszeit von mittlerweile 2 Jahren bestätigt werden[16]. Bei einer mittleren Beobachtungszeit von 24 Monaten beträgt die Freiheit von Therapieversagen bei den Patienten, die nur mit 6 Zyklen Chemotherapie behandelt

wurden 58%, bei den Patienten, die eine Kombination aus Chemotherapie und Rituximab erhalten hatten, beträgt sie 81%. Das heißt, dass fast einem Viertel der Patienten eine Rezidivtherapie erspart werden kann, durch die bekannter Maßen ca. 50% der Patienten doch noch in eine langfristig anhaltende Remission gebracht werden können.

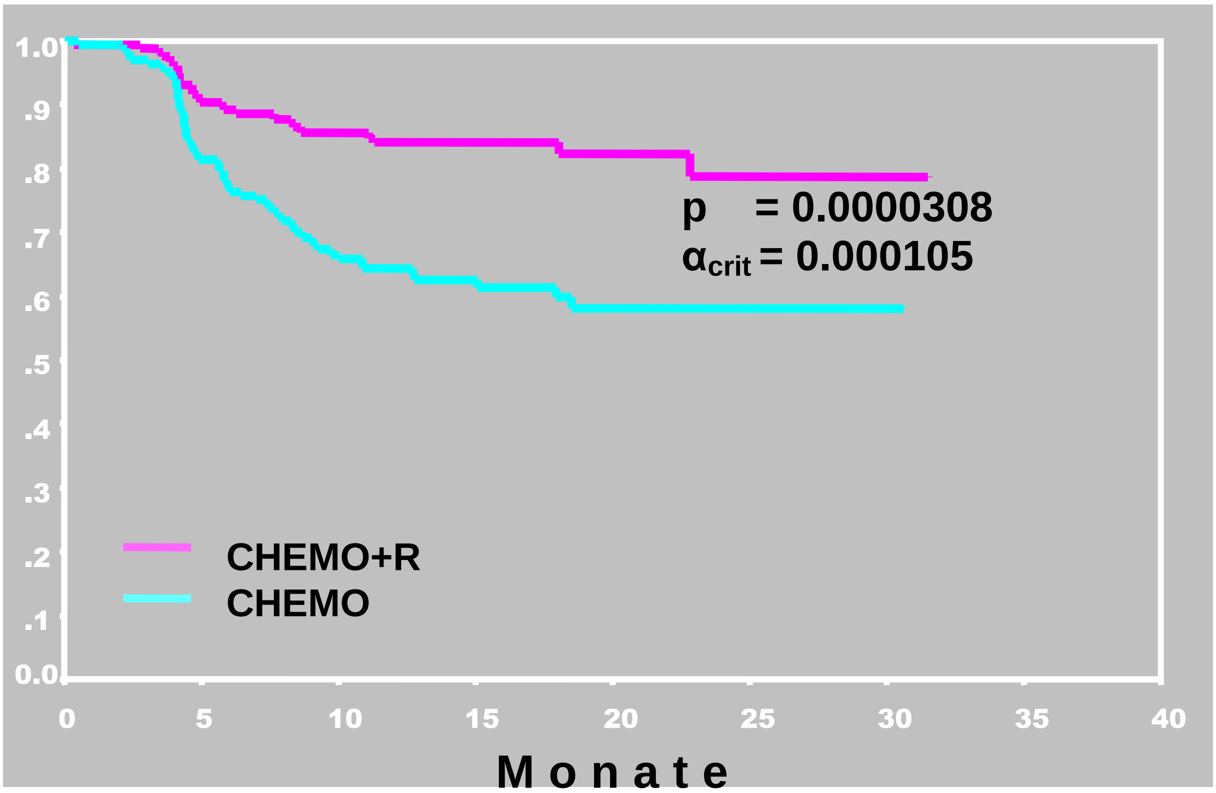

Abbildung 3: Time to treatment failure nach 6 Zyklen CHOP-ähnlicher Chemotherapie (CHEMO) verglichen mit 6 Zyklen CHOP-ähnlicher Chemotherapie in Kombination mit 6 Applikationen des monoklonalen CD20-Antikörpers Rituximab (R-CHEMO). Ergebnisse der ersten Interimanalyse von 326 Patienten (mittlere Beobachtungszeit: 16 Monate)

Obwohl durch eine solche Salvage-Therapie die Unterschiede zwischen beiden Armen verringert werden, ergibt sich nach 2 Jahren ein signifikanter Vorteil für Patienten, die mit der Kombination aus Chemotherapie und Rituximab behandelt worden waren, auch im Hinblick auf das Gesamtüberleben: dies wurde von 85% auf 95% nach 2 Jahren erhöht (Abb.4). Dies gilt auch für die ca. 50% der in der Interim-Analyse ausgewerteten Patienten, die CHOEP erhalten hatten.

Da die Hinzunahme von Rituximab zu einer CHOP-ähnlichen Chemotherapie (R-CHEMO) im Gegensatz zur Dosiseskalation („High-CHOEP“) zu einer wesentlichen Verbesserung aller Endpunkte geführt hat, müssen 6 Zyklen einer CHOP-ähnlichen Chemotherapie in Kombination mit Rituximab bis auf Weiteres als optimale Therapie und als Referenzarm für Studien bei jungen Patienten mit günstigem Risikoprofil mit $CD20^+$ aggressiven Lymphomen gelten.

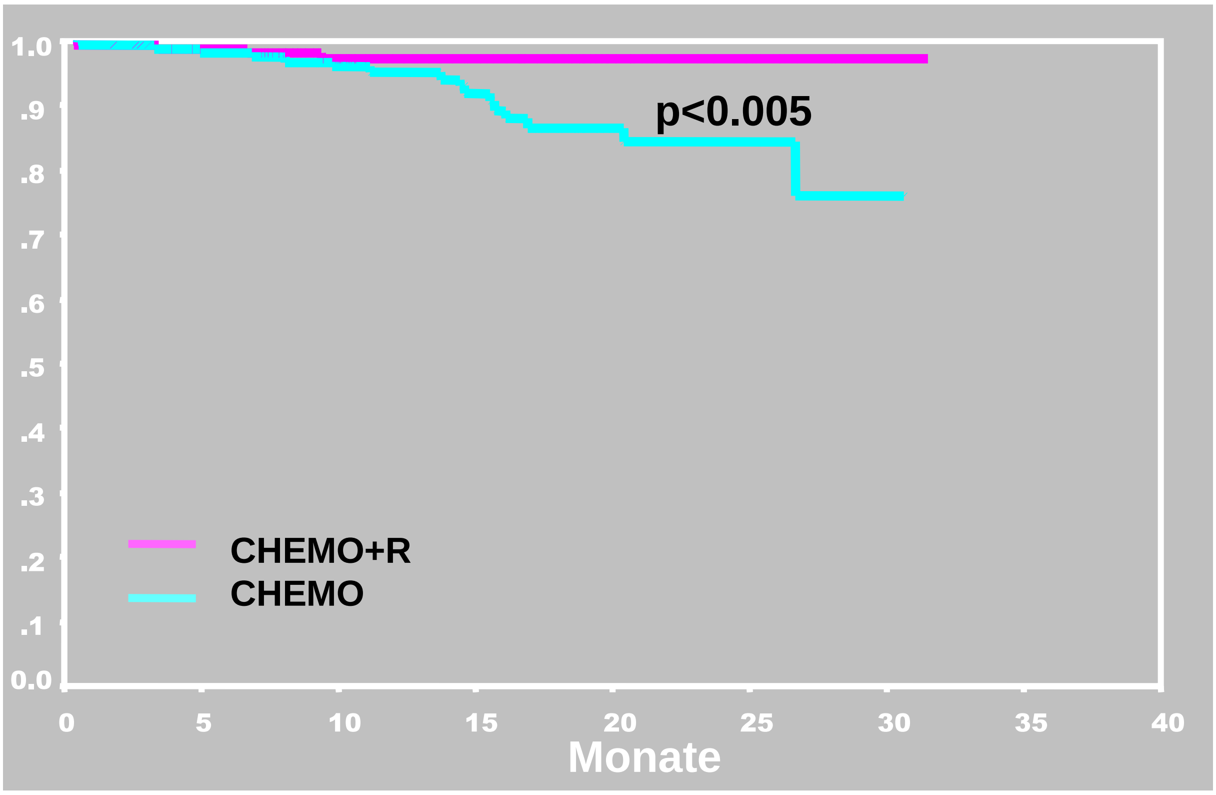

Abbildung 4: Gesamtüberleben nach 6 Zyklen CHOP-ähnlicher Chemotherapie verglichen mit 6 Zyklen CHOP-ähnlicher Chemotherapie und 6 Zyklen des monoklonalen CD20-Antikörpers Rituximab. Ergebnisse der ersten Interimanalyse von 326 Patienten (mittlere Beobachtungszeit: 16 Monate)

Eine Cox-Regression, die für die Stratifikationsvariablen Therapiearm, Risikofaktor nach IPI und dem Vorhandensein von Bulky Disease adjustierte, ergab, dass Art des Therapiearms und Bulk, weniger ausgeprägt auch Risikofaktor nach IPI, mit einem für das TTF relevanten Ereignis assoziiert waren. Tatsächlich ergeben sich deutlich Unterschiede sowohl im Hinblick auf TTF als auch auf das Gesamtüberleben, wenn man Patienten der MInT-Studie, die weder einen Risikofaktor noch Bulky Disease haben, den übrigen Patienten gegenüber stellt (Abb. 5).

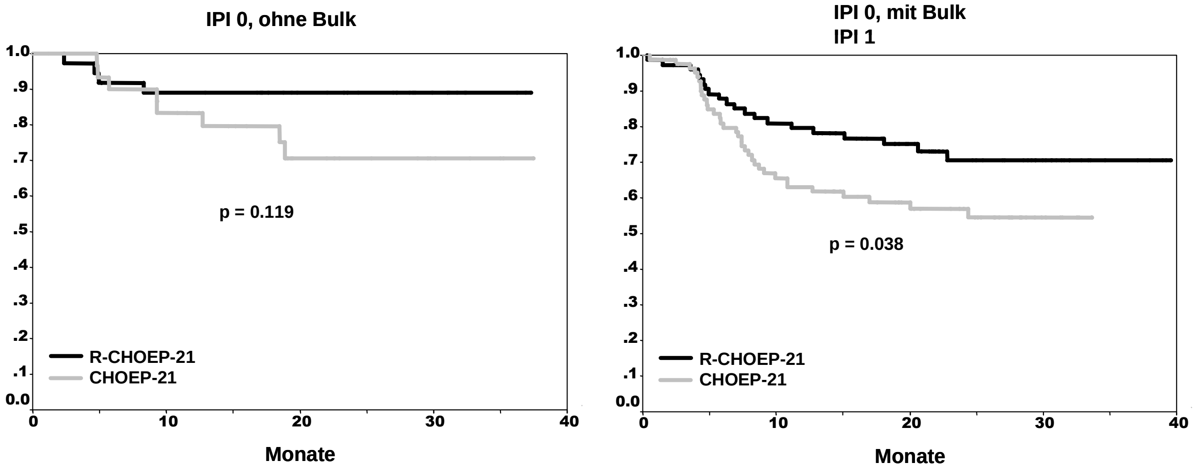

Abbildung 5: TTF für Patienten der MInT-Studie. Patienten mit IPI = 0 und ohne Bulk, die mit Chemotherapie alleine (1) oder Chemotherapie plus Rituximab behandelt wurden (2). Übrige Patienten, die mit Chemotherapie alleine (3) bzw. mit der Kombination Chemotherapie plus Rituximab (4) behandelt wurden.

Die Ergebnisse der MInT-Studie machen deutlich, dass innerhalb der Population der jungen Niedrig-Risiko-Patienten (aaIPI = 0,1) mit aggressiven Lymphomen zwei prognostische Gruppen unterschieden werden müssen: Junge Niedrig-Risiko-Patienten mit günstiger Prognose („young good-prognosis, favourable") sowie solche mit weniger günstiger Prognose („young good-prognosis, less favourable"). Entsprechend sollten diese Gruppen auch im Hinblick auf die Entwicklung neuer spezifischer Therapiestrategien differenziert behandelt werden (Abb. 6).

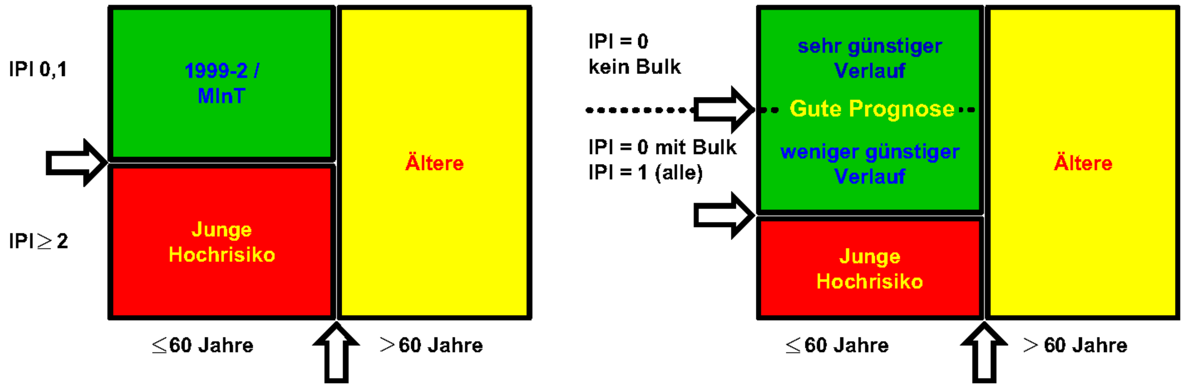

Abbildung 6: Einteilung therapeutischer Gruppen der 2. Studiengeneration (links) und der 3. Studiengeneration der DSHNHL.

2.2 Begründung für die Patientenzielgruppe für DSHNHL 2004-3

Die Ergebnisse der MInT-Studie haben gezeigt, dass junge (d.h. 18-60 Jahre alte) Patienten mit aggressiven Lymphomen, die einen Risiko-Faktor nach IPI und/oder (Einzel- oder Konglomerat-) Lymphome mit einem Durchmesser ≥7.5 cm haben, bereits nach 2 Jahren eine unbefriedigende TTF-Rate haben. Die Therapieergebnisse für diese Gruppe in der MInT-Studie waren wie folgt:

- komplette Remissionen 75%
- Progresse unter Therapie 10%
- TTF nach 3 Jahren 71%
- Überleben nach 2 Jahren 82%

Diese Ergebnisse sind für dieses vergleichsweise junge Patientenkollektiv unbefriedigend und bedürfen dringend einer Verbesserung.

2.3 Standard-Regime/Kontrollarm

Auch wenn teilweise direkte Vergleiche fehlen, so lässt sich ohne Zweifel feststellen, dass die Ergebnisse der Patienten, die innerhalb der MInT-Studie eine Kombination aus 6 Zyklen einer CHOP-21-ähnlichen Chemotherapie zusammen mit 6-maliger Gabe des chimären monoklonalen anti-CD20-Antikörpers Rituximab erhalten haben, die besten sind, die bisher für dieses Patientenkollektiv berichtet wurden. Dies gilt auch, wenn man die MInT-Ergebnisse mit den Ergebnissen nach einer verkürzten Chemotherapie (3-maliges CHOP) in Kombination mit einer recht hochdosierten „involved-field"-Radiotherapie[17] vergleicht, ebenso wie für Ansätze mit einer im Vergleich zu CHO(E)P-intensivierten Chemotherapie wie „High-CHOEP" (s.o.) oder die intensivierte 2-Wochen-CHOP-Variante ACVBP der französischen Arbeitsgruppe GELA[18]. 3 Zyklen dieser Therapie hatten sich in dieser Studie

einer verkürzten Chemotherapie mit 4 Zyklen CHOP in Kombination mit einer „involved-field“-Radiotherapie als signifikant überlegen erwiesen. Da sich die Nebenwirkungen nach 6 Zyklen R-CHEMO in der MInT-Studie nicht von 6 Zyklen der jeweiligen CHOP-ähnlichen Chemotherapie alleine unterscheiden, kann man 6 Zyklen R-CHEMO derzeit als optimale Therapie für dieses Patientenkollektiv ansehen.

Die jüngste Subgruppen-Analyse der MInT-Studie[19] hat die Überlegenheit von CHOEP-21 über CHOP-21 bestätigt; in der Kombination mit Rituximab ergab sich jedoch kein Vorteil von R-CHOEP-21 über R-CHOP-21. Da R-CHOEP-21 jedoch stärkere (insbesondere hämatologische) Nebenwirkungen aufwies und R-CHOP-21 (ein Ein-Tage-Regime) wesentlich einfacher zu applizieren ist als R-CHOEP-21 (ein Drei-Tage-Regime) bleibt daher hinsichtlich des Referenzarmes für die DSHNHL 2004-3 Studie festzustellen:

1) 6 Zyklen R-CHOP-21 in Kombination mit einer Strahlentherapie auf Bulky Disease haben die bisher besten Ergebnisse bei jungen Patienten mit günstigem Risikoprofil erbracht (MInT-Studie[16]).
2) 6 Zyklen R-CHOP-21 in Kombination mit einer Strahlentherapie auf Bulky Disease sind bei Patienten $\leq$60 Jahre gut verträglich und sehr gut durchführbar (mittlere relative Dosisintensität: 99%).
3) Die Tatsache, dass sich das Vorhandensein von Bulky Disease in der MInT-Studie trotz der durchgeführten Strahlentherapie in der multivariaten Cox-Regression als hochsignifikanter prognostischer Faktor erwies ($p = 0.0003$) lässt Zweifel an der Wirksamkeit der Strahlentherapie aufkommen.
4) Eine weitere Verbesserung der Ergebnisse beim Kollektiv der jungen Patienten mit günstigem Risikoprofil und mit ungünstigem Prognoseprofil (IPI = 1 und/oder Bulk) ist dringend indiziert.
5) Für eine solche Studie erscheinen 6 Zyklen R-CHOP-21 als der richtige Referenzarm.

Alle diese Punkte rechtfertigen die Wahl von 6 Zyklen R-CHOP-21 als Referenzarm für die vorliegende Studie DSHNHL 2004-3.

2.4 Begründung des Therapieoptimierungsversuchs

Wie oben ausgeführt, hat die Kombination aus 6 Zyklen CHOP-ähnlicher Chemotherapie mit 6 Zyklen des monoklonalen Antikörpers Rituximab bei der Gruppe der jungen Patienten mit günstigem Risikoprofil zu den besten Ergebnissen geführt, die jemals für diese Patientengruppe berichtet wurden. Allerdings zeigt eine Subgruppenanalyse der MInT-Studie, dass die Prognose junger Patienten mit günstigem Risikoprofil, die einen Risikofaktor entsprechend dem altersadaptierten IPI haben und/oder vor Therapiebeginn Bulky Disease aufweisen, weiterhin unbefriedigend sind und dringend einer Verbesserung bedürfen. Zur Verbesserung der Therapieergebnisse bei diesen Patienten bieten sich mehrere Strategien an, die im Folgenden diskutiert werden.

2.4.1 Intensivierung der Chemotherapie

Die Frage nach der optimalen Zykluszahl einer CHOP-ähnlichen Chemotherapie für die Behandlung aggressiver Lymphome ist offen. Sie ist in der DSHNHL-Studie 1999-1 (RICOVER-60) erstmals Gegenstand einer randomisierten Prüfung, und zwar werden in RICOVER-60 6 vs. 8 Zyklen CHOP-14 jeweils mit und ohne Rituximab randomisiert verglichen. Da es sich bei den Patienten, die sich für 2004-3 qualifizieren, um Patienten in frühen Stadien handelt, erscheint die Erhöhung der Zykluszahl im experimentellen Arm

wenig Erfolg versprechend. Auch die GELA behandelt ihre jungen Patienten in frühen Stadien eines aggressiven Lymphoms mit 3 Zyklen ACVBP, also mit 3/4 der Zykluszahl, die Patienten in fortgeschrittenen Stadien erhalten.

Ebenso wenig wie die Steigerung der Zykluszahl von 6 auf 8 erscheint es angesichts der Datenlage und der Interimsanalyse der DSHNHL 1999-2 Studie („High-CHOEP"; s.o.) als unwahrscheinlich, dass eine Eskalation der Chemotherapiedosis bei diesem Patientenkollektiv zum Erfolg führt. Wie weiter oben ausgeführt, hatte nämlich die erste geplante Zwischenauswertung der 1999-2 Studie ergeben, dass High-CHOEP-21, das nicht nur im Protokoll, sondern auch in der Wirklichkeit der Studienpraxis signifikant höher dosiert werden konnte als CHOEP-21, die Therapieergebnisse bei Patienten mit günstigem Risikoprofil nicht zu verbessern vermochte.

Im Gegensatz zur Erhöhung der Zykluszahl und Eskalation der Chemotherapiedosis führte die Verringerung der Therapieintervalle von 3 auf 2 Wochen nicht nur bei den älteren Patienten der NHL-B2-Studie, sondern auch bei den jungen Patienten mit günstigem Risikoprofil (normale LDH) der NHL-B1 Studie der DSHNHL zu einer signifikanten Lebensverlängerung[20]. Vergleicht man CHOEP-21 und CHOEP-14 mit dem früheren Standard CHOP-21, so bewirkte die Einführung von Etoposid (im CHOEP-21) lediglich eine signifikante Verbesserung des primären Endpunkts TTF gegenüber CHOP-21, während die gleichzeitige Verkürzung der Therapieintervalle (CHOEP-14) alle Endpunkte verbesserte, d.h. neben TTF auch die Rate kompletten Remissionen erhöhte, die Rate primärer Progresse verminderte sowie das Gesamtüberleben jeweils signifikant verlängerte[21]. Der positive Effekt einer Intervallverkürzung war besonders deutlich bei Patienten mit einer erhöhten LDH[22] und bei Patienten mit Bulky Disease[23]. Da wir davon ausgehen, dass ca. 40% der Patienten in dieser Studie eine erhöhte LDH und ca. 60% ein Bulky Disease haben, gehen wir davon aus, dass der positive Effekt einer Intervallverkürzung in dieser Studie stärker ausgeprägt sein dürfte als in der NHL-B1 Studie[24] und eher dem in der NHL-B2-Studie[25] beobachteten entsprechen dürfte.

Auch eine kürzlich erschienene Arbeit der französischen Arbeitsgruppe GOELAMS[21] unterstützt bei einer genaueren Analyse die Annahme, dass im Gegensatz zum suggestiven Titel der Arbeit nicht die Hochdosis, sondern die Dosisdichte („dose density") an der Überlegenheit des intensivierten Armes dieser Studie über 8 Zyklen des klassischen CHOP-21 verantwortlich ist: während die Patienten im experimentellen Arm zunächst zwei Zyklen einer intensivierten, d.h. dosiseskalierten und 15-tägigen CHOP-Variante erhalten hatten, gefolgt von hochdosiertem Methotrexat in Kombination mit Cytosinarabinosid und ihre Therapie einschließlich Hochdosis-BEAM am Tag 67 abgeschlossen war, hatten die Patienten im Kontrollarm zu diesem Zeitpunkt lediglich 4 der 8 geplanten CHOP-21 Zyklen erhalten, für deren Applikation insgesamt 148 Tage vorgesehen waren.

Insgesamt sprechen die publizierten Daten der DSHNHL sowie anderer Gruppen und die noch unveröffentlichten Ergebnisse der DSHNHL 1999-2 dafür, dass im Gegensatz zur Dosiseskalation die Dosisintensivierung über eine Verkürzung der Therapieintervalle („Dosisdensifikation") ein erfolgversprechender Ansatz zur weiteren Verbesserung der Therapieergebnisse bei jungen Patienten mit günstigem Risikoprofil und mit ungünstigem Prognoseprofil darstellt.

Das Protokollkomitee der DSHNHL und die Studienteilnehmer haben daher auf dem Treffen in Hamburg im Juni 2004 einstimmig beschlossen, in der DSHNHL 2004-3 Studie randomisiert zu prüfen, ob die Ergebnisse mit 6 Zyklen R-CHOP-21 durch Verkürzung der Therapieintervalle von 3 auf 2 Wochen (R-CHOP-14) bei diesem Patientenkollektiv zu verbessern sind.

2.4.2 Stellenwert der Strahlentherapie

Der Stellenwert der Strahlentherapie bei der Behandlung hochmaligner Non-Hodgkin-Lymphome ist nicht klar definiert und gründet sich in Ermangelung großer randomisierter Studien auch in Bezug auf die Behandlung von sog. Bulky Disease und Extranodalbefall in erster Linie auf retrospektive Rezidivanalysen.

Obwohl die Strahlentherapie lokal eine sehr wirksame Behandlungsmethode darstellt, nimmt das kurative Potenzial einer alleinigen Strahlentherapie mit zunehmendem Alter ab, und insbesondere ältere Patienten (> 70 Jahre) können durch eine alleinige Strahlentherapie auch im Stadium I nicht geheilt werden[4]. Deshalb ist eine alleinige Strahlentherapie für die Therapie aggressiver Lymphome heute auch für ältere Patienten obsolet.

Auch im Stadium I kann eine alleinige Strahlentherapie in Form einer involved-field-Therapie mit 40 bis 45 Gy einer Chemotherapie bzw. kombinierten Therapie vergleichbare Ergebnisse nur erreichen, wenn eine klinisch nicht erfassbare Dissemination durch eine Staging-Laparotomie ausgeschlossen wird[22;26]. Da diese Maßnahme mit einer signifikanten Morbidität und Letalität assoziiert ist, ist diese Therapiestrategie verlassen worden. Bei nur klinisch "gestageten" Patienten im Stadium I/IE sind die Ergebnisse einer alleinigen Strahlentherapie in größeren Studien nicht befriedigend[27]. Lediglich monozentrische und meist retrospektive Studien zeigen bei Patienten < 60 Jahren mit kleinen Lymphome (< 2.5cm) erkrankungsfreie Überlebensraten von 70-80%[24;25].

Ebenso wie die alleinige Chemotherapie sind die Daten, die einen kombinierten Therapieansatz wissenschaftlich begründen könnten, wenig überzeugend; insbesondere sind die optimale Zahl reduzierter Chemotherapiezyklen sowie Volumen und Dosis einer Strahlentherapie innerhalb eines solchen kombinierten Ansatzes nicht definiert. Manche Studiengruppen wie z. B. die französische GELA haben die Strahlentherapie bei der Behandlung aggressiver Lymphome bei ausgewählten Patientengruppen ganz verlassen.

Mit Kombinationstherapien aus wenigen Zyklen Chemotherapie und involved-field-Radiotherapie mit 35 bis 45 Gy wurden krankheitsfreie Überlebensraten von 75 bis 80% und 5-Jahres-Überlebensraten von 80-90% berichtet[26;27]. Allerdings zeigen längere Nachbeobachtungen, dass es auch nach 5 Jahren noch zu beträchtlichen Rezidivraten kommt, wenn nur wenige Chemotherapiezyklen gegeben wurden. Offenbar ist eine kurze Chemotherapie nicht in der Lage, den malignen Klon zu eliminieren, der für diese späten Rezidive verantwortlich ist. Dass es sich bei diesen Spätrezidiven nicht um Zweittumoren gehandelt hat, konnte durch Klonalitätsuntersuchungen nachgewiesen werden[28].

Ende der 90er Jahre des letzten Jahrhunderts schien eine kombinierte Chemo-Strahlentherapie von aggressiven Lymphomen in lokal begrenzten Stadien (I und II) auf Grund einer frühen Analyse einer randomisierten SWOG-Studie[17] sowie einer ECOG-Studie[29-31] gerechtfertigt. In der ECOG-Studie waren Patienten im Stadium I mit Bulk oder Extranodalbefall und Patienten im Stadium II in 8 Zyklen CHOP alleine oder mit einer zusätzlichen involved-field-Strahlentherapie mit 40 Gy randomisiert worden. Es ergab sich ein grenzgradig signifikanter Vorteil in Bezug auf DFS, nicht jedoch auf OS für die kombiniert behandelten Patienten. In die Studie der SWOG waren Patienten mit extranodalen Lymphomen aufgenommen, Patienten mit Stadium II und Bulky Disease aber ausgeschlossen worden. Eine frühe Auswertung dieser SWOG-Studie hatte einen Vorteil einer Kombination aus 4 Zyklen CHOP-Chemotherapie und einer intensiven (45 Gy) involved-Field-Therapie über eine alleinige Chemotherapie mit 8 Zyklen CHOP ergeben; ein Follow-up dieser Studie, deren Interpretation schon a priori durch zahlreiche methodische Probleme erschwert gewesen war,

zeigte jedoch ein Überschneiden der Kurven nach 7 Jahren[32]. Ebenso ergab eine kürzlich vorgestellte Studie der GELA einen Vorteil für eine in voller Zykluszahl durchgeführte Chemotherapie gegenüber einer verkürzten Chemotherapie in Kombination mit Strahlentherapie[18;33].

Eine *konsolidierende Strahlentherapie als additive Maßnahme* nach einer Chemotherapie mit ungekürzter Zykluszahl wird von einigen Arbeitsgruppen für ursprüngliches Bulky Disease empfohlen. Gestützt wird dieses Vorgehen durch die Ergebnisse einer kleinen randomisierten Studie aus Mexiko mit 88 Patienten, in der die zusätzliche Strahlentherapie auf ursprüngliches Bulky Disease zu einer Verlängerung des rezidivfreien Intervalls und des Gesamtüberlebens führte[34]. In der NHL-B-Studie der DSHNHL waren nach einer Bestrahlung ursprünglicher Bulk-Regionen mit 36 Gy Rezidive in diesen Regionen ebenso selten wie in Nicht-Bulk-Regionen[35]. Allerdings erwies sich „Bulky Disease“ als unabhängiger Risikofaktor trotz der additiv durchgeführten Radiotherapie. Dies ist am ehesten darauf zurückzuführen, dass der ungünstige Einfluss von Bulky Disease weniger durch eine erhöhte Rezidivrate als vielmehr durch eine erhöhte Rate primärer Progresse unter Chemotherapie vermittelt wird.
Dass eine additive Radiotherapie nach Chemotherapie sogar Nachteile haben kann, darauf deuten die Ergebnisse einer Subgruppen-Analyse einer großen Studie der GELA bei Patienten > 60 Jahren ohne IPI-Risikofaktor hin, wo eine additive Strahlentherapie nach 4 Zyklen CHOP-Chemotherapie bei Patienten > 70 Jahren zu einem schlechteren Gesamtüberleben führte[36].

Eine additive Strahlentherapie auf residuelle vergrößerte Lymphknoten nach Chemotherapie sollte nur innerhalb prospektiver Studien durchgeführt werden, da es auch hierfür keine überzeugenden prospektiven Studienergebnisse gibt[37]. Eine Unterscheidung residueller „narbiger“ Veränderungen von verbliebenem vitalen Tumorgewebe war bisher kaum möglich; ob diese Beurteilung durch die Durchführung eines posttherapeutischen FDG-PET[38] sicherer und mit klinischer Relevanz (d.h. besseren Langzeitergebnissen bei entsprechend früher einsetzender „Salvage-Therapie“) möglich sein wird, kann aufgrund der derzeitigen Datenlage nicht entschieden werden.

Die additive Strahlentherapie auf extranodale Befälle ist ebenfalls nicht durch prospektive Studien belegt. Auffallend ist jedoch, dass > 1 Extranodalbefall, der im IPI als unabhängiger Faktor gilt, in den Studien der DSHNHL, wo Extranodalbefälle eine additive Strahlentherapie erhielten, keinen Risikofaktor mehr darstellte[13;20].

Zusammengefasst wird der Stellenwert der Strahlentherapie bei der Behandlung hochmaligner Non-Hodgkin-Lymphome weltweit heftig diskutiert. Die Detektion der Patientengruppen, die potentiell von einer Bestrahlung profitieren bzw. nicht profitieren hat deshalb einen hohen Stellenwert[16,35;37.]

Erfahrungen der NHL-B1-Studie[13], der MInT-Studie[16] sowie der DSHNHL 1999-2 Studie lassen erwarten, dass bei dem Patientenkollektiv, das sich für DSHNHL 2004-3 qualifiziert, ca. 60% der Patienten ein Bulky Disease haben werden. Somit ist diese Studie mehr als jede andere dazu geeignet, prospektiv randomisiert die Frage nach dem Stellenwert einer konsolidierenden Strahlentherapie für ursprüngliches Bulky Disease und/oder Extranodalbefälle zu beantworten. Auf der Sitzung des Protokollkomitees und der Studienteilnehmer im Juni 2004 in Hamburg wurde daher einstimmig beschlossen, in die DSHNHL-Studie 2004-3 diese Fragestellung in Analogie zu einem 2x2 faktoriellen Design zu stellen.

2.4.3 Strahlentherapiedosis

Aggressive Lymphome sind sehr strahlensensibel: Strahlendosen zwischen 36 Gy und 45 Gy reichen aus, um den malignen Klon zu eliminieren. In Kombination mit einer voll dosierten CHOP-ähnlichen Chemotherapie sind eventuell auch geringere Dosierungen ausreichend. In der SWOG-Studie[17], in der ECOG-Studie[30] sowie in der GELA-Studie[18], die jeweils einen kombinierten Ansatz einer Chemotherapie in verkürzter Zykluszahl mit einer „involved-field“-Radiotherapie verglichen, wurden Dosen zwischen 36 Gy und 45 Gy gegeben. In den bisherigen Studien der DSHNHL (NHL-B1, NHL-B2, DSHNHL 1999-1, DSHNHL 1999-2, MInT) wurden 36 Gy auf Bulky Disease gegeben. Die Erfahrungen aus diesen Studien zeigen, dass eine Dosis von 36 Gy auf ein beschränktes Strahlenvolumen auch nach einer Chemotherapie mit voller Zykluszahl sehr gut vertragen wird. Andererseits kann man davon ausgehen, dass Strahlentherapien in einer Dosierung bis zu 40 Gy nach einer CHOP-ähnlichen Chemotherapie in voller Zykluszahl ebenfalls sehr gut verträglich sein dürften, während je nach Lage des Bulky Disease ab Strahlentherapiedosen von 45 Gy nach einer voll dosierten CHOP-ähnlichen Therapie mit verstärkten Nebenwirkungen der Strahlentherapie gerechnet werden muss.

Da in der vorliegenden Studie der Stellenwert einer konsolidierenden Strahlentherapie auf Bulk-Regionen randomisiert geprüft werden soll, haben die Panelmitglieder und die Studienteilnehmer auf ihrer Sitzung im Juni 2004 in Hamburg einstimmig beschlossen, in der vorliegenden Studie eine Strahlentherapiedosis von 39,6 Gy festzulegen. Diese Dosis erscheint einerseits hoch genug, um im Falle einer Unwirksamkeit der Bulk-Bestrahlung den Vorwurf der Unterdosierung zu entkräften; andererseits dürfte es durch die Erhöhung von 36 Gy auf 39,6 Gy zu keiner wesentlichen Erhöhung der Nebenwirkungen kommen.

2.4.4 Definition von Bulky Disease

Die Definition von Bulky Disease ist nicht einheitlich und schwankt zwischen 5.0 cm und 12.5 cm. Ab welchem Durchmesser ein Bulk zum Risikofaktor wird, darüber liegen keine retrospektiven oder gar prospektiven Untersuchungen aus großen randomisierten Studie vor. Auch in der MInT-Studie, in der sich Bulky Disease als wichtigster Prognosefaktor neben dem Therapiearm erwies, wurde der Bulk nicht einheitlich, sondern für jedes teilnehmende Land spezifisch definiert und schwankte zwischen 5 cm und 10 cm. Allerdings traf für 75% der Patienten in der MInT-Studie eine Bulk-Definition von 7.5 cm zu.

Die Tatsache, dass also eine Bulk-Größe von 7.5 cm wesentlich dazu beitrug, dass dieser Parameter in der MInT-Studie so hohe prognostische Bedeutung erlangte, und darüber hinaus eine a priori Definition eines Bulk mit 7.5 cm in den früheren Studien der DSHNHL sich als prognostisch relevant erwiesen hatte, legitimieren einen Cut-off von 7.5 cm für Bulky Disease bei einem maximalen Durchmesser eines einzelnen Lymphknotens oder eines Lymphknotenkonglomerats in dieser Studie. Sowohl der transversale als auch der longitudinale max. Durchmesser eines befallenen Lymphknotens bzw. Lymphknoten-Konglomerats müssen bestimmt werden.

2.4.5 Definition von qualifiziertem Extranodalbefall

Aufgrund des Ausbreitungsmusters bzw. der zu erwartenden Nebenwirkungen werden die folgenden Extranodalbefall-Lokalisationen prinzipiell von der Radiotherapie ausgeschlossen (s. 5.7.3.1):

- Knochenmarkbefall
- Lungenbefall (lokal oder diffus oder beides)
- Pleurabefall/Pleuraerguss (diffus, maligne)
- Perikardbefall (diffus)
- Nierenbefall (auch wenn nur eine Seite befallen)
- Dünndarmbefall (diffus)
- Dickdarmbefall (diffus)
- Leberbefall (lokal oder diffus oder beides)
- Milzbefall (lokal oder diffus oder beides)
- Peritonealbefall (Aszites)

Die übrigen extranodalen Lokalisationen werden prinzipiell als bestrahlbar angesehen und deshalb als qualifizierter Extranodalbefall bezeichnet. In Einzelfällen kann es jedoch auch hier aufgrund der Lokalisation bzw. Ausdehnung des Extranodalbefalls zu Kontraindikationen zur Radiotherapie kommen.

2.4.6 Zusammenfassende Begründung der Studie

Aus den unter 2.4.1 bis 2.4.4 diskutierten Punkten folgt, dass beim Kollektiv der Patienten von 18 bis 60 Jahre mit einem $CD20^{+}$ aggressiven B-NHL und mit günstigem Risikoprofil und weniger günstiger Prognose (IPI = 1 (alle) und IPI = 0 mit Bulk) die Verkürzung der Therapieintervalle von 6 Zyklen der Immunochemotherapie mit Rituximab in Kombination mit dem CHOP-Schema von 3 Wochen (R-CHOP-21) auf 2 Wochen (R-CHOP-14) aufgrund der derzeitigen Erkenntnisse die größte Wahrscheinlichkeit bietet, die dringend notwendige Verbesserung der Ergebnisse bei diesem Patientenkollektiv zu erreichen. Da gleichzeitig der Stellenwert einer zusätzlichen konsolidierenden Strahlentherapie umstritten ist und dringend einer Klärung bedarf, sollen in der vorliegenden Studie 6 Zyklen R-CHOP-21 mit 6 Zyklen R-CHOP-14 jeweils mit und ohne Strahlentherapie von Bulky Disease und/oder Extranodalbefällen prospektiv randomisiert und in Analogie zu einem 2 x 2 faktoriellen Design geprüft werden. Ziel ist es zu zeigen, dass die Verkürzung der Therapieintervalle von 3 auf 2 Wochen bzw. die Durchführung der Bulkbestrahlung zu einer Verbesserung der TTF-Rate nach 3 Jahren um 10% führt. Wir gehen davon aus, dass eine Rekrutierung von 214 Patienten pro Jahr über 5 Jahre die Fragestellung mit ausreichender Power beantworten wird.

3 Studienplan

3.1 Studiendesign

Die Studie 2004-3 der DSHNHL ist eine prospektive randomisierte Therapieoptimierungsstudie. Sie prüft, ob die Ergebnisse einer Kombination von 6 Zyklen einer kombinierten Immuno-Chemotherapie mit Rituximab in Kombination mit dem CHOP-Schema verbessert werden können, wenn man die Therapieintervalle von 3 Wochen (R-CHOP-21) auf 2 Wochen verkürzt (R-CHOP-14). Gleichzeitig wird geprüft, ob die konsolidierende Bulk- und/oder qualifizierte E-Befallbestrahlung die Therapieergebnisse verbessert. Es handelt sich um eine offene multizentrische Studie.

3.2 Teilnehmende Institutionen und Zahl der Patienten

An der Studie 2004-3 der DSHNHL nehmen prinzipiell alle Institutionen (aktuell 297) teil (s. Anhang 14.5), die innerhalb der DSHNHL kooperieren. Wir gehen davon aus, dass diese Zentren in einer ca. 9-jährigen Rekrutierungsphase, zwischen Oktober 2005 und September 2014 1072 Patienten einbringen werden.

3.3 Dauer der Studie

Die Phase der Immuno-Chemotherapie in dem Standardarm dauert 18 Wochen (6 x R-CHOP-21), in dem experimentellen Arm beträgt sie 12 Wochen (6 x R-CHOP-14). Im Falle einer zusätzlichen Strahlentherapie von Bulky Disease, Bestrahlung von qualifizierten E-Befällen sowie einer prophylaktischen Bestrahlung des kontralateralen Hodens bei Hodenbefall verlängert sich diese Therapiedauer einschließlich der Therapiepausen um ca. 8 Wochen.

3.4 Abbruch der Studie

3.4.1 Abbruch der Studie für einzelne Patienten

3.4.1.1 <u>Nachträgliche Nicht-Qualifikation</u>

Da die vorliegende Studie entsprechend international vereinbarter Kriterien[39] entsprechend dem Intention-to-Treat analysiert werden wird, wird kein Patient aus der Studie ausgeschlossen. **Einziger Ausschlussgrund ist die Rücknahme der Einwilligung zur Studienteilnahme durch den Patienten.** Wenn hingegen ein anderes Ausschlusskriterium nach Aufnahme in die Studie auftritt oder sich herausstellt, dass ein Ausschlusskriterium bereits zum Zeitpunkt der Meldung vorgelegen hat (hierzu gehören neben allen unter 4.2 genannten Ausschlusskriterien insbesondere die nachträgliche Änderung der histologischen Diagnose durch die Referenzpathologie), wird der Patient nicht aus der Studie ausgeschlossen. **Die nachträgliche Nicht-Qualifikation des Patienten wird dem behandelnden Arzt von der Studienleitung mitgeteilt. Die weitere Dokumentation der**

nachträglich nicht qualifizierten Patienten entspricht also genau der der qualifizierten Patienten.

3.4.1.2 Abbruch der Therapie

Folgende Gründe können zu einem Abbruch der Therapie eines Patienten führen:

- Nichtansprechen auf die Therapie entsprechend den Protokollkriterien
- schwere Protokollverletzung
- Non-Compliance des Patienten
- exzessive Toxizität
- Wunsch des Patienten
- Entscheidung des behandelnden Arztes
- Abbruch des Kontaktes durch den Patienten

Der Grund für den Therapieabbruch muss schriftlich dokumentiert und der Studienleitung mitgeteilt werden. Auch nach Abbruch der Therapie sind diese Patienten weiter zu dokumentieren (Remissionsstatus, Überleben mit und ohne Lymphom).

3.4.2 Abbruch der Studie bzw. einzelner Studienarme

Folgende Gründe können zu einem vorzeitigen Abbruch der Studie bzw. eines Studienarmes führen:

- Auftreten schwerer Nebenwirkungen der Therapie
- gehäuftes Auftreten von therapiebedingten Todesfällen
- nachweisliche Unterlegenheit eines Therapiearmes (Zwischenauswertung!)
- neue Erkenntnisse aus anderen Studien oder Publikationen
- nicht ausreichende Rekrutierungsrate
- Häufung von Protokollverletzungen in einem Therapiearm

Bei Auftreten dieser Ereignisse unterrichtet die Studienleitung das Protokollkomitee, das dann über den Abbruch der Studie innerhalb eines Monats entscheiden muss. Kann im Protokollkomitee keine einheitliche Meinungsbildung herbeigeführt werden, wird die Empfehlung eines unabhängigen Komitees (Daten- und Sicherheits- Monitoringkommite, s. 0.3.2) eingeholt, dem auf dem Gebiet der Therapieforschung bei Lymphomen international anerkannte Experten angehören. Sollte auch dies zu keiner einheitlichen Meinungsbildung im Protokollkomitee führen, entscheidet die Studienleitung über den Abbruch der Studie nach Einholung eines Votums der Ethikkommission.

4 Aufnahmekriterien

4.1 Einschlusskriterien

1) Alter:
18- 60 Jahre

2) Risikogruppe:
Niedrig-Risiko-Gruppe, weniger günstiger Verlauf
- (altersadjustierter) IPI = 1: alle Patienten
- (altersadjustierter) IPI = 0: nur Patienten mit Bulk (Definition s. Anhang 14.10, größter Einzel- oder Konglomerat-Befall durch Lymphom muss Durchmesser ≥ 7.5 cm haben)

3) Histologie:
Diagnose eines unbehandelten CD20-positiven aggressiven B-Zell-Lymphoms, gesichert durch eine Exzisionsbiopsie eines Lymphknoten oder durch eine ausreichend große Biopsie eines extranodalen Befalls, wenn kein Lymphknotenbefall vorliegt. Im einzelnen können folgende Entitäten im Rahmen dieser Studie behandelt werden:

- **B-NHL:**
  - follikuläres Lymphom III°b
  - follikuläres Lymphom III° und diffuses B-Zell Lymphom
  - diffus großzelliges B-Zell-Lymphom
    - centroblastisch
    - immunoblastisch
    - plasmoblastisch
    - anaplastisch-großzellig
    - T-Zell-reiches B-Zell Lymphom
  - primäres Ergusslymphom
  - intravasales B-Zell-Lymphom
  - primär mediastinales B-Zell-Lymphom
  - Burkitt-like Lymphom
  - Burkitt-Lymphom
  - aggressives Marginalzonenlymphom (monozytoid)
  - Mantelzell-Lymphom, blastoid

- **T-NHL:**
  T-NHL können nicht in diese Studie aufgenommen werden! Stattdessen sollen diese Patienten in Studien der DSHNHL aufgenommen werden, die speziell für diese Patienten entwickelt wurden (bitte aktuelle Rückfrage im Studiensekretariat).

4) Allgemeinzustand:
Allgemeinzustand ECOG 0-2 zum Zeitpunkt der Randomisation; Def. s. Anhang 14.11.

5) Vorliegen der Teilnahmeerklärung des Zentrums und der schriftlichen Einverständniserklärung des Patienten

4.2 Ausschlusskriterien

6) schon begonnene Therapie des Lymphoms (außer Vorphasetherapie dieser Studie)
7) schwere Begleiterkrankung bzw. eingeschränkte Organfunktion (insbes. Einschränkung der linksventrikulären Funktion oder schwerwiegende Herzrhythmusstörungen)
8) Thrombozyten < 100 000/mm^3, Leukozyten < 2 500/mm^3
9) bekannte Überempfindlichkeit gegen eingesetzte Medikamente
10) bekannte HIV-Positivität
11) aktive Hepatitis Infektion
12) Verdacht auf reduzierte Compliance des Patienten
13) simultane Teilnahme an anderen Therapiestudien
14) Chemo- oder Strahlentherapie bei früherer Erkrankung
15) vorausgegangene immunsuppressive Therapie mit Zytostatika
16) simultan vorliegende andere Tumorerkrankung und/oder Tumorerkrankung in den vorausgegangenen 5 Jahren (außer CIS und Basaliom der Haut)
17) Schwangerschaft und Stillzeit
18) > 1 Risikofaktor nach altersadaptiertem IPI (LDH > ONW, Stadium III/IV, ECOG > 1)
19) ZNS-Befall des Lymphoms (intrazerebral, meningeal, intraspinal)
20) MALT-Lymphome
21) Nichtvorliegen der Einschlusskriterien

Patienten mit primärem ZNS-Befall oder MALT-Lymphom sind nicht Gegenstand dieser Studie. Wir empfehlen für letztere Patienten die Einbringung in die Studien der Deutschen Studiengruppe GIT - NHL (Prof. Berdel, Dr. Koch, Münster bzw. der Deutschen Studiengruppe PCNSL (Prof. Thiel, Berlin; Prof. Weller, Tübingen). Patienten mit lymphoblastischem Lymphom sollten innerhalb der multizentrischen ALL-Studie (Prof. Hoelzer, Frankfurt) behandelt werden.

5 Individueller Studienablauf

5.1 Durchführung der Staging-Untersuchungen

5.1.1 Obligate Untersuchungen

1) Anamnese (Beginn der Beschwerden, B-Symptome, Allgemeinzustand in ECOG)
2) klinische Untersuchung
3) Laboruntersuchungen:
   - Blutbild mit Differentialblutbild
   - LDH
   - GPT
   - GOT
   - alkalische Phosphatase (Serum)
   - γ-GT
   - Bilirubin
   - Kreatinin
   - Elektrolyte (Na, K, Cl, Ca) nur im Falle von erhöhtem Kreatinin
   - HIV-Serologie (nach gesonderter Aufklärung und Einverständniserklärung)
   - Hepatitis-Serologie
   - Schwangerschaftstest
4) CT Hals/Thorax/Abdomen
5) Knochenmarkbiopsie (Histologie und Zytologie)
6) Lumbalpunktion mit Liquorzytologie bei Hodenbefall
7) EKG
8) Echokardiografie (alternativ: nuklearmedizinische Bestimmung der Ejektions-fraktion) vor Therapiebeginn (Empfehlung der Ärztekammer Nordrhein)
9) Messung Lungendiffusionskapazität (bei Pat. mit vorbestehender Lungenerkrankung)

5.1.2 Fakultative Untersuchungen

1) Laboruntersuchungen:
   - BSG
   - Gesamteiweiß und Albumin mit Eiweißelektrophorese und Immunelektrophorese (Paraprotein)
   - Immunglobuline IgG, IgA, IgM
   - beta2-Mikroglobulin
2) Skelettszintigramm
3) Rö-Thorax in 2 Ebenen im Stehen
4) Abdominalsonogramm
5) Gastroskopie (obligat bei Befall der Tonsillen)
6) HNO-ärztliche Untersuchung (obligat bei zervikalem Befall)
7) Hämoccult
8) Lumbalpunktion mit Liquorzytologie, sofern nicht obligat (s. 5.1 a)
9) Gallium-Szintigraphie
10) Positronenemissionstomographie
11) Kernspintomographie
12) Halssonographie

13) Leberbiopsie
14) Fertilitätsuntersuchungen, ggfls. Spermaasservation

5.2 Stadieneinteilung des Patienten/Risikozuordnung

Aufgrund der Ergebnisse der Staginguntersuchungen erfolgt die Zuordnung des Krankheitsstadiums des Patienten entsprechend den Kriterien der Konferenz von Ann Arbor (s. Anhang 14.9.1) und Cotswolds[40]. Außerdem erfolgt entsprechend der Zahl der aufgrund der Untersuchungsergebnisse vorliegenden Risikofaktoren die Zuordnung des Patienten zu einer der 4 Risikogruppen entsprechend des „International Prognostic (age adjusted) Index“ (IPI) in:

1) niedrige Risikogruppe
2) niedrig-intermediäre Risikogruppe
3) intermediär-hohe Risikogruppe
4) hohe Risikogruppe

Die Kriterien des altersadjustierten IPI sowie die Definitionen für „Bulky Disease“ und „E-Befall“ finden sich im Anhang 14.9 bis 14.12, zum Studienprotokoll. **Alle Patienten im Alter von 18 bis 60 Jahren mit altersadaptiertem IPI = 1 sowie Patienten mit IPI = 0, sofern sie ein Bulky Disease haben, können an dieser Studie teilnehmen.**

5.3 Zentrale Beurteilung der prätherapeutischen bildgebenden Diagnostik

Um einerseits standardisierte Definitionen von Bulky Disease und Extranodalbefällen zu erreichen, und andererseits einen Bestrahlungsvorschlag zu machen, muss die gesamte prätherapeutische bildgebende Diagnostik (CT, bzw. MRT) **aller in die Studie eingebrachten Patienten, unabhängig vom Randomisationsarm und vom Ausbreitungstyp,** zusammen mit den diagnostischen Radiologen im strahlentherapeutischen Referenzzentrum beurteilt werden. Dazu muss die gesamte prätherapeutisch erfolgte Bildgebung inklusive Befunde an das Studiensekretariat in Homburg geschickt werden. Dort werden sie kopiert und sofort an das behandelnde Zentrum zurückgeschickt.
Ein Bestrahlungsplan wird nur für Patienten in den Armen ARX und BRX vom Referenzzentrum erstellt und dem behandelnden Arzt zugesandt.

5.4 Aufklärung des Patienten über die Studie

Die Aufklärung des Patienten erfolgt vor jeglicher lymphomspezifischer Therapie durch den behandelnden Arzt.
Der Patient wird vom behandelnden Arzt (möglichst in Anwesenheit eines Zeugen) in verständlicher Sprache über die Diagnose eines aggressiven NHL und den derzeitigen Stand der Wissenschaft hinsichtlich Diagnostik und Therapie dieser Erkrankung sowie über die Ziele der Studie aufgeklärt. Die Aufklärung beinhaltet außerdem Informationen über zu erwartende und mögliche Wirkungen und Nebenwirkungen der Therapie sowie über den Versicherungsschutz, den der Patient bei einer Studienteilnahme genießt. Es muss gewährleistet sein, dass der Patient sich darüber bewusst ist, dass er jederzeit frei über seine Teilnahme an der Studie entscheiden kann, dass er diese Entscheidung jederzeit wieder rückgängig machen kann und dass ihm aus einer Nicht-Teilnahme an der Studie kein Nachteil

entsteht. Der Patient wird weiterhin darüber informiert, dass im Falle seiner Einwilligung zur Studie, zum Zwecke der Dokumentation Einsicht in seine Krankenakte genommen wird und personenbezogene, für die wissenschaftliche Überwachung des Krankheitsverlaufes notwendige Daten gesammelt und ausgewertet werden. Ziel und Zweck dieser Datensammlung werden dem Patienten erklärt. Weiterhin soll sich der Patient verpflichten, alle Gesundheitsstörungen, die im Verlauf der Therapie oder später auftreten und mit der Therapie in Zusammenhang stehen könnten (z.B. späte Veränderungen des Blutbildes) unmittelbar seinem behandelnden Arzt zu melden. Der Patient wird außerdem darüber aufgeklärt, dass regelmäßige Kontrolluntersuchungen nach Abschluss der Therapie in seinem Interesse, aber besonders auch im Interesse zukünftiger Patienten über viele Jahre hinweg durchgeführt werden und die Ergebnisse dieser Nachuntersuchungen der Studienzentrale gemeldet werden sollen.
Die Einverständniserklärung des Patienten erfolgt schriftlich und wird vom aufklärenden Arzt gegengezeichnet. Sie beinhaltet ausdrücklich die Zustimmung zur Erfassung der Patienten-daten sowie ihrer Weitergabe an die Studienzentrale und ihrer Auswertung in anonymer Form sowie die Zustimmung zur Durchführung wissenschaftlicher Begleituntersuchungen. Außerdem stimmt der Patient zu, dass er von der Studienzentrale direkt angeschrieben wird, wenn die Studienzentrale über den behandelnden Arzt die für das Erreichen der Studienziele notwendige Information nicht mehr erhalten kann. Das Protokoll der Einverständniserklärung wird von Patient und Arzt sowie gegebenenfalls durch den Zeugen unterzeichnet.
Formblätter für die Patienteninformation und Einverständniserklärung sind im Anhang 14.2 bis 14.4 des Studienprotokolls zu finden. Die Originale der Einverständniserklärung und des Protokolls der Einverständniserklärung verbleiben beim Arzt. Dem Patienten werden die zugehörigen Kopien, sowie die Patienteninformation ausgehändigt. Zusätzlich erhält der Patient eine Kopie der Patientenversicherung.

5.5 Meldung des Patienten für die Studie

Unmittelbar nach Abschluss der Staging-Untersuchungen, Aufklärung des Patienten über die Studie und Einholung der schriftlichen Einverständniserklärung und vor Beginn jeglicher lymphomspezifischer Therapie (in Ausnahmefällen später, s. unten, aber spätestens vor Beginn der Immuno-Chemotherapie) erfolgt die Meldung des Patienten an die Studienzentrale in Homburg durch den behandelnden Arzt.

Randomisation im Studiensekretariat der DSHNHL

Telefon: 06841/16-23084
Fax: 06841/16-23004
E-mail: dshnhl@uks.eu

Um einen Patienten in die Studie einzubringen senden Sie bitte an das Studiensekretariat Homburg per Fax *(Fax Nr.: 06841/16-23004)* Meldebogen I, Meldebogen II und den Stagingbogen sowie die Primärpathologie und die Einverständniserklärung des Patienten.
Sollten Rückfragen von Seiten des Studiensekretariates bestehen, erfolgt ein Anruf aus dem Studiensekretariat Homburg.
Für die Randomisation werden folgende Informationen benötigt:

- Institution und behandelnder Arzt
- Name des Pathologen

- Name und Unterschrift des Strahlentherapeuten (obligat für alle Patienten)
- Identifikation des Patienten
- Alter
- Geschlecht
- Histopathologischer Subtyp (WHO-Klassifikation) - und Feststellung, ob CD20+ B-NHL
- Erfüllung aller Einschlusskriterien
- Fehlen aller Ausschlusskriterien
- IPI-Kriterien:
  - LDH-Wert sowie oberer Normwert des Labors
  - Allgemeinzustand ECOG
  - Ann Arbor Stadium
  - Vorliegen von Extranodalbefall (Anzahl, Ort)
- Vorliegen von BULK (Lokalisation)
- Lokalisationen des Lymphombefalls
- Hämatologischer Status

Anmerkung:

Da bei den Studien der DSHNHL die Studienzuordnung und Stratifizierung bei der Randomisation entsprechend dem International Prognostic Index[1] erfolgt, müssen Stadium, Allgemeinzustand, Zahl der Extranodalbefälle sowie LDH zum Zeitpunkt der Randomisation bekannt sein und festliegen! Bei der Randomisation ist ein LDH-Wert anzugeben, der vor Therapiebeginn erhoben wurde. Es ist sorgfältig darauf zu achten, dass die Bestimmung nicht durch Hämolyse verfälscht wird. Ferner sind immer die laborspezifischen Normwerte anzugeben. Ein weiteres Stratifizierungsmerkmal ist das Vorliegen von Bulky Disease, was ebenfalls vor Randomisation bekannt sein und festliegen muss.

5.6 Aufnahme des Patienten in die Studie

Die Mitteilung des Ergebnisses der Randomisation erfolgt durch umgehenden Rückruf aus dem Studiensekretariat in Homburg. **Außerdem erhält die behandelnde Institution eine schriftliche Bestätigung der Randomisation per Fax und mit gleicher Post.**
Als qualitätssichernde Maßnahme ist von allen in die Studie eingebrachten Patienten **unabhängig von einer Bestrahlung** die prätherapeutisch-bildgebende Diagnostik möglichst in elektronischer Form (CD) an das Studiensekretariat in Homburg/Saar zu senden.
Der Primärpathologe erhält ein Schreiben des Leiters des Referenzpanels mit der Bitte um Zusendung der Gewebsblöcke zur referenzpathologischen Beurteilung. Das Studiensekretariat unterrichtet die Referenzpathologen monatlich über neu in die Studie aufgenommene Patienten.
Auf der Basis der prätherapeutisch bildgebenden Diagnostik dokumentiert das Strahlentherapie-Referenz-Panel das Ausmaß der Erkrankung und sendet für die Patienten in den Bestrahlungsarmen einen strahlentherapeutischen Therapieplan an das behandelnde Zentrum.
Im Rahmen des Randomisationsgesprächs wird der meldende Arzt über den Bedarf an Primärmaterial für die vom Scientific Advisory Board unterstützten wissenschaftlichen Begleitprojekte aufgeklärt und bei der Versendung des Materials von der Studienleitung logistisch unterstützt. In der Patienteninformation und der Einverständniserklärung werden die Patienten gebeten, den wissenschaftlichen Untersuchungen zuzustimmen und das primäre Lymphknotenmaterial für diese Untersuchungen der Studienzentrale zur Verfügung zu stellen.

Am Tag der Randomisation wird vom Studiensekretariat aus die Dokumentationsmappe an den meldenden Arzt verschickt.

5.7 Durchführung der Studientherapie

Die Vorphasetherapie kann in Ausnahmefällen vor Randomisation beginnen. Die Hauptphase der Therapie darf jedoch erst nach erfolgter Randomisation beginnen, muss sich dann aber direkt an die Vorphase anschließen. Die Randomisation muss spätestens vor der Studientherapie mit R-CHOP-21 / R-CHOP-14 erfolgen. Somit ist der notfallmäßige Beginn der Vorphasetherapie, z.B. bei ausgedehnter Tumormasse oder schlechtem Allgemeinzustand, auch vor Meldung in der Studienzentrale erlaubt. Die Vorphasetherapie ist nicht obligat und geht der Haupttherapiephase mit dem R-CHOP-21/14-Schema voran, die unabhängig vom Studienarm 6 Zyklen dauert. Nach dem 3. Zyklus erfolgt ein Zwischenstaging. Für alle Patienten, die zu diesem Zeitpunkt kein Ansprechen zeigen (PRO), wird eine Salvage-Therapie empfohlen. Auch die Salvage-Therapie soll innerhalb einer prospektiven Studie erfolgen. Näheres regelt ein eigenes Studienprotokoll für die Salvage-Therapie (ggf. Rückfrage). Nach dem 6. Zyklus erfolgt ein Abschlussstaging nach Chemotherapie. **Patienten mit Bestrahlungsindikation, deren Therapieerfolg im Abschlussstaging nach Immuno-Chemotherapie PR/CRu/CR lautet, erhalten eine konsolidierende Bestrahlung mit 39,6 Gy auf initiale BULK-Läsionen und/oder qualifizierte E-Befälle bzw. den kontralateralen Hoden bei Hodenbefall (ARX und BRX)!**

Patienten mit Bestrahlungsindikation, die im Abschlussstaging nach Chemotherapie weniger als eine CR, CRu oder PR (hinsichtlich der Besonderheiten einer PR verweisen wir auf Abschnitt 5.9.3) erreicht haben, sind prinzipiell progredient und somit weiter therapiebedürftig (Salvagetherapie). Dies ist in der Regel eine weitere Chemotherapie, kann aber nach Ermessen des behandelnden Arztes auch eine Strahlentherapie sein.

Patienten ohne Bestrahlungsindikation, deren Therapieerfolg im Abschlussstaging nach Chemotherapie PR/CRu lautet, erhalten 4 Wochen nach dieser Untersuchung eine Remissionsbestätigung, in der alle noch auffälligen ‚Restbefunde zur zweifelsfreien Festlegung des Therapieerfolges kontrolliert werden müssen. Aufgrund der biologischen Wachstumsdynamik aggressiver Lymphome sind alle Patienten, die in dieser Remissionsbestätigung keinen Progress haben, in CR/CRu! Patienten ohne Bestrahlungsindikation, die in dieser Remissionsbestätigung (4 Wochen nach Abschlussstaging nach Chemotherapie) weniger als eine CR oder CRu erreicht haben, sind prinzipiell progredient und somit weiter therapiebedürftig (Salvagetherapie; hinsichtlich der Besonderheiten einer PR verweisen wir auf Abschnitt 5.9.3). Dies ist in der Regel eine weitere Chemotherapie, kann aber nach Ermessen des behandelnden Arztes auch eine Strahlentherapie sein.

Das erste Follow-up erfolgt durch einen Internisten **<u>in allen Armen</u> 3 Monate nach Abschlussstaging nach Chemotherapie**

Angesichts der Intensität der Therapie dieser Studie wird eine engmaschige klinische Kontrolle empfohlen. Die Patienten sollten mindestens einmal pro Woche von einem erfahrenen Arzt gesehen werden, damit klinische Nebenwirkungen der Chemotherapie (z.B. Mucositis, Polyneuropathie, Verschlechterung des Allgemeinzustandes) rechtzeitig erkannt und ggf. entsprechende Maßnahmen eingeleitet werden können.

5.7.1 Vorphase-Therapie

Alle Patienten können vor Beginn eine einwöchige Vorphase bestehend aus Prednison und Vincristin erhalten:

**Vincristin**	1 mg	i.v.	Tag* -6	einmalige Gabe
**Prednison**	100 mg	p.o.	Tag* -6 bis Tag 0	7 Tage

*Tag 1 = 1. Therapietag des 1. R-CHOP-21/14 Zyklus

Diese Vorphase verhindert ein Tumorlysesyndrom bei großen Tumormassen, verbessert den Allgemeinzustand der Patienten und führt zu einer Reduktion der Toxizität der Chemotherapie des ersten Zyklus. Die **Vorphasen-Therapie ist zwar nicht obligat, sie wird aber empfohlen und kann auch in verkürzter Form gegeben werden!** Eine ausreichende Flüssigkeitszufuhr und sorgfältige Supportivmaßnahmen (s.u.) sind zu gewährleisten.

5.7.2 Chemotherapie

Die Dosierung von R-CHOP unterscheidet sich in den Armen A (R-CHOP-21) und B (R-CHOP-14) nicht:

- Arm A: 6 x R-CHOP-21
- Arm B: 6 x R-CHOP-14

R-Chop-Schema:

**Cyclophosphamid**	750 mg/m^2	i.v.	d1
**Doxorubicin**	50 mg/m^2	i.v.	d1
**Vincristin**	1,4 mg/m^2, max 2 mg	i.v.	d1
**Prednison**	100 mg (absolut)	p.o.	d1-5
**Rituximab**	375 mg/m^2	i.v.	d0 oder d1

Wiederholung: Tag 22 (R-CHOP-21), bzw. Tag 15 (R-CHOP-14); insgesamt 6 Zyklen

GCSF: siehe 5.7.2.4

"Ausschleichen" des Prednison:

Nach Absetzen des Prednison kann es zu starker Abgeschlagenheit kommen; wir empfehlen daher ggf. in folgenden Zyklen ein Ausschleichen des Prednison mit 50 mg am Tag 6, 25 mg am Tag 7 und 12,5 mg am Tag 8.
R-CHOP-21 wird am Tag 22, R-CHOP-14 am Tag 15 wiederholt. Voraussetzung für die Fortsetzung der Therapie sind:

1) Durchschreiten des Nadirs der Leukozyten und Thrombozyten
2) Leukozytenwerte > 2500/mm3 am Tag 22 bzw. Tag 15 nach Absetzen von G-CSF (Lenograstim®)und
3) Thrombozytenwerte > 80 000/mm3 am Tag 22 bzw. Tag 15
4) keine aktive Infektion

5) keine Thrombozytopenie < 20.000/mm³ während des gesamten vorherigen Zyklus
6) Dauer der Leukozytopenie < 1000/mm³ max. 4 Tage während des gesamten vorherigen Zyklus
7) keine schwerwiegende Organ- oder sonstige Toxizität

Bei Nicht-Erreichen der Grenzwerte von Leukozyten und Thrombozyten am Tag 22 bzw. Tag 15 wird der Beginn des nächsten Zyklus zunächst um 3 Tage, falls die Grenzwerte dann immer noch nicht erreicht sind, um weitere 3-4 Tage verschoben. Solche Verzögerungen sind ebenso wie prolongierte Leukozytopenien Indikation zur G-CSF-Gabe (Lenograstim®). Die G-CSF-Gabe soll in diesen Fällen bis Erreichen der notwendigen Leukozytenzahlen (s.o.) fortgesetzt und in den nächsten Zyklen ab Tag 4 wieder aufgenommen werden. Sollte mehr als 1 Woche Verzögerung nötig sein, so erfolgt eine Dosisreduktion (s. 5.7.2.1.).

5.7.2.1 Dosisreduktion

Werden die Kriterien für die Fortsetzbarkeit (s.5.7.2) auch nach einem Therapieaufschub um 1 Woche am Tag 29 nach R-CHOP-21 und am Tag 22 nach R-CHOP-14 nicht erreicht, wird unter 3-tägigen Blutbildkontrollen abgewartet, bis diese Werte erreicht sind. Der nächste Zyklus erfolgt dann in reduzierter Dosis:

	Cyclo-phosphamid	Doxorubicin	Vincristin	Prednison	Rituximab
**Therapie-verzögerung 0-7 Tage:**	keine Reduktion				
**Therapie-verzögerung 8-14 Tage**	75%	75%	100%	100%	100%
**Therapie-verzögerung > 14 Tage**	50%	50%	100%	100%	100%

Dosisreduktion bei Übergewicht:

Gemäß den “ASCO-Guideline on Appropriate Chemotherapy Dosing for Obese Adult Patients with Cancer” von April 2012 empfehlen wir, dass zur Behandlung übergewichtiger Patienten die volle gewichtsbasierte Chemotherapiedosis gegeben wird (Ausnahme: Vincristin wird bei 2 mg absolut „gekappt“). Die behandelnden Ärzte sollten auf alle behandlungsassoziierten Toxizitäten auf die gleiche Art und Weise reagieren wie bei nicht übergewichtigen Patienten. Sollte eine Dosisreduktion als Reaktion auf eine Toxizität durchgeführt worden sein, sollte eine Wiederaufnahme der vollen gewichtsbezogenen Dosierung in Erwägung gezogen werden, insbesondere wenn sich die mögliche Ursache für die Reduktion (z.B. eingeschränkte Nieren- oder Leberfunktion) normalisiert hat. Es gibt keinen Hinweis für die Notwendigkeit einer größeren Dosisreduktion bei übergewichtigen Patienten im Vergleich zu normalgewichtigen Patienten.[41]

Außerdem sind Dosisreduktionen einzelner Medikamente beim Auftreten anderer Toxizitäten (z.B. Polyneuropathie, schwere Mucositis) zu erwägen. In diesen Fällen wird eine Rücksprache mit der Studienleitung empfohlen.

5.7.2.2 Rituximab-Applikation

Rituximab 375 mg/m^2 i.v., Beginn jeweils 48 bis 2 h vor Beginn von CHOP-21/14.
Wiederholung: **alle 3 bzw. 2 Wochen, insgesamt 6 mal, synchron mit der Gabe von CHOP-21/14.**
Die Antikörperlösung wird 1:10 in 0.9% NaCl verdünnt (maximale Antikörperkonzentration: 1 mg/ml). Stärkeres Schütteln der Lösung ist zu vermeiden, um ein Ausfallen des Antikörpers zu verhindern.
Da Rituximab-Nebenwirkungen insbesondere bei der Erstapplikation auftreten, muss die erste Rituximab-Gabe unter stationären Bedingungen und mindestens 24 Stunden vor der Applikation der CHOP-Chemotherapie gegeben werden, damit Nebenwirkungen, die durch den Antikörper bedingt sind, von Nebenwirkungen der Chemotherapie klinisch möglichst eindeutig voneinander zu trennen sind! Vor der ersten Gabe ist der Patient ausreichend zu hydrieren, außerdem sollen die Patienten vor der ersten Gabe Allopurinol erhalten. Ein Tumorlysesyndrom ist nach vorausgegangener Vorphase zwar sehr unwahrscheinlich, ihm sollte aber durch diese Maßnahmen dennoch vorgebeugt werden.
Rituximab darf nicht als Bolus-Injektion gegeben werden!
Vor der ersten Rituximab-Gabe erfolgt eine Vorbehandlung mit Paracetamol (1000 mg) und 50 bis 100 mg Diphenhydraminhydrochlorid. Rituximab wird über einen peripheren oder zentralen Venenkatheter appliziert. Vor der ersten Infusion sollen Adrenalin und Diphenhydraminhydrochlorid für den Fall einer allergischen Reaktion vorgehalten werden. Alle notwendigen Einrichtungen zur Behandlung eines anaphylaktischen Schock müssen bereit gehalten werden.
Während der ersten Stunde der Rituximab-Infusion werden Blutdruck, Pulsfrequenz, Atemfrequenz alle 15 Minuten gemessen. Falls keine Nebenwirkungen während der ersten Stunde auftreten, kann die Infusionsrate auf 50 mg/h bis auf 300 mg/h erhöht werden, ab der zweiten Infusion bis auf 400 mg/h. Während der Infusion von Rituximab können Fieber und Schüttelfrost auftreten. In diesen Fällen soll die Antikörpergabe unterbrochen werden. Nach Verschwinden der Beschwerden kann die Infusion mit der halben Infusionsrate wieder fortgeführt werden. Nach Abschluss der Infusion sollte der venöse Zugang noch für eine Stunde beibehalten werden.
Grundsätzlich soll die Applikation von Rituximab synchronisiert mit der Chemotherapie gegeben werden. In den seltenen Fällen, wo Rituximab schon für den neuen Zyklus appliziert wurde, es dann aber doch zu einer unvorhergesehenen Verzögerung der Chemotherapie kommt, erfolgt die nachfolgende Rituximab-Applikation erst mit dem nächsten Chemotherapie-Zyklus.

5.7.2.3 ZNS-Prophylaxe/RX bei testikulärem Befall

Es erfolgt keine ZNS-Prophylaxe, auch nicht bei einem Hodenbefall, da sie bei bei der Kombinationsbehandlung CHOP mit Rituximab unwirksam war[42].
Allerdings sollte im Falle eines Hodenbefalls eine Liquorpunktion zum Ausschluss eines latenten ZNS-Befalls durchgeführt werden. Des Weiteren wird grundsätzlich eine Bestrahlung des kontralateralen Hodens bei testikulärem Lymphombefall empfohlen, weil sich gezeigt hat, dass diese prophylaktische Bestrahlung die Zahl des Auftretens von Lymphomen in den kontralateralen Hoden reduziert.Die Bestrahlung soll mit einer Fraktionierung von 1,8 Gy 5x/Woche bis zu einer Gesamtdosis von 30,6 Gy erfolgen.

5.7.2.4 G-CSF-Therapie

Patienten im Therapiearm A und ARX (R-CHOP-21):
erhalten kein G-CSF, wenn es nicht zu protrahierten Leukozytopenien (d.h. Leukozytopenien, die > 3d andauern mit Leukozytenwerten < 1 x 109 /L) oder Leukozytopenie-bedingten Therapieverzögerungen kommt. In diesen Fällen erfolgt die G-CSF-Applikation (Lenograstim®) in den folgenden Zyklen ab Tag 4.

Patienten im Therapiearm B und BRX (R-CHOP-14) erhalten grundsätzlich G-CSF (Lenograstim®) ab Tag 4 bis Tag 13 bzw. bis zum Wiederanstieg der Leukozytenwerte > 2,5 x109/L.

G-CSF: Lenograstim®: 263 µg täglich einmal s.c. appliziert

5.7.2.5 Nebenwirkungen der eingesetzten Therapiemodalitäten

- **Cyclophosphamid:**
  Myelosuppression, Übelkeit/Erbrechen, Haarausfall, hämorrhagische Zystitis

- **Doxorubicin:**
  Myelosuppression, Übelkeit/Erbrechen, Haarausfall, Kardiomyopathie (max. Gesamtdosis: 550 mg/m2), Nekrosen bei paravasaler Injektion.

- **Vincristin:**
  periphere Polyneuropathie, paralytischer Ileus, Nekrosen bei paravasaler Injektion

- **Prednison:**
  Unruhe, Magenbeschwerden, Zunahme des Appetits, Osteoporose, Myopathie, steroidinduzierter Diabetes mellitus

- **Rituximab:**
  Allergische Reaktionen bis hin zum allergischen Schock. Vor allem bei 1. Applikation: Übelkeit, Brechreiz, Schluckbeschwerden, Kopfschmerzen, Müdigkeit. Unter und nach Rituximab-Therapie besteht eine etwas erhöhte Inzidenz von viralen Infektionen (HSV-Stomatitis, Herpes zoster)

- **G-CSF:**
  Knochenschmerzen, selten Temperaturerhöhung, erhöhte LDH

- **Strahlentherapie:**
  Übelkeit, Brechreiz, Schluckbeschwerden, Kopfschmerzen, Müdigkeit, Leukozytopenie, Thrombozytopenie, Anämie, Hautveränderungen und Haarausfall im Strahlengebiet. Möglich sind außerdem Strahlenreaktionen des Lungengewebes (Luftnot), des Darms (Durchfälle), des Herzbeutels (Erguss)

- **Intrathekale Therapie:**
  Kopfschmerzen, Übelkeit und Erbrechen unter prophylaktischer MTX-Gabe

5.7.2.6 Supportive Maßnahmen

- **selektive Darmdekontamination:**
  grundsätzlich **nicht erforderlich;** allerdings sollte bei einer Leukozytopenie < 1000/mm3 eine prophylaktische orale Antibiotika-Gabe mit 2 x 500 mg Ciprofloxazin (Ciprobay®) erfolgen.

- **Pneumocystis-carinii-Prophylaxe:**
  nicht erforderlich

- **Zystitis-Prophylaxe:**
  insbesondere am 1. Therapietag ausreichende Flüssigkeitszufuhr, ggf. durch Infusion unter kardiopulmonalem Monitoring. Uromitexan (Mesna®) – Prophylaxe entsprechend lokalen Standards.

- **Antiemese:**
  Metoclopramid (Paspertin®) oder Alizaprid (Vergentan®), je 1 Ampulle i.v., Stunde 0, 4, 8 können ausreichen; Serotoninantagonisten (z.B. Ondansetron®, Granisetron®) werden aber empfohlen.

- **Mundhygiene:**
  auf eine gründliche Mundhygiene ist zu achten; dies gilt insbesondere für Prothesenträger. Prophylaktische Mundspülungen mit Chlorheximid (Hexoral®) und Nystatin (Amphomoronal®) nach jeder Mahlzeit werden bei empfindlicher Schleimhaut empfohlen.

5.7.2.7 Kontrolluntersuchungen unter Therapie

- **durchgehend 2 x/Woche:**
  Blutbild (Leukozyten, Thrombozyten, Hb) mit Differentialblutbild (Hinweis: der Leukozytennadir wird an Tag 10-12 (CHOP-21), bzw. an Tag 8-10 (CHOP-14) erwartet [Erfahrung aus der NHL-B1- und DSHNHL 1999-2 Studie])

- **vor jedem Zyklus:**
  klinische Untersuchung (insbesondere: Lymphknoten-status, Ausschluss von Polyneuropathien, Mucositis), Blutbild (Leukozyten, Thrombozyten, Hb), LDH, GPT, AP, Bilirubin, Kreatinin, Elektrolyte

- **zusätzlich:**
  Echokardiographie nach 200 mg/m^2 Doxorubicin (Empfehlung der Ethikkommission der Ärztekammer Nordrhein)

5.7.3 Konsolidierende Strahlentherapie

5.7.3.1 Indikation zur konsolidierenden Strahlentherapie

Eine involved-field-Strahlentherapie wird bei allen Patienten mit Bestrahlungsindikation durchgeführt.
Mindestens eine bestrahlbare Lokalisation (Bulk, nicht operativ entfernter und/oder bestrahlbarer E-Befall) liefert eine Bestrahlungsindikation.

Die konsolidierende Bestrahlung wird in den folgenden Regionen vorgenommen:

1) Bulky Disease:

- Regionen mit initialem Bulky Disease (LK-Konglomerat oder Extranodalbefall ≥ 7,5 cm, siehe Anhang 14.10).
- Die Gesamtdosis beträgt 39,6 Gy, Einzeldosis 1,8 Gy, 5x/Woche

2) Extranodaler Befall:

Folgende extranodalen Lokalisationen erhalten grundsätzlich **keine** Bestrahlung:

- Knochenmarkbefall
- Lungenbefall (lokal oder diffus oder beides)
- Pleurabefall/Pleuraerguss (diffus, maligne)
- Perikardbefall (diffus)
- Nierenbefall (auch wenn nur eine Seite befallen)
- Dünndarmbefall (diffus)
- Dickdarmbefall (diffus)
- Leberbefall (lokal oder diffus oder beides)
- Milzbefall (lokal oder diffus oder beides)
- Peritonealbefall (Aszites)

Alle anderen extranodalen Lokalisationen mit qualifiziertem Extranodalbefall werden bestrahlt.

Die Gesamtdosis beträgt 39,6 Gy, Einzeldosis 1,8 Gy, 5x/Woche. Im Falle eines großen zu bestrahlenden Areals kann die tägliche Dosis auf 1,6 Gy reduziert werden.

3) Besonderheit bei Hodenbefall:

Patienten mit Hodenbefall erhalten eine prophylaktische Bestrahlung des kontralateralen Hodens und Skrotums mit einer Gesamtdosis von **30,6 Gy**, Einzeldosis 1,8 Gy, 5 x/Woche.

4) Besonderheit nach operativer Entfernung eines Bulks bzw. E-Befalls:

Eine Ausnahme bildet nur die vollständige operative Entfernung von Lymphombefall; hier wird auf die Bestrahlung verzichtet. Wenn zusätzlich noch nicht operativ entfernte bestrahlbare Lokalisationen (Bulk- oder E-Befall) vorliegen, werden diese bestrahlt.

5.7.3.2 Beginn der konsolidierenden Strahlentherapie

Die konsolidierende Strahlentherapie beginnt 2-6 Wochen nach Ende des letzten Immuno-Chemotherapiezyklus, wenn eine vollständige Erholung des Knochenmarks (Leukozyten > 3000/mm3, Thrombozyten > 100 000/mm3) eingetreten ist und evtl. aufgetretene Mucositiden vollständig abgeheilt sind.

5.7.3.3 Gerätetechnische Voraussetzungen für eine protokollgerechte konsolidierende Strahlentherapie

- Anwendung von Linearbeschleunigern. Eine Bestrahlung mit Co-60 ist sollte vermieden werden. Bei Verwendung von Photonen-Grenzenergien > 8 MeV müssen für oberflächliche Lymphommanifestationen ggf. geeignete Moulagen verwendet werden.
- 3-D- konforme Bestrahlungsplanung anhand von Planungs-Computertomographien
- Möglichkeit der Herstellung von individuellen Abschirmblöcken bzw. Einsatz von Multileaf- Kollimatoren
- Einsatz von individuellen Lagerungshilfen (Maskentechnik)
- Bestrahlungsvorbereitung und Dokumentation am Therapiesimulator
- Herstellung von Verifikationsaufnahmen / portal imaging

5.7.3.4 Bestrahlungsvolumina

Zur einheitlichen Definition des Bulky Disease und des Extranodalbefalls ist die Einsendung der prätherapeutischen Bildgebung zusammen mit dem Stagingbogen an die Studienzentrale obligat.
Anhand der prätherapeutischen Bildgebung und des Staging-Bogens wird von der Referenzstrahlentherapie ein individueller Behandlungsvorschlag erstellt und dem jeweiligen Strahlentherapeuten zugeschickt.

1) Konsolidierende Bestrahlung bei Bulky Disease:

Die Ausdehnung der Bestrahlungsvolumina umfasst nicht zwangsläufig die gesamte(n) befallene(n) Lymphknotenregion(en), sondern erfolgt als involved-field – Bestrahlung entsprechend der Lymphomausdehnung nach prätherapeutischer Bildgebung.
Das Planungszielvolumen umfasst die initiale Lymphomausdehnung mit einem Sicherheitssaum von 1,5 cm. Die Wahl des Zielvolumens erfolgt nach der initialen Bildgebung. Bei sicher verdrängendem Wachstum ohne Hinweise auf Infiltration ist (v. a. in den lateralen Feldbegrenzungen) eine Reduktion des Zielvolumens auf das postchemotherapeutische Volumen durchzuführen. Dies ist in der Bestrahlungsdokumentation festzuhalten.
Zur Anwendung kommen konformale 3D-Bestrahlungstechniken mit optimaler Schonung der initial nicht involvierten Normalgewebe.
Die Dosisspezifikation erfolgt nach ICRU 50.
Prinzipiell ist die gleichzeitige Bestrahlung aller Regionen mit Bulk- bzw. Extranodalbefall anzustreben. Bei Bulky Disease beidseits des Zwerchfells kann zunächst die Bestrahlung der Region mit der ursprünglich größeren Tumormasse erfolgen, sofern eine gleichzeitige Bestrahlung aller Regionen aufgrund zu großer Behandlungsvolumina nicht durchführbar erscheint.

2) Konsolidierende Bestrahlung bei qualifiziertem Extranodalbefall:

Eine besondere Bestrahlungstechnik kann aufgrund der Vielzahl der möglichen Befallslokalsationen und -muster nicht vorgegeben werden. Die Zielvolumendefinition erfolgt analog zur Behandlungsrichtlinie für Bulky Disease. Das klinische Zielvolumen entspricht der initialen Lymphomausdehnung mit einem Sicherheitsabstand von 1,5 cm. Zur Anwendung kommen konformale 3D-Bestrahlungstechniken mit optimaler Schonung nicht involvierter Normalgewebe.

In Zweifelsfällen bitte Rücksprache mit der Referenzstrahlentherapie in Homburg

Dr. Berdel, Dr. Fleckenstein, Prof. Dr. Rübe

Tel. 06841/16-24606
E-Mail: radonk@uks.eu

5.7.3.5 Dokumentation der Strahlentherapie

Nach Beendigung der konsolidierenden Strahlentherapie werden der ausgefüllte Strahlentherapiebogen (RX-Bogen), Bestrahlungsplan, Simulationsaufnahmen, Verifikationsaufnahmen sowie die im Abschlussstaging nach Chemotherapie angefertigten Computertomogramme an die Studienzentrale geschickt. Diese Unterlagen werden einer Beurteilung durch das Panel der Referenz-Strahlentherapeuten unterzogen und der behandelnden Klinik zeitnah zurückgesandt.

5.8 Restaging- und Nachsorgeuntersuchungen

5.8.1 Zwischenstaging

2 Wochen nach Beginn des 3. Zyklus (unter Berücksichtigung der zeitgerechten Therapiefortsetzung):

- Anamnese
- Klinische Untersuchung (Lymphknotenregionen!)
- Kontrolle der Laborparameter wie bei Staginguntersuchungen (s. 5.1)
- EKG
- CT-Thorax/Abdomen (falls primär befallen)
- Beurteilung und Dokumentation des Therapieerfolgs (s. 5.9) und der Nebenwirkungen (s. 6.)

5.8.2 Abschlussstaging nach Chemotherapie

14 Tage nach Beginn des 6. Zyklus erneute Durchführung aller Untersuchungen wie in den Staginguntersuchungen:

- Anamnese
- Klinische Untersuchung (Lymphknotenregionen!)
- Kontrolle der Laborparameter wie bei Staginguntersuchungen (s. 5.1)
- EKG
- CT-Thorax/Abdomen (falls primär befallen)
- Adäquate Kontrolle aller anderen Primärmanifestationen (z.B. KM-Biopsie)
- Beurteilung und Dokumentation des Therapieerfolgs (s.5.9) und der Nebenwirkungen (s. 6.)

Dieses Staging wird für alle Patienten aller Arme als definitives Abschlussstaging gewertet.

5.8.3 Remissionsbestätigung

Bei Patienten ohne Bestrahlungsindikation muss der Remissionsstatus durch eine erneute CT-Kontrolle der Restbefunde bestätigt werden, falls im Abschlussstaging nach der Immuno-Chemotherapie aufgrund der dort nachweisbaren Restbefunde eine CRu/PR, vorliegt. Diese zusätzliche Kontrolle dient der zweifelsfreien Festlegung des Therapieerfolges und dem daraus resultierenden entsprechenden weiteren Vorgehen.

Patienten, die aufgrund dieser Kontrolle der Restbefunde die Kriterien einer CR/CRu erfüllen, erhalten eine protokollgemäße Nachsorge. Alle anderen Patienten werden einer Salvagetherapie zugeführt (hinsichtlich der Besonderheiten einer PR verweisen wir auf Abschnitt 5.9.3).

Die Remissionsbestätigung entfällt bei Patienten mit Bestrahlungsindikation.

5.8.4 Zusatzstaging nach Strahlentherapie durch den Strahlentherapeuten (nicht obligat)

Da das erste Follow-up in allen Armen bereits 3 Monate nach dem Abschlussstaging nach Chemotherapie durch den Internisten stattfindet, ist ein Zusatzstaging nach Strahlentherapie nicht vorgesehen. Das Zusatzstaging kann fakultativ durch den Strahlentherapeuten durchgeführt werden.

5.8.5 Restaging bei vorzeitiger Beendigung der Therapie

Bei vorzeitiger Beendigung der Therapie (z.B. auf Wunsch des Patienten oder wegen exzessiver Toxizität) erfolgt zur Feststellung des Therapieerfolges zum Zeitpunkt des Abbruches **möglichst umgehend** ein Restaging, das in seinem Umfang dem definitiven Abschlussstaging nach Chemotherapie (s. 5.8.2) entspricht.

5.8.6 Nachsorgeuntersuchungen

Alle Patienten werden bis zum Abschluss der Studiendurchführung und geplanten Nachbeobachtungszeit, d.h. mindestens bis Juni 2017 nachuntersucht. **Die erste Nachuntersuchung erfolgt in allen Armen 3 Monate nach dem Abschlusstaging nach Chemotherapie**. Die anschließenden Nachsorgeuntersuchungen erfolgen in den ersten beiden Jahren ebenfalls alle 3 Monate, vom dritten bis zum fünften Jahr alle 6 Monate und anschließend jährlich. Klinische Untersuchung, Laboruntersuchungen, bildgebende Diagnostik sowie Erhebung des Remissionsstatus und von therapiebedingten Folgeerkrankungen und Sekundärneoplasien werden wie im Therapieplan (s. 0.7) detailliert angegeben durchgeführt und auf den Dokumentationsbögen für die Nachsorge dokumentiert.

5.9 Erhebung der Zielkriterien

Der Therapieerfolg wird aufgrund der Ergebnisse der definitiven Restaginguntersuchungen beurteilt, sobald diese vorliegen. Die folgenden Remissionskriterien sind für das Restaging nach Abschluss einer Therapie verbindlich und sinngemäß auch für ein Zwischenstaging bzw. eine Remissionsbestätigung und während der Nachsorge anzuwenden. Zur Beurteilung der Remissionskriterien werden die kürzlich publizierten Empfehlungen des „International Workshop to Standardize Response Criteria for Non-Hodgkin´s Lymphomas“[39] angewendet und entsprechend der gebotenen Praktikabilität im Rahmen einer großen multizentrischen Studie für aggressive Lymphome modifiziert.

Anmerkung:
Bei PR (siehe 5.9.3!), NC und PRO besteht in der Regel Therapiebedarf!

Hinweis:

- Patienten mit CR/CRu, PR, NC im Zwischenstaging nach 3 Zyklen Chemotherapie erhalten 3 weitere Zyklen R-CHOP-21 (Arme A/ARX) bzw. R-CHOP-14 (Arme B/BRX).
- Patienten mit initialem BULK- und/oder Extranodal-Befall erhalten eine konsolidierende Bestrahlung mit 39.6 Gy auf diese Lokalisationen
- Patienten mit initialem Hodenbefall erhalten eine prophylaktische Bestrahlung des kontralateralen Hodens und Skrotums mit 30,6 Gy
- Patienten ohne Bestrahlungsindikation ohne CR/CRu in der Remissionsbestätigung 4 Wochen nach dem Abschlussstaging nach Chemotherapie werden einer Salvage-Therapie zugeführt.
- Patienten ohne Response (PRO) werden einer Salvage-Therapie zugeführt.

5.9.1 Komplette Remission (CR)

Verschwinden aller Krankheitszeichen (klinisch, radiologisch und labordiagnostisch [LDH]). In diesem Fall wird das Therapieergebnis als "CR mit vollständiger Rückbildung" (kurz: CR) bezeichnet. Andere aufgrund Lymphombefalls vergrößerte Organe (Milz, Leber, Niere) müssen in ihrer Größe zurückgegangen sein und dürfen keine Raumforderungen mehr aufweisen. Bei vorausgegangenem Knochenmarkbefall muss, durch eine Knochenmarkbiopsie abgesichert, das Knochenmark *lichtmikroskopisch* lymphomfrei sein. Das Blutbild muss sich normalisiert haben mit Granulozyten > 1500/µl, Hb > 12 g/dl und Thrombozyten > 100000/µl. **Nach Therapieende muss der Befund einer CR vom Zeitpunkt des Abschlussstagings nach Chemotherapie in allen Armen für mindestens 2 Monate vorliegen.**

5.9.2 Komplette Remission mit verbleibender Unsicherheit (CRu)

Sind alle Kriterien für eine CR erfüllt, aber in den bildgebenden Verfahren noch residuelle Veränderungen vorhanden, so ist das Therapieergebnis als "CR mit verbleibender Unsicherheit" (kurz CRu) zu bezeichnen. Bei Vorhandensein von persistierenden Lymphomzellen in einer Re-Biopsie kann der Therapieerfolg nicht als CRu gewertet werden. Analog zur CR muss nach Therapieende der Befund einer CRu, gemessen vom Zeitpunkt des Abschlussstagings nach Chemotherapie in allen Armen, für mindestens 2 Monate vorliegen. **Eine CRu am Ende der Therapie bedeutet, dass der behandelnde Arzt zum Zeitpunkt der Beurteilung keinen weiteren Therapiebedarf sieht**.

5.9.3 Partielle Remission (PR)

Eine partielle Remission liegt vor, wenn die folgenden Kriterien erfüllt sind:

- Weiterhin Vorliegen von Lymphomgewebe (im Zweifelsfall histologisch gesichert),
- jedoch deutliche Reduktion in allen Befallslokalisationen und Reduktion des Gesamtvolumens um mindestens 50%.
- Keine neuen Lymphommanifestationen
- Normalisierung des Blutbildes

Anmerkung:

1) In der Regel geht eine PR mit einem Tumorzell-Kill von mehreren Größenordnungen einher. Der Definition einer PR liegt die Vorstellung von der grundsätzlichen Kurabilität der Erkrankung zugrunde. Die Festlegung des behandelnden Arztes auf eine PR impliziert, dass er (falls keine Kontraindikationen bestehen) über die protokollgerechte Therapie hinausgehende therapeutische Maßnahmen (z. B. Salvage-Therapie) für indiziert hält. **Eine operationale Definition von PR beinhaltet einerseits, dass der behandelnde Arzt weitergehende Therapiebedürftigkeit konstatiert; sie beinhaltet angesichts der Wachstumsdynamik aggressiver Lymphome andererseits, dass man davon ausgehen kann, dass bei einer vermeintlichen CRu bei Persistenz von aktivem Tumorgewebe sich innerhalb von 2 Monaten wieder Tumorwachstum (Größenzunahme des Restbefundes) zeigen sollte, und sich somit eine vermeintliche CRu/PR als PRO demaskieren würde.** Dies wird bei der Auswertbarkeitserklärung und endgültigen Festlegung des Therapieerfolgs durch die Studienleitung berücksichtigt (s. 7.5.1) **Bei Unsicherheiten oder Unklarheiten, insbesondere bei der Abgrenzung einer CRu von einer PR empfehlen wir Rücksprache mit der Studienleitung.**

2) Die obige Definition geht von der Vorstellung aus, dass die Kinetik der Rückbildung großer umschriebener Läsionen einen Indikator für die Rückbildung aller Läsionen (d.h. auch kleiner umschriebener Läsionen oder diffusen Befalls) darstellt. Somit wird auf die Ausmessung aller Einzelbefälle verzichtet. Eine Ausnahme stellen Knochenbefälle dar, da dort keine vollständige Rückbildung in den bildgebenden Nachuntersuchungen zu erwarten ist.

5.9.4 No Change (NC)

Weiterhin Vorliegen von Lymphommanifestationen mit nur geringer Größenabnahme bzw. geringer Größenzunahme der Lymphome oder Organbefälle (Ausschluss von PRO und PR). Ein NC liegt vor, wenn gilt:

- kein Lymphom hat um mehr als 25% im größten Durchmesser zugenommen;
- die Erkrankungsrückbildung erfüllt nicht die Kriterien für eine PR (d.h. Rückbildung < 50%)

5.9.5 Progress (PRO)

Ein Progress der Erkrankung liegt vor bei:

- Wiederauftreten von Krankheitssymptomen
- Auftreten neuer lymphatischer oder extralymphatischer Läsionen
- deutliche Zunahme der Lymphommanifestationen um mehr als 25% im Vergleich zum Initialbefund

5.9.6 Rezidiv

Ein Rezidiv liegt vor, wenn nach einer mindestens 2 Monate andauernden CR oder CRu (gerechnet vom Zeitpunkt des Abschlussstaging) eines oder mehrere der folgenden Kriterien erfüllt sind:

- Wiederauftreten von Krankheitssymptomen
- Auftreten neuer lymphatischer oder extralymphatischer Läsionen
- deutliche Zunahme der Lymphommanifestationen um mehr als 25%

Bei kürzerem Intervall wird der Fall als Progress gewertet. **Bei Rezidiven ist immer eine erneute histologische Sicherung anzustreben; die Erfahrungen aus der ersten und zweiten Studiengenerationen der DSHNHL zeigen, dass sich dadurch in vielen Fällen unerwartete therapeutische Konsequenzen ergeben können!**

5.9.7 Beurteilung von nicht messbarem Tumor oder Knochenbefall

Bei nicht messbarem Tumor oder Knochenbefall bedeutet CR das Verschwinden aller Krankheitszeichen über mindestens 2 Monate (gerechnet vom Zeitpunkt des jeweiligen Abschlussstagings); CRu bedeutet deutlicher Rückgang aller Krankheitszeichen ohne Hinweis auf verbleibende Aktivität über mindestens 2 Monate (gerechnet vom Zeitpunkt des Abschlussstaging), Rückgang aller Krankheitszeichen, aber mit Hinweis auf Aktivität oder Größenzunahme innerhalb von 2 Monaten wird als Progress gewertet. **In Zweifelsfällen empfehlen wir grundsätzlich Rücksprache mit der Studienleitung**.

6 Feststellung der Sicherheit/Unerwünschte Ereignisse

6.1 Feststellung der Sicherheit

Zum Staging werden alle für die Therapiedurchführung wichtigen Laborparameter (s. 5.1) erhoben. Diese werden zum Zwischenstaging nach 3 Zyklen (s. 5.8.1), zum Abschlussstaging und nach Chemotherapie (s. 5.8.2) und in der Remissionsbestätigung (s.5.8.3) überprüft.
Zu Beginn der Chemotherapiezyklen und mindestens 2mal während des Zyklus, insbesondere im Nadirbereich, werden Blutbildkontrollen durchgeführt. Der Allgemeinzustand des Patienten (ECOG) wird vor Therapie erhoben und zu jedem Therapieabschnitt überprüft.

6.2 Unerwünschtes Ereignis (UE)

6.2.1 Definition erwartetes/unerwartetes Unerwünschtes Ereignis

Ein Unerwünschtes Ereignis ist jede nachteilige Veränderung des Gesundheitszustandes während und/oder nach der Therapie, verglichen mit dem Gesundheitszustand des Patienten vor Therapiebeginn, unabhängig davon, ob diese Veränderung therapiebezogen ist. Die Unerwünschten Ereignisse werden entsprechend den NCI Common Toxicity Criteria (CTC) (s. Anhang 14.14, deutsche Fassung der Deutschen Krebsgesellschaft) beurteilt.
Unerwünschte Ereignisse, welche nicht explizit in der Liste der CTC-Kriterien aufgeführt sind, werden unter „Sonstiges“ angegeben und in Analogie nach folgendem 4-Punkte-System beurteilt.

Grad 0	„Keine“
Grad 1	„Gering“/„Leicht“
Grad 2	„Mäßig“/„Deutlich“
Grad 3	„Stark“/„Ausgeprägt“
Grad 4	„Lebensbedrohlich“

Die folgenden in Zusammenhang mit der Therapie stehenden Ereignisse sind unerwünscht, aber erwartet:

- Myelosuppression
- Übelkeit/Erbrechen
- Haarausfall
- Infektionen, insbesondere während leukozytopenischer Phasen
- periphere Polyneuropathie
- Strahlenschäden der Lunge, des Herzbeutel oder des Darms

Darüber hinaus können auch unerwartete unerwünschte Ereignisse auftreten. Alle erwarteten und unerwarteten unerwünschten Ereignisse müssen sorgfältig dokumentiert werden (s. 6.2.2).

6.2.2 Erfassung der erwarteten/unerwarteten Unerwünschten Ereignisse

Der Grad der aus der Liste der CTC-Kriterien ausgewählten Unerwünschten Ereignisse soll auf allen Chemotherapie- und dem Radiotherapiebogen in den dafür vorgegebenen Feldern dokumentiert werden. Treten darüber hinaus Nebenwirkungen auf, welche auf den Dokumentationsbögen nicht explizit abgefragt werden, soll die entsprechende CTC-Nummer des Unerwünschten Ereignisses sowie der Grad dem Protokollanhang 14.14 entnommen werden und auf den Dokumentationsbögen unter Angabe der entsprechenden CTC – Nummer dokumentiert werden. Unerwünschte Ereignisse, welche nicht in den CTC-Kriterien aufgelistet sind, werden als Klartext angegeben und der Grad wird in Analogie zu den CTC-Kriterien entsprechend der Definition (s. 6.2.1) vergeben.
Nach Abschluss der Therapie werden auftretende Komplikationen auf dem Folgebogen nach einem Diagnoseschlüssel für Komplikationen (siehe Anhang 14.14) abgefragt.
Die interkurrenten Erkrankungen, welche sich nicht nach CTC verschlüsseln lassen, werden als Klartext angegeben.

6.3 Schwerwiegendes Unerwünschtes Ereignis (SUE)

6.3.1 Definition Schwerwiegendes Unerwünschtes Ereignis

Unerwünschte Ereignisse erhalten den Zusatz schwerwiegend, wenn es sich um Ereignisse handelt, welche für den Patienten eine besondere „Gefährdung“ darstellen.
Als SUE werden die folgenden Ereignisse gewertet:

- langdauernde (d.h. über 3 Monate über das Therapieende hinausreichende) transfusionsbedürftige Anämien und Thrombozytopenien
- lebensbedrohliche Infektion
- therapiebedingter Todesfall
- schwere Kardiomyopathie (NYHA Stadium III/IV)
- therapieinduzierte Myelodysplasien
- therapieinduzierte Sekundärneoplasie (insbesondere Leukämie)
- ungeplante stationäre Aufnahme aus medizinischen Gründen (Notfall)

Bei Ereignissen, welche darüber hinausgehen und von den behandelnden Ärzten als schwerwiegend eingeschätzt werden, sollte eine Rücksprache mit der Studienleitung erfolgen.
Ereignisse, welche ausschließlich in Zusammenhang mit der Tumorprogression stehen, werden nicht als SUE gewertet. Die SUEs unterliegen einer Meldepflicht (s. 6.3.2).

6.3.2 Erfassung der Schwerwiegenden Unerwünschten Ereignisse

Alle Schwerwiegenden Unerwünschten Ereignisse müssen auf dem SUE-Meldebogen dokumentiert werden und innerhalb eines Arbeitstages während der Therapie und innerhalb von 10 Tagen im Follow-up an die Studienleitung gefaxt werden.
Sollten SUEs in einem der beiden Therapiearme oder im Vergleich zur NHL-B-Studie gehäuft auftreten, so wird der vorzeitige Abbruch der Studie erwogen (s. 3.4.2).

7 Biometrische Aspekte der Studie

7.1 Randomisationsalgorithmus

Alle Patienten, bei denen keine Ausschlusskriterien vorliegen und die nach Abschluss der Staging-Untersuchungen alle Einschlusskriterien erfüllen, können randomisiert werden. Die Randomisation erfolgt mittels einer Minimierungsmethode[43]. Die Realisierung des Randomisationsalgorithmus erfolgt innerhalb der ORACLE-Datenbank. Die Minimierungsmethode ermöglicht eine Balancierung von Therapiearmen und Strata. Es wird für die Patienten mit Indikation für die konsolidierende Radiotherapie (initialer Bulkbefall (nicht operativ entfernt), bestrahlbarem E-Befall oder beidem) in die 4 Therapiearme R-CHOP-21 + RX, R-CHOP-21 – RX, R-CHOP-14 + RX und R-CHOP-14 – RX im Verhältnis 1:1:1:1 randomisiert.
Bei der letzten Zwischenauswertung vom 1.7.2012 war das formale Abbruchkriterium für den RX-Kontrast erfüllt. Die Therapiearme mit RX sind signifikant besser. Das DMSC hat die Schließung der beiden Therapiearme ohne RX beschlossen und die Randomisation wurde daher am 13. Juli.2012 für diese beiden Therapiearme gesperrt.
Für die Patienten ohne Indikation für die konsolidierende Strahlentherapie wird in die beiden Therapiearme R-CHOP-21 - RX und R-CHOP-14 - RX im Verhältnis 1:1 randomisiert. Es wird für beide Randomisationen nach folgenden Kriterien stratifiziert:

- Zentrum
- Wert der Serum-LDH (LDH≤ ONW vs. LDH > ONW)
- Stadium (I, II vs. III, IV)
- Allgemeinzustand des Patienten (ECOG = 0,1 vs. ECOG = 2,3)
- Bulkbefall
- Extranodaler Befall

Zusätzlich zum Zentrum wird also nach den prognostischen Faktoren des altersadjustierten Internationalen Prognostischen Index (LDH, Stadium, ECOG) stratifiziert.
Das Vorliegen von Bulkbefall und extranodalem Befall sind zwei weitere Stratifikationskriterien. Das Stratifizieren nach diesen Merkmalen ermöglicht die separate Auswertung der konsolidierenden Strahlentherapie sowohl für die Patienten mit Bulk als auch für die Patienten mit E-Befall. Darüberhinaus werden bei der 2-armigen Randomisation die operativ entfernten Bulkbefälle und die aus technischen Gründen nicht bestrahlbaren E-Befälle balanziert.
Patienten, bei denen nach Aufnahme in die Studie festgestellt wird, dass die Aufnahmekriterien zum Zeitpunkt der Randomisation nicht erfüllt waren, obwohl zum Zeitpunkt der Randomisation als erfüllt angenommen, werden nicht nachträglich gestrichen. Es werden nur Patienten gestrichen, wenn diese nachträglich ihr schriftliches Einverständnis zurückziehen. Bei Streichung dieser Patienten erfolgt eine Korrektur der Bilanzen im Randomisationsprogramm.

7.2 Endpunkte der Studie

7.2.1 Primärer Endpunkt – Hauptzielkriterium

Das Hauptzielkriterium der Studie ist die Zeit bis zum Therapieversagen (TTF = Time To Treatment Failure). Zur Schätzung des TTF wird die Methode von Kaplan und Meier verwendet. Die Zeit bis zum Therapieversagen ist die Zeit vom Randomisationsdatum (oder im Falle einer Vorphase-Therapie vor der Randomisation, der erste Tag der Vorphase) bis eines der folgenden Ereignisse eintritt:

- Progress unter Therapie (PRO)
- Abbruch der Therapie wegen exzessiver Toxizität und keine CR/CRu zu diesem Zeitpunkt
- keine CR/CRu oder PRnach Therapieabschluss
- Rezidiv nach erreichter CR/CRu
- Wechsel auf eine Salvagetherapie / zusätzliche nicht im Protokoll geplante Therapie
- Tod jeglicher Ursache

Das zuerst eintretende Ereignis zählt. Bei Patienten ohne Ereignis zählt die Zeit bis zur letzten vorliegenden Information. Die Bewertung von Protokollverstößen, Abbruch auf Wunsch des Patienten, Abbruch auf Wunsch des Arztes oder Sonstiges muss für jeden Einzelfall gesondert im Rahmen der Auswertbarkeitserklärungen (s. 7.5.1) erfolgen.

7.2.2 Sekundäre Endpunkte – Nebenzielkriterien

7.2.2.1 Nebenzielkriterien bezüglich Wirksamkeit

Nebenzielkriterien der Studie bezüglich Wirksamkeit sind die CR-Rate, die Progress-Rate, das Überleben, die Tumorkontrolle und das rezidivfreie sowie krankheitsfreie Überleben. Die Tumorkontrolle ermöglicht eine Einschätzung der biologischen Wirksamkeit unter Verringerung des Einflusses von Toxizität. Die Abschätzung des krankheitsfreien Überlebens gestattet den Vergleich der Zeitstruktur des Auftretens der Rezidive.

- **CR-Rate:**
  Zahl der aufgetretenen kompletten Remissionen (Patienten mit vorzeitigen Therapieabbrüchen eingeschlossen) geteilt durch die Anzahl der Patienten

- **Progress-Rate:**
  Zahl der aufgetretenen Progresse geteilt durch die Anzahl der Patienten

- **Überleben:**
  Zeit vom Randomisationsdatum (oder im Falle einer Vorphase-Therapie vor der Randomisation, der erste Tag der Vorphase) bis zum Tod jeglicher Ursache, bei lebenden Patienten Zeit bis zur letzten vorliegenden Information

- **Tumorkontrolle:**
  analog TTF, aber Ereignisse, die nicht tumorbedingt sind, werden zensiert

- **Krankheitsfreies Überleben:**

analog TTF, aber Ereignisse unter Therapie und unmittelbar am Ende der Therapie werden auf den Zeitpunkt ε = 0.01 Monat gesetzt

- **Rezidivfreies Überleben:**
  analog TTF, aber nur für Patienten mit CR/CRu nach einer kompletten Immuno-Chemotherapie

7.2.2.2 Nebenzielkriterien bezüglich Sicherheit

Nebenzielkriterien bezüglich Sicherheit sind:

- die Unerwünschten Ereignisse (UE) (s. 6.2)
- die Schwerwiegenden Unerwünschten Ereignisse (SUE) (s. 6.3)
- ausgewählte Laborparameter (s. 5.1, 5.7)
- Rate der Sekundärneoplasien

7.2.2.3 Nebenzielkriterien zur Gesundheitsökonomie

Zur Abschätzung der Unterschiede der direkten Kosten zwischen den Therapiearmen werden folgende gesundheitsökonomische Parameter erhoben:

- Gesamtdosis der Zytostatika
- Gesamtdosis von Rituximab
- Gesamtdosis G-CSF für jeden Patienten
- Tage im Krankenhaus
- Gesamttage der Antibiotikagabe
- Gesamtzahl der Erythrozyten- und Thrombozytenkonzentrate
- Therapiemaßnahmen zur Behandlung aufgetretener SUEs

7.2.2.4 Nebenzielkriterien zur Protokolladhärenz

- Zyklusdauer
- Gesamtdosis und Dosisintensität
- Gesamtdosis und Dauer der G-CSF-Gabe

7.2.2.5 Nebenzielkriterien zur konsolidierenden Strahlentherapie

Rezidivprofil von bestrahlten bzw. nicht bestrahlten Patienten mit Bulk und/oder qualifiziertem Extranodal-Befall getrennt nach Rezidiven im Bestrahlungsfeld und außerhalb des Bestrahlungsfeldes bzw. des Primärbefalls

7.3 Statistische Formulierung der Studienfrage und Fallzahlberechnung

In der vorliegenden Studie sollen die folgenden Fragen geprüft werden:

1) Kann die Wirksamkeit einer Immuno-Chemotherapie mit 6 Zyklen Rituximab in Kombination mit CHOP (Cyclophosphamid, Doxorubicin Vincristin und Prednison) verbessert werden, wenn die Therapieintervalle von 3 auf 2 Wochen reduziert werden (6 x R-CHOP-21 vs. 6 x R-CHOP-14)?

2) Führt die konsolidierende Bestrahlung von Bulk- im Anschluss an die Immuno-Chemotherapie zu einer Verbesserung der Therapieergebnisse?

Da nicht auszuschließen ist, dass die Erhöhung der Dosisintensität bzw. die Durchführung der Bulkbestrahlung mit höherer Toxizität einhergeht und diese den Therapieerfolg kompromittieren könnte, sollen die zwei Fragestellungen jeweils mit einem zweiseitigen Test beantwortet werden. Zur Beantwortung der Fragestellungen werden folgende statistische Hypothesen getestet:

zu 1) H_0: TTF (R-CHOP-21) = TTF (R-CHOP-14)
H_A: TTF (R-CHOP-21) ≠ TTF (R-CHOP-14)
zu 2) H_0: TTF (R-CHOP-21/14 - RX) = TTF (R-CHOP-21/14 + RX)
H_A: TTF (R-CHOP-21/14 - RX) ≠ TTF (R-CHOP-21/14 + RX)

Der aufzudeckende Unterschied wird sowohl aus dem Therapieversagen unter Therapie, als auch aus dem Rezidivverhalten resultieren. Aufgrund von Beobachtungen der Hazard-Raten in anderen Studiengruppen, von Schätzungen der Tumorlatenzzeiten und von theoretischen Modellabschätzungen, sollten sich die Unterschiede zwischen den Therapiearmen bezüglich der TTF-Rate bis zu einem Zeitraum von 3 Jahren aufbauen.
Aus der im Mai 2004 durchgeführten Zwischenauswertung der MInT-Studie wissen wir, dass die 3-Jahres TTF-Rate in dem – in diesem Protokoll definierten – Kollektivn der ≤ 60 Jährigen im Arm R-CHOP-21 bei 71% liegt. R-CHOP-21 ohne RX liegt tiefer, falls die Bestrahlung etwas bringt. Allerdings liegt der Kontrast aller Arme ohne RX wiederum etwas höher, falls R-CHOP-14 etwas bringt. Die Annahme von 71% zur Planung der Fallzahl scheint daher plausibel zu sein. Der Nachweis einer Verbesserung der Zeit bis zum Therapieversagen um 10% kann aufgrund der Vorerfahrungen als ein relevanter Fortschritt angesehen werden.
Im Rahmen dieser Studie soll eine Verbesserung der TTF-Rate von 71% auf 81% (d.h. ein konstantes Hazard Ratio von 0.615) gezeigt werden. Die zweiseitige Fragestellung soll mit einer Irrtumswahrscheinlichkeit von α = 5% und einer Power von 80% beantwortet werden.
Für die Fallzahlkalkulation wird ein Verfahren von Freedman verwendet. Die Berechnung erfolgte mit der Software nQuery Advisor, Version 2.0. Die Berechnungen ergaben eine für den Test erforderliche Fallzahl von 578 informativen Patienten mit Bulk, d.h. 289 Patienten pro Kontrast (ohne RX, mit RX). Die Fallzahlkalkulation unterstellt, dass bis zu 3 Jahren keine Dropouts auftreten. Sollten Patienten am Ende der Studie wegen fehlender Dokumentation nicht auswertbar sein, würde die Power dementsprechend geringer sein (s. Abbildung).

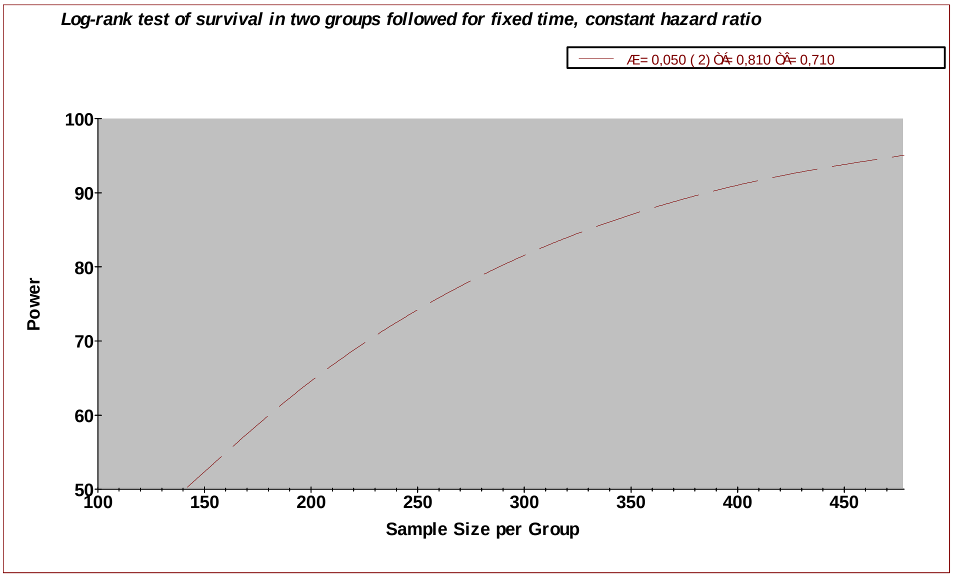

Da wir aus der High/CHOEP Studie wissen, dass der Anteil der Patienten mit Bulkbefall in dem hier zu untersuchenden Kollektiv bei ca. 56% liegt und bei der MInT-Studie bei 68% (Stadium I, non bulk war ausgeschlossen, daher eher zu hoch) sollten ca. 60% mehr Patienten randomisiert werden, um die Strahlentherapiefrage in Bezug auf Bulky-Disease mit ausreichender Power beantworten zu können. Damit müssen 964 Patienten in die Studie eingebracht werden. Um neben der Intention-to-Treat Analyse auch die ‚per protocol' Analyse (Patienten mit erfüllten Aufnahmekriterien und vorhandener referenzpathologischer Beurteilung) mit ausreichender Power durchführen zu können, sollen 10% mehr Patienten randomisiert werden. Das entspricht einer **Gesamtfallzahl von 1072** Patienten.
Da für die Beantwortung der Immuno-Chemotherapiefragestellung für die ‚per protocol' Analyse das gesamte Kollektiv der 964 Patienten zur Verfügung steht, wird zur Beantwortung dieser Fragestellung eine Power von 95% erreicht. Damit ist auch die Möglichkeit einer alpha-Adjustierung zur Berücksichtigung des multiplen Testens für die zweite Fragestellung gegeben.

Die Fallzahlkalkulation orientierte sich an den vierarmig randomisierten Patienten mit Indikation für die konsolidierende Radiotherapie (initialer Bulkbefall (nicht operativ entfernt), bestrahlbarem E-Befall oder beidem). Für dieses Kollektiv sollten im 2x2 faktoriellen Design die zwei Studienfragen zum Stellenwert der RX(+/-) und zu dem Stellenwert der Länge der Zyklusintervalle (14/21) beantwortet werden. Das formale Abbruchkriterium für den RX-Kontrast war in der letzten Zwischenauswertung erfüllt. Die beiden Therapiearme mit RX (6xR-CHOP-21+RX, 6xR-CHOP-14+RX) waren signifikant besser im Vergleich zu den beiden Therapiearmen ohne RX (6xR-CHOP-21-RX, 6xR-CHOP-14-RX). Wenn im 2x2 faktoriellen Design ein Kontrast vorzeitig geschlossen wird, dann werden die beiden übrigen Therapiearme weiter fortgeführt und an der Fallzahlkalkulation ändert sich nichts, es sei denn die damals getroffenen Annahmen zu dem Ausgangsniveau (3-Jahres EFS) sind nicht mehr zutreffend. Anhand der bei dieser Zwischenauswertung vorliegenden Daten wurde diese Annahme überprüft und festgestellt, dass diese weiterhin zutreffend ist.

7.4 Zwischenauswertungen und Abbruchkriterien

Die auftretenden Ereignisse unterliegen einem ständigen Monitoring durch die Studienärzte und Biometriker. Die Studienleitung entscheidet über den vorzeitigen Abbruch der Studie, wenn eines der in Abschnitt 3.4 angegebenen Kriterien erfüllt ist. Ein formales Abbruchkriterium wird über eine sogenannte „alpha spending function"[44] definiert. Diese erlaubt im Vergleich zu den traditionellen Gruppensequentialplänen für den Zeitpunkt der Zwischenauswertung zu adjustieren. Zur Berechnung der „stopping boundary" wird die von O' Brien-Fleming vorgeschlagene Methode verwendet[45]. Diese Methode hält nahezu die konventionellen p-Werte für die Abschlussauswertung ein, macht es aber schwierig, die Studie ungerechtfertigt zu früh zu beenden.
[Bei insgesamt 133 erwarteten Ereignissen würde man zum Beispiel nach $\tau_1 = 25$, $\tau_2 = 50$, $\tau_3 = 75$, $\tau_4 = 100$, $\tau_5 = 125$ Ereignissen folgende O'Brien-Fleming Boundaries erhalten:
$\alpha(\tau_1) < 0.0000061$, $\alpha(\tau_2) = 0.0013903$, $\alpha(\tau_3) = 0.0090525$, $\alpha(\tau_4) = 0.0237975$, $\alpha(\tau_5) = 0.0432024$]
Der vorzeitige Abbruch einer Studie erfordert eine komplexe Betrachtung verschiedenster Faktoren, daher kann das hier vorgeschlagene Abbruchkriterium nur den Entscheidungsprozeß über die vorzeitige Beendigung der Studie in Gang setzen.
Eine konklusive Zwischenauswertung zur Einschätzung der Wirksamkeit ist in den ersten 2 Jahren nicht zu erwarten. Daher soll die erste planmäßige Zwischenauswertung bezüglich Wirksamkeit im 3. Jahr nach Rekrutierungsbeginn mit ca. 40% der erwarteten Ereignisse stattfinden (voraussichtlich im Jahre 2008). Da zum Zeitpunkt der ersten formalen

Zwischenauswertung zunächst 650 Patienten (ca. 60% des Gesamtkollektivs) rekrutiert sind, ist auch im 4. Jahr nach Rekrutierungsbeginn eine weitere Zwischenauswertung zur Wirksamkeit während der Rekrutierungsperiode fest geplant. Da die hier vorgeschlagene „alpha spending function“ auch flexibel ist bezüglich der Anzahl der Zwischenauswertungen, kann die Studienleitung über Anzahl und Zeitpunkt weiterer Zwischenauswertungen im Verlauf der Studie entscheiden. Lediglich zur Überprüfung der Durchführbarkeit und Protokolladhärenz sollen in den ersten drei Jahren jährlich Zwischenauswertungen durchgeführt werden.
Eine Abschlussauswertung kann stattfinden, wenn die geplante Zahl der Ereignisse erreicht ist und die Dokumentation ausreichend ist. Zu jeder Auswertung wird durch den verantwortlichen Biometriker ein Bericht erstellt. Die Ergebnisse der Abschlussauswertung werden in einem Abschlußbericht dargestellt. Dieser beinhaltet eine Beschreibung des Patientenkollektivs getrennt nach Therapiearm, die Beschreibung der Durchführbarkeit der Therapie, sowie der Sicherheit der Therapie, insbesondere des Auftretens Unerwünschter und Schwerwiegender Unerwünschter Ereignisse, der Protokolleinhaltung und der vorzeitigen Therapieabbrüche. Es werden die Ergebnisse der Analyse zur Wirksamkeit und Sicherheit dargestellt. Die Ergebnisse der Auswertungen werden auf regelmäßig stattfindenden Studientreffen präsentiert.

7.5 Geplante Analysemethoden

7.5.1 Definition des Auswertungskollektivs

Vor jeder Auswertung werden die Daten jedes Studienpatienten durch ein Review-Panel bestehend aus Studienarzt, Biometriker und Dokumentar evaluiert. Es gelten die folgenden Evaluationskriterien:

- Erfüllung der Aufnahmekriterien;
- Bestätigung der Primärdiagnose (Referenzpathologie);
- adäquate Zuordnung und Randomisation;
- vollständige Dokumentation der Therapie;
- Beobachtungsdauer mindestens 2 Monate nach Abschlussstaging nach Chemotherapie und Vorliegen des 1. Follow-Up-Bogens;
- protokollgerechte Behandlung;
- bekannte Gründe für Studienabbruch.

Der Therapieverlauf, das endgültige Therapieergebnis und der Zeitpunkt des Endes der protokollgerechten Therapie werden auf der Auswertbarkeitserklärung dokumentiert und vom Review-Panel datiert unterzeichnet. Patienten mit vorliegender Auswertbarkeitserklärung können bei Zwischenauswertungen mit Analysen zur Wirksamkeit berücksichtigt werden. Bei Analysen zur Sicherheit und zur Auswertung der weiteren Nebenzielkriterien können alle Patienten mit mindestens einem durchgeführten und dokumentierten Chemotherapiezyklus (Vorphase eingeschlossen) berücksichtigt werden.
Alle Auswertungen werden nach dem Intention-to-treat Prinzip durchgeführt, dass heißt alle Patienten werden in dem Therapiearm ausgewertet, für den sie randomisiert wurden. Sollte die Protokolladhärenz wider Erwarten deutlich schlechter als bei der MInT-Studie sein und es gehäuft zum Wechseln des Therapiearms kommen, müssen zusätzlich „treatment given“ und weitere „per protocol“ Analysen durchgeführt werden, um den beobachteten Therapieeffekt besser einschätzen zu können.
Wir erwarten kaum Auswertungsverluste wegen mangelnder Dokumentation. Sollten die Dokumentationsverluste 10 % übersteigen, wird eine Sensitivitätsanalyse durchgeführt,

welche zur Überprüfung der Strukturgleichheit des auswertbaren und nicht auswertbaren Kollektivs dient.

7.5.2 Analyse zur Wirksamkeit und Sicherheit

7.5.2.1 Hauptzielkriterium

In einer primären Analyse wird der log-rank Test für den Vergleich der Zeit bis zum Therapieversagen für die beiden Fragestellungen benutzt. Dabei bezieht sich der log-rank Test bei der Fragestellung zur Immuno-Chemotherapie auf das Gesamtkollektiv und auf die Patienten mit initialem Bulk und bei der Strahlentherapiefragestellung auf die Patienten mit initialem Bulk. Zur Deskription sollen auch die Kaplan-Kurven der einzelnen Therapiearme dargestellt werden. Zusätzlich werden die 3-Jahres-TTF-Raten mit 95% Konfidenzintervall angegeben.
In einer sekundären Analyse wird mit dem multivariaten Regressionsmodell von Cox überprüft, ob die univariat getesteten Therapieeffekte auch nach Adjustierung für prognostische Faktoren stabil bleiben. Zusätzlich werden Interaktionen zwischen Therapieeffekt und prognostischen Faktoren (Shipp-Kriterien und Variablen mit prognostischer Relevanz innerhalb dieser Studie) untersucht, um zu prüfen, ob der Behandlungseffekt in verschiedenen prognostischen Teilkollektiven homogen ist. Es sind Subgruppenanalysen innerhalb der IPI-Gruppen geplant. Da im Verlauf der Studie neue Erkenntnisse zu der Rolle des Geschlechts unter rituximabhaltigen Schemata gewonnen wurden[46], sollen die Auswertungen auch getrennt nach Geschlecht erfolgen.
Die Schätzer werden als Hazard Ratio mit 95%-Konfidenzintervall und zugehörigem p-Wert angegeben. Ist die proportionale Hazardannahme verletzt, wird der Einsatz geeigneterer Auswertungsmethoden geprüft.
Im Rahmen der Analyse zur Wirksamkeit wird eine Übersicht erstellt, wie viele Patienten randomisiert wurden und auswertbar bezüglich der Haupt- und Nebenzielkriterien sind. Es wird detailliert angegeben wie viele Patienten das Therapieende erreicht haben bzw. wie viele Patienten vorzeitige abgebrochen haben und zu welchem Zeitpunkt. Die Auswertungen zum Hauptzielkriterium der Studie werden für das Intention-to-Treat Kollektiv und das ‚per protocol' Kollektiv (Patienten, welche die Aufnahmekriterien erfüllen und eine referenzpathologische Beurteilung haben) durchgeführt. Die gesamte Abschlussauswertung wird nach einem im Vorfeld klar definierten Auswertungsplan durchgeführt.

7.5.2.2 Nebenzielkriterien

Die konsolidierende Strahlentherapie extranodaler Befälle wird analog der Strahlentherapie für Bulk-Befälle untersucht.
Die CR-Raten und die PRO-Raten werden mit dem 95%-Konfidenzintervall angegeben. Die Überlebenszeitdaten der Nebenzielkriterien werden analog der primären Analyse des Hauptzielkriteriums ausgewertet (s. 7.5.2.1). Für qualitative Nebenzielkriterien wie Unerwünschte und Schwerwiegende Unerwünschte Ereignisse werden Häufigkeitsauszählungen angefertigt. Der Anteil der Patienten mit Schwerwiegenden Unerwünschten Ereignissen wird angegeben. Quantitative Nebenzielkriterien wie Laborwerte, Gesamtdosis der Zytostatika/Rituximab und G-CSF, Tage im Krankenhaus, Gesamttage der Antibiotikagabe, Gesamtzahl der Erythrozyten- und Thrombozytenkonzentrate, Zyklusdauer und Relative Dosisintensität werden durch Lagemaße (arithmetisches Mittel bzw. Median) und Streuungsmaße (Standardabweichung bzw. unteres und oberes Quartil) beschrieben. Als grafische Darstellung werden Fehlerbalken bzw. Boxplots verwendet. Die Auswertung der Nebenzielkriterien erfolgt auch getrennt nach Therapiearm.

8 Dokumentation und Monitoring

8.1 Aufbau der Dokumentationsmappe

Die teilnehmenden Institutionen verpflichten sich zu einer sorgfältigen und vollständigen Dokumentation des Krankheitsverlaufes jedes Patienten. Nach Aufnahme des Patienten in die Studie erhält der behandelnde Arzt umgehend eine Dokumentationsmappe für diesen Patienten vom Studiensekretariat in Homburg.
Die Dokumentationsmappe enthält:

- die Randomisationsbescheinigung
- Bögen zur Materialeinsendung für die wissenschaftlichen Begleitprojekte
- das Flowsheet der Studie
- eine Seite mit Ansprechpartnern
- Hinweise zum Ausfüllen der Dokumentationsbögen
- Adressetiketten zum Einsenden der Bögen
- Dokumentationsbögen (außer Staging-Bogen – muss schon bei Randomisation vorliegen) (s. Anhang 14.15)
- Bögen zur Meldung Schwerwiegender Unerwünschter Ereignisse
- ggf. Dokumentationsbögen für den Strahlentherapeuten (Arme ARX und BRX)

8.2 Bearbeitung der ausgefüllten Dokumentationsbögen

Die sorgfältig ausgefüllten Dokumentationsbögen werden an das Studiensekretariat geschickt:

Studiensekretariat der DSHNHL
Prof. Dr. M. Pfreundschuh
Universitätsklinikum des Saarlandes
Innere Medizin I, Geb. 40
D-66421 Homburg/Saar

Die Bearbeitung der eingesandten Dokumentationsbögen erfolgt entsprechend den in SOPs festgelegten Verfahren in mehreren Schritten:

Schritt 1 (Vorprüfung und 1. Monitoring):

Alle Dokumentationsbögen werden vom Studienarzt in der Studienzentrale in Homburg im Sinne einer medizinischen Vorprüfung auf folgende Gesichtspunkte hin überprüft:

- Abweichen vom Studienprotokoll;
- Auftreten von Unerwünschten Ereignissen;
- Schwerwiegende Unerwünschte Ereignisse (SUE-Meldefax)

Dies dient dazu, rechtzeitig medizinische Probleme zu erkennen und gezielte Nachfragen auszulösen. Außerdem werden die Dokumentationsbögen auf Plausibilität und

Vollständigkeit überprüft. Bei medizinischen Unklarheiten ruft der Studienarzt gegebenenfalls beim behandelnden Arzt an, um Missverständnisse aufzuklären oder ausstehende Informationen zu erhalten. Sich daraus ergebende Datenkorrekturen werden auf den jeweiligen Dokumentationsbögen vermerkt und vom Studienarzt datiert unterzeichnet.

Schritt 2 (2. Monitoring):

In der Studienzentrale in Homburg werden alle Dokumentationsbögen registriert und auftretende Ereignisse und Protokollabweichungen in der Datenbank vermerkt. Die Bögen werden auf Vollständigkeit, Plausibilität und Korrektheit durch den Monitor geprüft. Gegebenenfalls wird die teilnehmende Institution schriftlich oder telefonisch um ausstehende oder um zusätzliche Informationen gebeten. Ausstehende Dokumentationen werden regelmäßig angemahnt.
Beim Monitoring wird vor allem auf eine kontinuierliche Nachsorge geachtet und gegebenenfalls Patienten zur Vorstellung bei Ärzten ihrer Wahl aufgefordert. Es werden außerdem die Urteile der Referenzpathologie und Referenzstrahlentherapie sowie die Daten aus den Begleituntersuchungen eingeholt.

Schritt 3 (Datenbank, Data Entry):

Die vorgeprüften Dokumentationsbögen werden über die Formmasken in die ORACLE – Datenbank eingegeben. Die Datenqualität wird hierbei über Constraints und Trigger kontrolliert. Alle Daten werden zur Gewährleistung einer hohen Datenintegrität in einem sogenannten „second look“ von einem zweiten Datenerfasser geprüft.

Schritt 4 (Auswertbarkeit):

In gemeinsamen Konferenzen von Studienarzt, Monitor und Biometriker wird die Auswertbarkeit jedes einzelnen Patienten nach Vorliegen der gesamten Therapiedokumentation und des Urteils der Referenzpathologie erklärt und die Wertung eventueller Protokollabweichungen festgelegt.

Die Vorprüfung und das 1. Monitoring der Dokumentationsbögen (Schritt 1) sowie die Schritte 2 und 3 erfolgen im NHL-Studiensekretariat in Homburg (Prof. Pfreundschuh). Schritt 4 (Auswertbarkeitserklärung) erfolgt im Rahmen halbjährlicher Treffen von Studienarzt, Biometriker und Dokumentar.

8.3 Monitoring vor Ort

Die Studienleitung ist angehalten, die Qualität der Dokumentation durch die Studienteilnehmer stichprobenartig vor Ort zu verifizieren (“On-site Monitoring“ zur „Source Data Verification“). Die teilnehmenden Institutionen verpflichten sich, den „On-site Monitoren“ Einsicht in die Originalunterlagen des Patienten (Krankenakte, Laborblätter, Originalaufnahmen von bildgebenden Verfahren etc.) zu gewähren.

9 Referenzbeurteilungen

9.1 Referenzpathologische Beurteilung

Die Referenzbeurteilung für die DSHNHL-Studien kann durch folgende Pathologen erfolgen:

- Prof. Rosenwald, Würzburg (Federführung)
- Prof. Hansmann, Frankfurt
- Prof. Möller, Ulm
- Prof. Feller, Lübeck
- Prof. Klapper, Kiel
- Prof. Stein, Berlin

Die histologische Diagnose erfolgt primär durch den lokalen Pathologen aufgrund einer Biopsie eines vollständig exzidierten Lymphknotens. Ersatzweise kann die Diagnose auch aus einer adäquaten Biopsie eines anderen befallenen Organs erfolgen, wenn kein Lymphknotenbefall vorliegt. Die Diagnose "aggressives Lymphom, $CD20^{+}$" durch den lokalen Pathologen rechtfertigt die Meldung und Randomisation in der Studie.
Wann immer möglich, sollte Frischmaterial oder zumindest (formalinfixiertes) Nass- bzw. Frischmaterial für immunologische und molekularbiologische Untersuchungen asserviert bzw. direkt an die Referenzpathologie geschickt werden. Frischmaterial sollte in einem Weichplastikröhrchen in physiologischer Kochsalzlösung schockgefroren werden (flüssiger Stickstoff). Für molekularbiologische Untersuchungen sowie eingeschränkt immunhistochemische Untersuchungen ist auch Material zu verwenden, das im Rahmen einer gegebenenfalls stattgehabten Schnelldiagnostik tiefgefroren wurde.
Nach Aufnahme des Patienten in die Studie erhält der lokale Pathologe, der die Diagnose gestellt hat, ein Schreiben des federführenden Referenzpathologen mit der Bitte, die Paraffinblöcke und eventuelles Restmaterial mit einer Kopie seines Befundberichtes an einen der Referenzpathologen zu schicken sowie den beigefügten Meldezettel mit der Information, an welches Referenzzentrum das Material gesandt wurde, an das Studiensekretariat in Homburg zu faxen. Hier erfolgt dann das Monitoring zum Materialversand und zur Diagnoseverschlüsselung.
Ohne referenzpathologisches Urteil des paraffineingebetteten Gewebes wird ein Patient nur Intention-to-Treat ausgewertet.
Die Referenzpathologie bearbeitet das eingegangene Material in zwei Schritten:

Schritt 1:

Nach Eingang der Proben wird vom Referenzpathologen umgehend festgestellt, ob ein aggressives Non-Hodgkin-Lymphom vorliegt, oder sich der Verdacht auf eine andere maligne Erkrankung ergibt. Sollte sich hierdurch eine therapierelevante Änderung der Diagnose ergeben, so wird diese Feststellung *sofort* den einsendenden Hämato-Onkologen, den primär untersuchenden Pathologen und der Studienzentrale mitgeteilt. Das Ziel dieses ersten Schrittes ist es, die Diagnose abzusichern.

Schritt 2:

Die für eine Referenzbeurteilung ausgewählten Schnitte werden in gemeinsamen Sitzungen unabhängig voneinander beurteilt. Zur Auswahl der Schnitte und zur Bildung des Konsensurteils existieren SOPs. Über das Ergebnis der endgültigen wissenschaftlichen Klassifikation durch das Gremium der Referenzpathologen werden die Studienzentrale und der primär untersuchende Pathologe unterrichtet.

9.2 Referenzstrahlentherapeutische Beurteilung

Dem DSHNHL-Referenzpanel der Strahlentherapeuten gehören folgende Mitglieder an:

- Prof. Dr. Ch. Rübe, Homburg (Leitung und Koordination)
- Dr. M. Engelhard, Essen
- Prof. Dr. H. Schmidberger, Mainz

Nach Beendigung der konsolidierenden Strahlentherapie wird der ausgefüllte Strahlentherapiebogen sowie die Unterlagen zur Planung, Simulation und Verifikation an die Studienleitung geschickt. Er wird zunächst vom federführenden Referenzstrahlentherapeuten beurteilt. Sollte sich aufgrund dieser Dokumentation die Annahme eines Fehlers bei der Durchführung der Strahlentherapie ergeben, so unterrichtet der Referenzstrahlentherapeut den behandelnden Strahlentherapeuten. Das Panel wird alle Rezidivfälle im Sinne einer Qualitätskontrolle untersuchen.

10 Wissenschaftliche Begleitprojekte und krebshilfe verbundprojekt „molekulare mechanismen bei malignen lymphomen“

Zu definierten Zeitpunkten innerhalb der Studie (Ersterhebung, Zwischen- und Abschluss-staging, Nachsorge) soll Material für wissenschaftliche Begleituntersuchungen entnommen und eingesandt werden. Die entsprechenden Materialentnahmen sind zu den jeweiligen Zeitpunkten klinisch indiziert und stellen keine zusätzliche Belastung für den Patienten dar. Durch die enge Zusammenarbeit mehrerer Forschergruppen im Rahmen einer multizentrischen Studie und dem dadurch möglichen Vergleich wissenschaftlicher und klinischer Daten besteht die Chance, den klinischen Stellenwert experimentell erhobener Daten zu bestimmen. Die Patienten stimmen mit ihrer Unterschrift auf der Patienteneinverständniserklärung der Durchführung der wissenschaftlichen Untersuchungen zu unter der Bedingung, dass keine kommerziellen Projekte durchgeführt werden. Die Patienten stellen der Studienzentrale Lymphknoten- und Blutproben zur Verfügung.
Darüber hinaus wird es durch finanzielle Förderung seitens der Deutschen Krebshilfe im Rahmen des Verbundprojektes „Molekulare Mechanismen bei malignen Lymphomen“ erstmals möglich sein, bei allen Patienten dieser Studie neben den etablierten zytogenetischen Untersuchungen auch und vor allem Genexpressionsanalysen durchzuführen. Hierzu ist eine besondere Aufarbeitung des frischen Lymphknotenmaterials zur Gewinnung schockgefrorener Proben notwendig, die im Anhang beschrieben ist. Weitergehende Informationen, Anforderung von speziellem Verpackungsmaterial und Versandorganisation sowie Fragen zur finanziellen Kompensation sind über die Zentrale des Krebshilfe Verbundprojektes in Göttingen verfügbar.
Die behandelnden Ärzte werden gebeten, das Material zum Zeitpunkt des Stagings, Restagings und während der Nachsorge an die entsprechenden Sammelstellen für Material zu schicken.
In Abhängigkeit von der Art des Materials erfolgt die Sammlung an unterschiedlichen Stellen:

1) Blut, Serum, Knochenmark:

Studiensekretariat der DSHNHL
Prof. Dr. M. Pfreundschuh
Universitätsklinikum des Saarlandes
Innere Medizin I, Geb. 40
D-66421 Homburg/Saar

2) Referenzpathologie (formalinfixiert) und schockgefrorenes Frischmaterial (Bitte Anleitung zur Materialaufarbeitung im Anhang beachten):

Zentrale Krebshilfe Verbundprojekt (Prof. Dr. L. Trümper)
Verbundsekretariat
(Dr. Benjamin Stürzenhofecker, Maren Wehner), Göttingen

Hotline: 0700/59674663 („0700/Lymphome“)
Tel.: 0551/3914396, Fax. 0551/398587
E-Mail: lymphomverbund@medizin.uni-goettingen.de

Das **Scientific Advisory Board der DSHNHL** beurteilt in Zusammenarbeit mit der Studienleitung die Anträge interessierter Arbeitsgruppen. Diesem Advisory Panel gehören neben Vertretern der Studienleitung, der Referenzpathologie und der Biometrie ausgewiesene Experten auf dem Gebiet an (s. 0.2.3).
Für positiv begutachtete Anträge wird in Zusammenarbeit mit der Biometrie von den beteiligten Wissenschaftlern festgelegt, welche Stichproben (Patientenkollektive) in die Untersuchungen eingeschlossen werden und welche Daten wann der Biometrie übermittelt werden. Dabei werden den wissenschaftlichen Arbeitsgruppen klinische Daten für ihre Auswertung zur Verfügung gestellt. Klinische bzw. biometrische Daten werden von der Studienzentrale nur für solche wissenschaftlichen Projekte zur Verfügung gestellt, die vom Advisory Board empfohlen wurden.
Die Durchführung der Projekte wird auf jährlich stattfindenden Statusseminaren begutachtet.

11 Ethische Grundlagen

Die vorliegende Studie wird unter Berücksichtigung der ICH-GCP-Kriterien durchgeführt. Jeder Studienteilnehmer verpflichtet sich, die Deklaration von Helsinki zu beachten.
Das Protokoll unterliegt noch der 11. AMG-Novelle, da es am 16.07.04 bei der Ethikkommission der Ärztekammer des Saarlandes eingereicht wurde.
Der Protokollentwurf dieser Studie wurde auf dem Studientreffen am 18.06.2004 von der Protokollkommission der DSHNHL und den Teilnehmern der Studie gebilligt. Das Studienprotokoll liegt derzeit dem zuständigen regionalen Ethikkomitee, der Ethikkommission der Ärztekammer des Saarlandes vor. Die Ethikkommission wird über mögliche Änderungen oder Addenda zu diesem Protokoll (Dosisänderungen, Verlängerung der Behandlungsdauer, Verlängerung der Studiendauer, Änderung der Eingangskriterien, Erhöhung der Patientenzahl) umgehend informiert. Die Änderungen treten erst in Kraft, wenn ein positives Votum der zuständigen Ethikkommission vorliegt. Sollten gehäuft Schwerwiegende Unerwünschte Ereignisse (SUE) eintreten, so wird die Ethikkommission hierüber informiert und ein Votum eingeholt, ob Bedenken gegen eine Fortführung der Studie oder eines Studienarms bestehen.
Wir weisen darauf hin, dass regional abhängig Studienteilnehmer die für ihr Zentrum zuständige Ethikkommission informieren und die Notwendigkeit eines separaten Votums klären müssen; Patienten dürfen in diesen Fällen erst dann in die Studie aufgenommen werden, wenn das positive Votum der zuständigen Ethikkommission vorliegt!

12 Organisation

12.1 Datenverarbeitung und Archivierung

Die Daten der Dokumentationsbögen werden über Eingabemasken in einer Oracle-Datenbank erfasst. Bei der Eingabe werden die Daten über ein mehrstufiges Konzept durch Trigger und Constraints auf Korrektheit und Konsistenz überprüft. Die Studiendatenbank wird vor dem Einsatz durch den Datenbankprogrammierer in Zusammenarbeit mit dem verantwortlichen Biometriker und den Dokumentaren auf Fehler überprüft, validiert und zum Einsatz freigegeben. Es erfolgt eine tägliche Komplettsicherung aller Daten. Durch den Einsatz eines hierarchischen auf Rollen basierenden Zugriffskonzept ist ein unberechtigter Zugriff auf die Patientendaten unmöglich. Die Anonymität der Daten im Rahmen von Auswertungen ist sichergestellt. Die Dokumentationsbögen werden mindestens 10 Jahre im Studiensekretariat in Homburg aufbewahrt. Die elektronisch erfassten Daten werden mindestens 20 Jahre lang bei der Biometrie in Leipzig aufbewahrt. Die Zwischen- und Abschlussberichte der Studien werden im Studiensekretariat in Homburg 20 Jahre lang aufbewahrt.
Die behandelnden Ärzte der teilnehmenden Zentren sollen die Studienunterlagen (Patienteneinverständniserklärung, Protokoll der Patienteneinverständniserklärung, ausgefüllte und eingesandte Dokumentationsbögen) bis zum Zeitpunkt des Vorliegens des Abschlussberichtes der Studie aufbewahren.

12.2 Nachträgliche Protokolländerungen

Nachträgliche Änderungen des Protokolls können nur durch Beschluss der Protokollkommission erfolgen. Die Protokollkommission beschließt auch, wann solche Protokolländerungen wirksam werden sollen. Sind die Protokolländerungen schwerwiegend, d.h. betreffen sie Modifikationen eines Therapiearmes, Ein- oder Ausschlusskriterien, Rekrutierungszahl und Dauer, oder die Schließung eines Therapiearmes oder der gesamten Studie, so ist grundsätzlich das Votum des DMSC einzuholen. Die gemeinsame Studienleitkommission im Studienhaus Onkologie der Deutschen Krebsgesellschaft wird unterrichtet. Für alle nachträglichen Änderungen wird ein Votum der zuständigen Ethikkommission eingeholt. Bestehen gegen die Protokolländerungen keine Bedenken, werden die Studienteilnehmer über diese Protokolländerungen schriftlich informiert. Außerdem werden im Studienbuch Datum der Protokolländerung durch die Protokollkommission, des Votums der Ethikkommission, der Information der Studienteilnehmer und des Inkrafttretens der Änderungen festgehalten. Die Änderungen müssen außerdem als Amendement im Studienprotokoll festgehalten werden.

12.3 Finanzierung und Versicherungen

Eine Unterstützung für die Durchführung dieser Studie durch die Deutsche Krebshilfe / Mildred-Scheel-Stiftung liegt vor.
Alle Patienten werden durch einen Vertrag mit dem Gerling-Konzern (Köln) im Rahmen einer Probandenversicherung versichert. Den Patienten ist eine Kopie des Versicherungsscheines auszuhändigen (s. Anhang 14.8).

12.4 Publikationsvereinbarungen

Die Ergebnisse der Studie sollen in international anerkannten wissenschaftlichen Zeitschriften publiziert werden. Über die Autorenschaft entscheidet die Protokollkommission. Dabei soll neben dem konzeptuellen Beitrag auch die aktive Mitarbeit in der Studie gemessen an der Zahl der rekrutierten Patienten Berücksichtigung finden. Manuskripte dürfen erst eingereicht werden, wenn alle Autoren ihre Zustimmung zum Inhalt des Manuskripts gegeben haben. Der federführende Autor geht von der Zustimmung der Koautoren aus, wenn er nicht binnen 2 Wochen nach Verschickung des Manuskriptentwurfs von den Koautoren über Änderungswünsche informiert wird.

13 Literaturverzeichnis

1. A predictive model for aggressive non-Hodgkin's lymphoma. The International Non-Hodgkin's Lymphoma Prognostic Factors Project. N.Engl.J.Med. 1993;329:987-994.

2. Harris NL, Jaffe ES, Diebold J et al. World Health Organization classification of neoplastic diseases of the hematopoietic and lymphoid tissues: report of the Clinical Advisory Committee meeting-Airlie House, Virginia, November 1997. J.Clin.Oncol. 1999;17:3835-3849.

3. Hiddemann W, Longo DL, Coiffier B et al. Lymphoma classification--the gap between biology and clinical management is closing. Blood 1996;88:4085-4089.

4. Vaughan Hudson B., Vaughan Hudson G., Maclennan KA, Anderson L, Linch DC. Clinical stage 1 non-Hodgkin's lymphoma: long-term follow-up of patients treated by the British National Lymphoma Investigation with radiotherapy alone as initial therapy. Br.J.Cancer 1994;69:1088-1093.

5. McKelvey EM, Gottlieb JA, Wilson HE et al. Hydroxyldaunomycin (Adriamycin) combination chemotherapy in malignant lymphoma. Cancer 1976;38:1484-1493.

6. Yi PI, Coleman M, Saltz L et al. Chemotherapy for large cell lymphoma: a status update. Semin.Oncol. 1990;17:60-73.

7. Canellos GP, Skarin AT, Klatt MM et al. The m-BACOD combination chemotherapy regimen in the treatment of diffuse large cell lymphoma. Semin.Hematol. 1987;24:2-7.

8. Klimo P, Connors JM. Updated clinical experience with MACOP-B. Semin.Hematol. 1987;24:26-34.

9. McMaster ML, Greer JP, Greco FA et al. Effective treatment of small-noncleaved-cell lymphoma with high- intensity, brief-duration chemotherapy. J.Clin.Oncol. 1991;9:941-946.

10. Coiffier B, Gisselbrecht C, Herbrecht R et al. LNH-84 regimen: a multicenter study of intensive chemotherapy in 737 patients with aggressive malignant lymphoma. J.Clin.Oncol. 1989;7:1018-1026.

11. Straus DJ, Wong GY, Liu J et al. Small non-cleaved-cell lymphoma (undifferentiated lymphoma, Burkitt's type) in American adults: results with treatment designed for acute lymphoblastic leukemia. Am.J Med. 1991;90:328-337.

12. Fisher RI, Gaynor ER, Dahlberg S et al. Comparison of a standard regimen (CHOP) with three intensive chemotherapy regimens for advanced non-Hodgkin's lymphoma. N.Engl.J.Med. 1993;328:1002-1006.

13. Pfreundschuh M, Trumper L, Kloess M et al. Two-weekly or 3-weekly CHOP chemotherapy with or without etoposide for the treatment of young patients with good-prognosis (normal LDH) aggressive lymphomas: results of the NHL-B1 trial of the DSHNHL. Blood 2004;104:626-633.

14. Hasenclever D, Brosteanu O, Gerike T, Loeffler M. Modelling of chemotherapy: the effective dose approach. Ann.Hematol. 2001;80 Suppl 3:B89-B94.

15. Pfreundschuh M, Truemper L, Ma D et al. Randomised Intergroup Trial of first line treatment for young low-risk patients (<61 years) with diffuse large B-cell non-Hodgkin´s lymphoma with a CHOP-like regimen with or without the anti-CD20

antibody rituximab – early stopping after first interim analysis. Proc.Am.Soc Clin.Oncol. 2004;23:

16. Pfreundschuh M, Truemper L, Ma D et al. The MInT trial (CHOP-like regimens with or without rituximab for young low-risk patients with diffuse large B-cell lymphoma): early stopping after first interim analysis. Hematology Journal 2004;5 (supplement 2):S 204.

17. Miller TP, Dahlberg S, Cassady JR et al. Chemotherapy alone compared with chemotherapy plus radiotherapy for localized intermediate- and high-grade non-Hodgkin's lymphoma. N.Engl.J.Med. 1998;339:21-26.

18. Reyes F, Lepage E, Munck JN et al. Superiority of chemotherapy alone with the ACVBP regimen over treatment with three cycles of CHOP plus radiotherapy in low-risk localized aggressive lymphoma: The LNH93-1 GELA study. Blood 2002;100;93 a (Abstract #343).

19. Pfreundschuh M, Ho A, Wolf M et al. Treatment results of CHOP-21, CHOEP-21, MACOP-B and PMitCEBO with and without rituximab in young good-prognosis patients with aggressive lymphomas: Rituximab as and "equalizer" in the MInT study. J Clin.Oncol 2005;23:566s.

20. Pfreundschuh M, Trumper L, Kloess M et al. Two-weekly or 3-weekly CHOP chemotherapy with or without etoposide for the treatment of elderly patients with aggressive lymphomas: results of the NHL-B2 trial of the DSHNHL. Blood 2004;104:634-641.

21. Milpied N, Deconinck E, Gaillard F et al. Initial treatment of aggressive lymphoma with high-dose chemotherapy and autologous stem-cell support. N.Engl.J Med. 2004;350:1287-1295.

22. Vokes EE, Ultmann JE, Golomb HM et al. Long-term survival of patients with localized diffuse histiocytic lymphoma. J.Clin.Oncol. 1985;3:1309-1317.

23. Hoederath A, Sack H, Stuschke M, Lampka E. Radiotherapy of primary extranodal non-Hodgkin's lymphoma of the head and neck region. Results of a prospective multicenter study. Study Group NHL: early studies. Strahlenther.Onkol. 1996;172:356-366.

24. Kaminski MS, Coleman CN, Colby TV, Cox RS, Rosenberg SA. Factors predicting survival in adults with stage I and II large-cell lymphoma treated with primary radiation therapy. Ann.Intern.Med. 1986;104:747-756.

25. Brierley JD, Rathmell AJ, Gospodarowicz MK, Sutcliffe SB, Pintillie M. Late relapse after treatment for clinical stage I and II Hodgkin's disease. Cancer 1997;79:1422-1427.

26. Tondini C, Zanini M, Lombardi F et al. Combined modality treatment with primary CHOP chemotherapy followed by locoregional irradiation in stage I or II histologically aggressive non-Hodgkin's lymphomas. J Clin.Oncol. 1993;11:720-725.

27. Shenkier TN, Voss N, Fairey R et al. Brief chemotherapy and involved-region irradiation for limited-stage diffuse large-cell lymphoma: an 18-year experience from the British Columbia Cancer Agency. J Clin.Oncol. 2002;20:197-204.

28. de Jong D, Glas AM, Boerrigter L et al. Very late relapse in diffuse large B-cell lymphoma represents clonally related disease and is marked by germinal center cell features. Blood 2003;102:324-327.

29. Glick J, Kim K, Earle J, O´Connell M. An ECOG randomized phase III trial of CHOP vs. CHOP+radiotherapy for intermiediate grade early stage non-Hodgkin´s lymphoma. Proc.Am.Soc Clin.Oncol. 1995;18:391.

30. Horning SJ, Glick J, Kim K. CHOP vs. CHOP + radiotherapy for limited-stage diffuse aggressive lymphoma. Blood 2001;100:724a.

31. Horning SJ, Weller E, Kim K et al. Chemotherapy with or without radiotherapy in limited-stage diffuse aggressive non-Hodgkin's lymphoma: Eastern Cooperative Oncology Group study 1484. J Clin.Oncol 2004;22:3032-3038.

32. Miller TP, LeBlanc M, Spier C, Chase E, Fisher RI. CHOP alone compared to CHOP plus radiotherapy for early stage aggressive non-Hodgkin´s lymphomas: Update of the Sotuhwest Oncology Gourp randomized trial. Blood 2001;98:724a.

33. Reyes F, Lepage E, Ganem G et al. ACVBP versus CHOP plus radiotherapy for localized aggressive lymphoma. N.Engl.J Med. 2005;352:1197-1205.

34. Aviles A, Delgado S, Nambo MJ, Alatriste S, Diaz-Maqueo JC. Adjuvant radiotherapy to sites of previous bulky disease in patients stage IV diffuse large cell lymphoma. Int.J.Radiat.Oncol.Biol.Phys. 1994;30:799-803.

35. Rube C, Nguyen TP, Kloss M et al. Consolidation radiotherapy to bulky disease in aggressive NHL. First results of the NHL B-94 trial of the DSHNHL. Ann.Hematol. 2001;80 Suppl 3:B84-B85.

36. Fillet G, Bonnet C, Mounier N et al. Radiotherapy is unnecessary in elderly patients with localied aggressive non-Hodgkin´s lymphoma: results of the GELA LNH 93-4 study. Blood 2002;100:92a.

37. Wilder RB, Rodriguez MA, Tucker SL et al. Radiation therapy after a partial response to CHOP chemotherapy for aggressive lymphomas. Int.J Radiat.Oncol Biol.Phys. 2001;50:743-749.

38. Spaepen K, Stroobants S, Verhoef G, Mortelmans L. Positron emission tomography with [(18)F]FDG for therapy response monitoring in lymphoma patients. Eur.J.Nucl.Med.Mol.Imaging 2003

39. Cheson BD, Horning SJ, Coiffier B et al. Report of an international workshop to standardize response criteria for non-Hodgkin's lymphomas. NCI Sponsored International Working Group. J.Clin.Oncol. 1999;17:1244.

40. Lister TA, Crowther D, Sutcliffe SB et al. Report of a committee convened to discuss the evaluation and staging of patients with Hodgkin's disease: Cotswolds meeting. J.Clin.Oncol. 1989;7:1630-1636.

41. Griggs JJ, Mangu PB, Anderson H et al. Appropriate chemotherapy dosing for obese adult patients with cancer: American Society of Clinical Oncology clinical practice guideline. J.Clin.Oncol. 2012;30:1553-1561.

42. Schmitz N, Zeynalova S, Glass B et al. CNS Disease In Younger Patients (<=60 years) with Aggressive Lymphoma Treated In Trials of the German High Grade Non Hodgkin Lymphoma Study Group (DSHNHL) and the MabThera International Trial (MInT). Blood 2010;116:abstract #112.

43. Pocock SJ. Clinical Trials.; 1983.

44. DeMets DL, Lan KK. Interim analysis: the alpha spending function approach. Stat.Med. 1994;13:1341-1352.

45. O'Brien PC, Fleming TR. A multiple testing procedure for clinical trials. Biometrics 1979;35:549-556.

46. Muller C, Murawski N, Wiesen MH et al. The role of sex and weight on rituximab clearance and serum elimination half-life in elderly patients with DLBCL. Blood 2012;119:3276-3284.

14 Anhang

14.1 Formular der Teilnahmeerklärung der Zentren

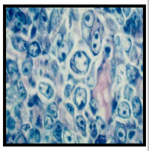

DEUTSCHE STUDIENGRUPPE HOCHMALIGNE NON-HODGKIN-LYMPHOME*
German High Grade Non-Hodgkin's Lymphoma Study Group

***(gefördert durch die Deutsche Krebshilfe)**

An das<br>Studiensekretariat der DSHNHL<br>Prof. Dr. M. Pfreundschuh<br>Med. Klinik und Poliklinik, Innere Medizin I<br>Universitätsklinikum des Saarlandes<br>66421 HOMBURG/SAAR<br>Tel: 06841/16-23084 Fax: 06841/16-23004	**Teilnahmeerklärung des Zentrums – I**<br>**DSHNHL 2004-3**

**FÖRMLICHE ERKLÄRUNG ZUR TEILNAHME
AN DER THERAPIEOPTIMIERUNGSSTUDIE DSHNHL 2004-3
(6 x R-CHOP-21 vs. 6 x R-CHOP-14, jeweils mit oder ohne Strahlentherapie)
FÜR AGGRESSIVE NON-HODGKIN-LYMPHOME**

Mit meiner Unterschrift erkläre ich mich bereit, mit meiner Institution an der oben genannten Studie teilzunehmen. Ich verpflichte mich, alle Patienten mit der Diagnose „aggressives Non-Hodgkin-Lymphom", welche die Aufnahmekriterien für die Studie erfüllen, an die Studienzentrale, Professor Pfreundschuh, in Homburg zu melden. Ich werde die Patienten über den Inhalt der Studie aufklären und bei deren Einverständnis, die Patienten gemäß dem Studienprotokoll therapieren und eine kontrollierte Nachsorge durchführen.

Für die Institution, an der ich tätig bin, ist gewährleistet, dass sowohl die internistisch-chemotherapeutische Patientenbehandlung, als auch die konsolidierende Strahlentherapie nach den im Protokoll genannten Voraussetzungen durchführbar ist. Ich verpflichte mich, die Kooperationspartner (Pathologen, Strahlentherapeuten) meiner Einrichtung über die Studie zu informieren.

Ich werde der Studienzentrale denjenigen ärztliche Mitarbeiter/in bzw. Dokumentationsbeauftragten benennen, der für die Studienzentrale als Ansprechpartner zur Verfügung steht und berechtigt und verpflichtet ist, alle Belange der Meldung, Dokumentation und Nachsorge in meiner Einrichtung zu koordinieren. Sollten Ärzte die Klinik verlassen, oder Patienten die Einrichtung wechseln, wird der Studienleitung mitgeteilt, wer die Behandlung und die weitere Dokumentation für die Studienpatienten übernimmt. Patienten mit vorzeitigem Therapieabbruch werden der Studienleitung gemeldet. Wir verpflichten uns durch Materialeinsendungen die wissenschaftlichen Begleitprojekte zu unterstützen. Ich bin außerdem damit einverstanden, dass die Studienleitung stichprobenartig die protokollgerechte Teilnahme meiner Institution an der Studie vor Ort überprüft („on site monitoring") und Einsicht in die Krankenakte erhält („source data verification").

Siehe Teil II →

DEUTSCHE STUDIENGRUPPE HOCHMALIGNE NON-HODGKIN-LYMPHOME*
German High Grade Non-Hodgkin's Lymphoma Study Group

***(gefördert durch die Deutsche Krebshilfe)**

An das<br>Studiensekretariat der DSHNHL<br>Prof. Dr. M. Pfreundschuh<br>Med. Klinik und Poliklinik, Innere Medizin I<br>Universitätsklinikum des Saarlandes<br>66421 HOMBURG/SAAR<br>Tel: 06841/16-23084 Fax: 06841/16-23004	**Teilnahmeerklärung des Zentrums – II**<br>**DSHNHL 2004-3**

Weiterhin verpflichte ich mich, persönlich oder durch einen mitarbeitenden Kollegen meiner Institution vertreten, an den regelmäßig stattfindenden Arbeitssitzungen („Studientreffen“) teilzunehmen und die Dokumentationsbögen gewissenhaft und vollständig auszufüllen und umgehend der Studienzentrale zuzuleiten.

Datum: ....
T T M M J J J J

Klinikchef:...
(bitte in Druckschrift)

Unterschrift ....

Stempel der Einrichtung:

Tel.: (......................)...................-................... Fax: (......................)...................-....................

Ansprechpartner für die Studienbelange/ Dokumentationsbeauftragte(r):

Name: ....
(bitte in Druckschrift)

E-mail: ....

Tel.: (......................)...................-................... Fax: (......................)...................-....................

für Geldüberweisungen (z. B. Dokumentationsgelder):

Bank: ....

Bankleitzahl:.. Kontonummer:..

Titel: ....

Verwendungszweck: ....

14.2 Patienteninformationen und Patientenaufklärung

14.2.1 Patienteninformation zur Studie DSHNHL 2004-3 (UNFOLDER)

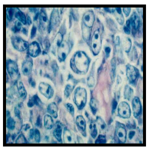

DEUTSCHE STUDIENGRUPPE HOCHMALIGNE NON-HODGKIN-LYMPHOME*
German High Grade Non-Hodgkin's Lymphoma Study Group

***(gefördert durch die Deutsche Krebshilfe)**

Studiensekretariat der DSHNHL<br>Prof. Dr. M. Pfreundschuh<br><br>Telefon für Notfälle:<br>06841/16-23084<br>06841/16-23000 (Pforte der Klinik)	**Patienteninformation – I**<br>**DSHNHL 2004-3**<br><br>**(Kopie für den Patienten)**

Sehr geehrte Patientin, sehr geehrter Patient,

wir möchten Sie um Ihr Einverständnis zur Teilnahme an einer klinischen Studie bitten. Klinische Studien dienen der Verbesserung etablierter Therapieverfahren, mit dem Ziel, die Behandlungsergebnisse zu verbessern.

Sollten Sie an bestimmten Begleiterkrankungen, und hierbei insbesondere chronischen Virusinfekten (v.a. HIV, Hepatitis), Erkrankungen von Herz, Lungen und Nieren mit deutlicher Funktionseinschränkung sowie einer anderen Tumorerkrankung in der Vergangenheit leiden, können Sie an dieser Studie nicht teilnehmen, da möglicherweise eine Verschlechterung der Begleiterkrankung oder vermehrte Nebenwirkungen der Chemotherapie auftreten können.

Ihr behandelnder Arzt hat Sie darüber aufgeklärt, dass Sie an einem aggressiven Non-Hodgkin-Lymphom erkrankt sind, einer bösartigen Erkrankung des lymphatischen Systems, die durch eine Behandlung mit Zytostatika (Zellgiften), ggf. zusammen mit einer Strahlentherapie, in vielen Fällen geheilt werden kann. Weiterhin tragen die Lymphomzellen eine Eiweißstruktur auf ihrer Oberfläche, das sogenannte CD20-Molekül, welches sich als Angriffspunkt für eine zusätzliche Immuntherapie mit dem dafür spezifischen Antikörper Rituximab anbietet. Der Antikörper ist in der Lage, diese Zellen zu erkennen, sich an diese anzuheften und dadurch Mechanismen des Immunsystems zu aktivieren, die zu einer Zerstörung der Zielzellen führen können. Zudem verstärkt er wahrscheinlich die Wirksamkeit der Chemotherapie ohne Steigerung der Nebenwirkungen. Da auf diese Weise viele, aber bei weitem noch nicht alle Patienten mit dieser Erkrankung geheilt werden können, wird zurzeit versucht, die Therapie weiter zu verbessern. Die Möglichkeit einer Verbesserung besteht darin, die Intervalldauer zwischen den Behandlungskursen der bekannt wirksamen Chemotherapie zu verkürzen.

Außerdem wurde in dieser Studie untersucht, ob eine sich nach der Chemotherapie anschließende Strahlentherapie von besonders großen Lymphommassen (über 7,5 cm; sogenannter „Bulk") und/oder von Befällen außerhalb von lymphatischen Gewebe, sogenannte Extranodalbefälle zu einer weiteren Verbesserung des Therapieergebnisses führt. Eine geplante Zwischenanalyse im Juli 2012 hatte gezeigt, dass eine solche zusätzliche Strahlentherapie die Therapieergebnisse tatsächlich hochsignifikant verbessert. Deshalb erhalten seit Juli 2012 alle Patienten mit Bulk und/oder Extranodalbefall eine zusätzliche Bestrahlung dieser Befälle.

Siehe Teil II →

DEUTSCHE STUDIENGRUPPE HOCHMALIGNE NON-HODGKIN-LYMPHOME*
German High Grade Non-Hodgkin's Lymphoma Study Group

***(gefördert durch die Deutsche Krebshilfe)**

Studiensekretariat der DSHNHL<br>Prof. Dr. M. Pfreundschuh<br><br>Telefon für Notfälle:<br>06841/16-23084<br>06841/16-23000 (Pforte der Klinik)	**Patienteninformation - II**<br>**DSHNHL 2004-3**<br><br>**(Kopie für den Patienten)**

Zweck dieser Therapieoptimierungsstudie

Obwohl es in den letzten Jahren gelungen ist, die Heilungschancen der Patienten mit aggressiven Lymphomen durch eine Intensivierung der Chemotherapie zu verbessern, erscheint eine weitere Dosissteigerung der Chemotherapie nicht mehr möglich, da eine solche Dosissteigerung zu einer Erhöhung der Rate und des Ausmaßes von Nebenwirkungen führt. Die in der Deutschen Studiengruppe Hochmaligne Non-Hodgkin-Lymphome zusammengeschlossenen Ärzte haben daher eine wissenschaftliche Studie entworfen, die prüfen soll, ob durch die Verkürzung der Zyklusdauer von drei auf zwei Wochen der derzeit als Standard anzusehenden Chemotherapie nach dem CHOP-Regime eine Verbesserung der Therapieerfolge erreicht werden kann. Aus früheren Studien (NHL-B1) der Studiengruppe liegen ausreichende Erfahrungen darüber vor, dass eine Zyklusverkürzung der bekannten wirksamen Medikamente gefahrlos und ohne wesentliche Erhöhung der Nebenwirkungen durchführbar ist.

Standardtherapie für Ihr Stadium eines aggressiven Lymphoms sind 6 Zyklen CHOP-Chemotherapie in dreiwöchigen Abständen mit 6 Applikationen des monoklonalen Antikörpers Rituximab (6 Zyklen R-CHOP-21). Da bisher nicht bekannt ist, ob eine Verkürzung der Zyklusdauer, d. h. der Intervalle zwischen den einzelen Chemotherapie-Zyklen Vorteile bringt, müssen wir die zwei Behandlungsformen 6 x R-CHOP-21 und 6 x R-CHOP-14 (intervallverkürzt) jeweils mit zusätzlicher Bestrahlung von Bulk und/oder Extranodalbefällen nach dem Zufallsprinzip miteinander vergleichen. **Dies bedeutet, dass Sie im Falle Ihrer Einwilligung zu dieser Behandlungsstudie nach dem Zufallsprinzip einem der zwei vorgesehenen Therapiearme zugeteilt werden. Auf diese Zufallszuteilung hat Ihr behandelnder Arzt keinen Einfluss.**

Studienverlauf

Sie erhalten die derzeit aus wissenschaftlicher Sicht für Ihre Erkrankung wirksamste zytostatische Kombinationschemotherapie mit 6 Zyklen R-CHOP. Im Falle von Vorliegen eines Bulks und/oder Extranodalbefall jeweils mit zusätzlicher Bestrahlung. Je nach dem Ergebnis der Randomisation erhalten Sie diese Therapie entweder alle 3 Wochen (R-CHOP-21) oder alle 2 Wochen (R-CHOP-14). Die R-CHOP Kombinationschemotherapie beinhaltet die Substanzen Cyclophosphamid, Doxorubicin, Vincristin und Prednison, sowie den monoklonalen anti-CD20-Antikörper „Rituximab". Im Fall, dass bei Ihnen große Tumormassen (Bulk) und/oder ein extranodaler Befall durch das Lymphom vorliegen, erhalten Sie im Anschluss an die Chemotherapie eine Strahlentherapie dieser Regionen.

Sollte bei Ihnen ein Befall des Hodesn vorliegen wird eine vorbeugende Bestrahlung des nicht befallenen Hodens der Gegenseite durchgeführt.

Siehe Teil III →

DEUTSCHE STUDIENGRUPPE HOCHMALIGNE NON-HODGKIN-LYMPHOME*
German High Grade Non-Hodgkin's Lymphoma Study Group

***(gefördert durch die Deutsche Krebshilfe)**

Studiensekretariat der DSHNHL<br>Prof. Dr. M. Pfreundschuh<br><br>Telefon für Notfälle:<br>06841/16-23084<br>06841/16-23000 (Pforte der Klinik)	**Patienteninformation – III**<br>**DSHNHL 2004-3**<br><br>**(Kopie für den Patienten)**

Die Studie beginnt im Oktober 2005 und wird, einschließlich Nachuntersuchungen, bis mindestens September 2017 andauern. An der Studie sollen insgesamt 1072 Patienten teilnehmen. Vor Beginn der Behandlung werden bei Ihnen ausführliche Untersuchungen durchgeführt, wie sie allgemein bei Lymphomerkrankungen auch außerhalb einer klinischen Studie, erforderlich sind. Dabei soll der genaue Ausbreitungsgrad der Erkrankung ermittelt werden. Neben Blutuntersuchungen sind die Untersuchungen des Knochenmarks (Gewebeentnahme) und Röntgenuntersuchungen einschließlich Computertomographie (CT) und/oder Ultraschalluntersuchungen notwendig. Bei Befall des Hodens sind sog. Lumbalpunktionen zur Untersuchung des Liquors (Hirn- Rückenmarksflüssigkeit) erforderlich. Neben einem Test zur Beurteilung Ihrer Herzfunktion und Lungenfunktion gehören auch Untersuchungen auf Virusinfektionen (u. a. HIV) zur initialen Befunderhebung. Durch weitere Untersuchungen im Verlauf der Studie wird die Wirkung der Behandlung auf Ihren Krankheitsverlauf überprüft. Wie bei jeder Chemotherapie eines Lymphoms sind während der Behandlungsphase wöchentliche Untersuchungen des Blutbildes (Bestimmung des Hb-Wertes sowie der Leuko- und Thrombozytenzahlen im Blut) notwendig, die auch von Ihrem Hausarzt vorgenommen werden können. Der Hausarzt wird in diesem Fall genau über Art und Zeitpunkt der notwendigen Blutbildkontrollen durch den Studienarzt schriftlich informiert. Nach Abschluss der Chemotherapie werden Sie in den ersten 2 Jahren alle 3 Monate, danach in den Jahren 3 bis 5 alle 6 Monate nachuntersucht. Die Nachbeobachtungsdauer im Rahmen der Studie beträgt für Sie 3 Jahre beginnend mit der Abschlussuntersuchung nach der Chemotherapie. Anschließend findet außerhalb der Studie eine (freiwillige) lebenslange jährliche Nachuntersuchung statt. An diesen Terminen werden Sie auch zu möglichen Nebenwirkungen, Begleitmedikationen und Infektionen befragt.

Neben den ohnehin notwendigen Untersuchungen und Blutabnahmen erfolgen im Rahmen dieser Studie keine weiteren zusätzlichen Untersuchungen. Wir werden Sie um Ihr Einverständnis bitten, dass Teile der im Rahmen der Routinediagnostik vor Therapiebeginn entnommenen Blutproben und Gewebeproben für wissenschaftliche Untersuchungen (z.B. Chromosomenveränderungen, Überexpression von Tumorwachstum fördernden Genen bzw. Verminderung von Tumorwachstum hemmenden Genen, biologische Tumormarker) eingefroren und aufbewahrt werden. Diese Untersuchungen werden durchgeführt, um die Behandlung zukünftiger Patienten gezielter durchführen zu können. Die Ergebnisse der Untersuchungen werden nicht zu kommerziellen Zwecken verwendet. Wir werden Sie um Ihr Einverständnis bitten, dass Sie Ihr Verfügungsrecht über diese Materialien der Studienleitung übertragen, die für die sachgerechte und wissenschaftlich fundierte Durchführung der Arbeiten Sorge trägt und von einem wissenschaftlichen Beratungsgremium darin unterstützt wird.

Siehe Teil IV →

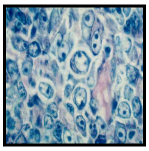

DEUTSCHE STUDIENGRUPPE HOCHMALIGNE NON-HODGKIN-LYMPHOME*
German High Grade Non-Hodgkin's Lymphoma Study Group

***(gefördert durch die Deutsche Krebshilfe)**

Studiensekretariat der DSHNHL<br>Prof. Dr. M. Pfreundschuh<br>Telefon für Notfälle:<br>06841/16-23084<br>06841/16-23000 (Pforte der Klinik)	**Patienteninformation - IV**<br>**DSHNHL 2004-3**<br><br>**(Kopie für den Patienten)**

Im Rahmen der Studie werden weiterhin die durch den behandelnden Arzt in Auftrag gegebenen pathologischen Untersuchungsbefunde sowie die entnommenen Gewebeproben zur Qualitätssicherung an eine referenzpathologische Einrichtung weitergeleitet. Diese ausgewiesenen Experten auf dem Gebiet der Lymphomerkrankungen erstellen für die DSHNHL ein Expertengutachten. Dazu ist es notwendig, Ihre Identifikationsdaten an diese Referenzzentren weiterzuleiten. Dieses ist eine Voraussetzung zur Teilnahme an der Studie.

Verantwortlichkeiten des Patienten

Sollten Sie sich dazu entschließen, an der Studie teilzunehmen, werden Sie gebeten, in folgender Weise mit Ihrem Studienarzt zusammenzuarbeiten und dabei folgende Punkte zu berücksichtigen:

- Regelmäßiges Erscheinen zu allen Kontrolluntersuchungen
- Einhalten der Anweisungen des Prüfarztes
- Mitteilung anderer Medikamente, die zeitgleich mit der Studie eingenommen werden Mitteilung von Begleiterkrankungen
- Im Falle eines vorzeitigen Therapieabbruchs zur Abschlussuntersuchung und zu den Nachsorgeuntersuchungen zu erscheinen

Schwangerschaft

Aufgrund der fruchtschädigenden (teratogenen) Eigenschaften der Chemotherapie und fehlender Daten bezüglich der Teratogenität von Rituximab **muß für alle Frauen im gebärfähigen Alter vor Therapiebeginn eine Schwangerschaft ausgeschlossen werden**. Weiterhin ist unter der Therapie und bis 12 Monate danach eine sichere Empfängnisverhütung durchzuführen, dies gilt sowohl für weibliche als auch männliche Patienten. Falls Sie bzw. Ihr (weiblicher) Partner im Verlauf der Studie oder bis 12 Monate danach schwanger werden, müssen Sie Ihren Arzt darüber umgehend informieren.

Risiken

Sowohl Chemotherapie mit Zytostatika, die Gabe des Cortisonabkömmlings Prednison, die Immuntherapie mit dem monoklonalen Antikörper Rituximab als auch die Strahlentherapie sind mit medikamentenspezifischen Nebenwirkungen behaftet. Die möglichen Nebenwirkungen der Ihnen vorgeschlagenen Behandlung umfassen im einzelnen:

Nach Chemotherapie:

Nach *Cyclophosphamid*: Übelkeit und Erbrechen; Unterdrückung der Blutbildung und Verminderung der Blutzellen, dadurch bedingtes Risiko der Infektion, Blutung und Anämie; Haarausfall; Entzündungen der Schleimhäute; Blasenschädigung; allergische Reaktionen. Entzündung der Blase oder des Darmes auftreten.

Siehe Teil V →

DEUTSCHE STUDIENGRUPPE HOCHMALIGNE NON-HODGKIN-LYMPHOME*
German High Grade Non-Hodgkin's Lymphoma Study Group

***(gefördert durch die Deutsche Krebshilfe)**

Studiensekretariat der DSHNHL<br>Prof. Dr. M. Pfreundschuh<br><br>Telefon für Notfälle:<br>06841/16-23084<br>06841/16-23000 (Pforte der Klinik)	**Patienteninformation - V**<br>**DSHNHL 2004-3**<br><br>**(Kopie für den Patienten)**

Nach *Doxorubicin*: Übelkeit und Erbrechen; Unterdrückung der Blutbildung und Verminderung der Blutzellen, dadurch bedingtes Risiko der Infektion, Blutung und Anämie; Haarausfall; Schädigung des Herzmuskels; Gewebeschädigung bei falscher Injektion der Chemotherapie; Entzündungen der Schleimhäute; allergische Reaktionen.

Nach *Vincristin*: Übelkeit und Erbrechen;Nervenschädigung; Haarausfall; Gewebeschädigung bei falscher Injektion der Chemotherapie; allergische Reaktionen.

Nach *Prednison*: Unruhe, Magenbeschwerden, Zunahme des Appetits, Osteoporose, Myopathie, steroidinduzierter Diabetes mellitus.

Während der chemotherapeutischen Behandlung sollten Sie keine alkoholhaltigen Getränke zu sich nehmen.

Nach der Strahlentherapie können auftreten: Infertilität, Dysfunktion der Leydigschen Zwischenzellen, Übelkeit, Brechreiz, Schluckbeschwerden, Kopfschmerzen, Müdigkeit, Leukozytopenie, Anämie, Hautveränderungen und Haarausfall im Strahlengebiet; möglich sind außerdem Strahlenreaktionen des Lungengewebes (Luftnot), des Darms (Durchfälle), des Herzbeutels (Erguss).

Nach der Gabe von Rituximab können auftreten: Fieber, Muskel- und Gelenkschmerzen, Blutdruckabfall sowie gelegentlich Schüttelfrost und Hautausschläge. Theoretisch sind schwere allergische Reaktionen bis hin zu einem lebensbedrohlichen Zustand denkbar (sog. „anaphylaktischer Schock").

Zur Erholung der unter Therapie abfallenden Leukozytenzahlen und damit zeitgerechten Gabe des nächsten Chemotherapiezyklus ist, insbesondere unter R-CHOP-14, die Gabe des Wachstumsfaktors G-CSF notwendig. *Unter G-CSF* kann es zu Knochenschmerzen, Temperaturerhöhung und Erhöhung des LDH-Wertes im Blut kommen.

Nach der Gabe von Immunochemotherapie oder nach Strahlentherapie kann es, noch verstärkt durch die Gabe von Medikamenten, die Übelkeit verhindern, zur vorübergehenden Beeinträchtigung der Fahrtüchtigkeit kommen. Sie sollten in der betreffenden Zeit nicht eigenhändig ein Fahrzeug führen.

Zur Vermeidung bzw. Verringerung von Nebenwirkungen und Komplikationen, die durch eine raschen Zellzerfall, insbesondere zu Beginn einer Chemotherapie auftreten können, wird eine sogenannte Vorphase vor den eigentlichen R-CHOP-Chemotherapiezyklen empfohlen. Im Rahmen dieser Vorphase, die fester Bestandteil der Behandlung ist, erhalten Sie eine intravenöse Spritze am ersten Tag, die die Chemotherapiesubstanz Vincristin enthält, und Cortisontabletten über 7 Tage.

Siehe Teil VI ➔

DEUTSCHE STUDIENGRUPPE HOCHMALIGNE NON-HODGKIN-LYMPHOME*
German High Grade Non-Hodgkin's Lymphoma Study Group

***(gefördert durch die Deutsche Krebshilfe)**

Studiensekretariat der DSHNHL<br>Prof. Dr. M. Pfreundschuh<br><br>Telefon für Notfälle:<br>06841/16-23084<br>06841/16-23000 (Pforte der Klinik)	**Patienteninformation - VI**<br>**DSHNHL 2004-3**<br><br><br>**(Kopie für den Patienten)**

Zu Beginn der Chemotherapie kann es bei raschem Zerfall großer Tumormassen zu erhöhten Harnsäurespiegeln im Blut kommen. Sie erhalten daher bereits vor Beginn der Therapie eine Tablette Allopurinol (300 mg) pro Tag. Abhängig von den Harnsäurespiegeln kann es indiziert sein, diese Therapie auch in späteren Therapiezyklen fortzusetzen. Während der gesamten Zeit der Chemotherapie sind Sie verstärkt durch Infektionen gefährdet. Dies gilt insbesondere für die Zeit zwischen Tag 6 und 12 nach Beginn des jeweiligen Chemotherapiezyklus, wenn es zum vorübergehenden Abfall der Leukozyten kommt. Während der gesamten Behandlungszeit sollten sie daher auf eine ausreichenden Hygiene, insbesondere Mundhygiene achten. Bei Abfall der Leukozyten auf weniger als 1000/µl wird eine Mundpflege nach jeder Mahlzeit mit Hexetidin und Amphothericin B empfohlen. Sollte es bei Ihnen zu einem stärkeren Abfall der Leukozyten kommen, wird Ihnen Ihr Arzt/Ärztin evtl. Antibiotika (z. B. Ciprofloxacin 2x 500mg Tablette pro Tag) zur Vermeidung von Infektion verordnen. Sie sollten diese entsprechend der Anweisungen einnehmen. Sollte es bei Ihnen in dieser Zeit zu Infektzeichen, insbesondere Fieber oder Schüttelfrost kommen, setzen Sie sich bitte umgehend mit Ihrem behandelnden Arzt bzw. einem Krankenhaus in Verbindung, um eine entsprechende Abklärung und zeitgerechte Einleitung einer Antibiotikabehandlung zu ermöglichen. Es ist möglich, dass während der Behandlung die Gabe von Blutprodukten, insbesondere von Erythrozyten (roten Blutkörperchen), seltener von Thrombozyten (Blutplättchen) erforderlich ist. Diese Gabe ist trotz sorgfältiger Untersuchung mit einem geringen Restrisiko einer Infektionsübertragung verbunden.

Möglicher Nutzen der Studienbehandlung

Falls die Verkürzung der Zyklusdauer von 21 auf 14 Tage zu einem verbesserten Ansprechen auf die Behandlung und einer Verlängerung Ihrer krankheitsfreien Zeit führt, kann dies für Sie von Nutzen sein. Ob und wie stark dieser Effekt auch im Einzelfall eintritt, kann zurzeit noch nicht gesagt werden. Ein weitergehender, möglicher Nutzen für die Allgemeinheit ist die Verbesserung der Therapiewirksamkeit bei akzeptablen Nebenwirkungen. Dieses kann nur durch den direkten Vergleich mit der bisherigen optimalen Therapie geprüft werden.

Andere Therapiemöglichkeiten

Falls Sie sich entscheiden, nicht an dieser Studie teilzunehmen, erhalten Sie die Standardtherapie (6 Zyklen R-CHOP-21) oder können Ihre Therapie frei wählen. Ihre weitere medizinische Betreuung ist nicht von Ihrer Entscheidung zur Teilnahme an dieser klinischen Prüfung abhängig.

Siehe Teil VII →

DEUTSCHE STUDIENGRUPPE HOCHMALIGNE NON-HODGKIN-LYMPHOME*
German High Grade Non-Hodgkin's Lymphoma Study Group

***(gefördert durch die Deutsche Krebshilfe)**

Studiensekretariat der DSHNHL
Prof. Dr. M. Pfreundschuh

Telefon für Notfälle:
06841/16-23084
06841/16-23000 (Pforte der Klinik)

Patienteninformation - VII
DSHNHL 2004-3

(Kopie für den Patienten)

Weitere Informationen

Dieses Studienprotokoll wurde der Ethikkommission der Ärztekammer des Saarlandes zur Prüfung auf ethische und berufsrechtliche Belange vorgelegt und in der Sitzung vom 30.09.2004 positiv begutachtet. Das Amendment vom 22.11.2012 wurde der Ethikkommission der Ärztekammer des Saarlandes zur Prüfung auf ethische und berufsrechtliche Belange vorgelegt und begutachtet. Die Teilnahme an dieser klinischen Prüfung ist freiwillig.

Ihr Arzt wird Sie bitten, ein Einverständnisformular zu unterzeichnen und damit auch zu bestätigen, dass Sie vollständig über die Studie informiert wurden und deren Zielsetzung verstehen. Der behandelnde Arzt ist bereit, alle die Prüfung betreffenden Fragen in Einzelheiten zu erläutern, auch jederzeit während des weiteren Studienverlaufs. Sie haben jedoch das Recht, Ihre Einwilligung zur Teilnahme an der Studie jederzeit und ohne Angabe von Gründen zu widerrufen, ohne dass das Vertrauensverhältnis zu Ihrem behandelnden Arzt in irgendeiner Weise leidet. Aus Sicherheitsgründen soll jedoch auch bei vorzeitigem Studienabbruch eine abschließende Untersuchung stattfinden. Bei vorzeitigem Abbruch erhalten Sie weiterhin alle Maßnahmen, die für Ihre Krankheit angemessen sind.

Sie werden selbstverständlich auch über jegliche Informationen, die für die Teilnahme an der Studie relevant sein könnten, umgehend informiert.

Versicherungsschutz

Entsprechend dem deutschen Arzneimittelgesetz (AMG) wurde für Sie eine Patienten-Versicherung beim

Gerling-Konzern (Regionalzentrum Südwest) – Theodor-Heuss-Allee 108, 60486 Frankfurt, Tel. 06977567-466 (Police Nr. 70-005868405-2)

bis zu 3 Jahren nach der Abschlussuntersuchung nach der Chemotherapie abgeschlossen. Voraussetzung für den Versicherungsschutz ist die Einhaltung folgender Hinweise:

Sie dürfen sich einer anderen medizinischen Behandlung nur im Einvernehmen mit dem Prüfarzt unterziehen, außer im Notfall.

Falls Sie an sich eine Gesundheitsschädigung beobachten oder vermuten, die als Folge der klinischen Prüfung eingetreten sein könnte, müssen Sie dies bitte **unverzüglich** der Versicherung sowie nach Möglichkeit in Kopie dem Prüfarzt (d.h. dem Arzt, der Sie innerhalb dieser Studie betreut) und unserer Studienzentrale melden. Die Anzeigepflicht erfasst alle körperlichen Schäden, die in ursächlichem Zusammenhang mit der Anwendung des Prüfpräparates oder mit einer Maßnahme, die im Zusammenhang mit der klinischen Prüfung durchgeführt wurde, stehen können.

Siehe Teil VIII →

DEUTSCHE STUDIENGRUPPE HOCHMALIGNE NON-HODGKIN-LYMPHOME*
German High Grade Non-Hodgkin's Lymphoma Study Group

***(gefördert durch die Deutsche Krebshilfe)**

Studiensekretariat der DSHNHL<br>Prof. Dr. M. Pfreundschuh<br><br>Telefon für Notfälle:<br>06841/16-23084<br>06841/16-23000 (Pforte der Klinik)	**Patienteninformation - VIII**<br>**DSHNHL 2004-3**<br><br>**(Kopie für den Patienten)**

Unterrichtung des Hausarztes

Ihr Einverständnis vorausgesetzt, wird Ihr Hausarzt informiert, dass Sie an dieser Studie teilnehmen. Wünschen Sie, dass die (auch außerhalb einer Studie) notwendigen Blutbildkontrollen durch den Hausarzt durchgeführt werden, so wird der Hausarzt über den empfohlenen Zeitpunkt und das Ausmaß dieser Untersuchungen (Hb-Wert, Leukozyten und Thrombozyten) schriftlich durch den Prüfarzt informiert.

Studienabbruch

Ihr Prüfarzt, der Studienleiter, die unabhängige Ethikkommission an dieser Einrichtung, oder sonstige Gesundheitsbehörden können diese Studie oder Ihre Teilnahme daran jederzeit aus einem beliebigen Grund und ohne Ihr Einverständnis abbrechen.

Gründe für einen Studienabbruch können sein (Beispiele):

- *durch den Patienten* selbst, dadurch dass er sein Einverständnis zur Teilnahme an dieser Studie zurückzieht;

- *durch den Prüfarzt* (ihr behandelnder Arzt), wenn er eine weitere Teilnahme für nicht mehr vertretbar hält;

- *durch den Studienleiter*, wenn aufgrund neuer Erkenntnisse durch die regelmässig durchgeführten Zwischenprüfungen eine Weiterführung der Studie nicht mehr zu vertreten ist. In diesem Fall werden Ihnen diese Erkenntnisse mitgeteilt.

Siehe Teil IX →

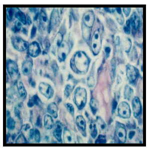

DEUTSCHE STUDIENGRUPPE HOCHMALIGNE NON-HODGKIN-LYMPHOME*
German High Grade Non-Hodgkin's Lymphoma Study Group

***(gefördert durch die Deutsche Krebshilfe)**

Studiensekretariat der DSHNHL<br>Prof. Dr. M. Pfreundschuh<br><br>Telefon für Notfälle:<br>06841/16-23084<br>06841/16-23000 (Pforte der Klinik)	**Patienteninformation - IX**<br>**DSHNHL 2004-3**<br><br>**(Kopie für den Patienten)**

Datenschutzerklärung / Vertraulichkeit der Unterlagen

Alle im Rahmen der Studie anfallenden Daten über einzelne Patienten werden an die Datenauswertung in Leipzig (Institut für Medizinische Informatik, Statistik und Epidemiologie) sowie gegebenenfalls an das Pathologieregister des Lymphomverbundprojektes zur vertraulichen Verwendung weitergegeben. Im Rahmen der Studie werden weiterhin die durch den vom behandelnden Arzt in Auftrag gegebenen pathologischen Untersuchungsbefunde sowie die entnommenen Gewebeproben an eine referenzpathologische Einrichtung weitergeleitet. Die Belange der ärztlichen Schweigepflicht und des Datenschutzes werden voll gewahrt. Auch im Fall von Veröffentlichungen in wissenschaftlichen Zeitschriften wird der Datenschutz voll umfänglich gewahrt. Alle Personen, die Einblick in die gespeicherten Daten haben, sind zur Wahrung des Datenschutzes verpflichtet. Sie können jederzeit in die über Ihre Erkrankung gespeicherten Daten Einsicht nehmen.

Die DSHNHL und das Lymphomverbundprojekt (Referenzpathologien) sind Mitglieder im "Kompetenznetz Maligne Lymphome e.V." (KML). Das KML hat ein projektübergreifendes Datenschutzkonzept entwickelt, um einerseits den Patienten einen umfassenden Schutz Ihrer persönlichen Daten zu gewährleisten und andererseits interessierten Forschern einen Zugang zu medizinischen Daten und Gewebeproben innerhalb der Studie zu ermöglichen.

Dieses Datenschutzkonzept des KML wird Ihnen separat vorgestellt, damit Sie die Möglichkeit haben, diesen Projekten getrennt von dieser Studie zuzustimmen.

14.2.2 Patienteninformation zum Datenschutzkonzept des Kompetenznetz Maligne Lymphome (KML)

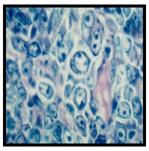

DEUTSCHE STUDIENGRUPPE HOCHMALIGNE NON-HODGKIN-LYMPHOME*
German High Grade Non-Hodgkin's Lymphoma Study Group

***(gefördert durch die Deutsche Krebshilfe)**

Studiensekretariat der DSHNHL<br>Prof. Dr. M. Pfreundschuh<br>Telefon für Notfälle:<br>06841/16-23084<br>06841/16-23000 (Pforte der Klinik)	**Patienteninformation - KML I**<br>**DSHNHL 2004-3 - Datenschutzkonzept: Kompetenznetz Maligne Lymphome**<br>Kompetenznetz Maligne Lymphome **(Kopie für den Patienten)**

Allgemeine Informationen über das Kompetenznetz Maligne Lymphome

Die Deutsche Studiengruppe Hochmaligne Non-Hodgkin Lymphome (DSHNHL), der Lymphomverbund sowie die Projektgruppe „Molekulare Mechanismen bei malignen Lymphomen“ (MMML) sind Mitglieder im Kompetenznetz Maligne Lymphome e.V. (KML e.V., Zentrale des KML, Klinikum der Universität zu Köln, Tel.: 0221/478-7400) mit Sitz in Köln (www.lymphome.de). Ziel des KML ist es, durch klinische und experimentelle Forschungsprojekte im Bereich der Therapie, Diagnostik und Pathogenese von Lymphomen neue wissenschaftliche Erkenntnisse zu gewinnen und diese möglichst schnell und effektiv in die klinische Versorgung umzusetzen. Dies schafft die Voraussetzungen für die Etablierung einer präziseren Diagnostik und einer wirksameren Therapie bei Malignen Lymphomen. Wir möchten Sie um Ihre Zustimmung bitten, dass Ihre Daten und (Gewebe-) Proben unter Wahrung des Datenschutzes in diese Tätigkeit des KML einbezogen werden dürfen.

Welche Daten und Materialien werden über Sie im KML gespeichert bzw. gelagert und wozu werden sie verwendet?

In der Patientenliste des KML werden Ihre personenidentifizierenden Daten (Name, Vorname, Geburtsdatum und Geschlecht) gespeichert. Eine weitere Liste des KML enthält so genannte Kenndaten, die angeben, an welchen Studien Sie teilnehmen bzw. teilgenommen haben oder wo im Studienzusammenhang gewonnene Materialien oder Befunde lagern (z. B. Lymphknotenmaterial, Blutproben, Röntgenbilder). Die in beiden Listen des KML gespeicherten Daten dürfen nur von Personen eingesehen werden, die durch den Vorstand des KML beauftragt wurden. Die Speicherung der Daten erfolgt gemäß den gültigen Datenschutzbestimmungen.

Die im KML erfassten Daten und Materialien (in den beiden KML-Listen sowie in den Studiengruppen und Projekten des KML) werden ausschließlich von Mitgliedern des KML für festgelegte Forschungsprojekte zum Thema „Maligne Lymphome“ zusammengeführt und ausgewertet. Die jeweiligen Projekte werden zuvor dem Vorstand des KML zur Kenntnis gebracht und durch den „Ausschuss Datenschutz“ im Einzelfall auf die Einhaltung der Datenschutzbestimmungen hin überprüft.

Die Daten und Materialien werden in Abhängigkeit von den Forschungszielen und Möglichkeiten entweder pseudonymisiert (Ihr Name wird durch eine Nummer = Pseudonym ersetzt mit der Möglichkeit, zu einem späteren Zeitpunkt, wenn nötig, auf Ihre Person zurückzuschliessen = Rückidentifizierung) oder anonymisiert (in den Daten wird der Bezug zu Ihrer Person gelöscht) an das jeweilige Forschungsprojekt weitergegeben.

Siehe Teil KML II →

DEUTSCHE STUDIENGRUPPE HOCHMALIGNE NON-HODGKIN-LYMPHOME*
German High Grade Non-Hodgkin's Lymphoma Study Group

***(gefördert durch die Deutsche Krebshilfe)**

Studiensekretariat der DSHNHL
Prof. Dr. M. Pfreundschuh

Telefon für Notfälle:
06841/16-23084
06841/16-23000 (Pforte der Klinik)

Patienteninformation - KML II
DSHNHL 2004-3 - Datenschutzkonzept:
Kompetenznetz Maligne Lymphome

Kompetenznetz Maligne Lymphome

(Kopie für den Patienten)

Der Vorstand des KML beauftragt hierfür eine Institution (Adresse und Telefonnummer können Sie über die untenstehende Kontaktadresse erfahren) mit der Pseudonymisierung bzw. Anonymisierung.

Die Vorgehensweise wurde von den zuständigen Datenschutzbeauftragten der Länder und des Bundes geprüft und zustimmend bewertet.

Rückidentifizierung

Wenn Ihre Daten pseudonymisiert gespeichert bzw. Ihre Materialien pseudonymisiert gelagert werden, hat nur die oben genannte Institution die Möglichkeit, diese Angaben/Materialien wieder Ihrer Person zuzuordnen (Rückidentifizierung). Dies erfolgt nur in bestimmten Fällen, z. B. wenn es für Sie selbst und Ihre Behandlung medizinisch notwendig oder wichtig ist.

Rücknahme der Einwilligungserklärung, Recht auf Einsicht und Berichtigung der Daten in den Listen des KML

Sie haben jederzeit das Recht, die über Sie im KML gespeicherten Informationen einzusehen. Sie können Ihre Einwilligung in die Speicherung jederzeit widerrufen und die Löschung der in den beiden KML-Listen gespeicherten Daten verlangen. Hierfür ist Ihr behandelnder Arzt *(Adresse und Tel-.Nr. müssen am Ende dieser Patienteninformation vom behandelnden Arzt ausgefüllt werden),* die Studienzentrale/ Projektzentrale (*Studiensekretariat der DSHNHL, Universitätsklinikum des Saarlandes, Innere Medizin I, Geb. 40, D-66421 Homburg /Saar, Tel. 06841/16-23084*) oder direkt das KML Ihr Ansprechpartner – ebenso wie für weitere Fragen:

Kompetenznetz Maligne Lymphome e. V.
Joseph-Stelzmann-Straße 9
50924 Köln
0221/478 – 7400
lymphome@uk-koeln.de

Benachrichtigung über Forschungsergebnisse

Falls im Verlauf der Studie oder eines Forschungsvorhabens wichtige neue Erkenntnisse im Hinblick auf die Diagnostik und Therapie Ihrer Erkrankung bekannt werden, werden Ihre Daten - Materialien und Befunde - durch die oben genannte Stelle wieder Ihrer Person zugeordnet (rückidentifiziert). Damit können Ihnen die Forschungsergebnisse unter Hinzuziehung Ihres behandelnden Arztes mitgeteilt werden. In der Patienten-/Probandeneinwilligung am Ende dieses Dokuments können Sie entscheiden, ob Sie über solche neuen Erkenntnisse informiert werden möchten.

Siehe Teil KML III →

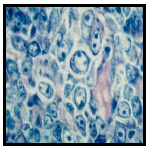

DEUTSCHE STUDIENGRUPPE HOCHMALIGNE NON-HODGKIN-LYMPHOME*
German High Grade Non-Hodgkin's Lymphoma Study Group

***(gefördert durch die Deutsche Krebshilfe)**

Studiensekretariat der DSHNHL
Prof. Dr. M. Pfreundschuh

Telefon für Notfälle:
06841/16-23084
06841/16-23000 (Pforte der Klinik)

Patienteninformation - KML III
DSHNHL 2004-3 - Datenschutzkonzept: Kompetenznetz Maligne Lymphome

Kompetenznetz Maligne Lymphome

(Kopie für den Patienten)

Autorisierte Dritte mit Zugangsberechtigung zu den Listen des KML

Aufgrund gesetzlicher Regelungen haben auch Mitarbeiter der zuständigen Überwachungsbehörden das Recht, in Ihre in den KML-Listen gespeicherten Daten Einsicht zu nehmen, um diese zu prüfen. Alle Personen, die Einblick in die gespeicherten Daten haben, sind zur Wahrung des Datenschutzes verpflichtet.

Stand dieser Patienteninformation

Diese Patienteninformation berücksichtigt alle Aspekte, die zum Zeitpunkt der Erstellung bekannt waren. Sollten sich wesentliche Änderungen ergeben, die Ihnen als Teilnehmer an Studien/Projekten des KML zur Kenntnis gebracht werden sollen, wird Sie Ihr Arzt umgehend darüber informieren.

14.2.3 Patienteninformation – Daten des aufklärenden Arztes

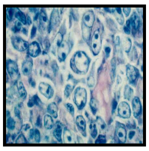

DEUTSCHE STUDIENGRUPPE HOCHMALIGNE NON-HODGKIN-LYMPHOME*
German High Grade Non-Hodgkin's Lymphoma Study Group

***(gefördert durch die Deutsche Krebshilfe)**

Studiensekretariat der DSHNHL<br>Prof. Dr. M. Pfreundschuh<br>Telefon für Notfälle:<br>06841/16-23084<br>06841/16-23000 (Pforte der Klinik)	**Patienteninformation**<br>**DSHNHL 2004-3**<br>**Aufklärender Arzt**<br>**(Kopie für den Patienten)**
Behandelnder Arzt	
Name:	
Vorname:	
Praxis / Einrichtung:	
Strasse + Nr.	
PLZ + Ort:	
Telefon:	
Fax:	
Stempel der Praxis / Einrichtung:	
Wir danken für Ihre Mitarbeit<br><br>...<br>Datum und Unterschrift des aufklärenden Arztes	

14.3 Protokoll der Patientenaufklärung

DEUTSCHE STUDIENGRUPPE HOCHMALIGNE NON-HODGKIN-LYMPHOME*
German High Grade Non-Hodgkin's Lymphoma Study Group

***(gefördert durch die Deutsche Krebshilfe)**

**-Zum Verbleib-**	**Protokoll der Patientenaufklärung<br>(Prof. Dr. M. Pfreundschuh)<br>DSHNHL 2004-3**

Name des Patienten:

Nachname: Vorname: Geb.-Dat.: ______

T T M M J J J J

Das Aufklärungsgespräch erstreckt sich auf:

1. Art der Erkrankung
2. Prognose der Erkrankung bei protokollgerechter Therapie
3. Stand der Wissenschaft, Ziele der Studie
4. Wirkungen und Nebenwirkungen
   Wirkungen: Möglichkeit der vollständigen Rückbildung aller Krankheitszeichen und Heilung. Nebenwirkungen: Nach Chemotherapie: Übelkeit und Erbrechen; Unterdrückung der Blutbildung mit Verminderung der Blutzellen, dadurch bedingtes Risiko der Infektion, Blutung und Anämie; Haarausfall; Nervenschädigung; Schädigung des Herzmuskels; Gewebeschädigung bei falscher Injektion der Chemotherapie; Blasenschädigung; allergische Reaktionen. Kortikoidnebenwirkungen. Nach Strahlentherapie: Übelkeit, Brechreiz, Schluckbeschwerden, Kopfschmerzen, Müdigkeit, Leukozytopenie (Abfall der weißen Blutkörperchen mit erhöhter Gefährdung durch Infekte), Anämie (Blutarmut), Hautveränderungen und Haarausfall im Strahlengebiet; möglich sind außerdem Strahlenreaktionen des Lungengewebes (Luftnot), des Darms (Durchfälle), des Herzbeutels (Erguß). Nach Gabe des Wachstumsfaktors der Blutbildung (G-CSF): Knochenschmerzen, Temperaturerhöhung. Nach Gabe des Rituximab-Antikörpers: Fieber, Schüttelfrost, Muskel- und Gelenkschmerzen, Blutdruckabfall, Hautausschläge, selten schwere allergische Nebenwirkungen bis hin zum Schock.
5. Sichere Antikonzeption unter Therapie und bis 12 Monate danach für weibliche u. männliche Patienten
6. Weitergabe von Daten (Datenschutz) inklusive aller angegliederter Projekte
   Der Patient ist damit einverstanden, dass seine im Rahmen der klinischen Studie erhobenen Krankheits-/Studiendaten aufgezeichnet und zur Auswertung durch die Studiengruppe sowie zur Überprüfung an die Überwachungsbehörde oder zuständige Bundesoberbehörde weitergegeben werden. Er ist ferner damit einverstanden, dass von der Studiengruppe oder den vorgenannten Behörden beauftragte Personen seine Original-Krankenakten sowie die Original-Studiendaten einsehen und personenbezogene Daten und Informationen an Dritte zum Zweck der wissenschaftlichen Auswertung der Studie weitergegeben werden. Der Patient ist damit einverstanden, dass die Studienzentrale Kontakt mit ihm aufnimmt, wenn über die Dauer eines Jahres keine Informationen über seinen Krankheitsverlauf eingehen.
7. Entscheidungsfreiheit des Patienten
   Der Patient kann frei über die Teilnahme an der Studie entscheiden.
8. Patientenentscheidung:: ○ Teilnahme ○ keine Teilnahme

Arzt:

....	....	....
Funktion	Datum	Unterschrift

Patient:

....	....	....
	Datum	Unterschrift

14.4 Patienten-Einverständniserklärung

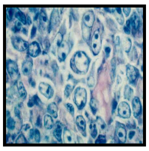

DEUTSCHE STUDIENGRUPPE HOCHMALIGNE NON-HODGKIN-LYMPHOME*
German High Grade Non-Hodgkin's Lymphoma Study Group

***(gefördert durch die Deutsche Krebshilfe)**

An das<br>Studiensekretariat der DSHNHL<br>Prof. Dr. M. Pfreundschuh<br>Med. Klinik und Poliklinik, Innere Medizin I<br>Universität des Saarlandes<br>66421 HOMBURG/SAAR<br>Tel: 06841/16-23084 Fax: 06841/16-23004	**Patienten-Einverständniserklärung – I**<br>**DSHNHL 2004-3**<br><br>**(Original für den Arzt)**<br>**(Kopie für den Patienten)**<br>**(Kopie für das Studiensekretariat)**

Ich, Nachname: -- Vorname: --

geboren am: --

wurde von den behandelnden Ärzten in einem ausführlichen Gespräch darüber aufgeklärt, dass ich an einem hochmalignen Non-Hodgkin-Lymphom erkrankt bin, einer bösartigen Erkrankung des lymphatischen Systems, die durch eine Behandlung mit Zytostatika (Zellgiften) + Immuntherapie, ggf. zusammen mit einer Strahlentherapie in vielen Fällen geheilt werden kann. Die Standardtherapie für mein Stadium eines aggressiven Lymphoms sind 6 Zyklen CHOP-Chemotherapie in dreiwöchigen Abständen mit 6 Applikationen des monoklonalen Antikörpers Rituximab (6 Zyklen R-CHOP-21). Ich wurde darüber informiert, dass zur Zeit eine Studie durchgeführt wird, bei der versucht wird, die Wirksamkeit einer Chemotherapie mit 6 Zyklen R-CHOP-21 durch eine Verkürzung der Zyklusdauer zu verbessern. Die Verkürzung der Zyklusdauer von 21 auf 14 Tage bietet die theoretische Möglichkeit, dass die Wirksamkeit einer solchen Therapie erhöht ist. Allerdings kann nicht ausgeschlossen werden, dass es zu stärkeren Nebenwirkungen kommt. Alle Medikamente sind für den Einsatz bei Non-Hodkgin-Lymphomen vom Bundesgesundheitsamt zugelassen.

Über das Ziel der Studie bin ich informiert und erkläre mich in Kenntnis dieser Information freiwillig bereit, an der Studie teilzunehmen.

Ich bin mit der im Rahmen dieser Studie erfolgenden Aufzeichnung von Krankheits-/Studiendaten und ihrer anonymisierten Weitergabe zur Überprüfung an die zuständige Überwachungsbehörde oder Bundesoberbehörde einverstanden. Darüber hinaus bin ich damit einverstanden, dass zum Zwecke der Dokumentation Einsicht in meine Krankenakte genommen wird und dass meine personenbezogenen, für die wissenschaftliche Überwachung des Krankheitsverlaufes oder zur Dokumentation notwendigen Daten in nicht anonymisierter Form gesammelt und ausgewertet werden von zur Verschwiegenheit verpflichteten Beauftragten der Studiengruppe oder der Behörden.

Ich habe Anspruch auf Information über Ziel, Zweck und Verbleib dieser Datensammlung. Die Verarbeitung dieser Daten (Speicherung, Übermittlung, Veränderung, Löschung) in der Studienzentrale dient der medizinischen Dokumentation von Therapie und Nachsorge im Rahmen der wissenschaftlichen Zusammenarbeit mehrerer Kliniken. Alle Personen, die Zugang zu den Daten haben, sind zur Wahrung des Datengeheimnisses verpflichtet Publikationen erfolgen nur anonymisiert.

Ich bin mit der Weitergabe meiner personenbezogenen Daten an den von mir benannten Hausarzt und die in die Behandlung einbezogenen Ärzte/Ärztinnen einverstanden**.**

Siehe Teil II →

DEUTSCHE STUDIENGRUPPE HOCHMALIGNE NON-HODGKIN-LYMPHOME*
German High Grade Non-Hodgkin's Lymphoma Study Group

***(gefördert durch die Deutsche Krebshilfe)**

An das
Studiensekretariat der DSHNHL
Prof. Dr. M. Pfreundschuh
Med. Klinik und Poliklinik, Innere Medizin I
Universität des Saarlandes
66421 HOMBURG/SAAR
Tel: 06841/16-23084 Fax: 06841/16-23004

Patienten-Einverständniserklärung – II
DSHNHL 2004-3

(Original für den Arzt)
(Kopie für den Patienten)
(Kopie für das Studiensekretariat)

Ich verpflichte mich, alle Gesundheitsstörungen, die im Verlauf der Therapie oder später auftreten und mit der Therapie in Zusammenhang stehen könnten (z.B. späte Veränderungen des Blutbildes), **unverzüglich** der Versicherung sowie nach Möglichkeit in Kopie dem Prüfarzt (d.h. dem Arzt, der Sie innerhalb dieser Studie betreut) und unserer Studienzentrale.zu melden. Die Anzeigepflicht erfasst alle körperlichen Schäden, die in ursächlichem Zusammenhang mit der Anwendung des Prüfpräparates oder mit einer Maßnahme, die im Zusammenhang mit der klinischen Prüfung durchgeführt wurde, stehen können.

Meine Teilnahme an der Studie erfolgt freiwillig und ich kann meine Teilnahme an der Studie jederzeit ohne Angabe von Gründen widerrufen. Ich erhalte bei Verweigerung der Studienteilnahme die Standardtherapie (6 Zyklen R-CHOP-21) oder kann eine Therapie frei wählen. Ich bin außerdem damit einverstanden, dass die mir entnommene Gewebsprobe einem besonders qualifizierten Pathologen (Referenzpathologen) zur Überprüfung der Diagnose zugeschickt wird und diese Daten zum Zwecke wissenschaftlicher Auswertungen gespeichert werden.

Ich bin weiterhin damit einverstanden, dass Teile der zu diagnostischen Zwecken entnommenen Blut-, Knochenmark- und Gewebeproben zu wissenschaftlichen, nicht-kommerziellen Untersuchungen zur Ätiologie, Diagnose und Therapie der Lymphome verwendet werden und übertrage hiermit der Studienleitung der DSHNHL das Verfügungsrecht an diesem Material. Das Eigentumsrecht an den von mir entnommenen Gewebeproben verbleibt bei mir. Keinesfalls werden zusätzliche Gewebs- oder Blutuntersuchungen über das aus medizinischer Indikation notwendige Maß hinaus durchgeführt.

Weiterhin bin ich damit einverstanden, dass im Zusammenhang mit den Gewebeproben personenbezogene Daten im Pathologieregister des Lymphomverbundes gespeichert werden.

Ich wurde darüber aufgeklärt, dass regelmäßige Kontrolluntersuchungen in meinem Interesse, aber besonders auch im Interesse zukünftiger Patienten, über viele Jahre hinweg durchgeführt werden sollen. Die Ergebnisse dieser Nachuntersuchungen werden der Studienzentrale gemeldet.

Die Aufklärung über die Teilnahme an der Studie war mir in allen Punkten verständlich. Ich hatte ausreichend Gelegenheit, die Einzelheiten der Behandlung, ihre Ziele und Nebenwirkungen mit den behandelnden Ärzten zu diskutieren. Ich stimme in Kenntnis dieser Information den geplanten wie auch weiteren, sich durch die Umstände ergebenden und für die Behandlung notwendigen Maßnahmen zu. Ich bestätige, dass mir eine Kopie der Patienteninformation und Einwilligungserklärung sowie eine Kopie der Patientenversicherung ausgehändigt wurde.

Ich erkläre mich damit einverstanden, dass ich nach Ablauf eines Jahres ohne Kontrolluntersuchung von der Studienzentrale selbst angeschrieben werden kann. Diese kann mich zu einer Nachuntersuchung durch einen Arzt meiner Wahl auffordern.

Siehe Teil III →

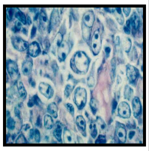

DEUTSCHE STUDIENGRUPPE HOCHMALIGNE NON-HODGKIN-LYMPHOME*
German High Grade Non-Hodgkin's Lymphoma Study Group

***(gefördert durch die Deutsche Krebshilfe)**

An das
Studiensekretariat der DSHNHL
Prof. Dr. M. Pfreundschuh
Med. Klinik und Poliklinik, Innere Medizin I
Universität des Saarlandes
66421 HOMBURG/SAAR
Tel: 06841/16-23084 Fax: 06841/16-23004

Patienten-Einverständniserklärung – III
DSHNHL 2004-3

(Original für den Arzt)
(Kopie für den Patienten)
(Kopie für das Studiensekretariat)

Datenspeicherung im Kompetenznetz Maligne Lymphome

In der Patientenliste des KML werden meine persönlichen Daten (Name, Vorname, Geb.-Datum) gespeichert Die Kenndatenliste des KML enthält Verweise, anhand derer erkennbar ist, in welcher Institution weitere Daten von mir gespeichert bzw. wo Materialien von mir gelagert sind. Mit Hilfe der in den beiden Listen des KML gespeicherten Information kann das KML die in den Studienzentralen oder Forschungsprojekten entsprechend der Patienten-information gespeicherten Daten (Studien-, Bilddaten) sowie Materialien (z. B. Blutproben, Gewebeproben) pseudonymisiert zusammenführen. Diese können im Rahmen von Forschungsprojekten des KML zum Thema „Maligne Lymphome" untersucht und ausgewertet werden. Mit der Verarbeitung meiner Daten und Nutzung der Materialien nach der beschriebenen Art und Weise erkläre ich mich hiermit einverstanden.

Im Rahmen der Qualitätssicherung wird die Diagnose durch eine Referenzbegutachtung überprüft. Die Übermittlung dieser Referenzdiagnose von den Experten der Referenzeinrichtung an die Studienzentrale erfolgt elektronisch über die in den KML-Listen gespeicherten Daten (Identifikationsdaten und Kenndaten). Mit der Verarbeitung meiner Daten zum Zwecke der Referenzdiagnostik erkläre ich mich einverstanden. Auch nach Abschluss der Studie bzw. in/nach der Nachbehandlungsphase kann ich über neue Erkenntnisse im Hinblick auf meine Erkrankung unter Hinzuziehung meines behandelnden Arztes oder des Studienarztes bzw. deren Vertreter informiert werden. Mit einer Kontaktaufnahme erkläre ich mich hiermit einverstanden. Darüber hinaus kann ich von Personen des KML e. V. kontaktiert werden, um zu einem späteren Zeitpunkt an Projekten oder Studien des KML teilzunehmen. Mit einer Kontaktaufnahme für spätere Projekte erkläre ich mich hiermit einverstanden.
Ich kann jederzeit auch ohne Angabe von Gründen meine Einwilligung zur Teilnahme und Verarbeitung meiner Daten in den Listen des KML für Forschungszwecke innerhalb des KML zurückziehen, ohne dass mir dadurch Nachteile entstehen. Meine in den Forschungsvorhaben des KML analysierten Daten werden anonymisiert, d. h. der Bezug zu meiner Person wird gelöscht.

Hiermit erteile ich mein Einverständnis für:

die Teilnahme an der Studie DSHNHL 2004-3 (UNFOLDER):	○ **einverstanden**	○ **nicht einverstanden**
die Weitergabe meiner Daten zu Forschungszwecken innerhalb des KML:	○ **einverstanden**	○ **nicht einverstanden**

Siehe Teil IV →

DEUTSCHE STUDIENGRUPPE HOCHMALIGNE NON-HODGKIN-LYMPHOME*
German High Grade Non-Hodgkin's Lymphoma Study Group

***(gefördert durch die Deutsche Krebshilfe)**

An das
Studiensekretariat der DSHNHL
Prof. Dr. M. Pfreundschuh
Med. Klinik und Poliklinik, Innere Medizin I
Universität des Saarlandes
66421 HOMBURG/SAAR
Tel: 06841/16-23084 Fax: 06841/16-23004

Patienten-Einverständniserklärung – IV
DSHNHL 2004-3

(Original für den Arzt)
(Kopie für den Patienten)
(Kopie für das Studiensekretariat)

Arzt: Funktion: ------------------------------

Datum: ---------------- Unterschrift: ------------------------------
T T / M M / J J J J

Stempel der Praxis / Einrichtung:

Zeuge: ------------------------------

Datum: ---------------- Unterschrift: ------------------------------
T T / M M / J J J J

Patient:

Name: ------------------------------ Vorname: ------------------------------
------------------------------(Bitte in Druckschrift)------------------------------

Geb.-Dat.: ----------------
T T / M M / J J J J

Adresse des Patienten:

Strasse: ------------------------------

PLZ: ---------------- Ort: ------------------------------

Datum: ---------------- Unterschrift: ------------------------------
T T / M M / J J J J

14.5 Liste der teilnehmenden Zentren

Ort	Plz	KID	Zentrum/Praxis		Strasse	H.Nr
Aachen	52070	30	Hämatologische Praxis	Dr. Weinberg, Dr. Tummes, Fr. Dr. Guggenberger	Adalbertsteinweg	12
Amberg	92224	314	Klinikum St. Marien Amberg	Med. Klinik II/ Hämat.-Onkologie	Mariahilfbergweg	7
Ansbach	91522	357	Gemeinschaftspraxis	Dres. Müller/ Hahn	Endresstr.	14
Aschaffenburg	63739	285	Klinikum Aschaffenburg	Med. Klinik II	Am Hasenkopf	
Aschaffenburg	63739	327	Schwerpunktpraxis für Onkologie	Fr. Dr. Klausmann	Weissenburger Str.	30
Augsburg	86150	350	Praxis für Hämatologie-Onkologie	Dr. Slawik	Prinzregentenstr.	1
Augsburg	86150	333	Gemeinschaftspraxis	Dr. Brudler, Dr. Heinrich	Halderstraße	29
Augsburg	86156	42	Zentralklinikum Augsburg	II. Medizin. Klinik/ Hämat.-Onkologie	Stenglinstr.	2
Aurich	26603	153	Kreiskrankenhaus Aurich	Innere Med./ Hämatologie	Wallinghausenerstr.	12
Bad Friedrichshall	74177	308	Kreiskrankenhaus Am Plattenwald	Hämat./ Internist. Onkologie	Am Plattenwald	1
Bad Hersfeld	36251	287	Kreiskrankenhaus Bad Hersfeld	Medizinische Klinik (Hämat.-Onkologie)	Seilerstr.	29
Bad Saarow	15526	319	Humaine - Klinikum Bad Saarow/ Fürstenwalde	Innere Medizin	Pieskower Str.	33
Bad Säckingen	79704	476	Kreiskrankenhaus Bad Säckingen	Krankenhäuser und Pflegeheime des Landkreises Waldshut		
Bad Salzuflen	32105	379	Hämatologie und Intern. Onkologie	Dr. Weinert	Grabenstr.	12
Bad Soden/ TS.	65812	424	Hämatologie/ Internistische Onkologie		Kronberger Str.	38
Bad Urach	72574	419	Ermstalklinik Bad Urach	Innere Abteilung	Stuttgarter Str.	100
Bayreuth	95445	275	Krankenhaus Bayreuth Hohe Warte	Med. Klinik	Hohe Warte	8
Berlin	13357	349	Gemeinschaftspraxis	Dr. Ihle, Dr. Blau	Badstr.	57
Berlin	12200	405	Universitätsklinikum Benjamin Franklin	Medizinische Klinik III	Hindenburgdamm	30
Berlin	13125	133	Universitätsklinikum Charité	Robert Rössle Klinik/ Abt. Med. Onkologie	Lindenberger Weg	80
Berlin	13347	392	Dr. med Frank Strohbach/ Dr. med. Christoph Mayr	Hämatologie und Internistische Onkologie	Seestraße	64
Berlin	10249	423	Praxis Herr Dr. med. H. Lebahn	Internist	Matthiasstr.	7
Berlin	12351	471	Vivantes - Klinikum Neukölln	Klinik f. Innere Medizin, Hämatologie u. Onkologie	Rudower Str.	48
Berlin	10559	456	Facharztin für Innere Medizin, Hämatologie und Internistische Onkologie		Rathenowerstr.	5

14.5 Liste der teilnehmenden Zentren

DSHNHL 2004-3 Version 4.0 vom 22.11.2012 3

Ort	Plz	KID	Zentrum/Praxis		Strasse	H.Nr
Berlin	13589	457	Ev. Waldkrankenhaus Spandau		Stadtrandstr.	555
Berlin	12559	447	DRK Kliniken Berlin Köpenick	Med. Klinik II, Schwerpunkt Gastroenterologie	Salvador-Allende-Str.	2-8
Berlin	10117	468	Campus Charite Mitte	Medizinische Klinik mit Schwerpunkt Onkologie und Hämatologie	Schumannstr.	20/21
Berlin	14195	394	Fachärzte für Innere Medizin	Hämatologie und Internistische Onkologie	Clayallee	225 a
Bernburg	06406	324	Klinikum Bernburg	Innere Klinik	Kustrenaner Str.	98
Bielefeld	33615	366	Franziskus-Hospital	Med. Klinik II	Kiskerstr.	26
Bietigheim	74319	297	Krankenhaus Bietigheim	Innere Medizin I	Riedstr.	12
Bocholt	46397	116	St.-Agnes-Hospital Bocholt	II. Med. Abteilung	Barloer Weg	125
Bochum	44791	354	St. Josef-Hospital	Medizin. Klinik I	Gudrunstr.	56
Bochum	44791	494	Augusta-Kranken-Anstalt gGmbH		Bergstr.	26
Bochum	44892	278	Universitätsklinik Bochum	Knappschaftskrankenhaus/ Innere Medizin	In der Schornau	23-25
Bonn	53111	90	Med. Univ. Poliklinik Bonn		Wilhelmstr.	35-37
Bonn	53105	364	Universitätsklinikum Bonn	Innere Med. I, Hämatologie/Onkologie	Siegmund-Freud-Str.	25
Bonn	53113	489	Johanniter Krankenhaus	Innere Abteilung		
Borken	46325	465	Hämatologie und Internistische Onkologie		Propst-Sievert-Weg	9
Bottrop	46242	368	Knappschaftskrankenhaus	Innere Abteilung	Osterfelder Str.	157
Braunschweig	38114	474	Städt. Klinikum Braunschweig	Medizinische Klinik III	Celler Straße	38
Bremen	28239	232	Evang. Diakonie-Krankenhaus gGmbH	Medizinische Klinik/ Hämat. u. Onkologie	Gröpelinger Heerstr.	406
Bremerhaven	27574	411	Zentralkrankenhaus Reinkenheide	Medizinische Klinik 1	Postbrookstraße	103
Bremerhaven	27568	491	St. Josepf-Hospital Bremerhaven	Medizinische Klinik mit SchwerpunktHämatologie/intern. Onkologie	Wiener Straße	1
Bünde	32257	336	Lukaskrankenhaus	Hämat. u. Intern. Onkologie	Hindenburgstr.	56
Burgwedel	30938	426	Facharzt für Innere Medizin	Hämatologie und intern. Onkologie	Hannoversche-Str.	2
Celle	29223	322	Allgemeines Krankenhaus	Klinik für Gastroenterologie	Siemensplatz	4
Celle	29221	480	Dr. Felix Marquard/Praxis	FA für Innere Medizin, Hämatologie u. intern. Onkologie	Neumarkt	1d
Chemnitz	09113	141	Krankenhaus Küchwald Chemnitz	Klinik für Innere Medizin	Bürgerstr.	2
Coesfeld	48653	462	Hämatologie - Internistische Onkologie	Dr. med. M. Glados, Facharzt für Innere Medizin	Südring	15
Cottbus	03048	204	Carl-Thiem-Klinikum Cottbus	Hämatologie u. Internist. Onkologie	Thiemstr.	111
Darmstadt	64283	271	Städtische Kliniken Darmstadt	Med. Klinik V/ Hämat.-Onkol.	Grafenstr.	9

DSHNHL 2004-3 Version 4.0 vom 22.11.2012

Ort	Plz	KID	Zentrum/Praxis		Strasse	H.Nr
Dessau	06822	374	Städtisches Klinikum	Abt. Hämatologie-Onkologie	Auenweg	38
Dortmund	44137	107	St. Johannes-Hospital Dortmund	Med. Klinik II	Johannesstr.	9-17
Dresden	01307	289	Universitätsklinikum Dresden	Med. Klinik I	Fetscherstr.	74
Duisburg	47166	22	St. Johannes-Hospital Duisburg	Medizinische Klinik II	An Der Abtei	7-11
Duisburg	47228	472	Johanniter-Krankenhaus Rheinhausen	Medizinische Klinik II	Kreuzacker	1-7
Dülmen	48249	298	Franz-Hospital Dülmen	Hämat. u. Internist. Onkologie	Vollenstr.	10
Düsseldorf	40489	317	Diakonie Kaiserswerth , FNK	Innere Medizin	Kreuzbergstr.	79
Düsseldorf	40225	399	Universitätsklinikum Düsseldorf	Klinik für Onkologie und Klin. Immunologie	Moorenstr.	5
Eberswalde	16225	473	Klinikum Barnim GmbH	Werner Forßmann Krankenhaus	Rudolf-Breitscheid-Str.	100
Emden	26721	291	Hans-Susemihl-Krankenhaus Emden	Med. Klinik I	Bolardusstr.	20
Erfurt	99089	356	Klinikum Erfurt GmbH	Innere Klinik	Nordhäuser Straße	74
Eschweiler	52249	72	St.-Antonius-Hospital Eschweiler	Abt. f. Hämatologie/ Onkologie	Dechant-Deckers-Str.	8
Essen	45147	47	Universitätsklinikum Essen	Hämatologische Abteilung	Hufelandstr.	55
Essen-Werden	45239	53	Ev. Krankenhaus Essen-Werden		Pattbergstr.	1-3
Esslingen a. N	73730	440	Städtische Kliniken	Medizinische Klinik	Hirschlandstr.	97
Frankfurt am Main	60488	151	Krankenhaus Nordwest Frankfurt	II. Med. Klinik, Hämatologie/ Onkologie	Steinbacher Hohl	2-26
Frankfurt/ M.	60389	428	Gemeinschaftspraxis	Hämatologie u. Internistische Onkologie	Im Prüfling	17-19
Frankfurt/ Main	60590	369	Klinikum d. Johann-Wolfgang-Goethe-Univ.	Medizinische Klinik III	Theodor-Stern-Kai	7
Frankfurt/ Main	60318	410	St. Marienkrankenhaus	Katharina-Kasper-Kliniken	Richard-Wagner-Str.	14
Frankfurt/Oder	15236	221	Klinikum Frankfurt (Oder)	Innere/ Hämatologie-Onkologie	Müllroser Chaussee	7
Freiburg	79110	156	Evang. Diakoniekrankenhaus Freiburg	Innere Abteilung M1b	Wirthstr.	11
Freiburg	79098	422	Gemeinschaftspraxis Drs. med. Reiber/ Semsek	Fachärzte f. Innere Medizin/ Hämatol./ Onkologie	Schreiberstr.	20
Freiburg	79106	415	Universitätsklinikum Freiburg	Hämatologie/ Onkologie, Abt. Innere Med. I	Hugstetter Str.	55
Fulda	36043	347	Städt. Klinikum Fulda	III. Medizinische Klinik	Pacelliallee	4
Garmisch-Partenkirchen	82467	395	Klinikum Garmisch-Partenkirchen	Zentrum für Innere Med., Hämatologie/ Onkologie	Auenstr.	6
Gehrden	30989	339	Robert-Koch-Krankenhaus	Med. Klinik	Von-Redon-Str.	1
Gelsenkirchen	45899	449	St. Josef-Hospital Gelsenkirchen	Abt. f. Onkologie u. Hämatologie	Rudolf-Bertram-Platz	1
Germering	82110	425	PD Dr. med. Dr. med. habil. J. Mittermüller	Internist-Hämatologie-Onkologie	Schillerstr.	15

DSHNHL 2004-3 Version 4.0 vom 22.11.2012 3

Ort	Plz	KID	Zentrum/Praxis		Strasse	H.Nr
Gießen	35392	235	Zentrum für Innere Medizin Gießen	Med. Klinik I + II	Klinikstr.	36
Goch	47574	421	Wilhelm-Anton-Hospital	Medizinische Klinik I	Vossheider Str.	214
Görlitz	02828	493	Städt. Klinikum Görlitz GmbH	Medizinische Klinik	Girbigsdorfer STr.	1-3
Goslar	38642	450	Harzkliniken Goslar	Med. Klinik II, Onkologie u. Hämatologie	Kösliner Str.	12
Göttingen	37075	61	Georg-August-Universität Göttingen	Zentrum f. Innere Med./ Abt. Hämat.-Onk.	Robert-Koch-Str.	40
Grefrath	47929	486	Dr. med. R. Thomas	Praxis	Hinsbecker Str.	5a
Greifswald	17487	150	Ernst-Moritz-Arndt-Universität	Abt. f. Hämatologie u. Onkologie	Sauerbruchstr.	
Gummersbach	51643	436	Krankenhaus Gummersbach GmbH	Medizinische Klinik	Wilhelm-Breckow-Allee	20
Güstrow	18273	418	Gemeinschaftspraxis Dr. S. Duda/ Dr. Eschenburg	Fachärzte für Innere Medizin	Am Wall	1
Gütersloh	33332	233	Städtisches Krankenhaus Gütersloh	Med. Klinik	Reckenbergerstr.	19
Hagen	58095	106	St.-Josefs-/St.-Marien-Hospital Hagen	Hämatologie und Onkologie	Bergstr.	56
Hagen	58095	409	Allgemeines Krankenhaus - Strahlentherapie		Grünstr.	35
Hagen	58095	276	Allgemeines Krankenhaus Hagen	Medizinische Klinik	Grünstr.	35
Halberstadt	38820	382	St. Salvator-Krankenhaus	Medizinische Klinik	Gleimstr.	5
Halle	06120	304	Städt. Krankenhaus Martha-Maria Halle	Klinik f. Innere Med. II	Röntgenstraße	12
Halle	06120	306	Universitätsklinikum Halle-Wittenberg	Innere Medizin IV	Ernst-Grube-Str.	40
Halle/ Saale	06110	359	St. Elisabeth/ St. Barbara-Krankenhaus	Medizin. Klinik II	Mauerstr.	5
Halle (Saale)	06110	387	Gemeinschaftspraxis	PD Dr. med. Rohrberg/ Dr. med. Hurtz	Niemeyerstr.	23
Haltern	45721	484	St. Sixtus-Hospital		Gartenstraße	2
Hamburg	22765	11	Praxis Prof. Kleeberg		Max-Brauer-Allee	52
Hamburg	20251	58	Universitätskrankenhaus Eppendorf	Abt. Hämatologie/ Onkologie	Martinistr.	20
Hamburg	20095	293	Gemeinschaftspraxis	Dr. Zeller, Dr. Veerpoort	Am Ballindamm	3
Hamburg	22763	229	Allgemeines Krankenhaus Hamburg Altona	II. Medizin. Abteilung/ Onkol. u. Hämat.	Paul-Ehrlich-Str.	1
Hamburg	22417	363	Klinikum Nord, Heidberg	Abteilung für Innere Medizin	Tangstedter Landstr.	400
Hamburg	22087	390	Marienkrankenhaus	1. Medizinische Abteilg.	Alfredstraße	9
Hamburg	21029	492	Praxis für Innere Medizin		Glindersweg	80
Hamburg	20099	401	Allgemeines KH St. Georg	Hämatologische Abteilung	Lohmühlenstr.	5
Hamm	59063	301	Evangelisches Krankenhaus Hamm	Innere Medizin/ Hämat.-Onkologie	Werler Str.	110

DSHNHL 2004-3 Version 4.0 vom 22.11.2012 3

Ort	Plz	KID	Zentrum/Praxis		Strasse	H.Nr
Hamm	59071	417	St. Marien-Hospital Hamm	Klinik für Hämatologie	Knappenstr.	19
Hanau	63450	413	Klinikum Stadt Hanau	Med. Klinik II, Abt. Hämatologie/ Onkologie	Leimenstraße	20
Hannover	30559	320	Henriettenstift	Med. Klinik II	Schwemannstraße	17
Hannover	30449	299	Klinikum Hannover - Siloah	Med. Klinik III f. Hämatologie u. Onkologie	Roesebeckstr.	15
Hannover	30169	34	Gemeinschaftspraxis	Dr. Gaede, Dr. Wysk	Calenberger Esplanade	1
Hannover	30623	1	Medizinische Hochschule Hannover	Abt. Hämatologie/ Onkologie	Carl-Neuberg-Str.	1
Harrislee	24955	325	Onkologische Schwerpunktpraxis	Dr. med. W. Grimm; Fr. Dr. med. Japsen-Schiemann	Am Hang	1
Heidelberg	69120	70	Ruprecht-Karls-Universität Heidelberg	Med. Klinik, Abt. Innere Medizin V, Hämatologie/Onkologie/ Rheumatologie	Im Neuenheimer Feld	410
Heidelberg	69126	71	Thoraxklinik Heidelberg	Internistisch-Onkologische Abteilung	Amalienstr.	5
Heidenheim	89522	147	Kreiskrankenhaus Heidenheim	Innere Abt.	Schloßhaustr.	100
Herford	32049	46	Klinikum Kreis Herford	Medizin. Klinik II	Schwarzenmoorstr.	70
Herne	44625	290	Marienhospital Herne	Universitätsklinik, Medizin. Klinik I	Hoelkeskampring	40
Herrsching	82211	348	Privatklinik Dr. med. R. Schindlbeck	Onkolog.-Hämat. Praxis Dr. med. Dietzfelbinger	Seestraße	43
Hildesheim	31134	332	Onkologische Schwerpunktpraxis		Bernwardstr.	6
Hildesheim	31134	427	St. Bernward Krankenhaus	Med. Klinik II	Treibestr.	9
Hildesheim	31134	402	Städt. Krankenhaus Hildesheim	Med. Klinik II	Weinberg	1
Holzminden	37603	340	Evang. Krankenhaus	Hämat. / Onkologie	Forster Weg	34
Homburg	66421	113	Universitätsklinikum des Saarlandes	Innere Medizin I	Kirrberger Straße	
Hoyerswerda	02977	328	Klinikum Hoyerswerda	1. Med. Klinik	M.-Grollmuß-Str.	10
Idar-Oberstein	55743	400	KMT-Klinik		Dr.-Ottmar-Köhler Str.	2
Jena	07743	300	Gemeinschaftspraxis	Fr. Dr. med. Hahnfeld, Dr. med. Krombholz	Ernst - Abbe - Str.	3
Jena	07747	210	Klinikum der Friedrich-Schiller-Uni.	Klinik f. Innere Medizin - Onkologie	Erlanger Allee	101
Kaiserslautern	67655	73	Westpfalz-Klinikum GmbH	Standort I Kaiserslauterrn	Hellmut-Hartert-Str.	1
Kaiserslautern	67655	482	Schwerpunktpraxis	Hämatologie und Onkologie	Stiftsplatz	5
Karlsruhe	76133	59	Städtisches Klinikum Karlsruhe	Med. Klinik II/ Hämatologie	Moltkestr.	80
Karlsruhe	76137	381	St.-Vincentius-Krankenhäuser	II. Med. Abt./ Hämatologie, Onkologie	Südendstr.	32
Kassel	34121	309	Rotes Kreuz Krankenhaus Kassel	Akademisches Lehrkrankenhaus	Hansteinstr.	29
Kassel	34117	454	Gemeinschaftspraxis	Fachärzte für Medizin, Hämatologie und internistische Onkologie	Jordanstr.	6

Ort	Plz	KID	Zentrum/Praxis		Strasse	H.Nr
Kempten (Allgäu)	87439	398	Klinikum Kempten-Oberallgäu gGmbH	Innere Medizin I Hämatologie und Onkologie	Memminger-Str.	50-52
Kiel	24116	10	Städtisches Krankenhaus Kiel	II. Medizinische- u. Poliklinik	Chemnitzstr.	33
Koblenz	56068	99	Evang. Stift St. Martin gGmbH Koblenz	Klinik für Innere Medizin	Johannes-Müller-Str.	7
Koblenz	56068	295	Gemeinschaftspraxis	Dr. Köppler, Dr. Heymanns, PD Dr. Weide	Neversstr.	5
Köln	50677	313	Gemeinschaftspraxis für Hämat. & Intern. Onkologie	Dr. med. Schmitz, Dr. med. Steinmetz	Sachsenring	69
Köln	50937	24	Universitätsklinik Köln	Klinik I für Innere Medizin	Kerpener Straße	62
Köln	51067	464	Krankenhaus Holweide		Neufelderstr.	32
Köln-Kalk	51105	312	Onkologische Fachpraxis Köln	Dr. Mainka	Rolshoferstr.	4-6
Köln-Merheim	51109	326	Lungenklinik Köln-Merheim		Ostmerheimer Str.	200
Kornwestheim	70806	343	Leonardis- Klinik	Innere Medizin	Albstr.	9
Krefeld	47805	50	Städt. Krankenanstalten Krefeld	Medizinische Klinik II	Lutherplatz	40
Krefeld	47798	460	Dr. med. Michael Neise	Internist/ Hämatologie/ Internistische Onkologie	Marktstr.	186188
Kronach	96317	270	Kreiskrankenhaus Kronach	Station 13	Friesener Str.	41
Kronach	96317	420	Gemeinschaftspraxis Dr. Stauch/ Dr. Scheib		Niederbronner Str.	2
Landshut	84038	458	Dr. med. Ursula Vehling-Kaiser	Internistin, Hämatologie und Internistische Onkologie, Sportmedizin	Heilig-Geist-Gasse	411
Landshut	84034	453	Klinikum Landshut	Med. Klinik I	Robert-Koch-Str.	1
Lebach	66822	146	Caritas-Krankenhaus Lebach	Innere Abt./ Hämat.-Onkologie	Heeresstr.	
Leer	26789	389	Kreiskrankenhaus Leer	Innere Medizin	Annenstr.	9
Leer	26788	452	Schwerpunktpraxis f. Hämatologie u. Intern. Onkologie	Dr. med. L. Müller	Annenstr.	11
Leipzig	04129	362	Städtisches Klinikum St. Georg	Abt. für Internistische Onkologie/ Hämatologie	Delitzscher Str.	141
Leipzig	04103	205	Universität Leipzig	Med. Klinik u. Poliklinik/ Hämat.-Onkol.	Philip-Rosenthal-Straße	23-25
Leipzig	04289	442	Park-Krankenhaus Leipzig-Südost GmbH	Klinik für Innere Medizin/ Gastroenterologie, Onkologie	Strümpellstr.	41
Leipzig	04179	386	Onkolog. Praxis am Diakonissenhaus	Innere Medizin	Georg-Schwarz-Str.	53
Leipzig	04289	344	Gemeinschaftspraxis	Dr. med. Aldaoud, Dr. med. Schwarzer	Strümpellstr.	41
Lemgo	32655	125	Klinikum Lippe-Lemgo GmbH	II. Med. Klinik	Rintelner Str.	85
Limburg	65549	292	St. Vincenz-Krankenhaus Limburg	Abt. Hämatologie und Onkologie	Auf dem Schafsberg	
Lingen	49808	288	St. Bonifatius Hospital Lingen	Innere Medizin	Wilhelmstr.	13
Lippstadt	59555	345	Dreifaltigkeitshospital	Hämatologie-Onkologie	Klosterstr.	31

Ort	Plz	KID	Zentrum/Praxis		Strasse	H.Nr
Lörrach	79539	378	Kreiskrankenhaus Lörrach	Hämat. und internist. Onkologie	Spitalstr.	25
Lörrach	79539	490	Praxis Dr. med. Jan Knoblich		Sanser Platz	2
Lübeck	23538	45	Universitätsklinikum Schleswig-Holstein	Med. Klinik I - Hämatologie/ Onkologie	Ratzeburger Allee	160
Lübeck	23562	485	Schwerpunktpraxis für Hämatologie/Onkologie	Gemeinschaftspraxis	Osterweide	10
Lübeck	23560	397	Sana Kliniken Lübeck GmbH, Krankenhaus Süd	Klinik für Hämatologie u. Onkologie	Kronsforder Allee	71-73
Lüdenscheid	58515	459	Märkische Kliniken GmbH	Klinikum Lüdenscheid	Paulmannshöher Str.	14
Ludwigsburg	71640	281	Klinikum Ludwigsburg	Med. Klinik II	Posilipostr.	4
Ludwigshafen	67063	243	Klinikum der Stadt Ludwigshafen	Medizinische Klinik A	Bremserstr.	79
Ludwigshafen	67067	122	St. Marienkrankenhaus Ludwigshafen	Med. Klinik	Salzburger Str.	15
Magdeburg	39120	268	Medizinische Akademie Magdeburg	Klinik f. Innere Medizin/ Hämatologie	Leipziger Str.	44
Magdeburg	39002	129	Krankenhaus Altstadt Magdeburg	Klinik für Hämatologie/ Onkologie	Max-Otten-Str.	11-15
Mainz	55101	355	Klinikum der Universität	III. Medizinische Klinik und Poliklinik	Langenbeckstr.	1
Mainz	55131	466	Kath. Klinikum/ St. Vicenz- II. Elisabeth-Hospital		An der Goldgrube	11
Mannheim	68305	23	Klinikum der Stadt Mannheim	III. Med. Klinik	Wiesbadener Str.	7-11
Marburg	35057	358	Schwerpunktpraxis Internist. Onkologie	Dr. med. Weidenbach	Erlenring	19
Marburg	35043	12	Universitätsklinikum Marburg	Abteilung für Hämatologie	Baldingerstr./Lahneberg e	
Mayen	56727	241	Kreiskrankenhaus Mayen	Innere Abteilung	Siegfriedstr.	20
Minden	32427	323	Gemeinschaftspraxis	Dr. Becker, Fr. Dr. Kreisel-Büstgens	Königstr.	69
Minden/Westf.	32423	144	Klinikum Minden	Abt. Hämatologie/ Onkologie	Portastr.	7-9
Mönchengladbach	41063	145	Krankenhaus Maria-Hilf II Franziskushaus	Innere Medizin I	Viersener Strasse	450
Mönchengladbach	41061	120	Ev. Krankenhaus Bethesda Mönchengladbach	Med. Klinik	Ludwig-Weber-Str.	15
Mönchengladbach/ Rheydt	41239	331	Hämatologisch-Onkologische Praxis	Dr. Grabenhorst	Wildstr.	11
Mülheim	45473	412	PD Dr. J. Schröder	Hämatologe/ Onkologe	Aktienstr.	277
Mülheim/Ruhr	45466	318	Ev. Krankenhaus	Med. Klinik	Wertgasse	30
Mülheim/Ruhr	45468	282	St.-Marien-Hospital Mühlheim/Ruhr	1. Medizin. Klinik	Kaiserstraße	50
Münchberg	95213	94	Kreiskrankenhaus Münchberg	Innere Medizin	Hoferstr.	40
München	80336	338	Klinikum der Universität München Innenstadt	Medizinische Klinik	Pettenkoferstr.	8a
München	80335	330	Onkolog. Gemeinschaftspraxis	PD Tigges, Dres. Böning/ Abenhardt/ Bosse	Prielmeyerstr.	1

Ort	Plz	KID	Zentrum/Praxis		Strasse	H.Nr
München	81377	337	Klinikum Großhadern	Med. Klinik 3	Marchioninistr.	15
München	81675	372	Klinikum rechts der Isar	III. Med. Klinik u. Poliklinik	Ismaninger Str.	22
München	81545	370	Städt. Krankenhaus München-Harlaching	Interdisziplinäres Tumorzentrum	Sanatoriumsplatz	2
München	80804	65	Städt. Krankenhaus München-Schwabing	I. Med. Abteilung	Kölner Platz	1
München	81737	296	Städt. Krankenhaus München	Krankenhaus Neuperlach	Oskar Maria Graf Ring	51
Münster	48149	43	Universitätsklinikum Münster	Innere Medizin A	Albert-Schweitzer-Str.	33
Münster	48149	380	Onkologische Schwerpunktpraxis	Gemeinschaftspraxis	Steinfurter Str.	60b
Naunhof	04683	487	Praxis Dr. med. Jens Uhlig	Facharzt für Innere Medizin	Leipziger Straße	9
Nettetal	41334	376	Städtisches Krankenhaus Nettetal	Innere Medizin	Sassenfelder Kirchweg	1
Neubrandenburg	17022	384	Dietrich-Bonhoeffer-Klinikum Neubrandenburg	Klinik für Innere Medizin		
Neumarkt	92318	269	Kreiskrankenhaus Neumarkt	Hämatologie u. Internistische Onkologie	Nürnberger Str.	12
Neumarkt	92318	434	Onkologische Schwerpunktpraxis	Dr. med. E. Ladda	Bahnhofstr.	4
Neunkirchen	66538	262	Krankenhaus Neunkirchen gGmbH	Abt. Innere Medizin	Brunnenstraße	20
Neunkirchen	66538	496	Onkologische Schwerpunktpraxis		Hebbelstr.	2
Neunkirchen	66538	431	Dr. med. Peter Schmidt	Hämatologie/ Onkologie	Hebbelstr.	2
Neuss	41464	27	Lukaskrankenhaus Neuss	Med. Klinik II	Preußenstr.	84
Neuss	41460	407	Facharzt für Innere Medizin	Schwerpunkt Hämatologie u. Internistische Onkologie	Hammer Landstraße	1a
Neuwied	56564	408	DRK-Krankenhaus Neuwied	I. Medizinische Abteilung	Marktstraße	
Norderstedt	22844	367	Praxis für Hämat.- internist. Onkologie	Dr. R. Hoffmann	Langenharmerweg	19
Northeim	37154	393	Gemeinschaftspraxis für Hämatologie	Dr. med. S. Detken	Sturmbäume	8-10
Nürnberg	90419	137	Klinikum Nürnberg/ Med. Klinik 5	Klinikum Nord/ Hämat.-Onkologie	Prof.-Ernst-Nathan-Str.	1
Oberstaufen	87534	483	Schloßbergklinik Oberstaufen	Innere - Hämatologie	Schloßstr.	27-29
Oberstaufen	87534	495	Humaine Schloßbergklinik		Schloßstr.	27-29
Offenbach/ M.	63065	403	Gemeinschaftspraxis, Hämatologie u. Intern. Onkologie	Dres. med. H. E. Balló/ H.-P. Böck	Marktplatz	11
Offenburg	77654	246	Kreiskrankenhaus Offenburg	Med. Klinik II	Ebertplatz	12
Oldenburg	26121	346	Gemeinschaftspraxis für Hämat. u. Onkol.	Dres. B. Otremba, D. Reschke	Auguststr.	22
Oldenburg	26122	7	Ev. Krankenhaus Oldenburg	Medizin. Klinik/ Hämatolog. Abt.	Steinweg	13-17
Oldenburg	26133	230	Städtische Kliniken Oldenburg	Innere Med., Hämatologie/Onkologie	Dr.-Eden-Str.	10

Ort	Plz	KID	Zentrum/Praxis		Strasse	H.Nr
Olpe	57462	461	St. Martinus Hospital	Medizinische Klinik	Hospitalweg	6
Osnabrück	49076	311	Paracelsus-Klinik Osnabrück	Hämat. u. Internistische Onkologie	Am Natruper Holz	69
Osnabrück	49074	406	Marienhospital Osnabrück	Innere Medizin	Johannisfreiheit	2-4
Parchim	19370	477	Asklepios Klinik Parchim			
Pforzheim	75175	342	Städtisches Klinikum	Med. Klinik - Station M 3	Kanzlerstr.	2-6
Pforzheim	75179	478	Krankenhaus Siloah	Klinik für Innere Medizin	Wilferdinger Straße	67
Pinneberg	25421	467	Dr. med. Gerold Baake	Facharzt für Innere Medizin, Hämatologie und Internistische Onkologie	Fahltskamp	74
Potsdam	14467	218	Klinikum Ernst von Bergmann -Potsdam-	Abt. für Internistische Onkologie	Charlottenstr.	72
Radebeul	01445	310	Kreiskrankenhaus Radebeul	Innere Klinik	Heinrich-Zille-Str.	13
Ravensburg	88212	273	St. Elisabethen-Krankenhaus Ravensburg	Innere Abteilung	Elisabethenstr.	15
Recklinghausen	45661	414	Elisabeth-Krankenhaus GmbH	Med. Klinik f. Onkologie + Hämatologie	Röntgenstr.	10
Regensburg	93049	341	Krankenhaus der Barmherzigen Brüder	Klinik für intern. Onkologie u. Hämat.	Prüfeninger Str.	86
Regensburg	93047	334	Praxis für Hämatologie/ Onkologie	Dr. Dengler	Bahnhofstr.	24
Regensburg	93042	132	Klinikum der Universität Regensburg	Hämatologie/ Onkologie	Franz-Josef-Strauß-Allee	11
Rehling	86508	396	Hämatologe-Internistischer Onkolog. Praxis	FA für Innere Medizin	Bergstr.	9a
Remscheid	42859	439	Sana-Klinikum Remscheid GmbH	Med. Klinik I	Burger Str.	211
Rheine	48431	280	Jakobi-Krankenhaus Rheine	Innere Abteilung	Hörstkamp	12
Rostock	18057	352	Praxis für Hämatologie/ Onkologie	Dr. Lakner	Wismarsche Str.	32
Rostock	18059	224	Klinikum Rostock Südstadt	Innere Medizin	Südring	81
Rostock	18055	223	Universität Rostock	Innere Medizin/ Hämatologie	Ernst-Heydemann-Str.	6
Rüsselsheim	65428	373	Gemeinschaftspraxis	Dres. W. Würmel, M. Baldus	August-Bebel-Str.	52
Saarbrücken	66113	353	Gemeinschaftspraxis Hämat. und Intern. Onkologie	Dr. Schimke, Dr. Jacobs	Am Ludwigsberg	78
Saarbrücken	66113	64	Caritasklinik St. Theresia Saarbrücken	Klinik für Hämatologie und Onkologie	Rheinstr.	2
Saarlouis	66713	360	St. Elisabeth-Krankenhaus	Onkologie/ Strahlentherapie	Kapuzinerstr.	4
Schleswig	24837	130	Martin-Luther-Krankenhaus Schleswig	Innere Abteilung	Lutherstr.	12
Schwäbisch Hall	74523	321	Diakoniekrankenhaus	Innere Medizin	Diakonistr.	10
Schweinfurt	97422	267	Leopoldina-Krankenhaus Schweinfurt	Med.-Klinik II	Gustav Adolf Str.	8
Schweinfurt	97421	437	Gemeinschaftspraxis Hämatologie/ Internist	Dr. med. R. von Hirschhausen	Schultesstr.	13-15

DSHNHL 2004-3 Version 4.0 vom 22.11.2012 3

Ort	Plz	KID	Zentrum/Praxis		Strasse	H.Nr
Schwelm	58332	432	Helios-Klinikum	Medizinische Klinik	Dr.-Möller-Str.	15
Schwerin	19049	385	Klinikum Schwerin	Klinik f. Innere Medizin ll	Wismarsche Str.	397
Siegen	57074	316	Evang. Jung-Stilling-Krankenhaus	Medizin. Klinik/ Innere	Wicherstraße	40
Siegen	57072	371	St. Marien-Krankenhaus	Hämatologie und Onkologie	Kampenstr.	51
Solingen	42697	135	St.-Lukas-Klinik Solingen		Schwanenstr.	131
Stadtlohn	48703	438	Krankenhaus Maria-Hilf	Hämatologie u. Int. Onkologie	Vredenerstr.	58
Straubing	94315	404	Dr. med. Matthias Dermandt	Internist-Hämatologie und Internistische Onkologie	Bahnhofsplatz	1
Stuttgart	70174	279	Katharinenhospital Stuttgart	Klinik für Onkologie	Kriegsbergstr.	60
Stuttgart	70173	377	Onkologische Schwerpunktpraxis	Dr. med. G. Springer/ Dr. med. H. Fiechtner	Königstr.	1 B
Stuttgart	70176	388	Gemeinschaftspraxis	Dr. med. E. Höring/ Dr. med. M. von Ehr	Schloßstr.	100
Stuttgart	70376	63	Robert-Bosch-Krankenhaus Stuttgart	Abt. Hämatologie/ Onkologie	Auerbachstr.	110
Stuttgart	70176	96	Diakonissenkrankenhaus Stuttgart	Innere Medizin II/ I	Rosenbergstr.	38
Stuttgart	70374	115	Krankenhaus Bad Cannstatt	Med. Klinik	Prießnitzweg	24
Stuttgart	70191	105	Bürgerhospital Stuttgart	Med. Klinik I	Tunzhofer Str.	14-16
Thuine	49832	283	St. Elisabeth-Krankenhaus Thuine	Med.Klinik	Klosterstr.	4
Traunstein	83278	277	Klinikum Traunstein	Innere Abteilung	Cuno-Niggel-Str.	3
Trier	54290	52	KA Mutterhaus der Borromäerinnen	Med. 1/ Hämat.- Onkologie	Feldstr.	16
Trier	54290	383	Gemeinschaftspraxis	Dres. Strauß/ Rendenbach	Kutzbachstr.	7
Trier	54290	152	Praxis Fr. Dr. Grundheber		Friedrich-Wilhelm-Str./ Ecke Gilbertstr.	23
Trier	54292	39	Krankenhaus der Barmherzigen Brüder	1. Med. Abteilung	Nordallee	1
Troisdorf	53840	416	Dr. med. Helmut Forstbauer	Internist. Hämatologie/ Onkologie	Schloßstr.	18
Tutzing	82327	351	Krankenhaus	Innere Abteilung	Bahnhofstr.	5
Ulm	89081	17	Med. Universitätsklinik Ulm	Innere Medizin III	Robert-Koch-Str.	8
Unna	59403	307	Ev. Krankenhaus Unna	Innere Abteilung I	Hohlbeinstr.	10
Vechta	49377	335	St. Marienhospital	Hämatologie u. Internistische Onkologie	Marienstr.	6-8
Villingen-Schwenningen	78050	361	Klinikum der Stadt	Villingen-Schwenningen GmbH	Vöhrenbacher Str.	23
Waldbröl	51545	265	Kreiskrankenhaus Waldbröl	Hämat.-Onkologie	Dr. Goldenbogen Str.	
Weiden	92637	470	Hämatologie und Int. Onkologie	Internist	Friedrich-Ebert-Str.	12a

DSHNHL 2004-3 Version 4.0 vom 22.11.2012 3

Ort	Plz	KID	Zentrum/Praxis		Strasse	H.Nr
Wendlingen	73240	365	Praxis für Onkologie	Dr. Kamp	Weberstr.	16
Westerland	25980	488	Asklepios Nordseeklinik	Hämatologie und Onkologie	Norderstraße	81
Wetzlar	35578	274	Krankenhaus Wetzlar	Med.Klinik II	Forsthausstr.	
Wiesbaden	65191	329	Deutsche Klinik für DiagnostiK/Onkologie	Praxis für Hämat. und Onkologie	Aukammallee	33
Wiesbaden	65199	89	Dr.-Horst-Schmidt-Kliniken Wiesbaden	Hämatologie/ Onkologie	Ludwig-Erhard-Str.	100
Witten	58455	286	Ev. Krankenhaus Witten-Herdecke		Pferdebachstr.	27
Wuppertal	42283	237	Kliniken St. Antonius Wuppertal	Abt Hämatologie/Onkologie	Carnaper Str.	48
Wuppertal	42283	375	Helios Klinikum Wuppertal	Medizinische Klinik 1/ Hämat.-Onkologie	Heusnerstr.	40
Würzburg	97070	249	Universität Würzburg	Medizinische Poliklinik	Klinikstr.	6 - 8
Zwickau	08009	211	Heinrich-Braun-KH/ Städtisches Klinikum Zwickau	Klinik f. Innere Medizin	Karl-Keil-Str.	35
Stand Juli 2005						

DSHNHL 2004-3 Version 4.0 vom 22.11.2012 3

14.6 Adressen der Mitglieder des Protokollkomitee und des DSMK

Name	Institution	Adresse
Prof. Dr. M. Bentz	Städtisches Klinikum Karlsruhe<br>Medizinische Klinik II	Moltkestr. 90<br>76133 Karlsruhe
Prof. Dr. G. Brittinger	Universitätsklinikum Essen<br>Zentrum für Innere Medizin	Hufelandstr. 55<br>45147 Essen
Prof. Dr. G. Borchmann	Klinik I für Innere Medizin<br>Hämato-Onkol. Ambulanz	Kerpener Str. 62<br>50937 Köln
PD Dr. A. Buck	Klinikum rechts d. Isar	Ismaninger Str. 22<br>81675 München
Prof. Dr. A. Bücker	Universitätsklinikum des Saarlandes<br>Klinik für Radiologie	66421 Homburg
Prof. Dr. V. Diehl	Universitätsklinik Köln<br>Klinik I für Innere Medizin	Joseph-Stelzmannstr. 9<br>50924 Köln
PD Dr. M. Dreyling	LMU München<br>Klinikum Großhadern<br>Medizinische Klinik 3	Marchioninistr. 15<br>81377 München
Dr. M. Engelhard	Universitätsklinikum Essen<br>Strahlentherapie	Hufelandstr. 55<br>45147 Essen
PD Dr. N. Frickhofen	Zentrum Innere Medizin,<br>Innere Medizin III<br>Hämatologie/Onkologie<br>Dr. Horst-Schmidt-Kliniken GmbH	Ludwig-Erhard-Str. 100<br>65199 Wiesbaden
Prof. Dr. B. Glass	Allgemeines Krankenhaus St. Georg<br>Abteilung Hämatologie	Lohmühlenstraße 5<br>20099 Hamburg
PD Dr. M. Hänel	Klinikum Chemnitz gGmbH<br>Krankenhaus Küchwald<br>Klini f. Innere Med. III	Bürgerstr. 2<br>09009 Chemnitz
Prof. Dr. F. Hartmann	Klinikum Lippe-Lemgo<br>Medizinische Klinik II	Rintelnerstr. 85<br>32657 Lemgo
Prof. Dr. K. Havemann	Universitätsklinikum Marburg<br>Abteilung für Hämatologie	Baldingerstr./ Lahneberge<br>35043 Marburg
Prof. Dr. R. Herrmann	Abteilung für Onkologie<br>Kantonsspital	CH 4023 Basel
PD Dr. G. Held	Universitätsklinikum d. Saarlandes<br>Med. Klinik u. Poliklinik<br>Innere Med. I	66421 Homburg
Prof. Dr. D. Hellwig	Universitätsklinikum d. Saarlandes<br>Klinik für Nuklearmedizin	66421 Homburg
Prof. Dr. U. Kaiser	II. Medizinische Klinik<br>St. Bernward Krankenhaus	Treibestr.9<br>31132 Hildesheim
Prof. Dr. C.-M. Kirsch	Universitätsklinikum d. Saarlandes<br>Klinik für Nuklearmedizin	66421 Homburg
Prof. Dr. W. Knauf	Gemeinschaftspraxis<br>Dres.Klippstein/Grunewald/<br>Tesch/Knauf	Im Prüfling 17-19<br>60389 Frankfurt/Main

Name	Institution	Adresse
Dr. P. Koch	Med. Klinik A der<br>Universitätsklinik Münster	Albert-Schweitzer-Str. 33<br>48149 Münster
PD Dr. E. Lengfelder	Universitätsmedizin Mannheim<br>III. Med. Universitätsklinik	Theodor-Kutzer-Ufer 1-3<br>68167 Mannheim
Dr. R. Liersch	Universitätsklinikum Münster<br>Med. Klinik u. Poliklinik A<br>Hämatologie/Onkologie	Albert-Schweitzer-Str. 33<br>48129 Münster
Dr. W. Lindemann	Kath. Krankenhaus Hagen<br>St.-Josefs/St.-Marien-Hospital	Bergstr. 56<br>58095 Hagen
Prof. Dr. M. Löffler	Institut für Medizinische<br>Informatik, Statistik und<br>Epidemiologie	Härtelstr. 16-18<br>04107 Leipzig
Dr. B. Metzner	Klinikum Oldenburg<br>Abt. Hämatologie/Onkologie	Rahel-Straus-Str. 10<br>26133 Oldenburg
Dr. M. Nickelsen	Allgemeines Krankenhaus St.<br>Georg<br>Abteilung Hämatologie	Lohmühlenstraße 5<br>20099 Hamburg
Dr. N. Peter	Carl-Thiem-Klinikum Cottbus<br>Med. Klinik II<br>Hämatologie/Onkologie	Thiemstr. 111<br>03048 Cottbus
Prof. Dr. M. Pfreundschuh	Universitätsklinikum des<br>Saarlandes<br>Medizinische Klinik und Poliklinik<br>Innere Medizin I	Kirrberger Str.<br>66424 Homburg
Viola Pöschel	Universitätsklinikum des<br>Saarlandes<br>Med. Klinik u. Poliklinik<br>Innere Med. I	66421 Homburg
Prof. Dr. A. Rosenwald	Universität Würzburg<br>Institut f. Pathologie	Josef-Schneider-Str. 2<br>97080 Würzburg
Dr. Ch. Rudolph	DRK Kliniken Berlin Köpenick<br>Medizinische Klinik II<br>Schwerpunkt Gastroenterologie	Salvator-Allende-Straße 2<br>12559 Berlin
Prof. Dr. Ch. Rübe	Klinik für Strahlentherapie und<br>Radioonkologie der<br>Radiologischen Universitätsklinik<br>Gebäude 49	66421 Homburg/Saar
Prof. Dr. H. Schmidberger	Universitätsklinikum Mainz<br>Klinik und Poliklinik für<br>Radioonkologie	Langenbeckstr.1<br>55131 Mainz
PD Dr. R. Schmits	Praxis für Onkologie	Am Ludwigsberg 86<br>66113 Saarbrücken
Prof. Dr. N. Schmitz	Allgemeines Krankenhaus St.<br>Georg<br>Abteilung Hämatologie	Lohmühlenstraße 5<br>20099 Hamburg
Prof. Dr. J. Schubert	Evangelisches Krankenhaus<br>Med. Klinik	Werler Str. 110<br>59063 Hamm
PD Dr. S. Stilgenbauer	Universitätsklinikum Ulm<br>Innere Med. III	Albert-Einstein-Allee 23<br>89081 Ulm

**Name**	**Institution**	**Adresse**
Prof. Dr. L. Trümper	Zentrum Innere Medizin<br>Abt. Hämatologie/Onkologie<br>Universitätsklinik Göttingen	Robert-Koch-Str. 40<br>37075 Göttingen
PD Dr. U. Wedding	Universitätsklinikum Jena<br>Klinik u. Poliklinik für<br>Innere Med. II	07740 Jena
PD Dr. M. Witzens-Harig	Ruprecht-Karls-Universität<br>Heidelberg<br>Med. Klinik<br>Abt. Innere Medizin	Im Neuenheimer Feld 410<br>69120 Heidelberg
Prof. Dr. G. Wulf	Zentrum Innere Medizin<br>Abt. Hämatologie/Onkologie<br>Universitätsklinik Göttingen	Robert-Koch-Str. 40<br>37075 Göttingen
Dr. rer.-nat. S. Zeynalova	Institut für Med. Informatik,<br>Statistik u. Epidemiologie	Härtelstr. 16-18<br>04107 Leipzig
Dr. rer.-nat. M. Ziepert	Institut für Med. Informatik,<br>Statistik u. Epidemiologie	Härtelstr. 16-18<br>04107 Leipzig

14.7 Voten der Ethikkommission

DSHNHL HOM
08. Okt. 2004

Ethik-Kommission

Ärztekammer des Saarlandes

Ärztekammer des Saarlandes · Postfach 10 02 62 · 66002 Saarbrücken

Herrn Professor
Dr. med. F. Hartmann
DSHNHL - Studiensekretariat Homburg
Medizinische Klinik I
Universitätsklinikum des Saarlandes

66421 Homburg

Körperschaft
des öffentlichen Rechts

Faktoreistraße 4
66111 Saarbrücken

Telefon (06 81) 4 00 30
Telefon-Durchwahl (06 81) 40 03- 378
Telefax (06 81) 4 00 33 40
E-Mail: info-aeks@aeksaar.de
Internet: www.aerztekammer-saarland.de

Kernarbeitszeit:
Mo. bis Do. 8.30 bis 11.30 Uhr
13.30 bis 15.15 Uhr
Fr. 8.30 bis 12.00 Uhr

Unser Zeichen:	Ihr Schreiben vom:	Ihr Zeichen:	Datum:
Dr. Ertz/Gn			02. Okt. 2004

Randomisierte Studie zum Vergleich einer Immuntherapie mit 6 Zyklen des monoklonalen anti-CD20-Antikörpers Rituximab in Kombination mit 6 Zyklen einer Chemotherapie mit CHOEP in 21-tägigen oder 14-tägigen Intervallen jeweils mit oder ohne Strahlentherapie von großen Tumormassen bei Patienten mit aggressiven CD20-positiven B-Zell-Lymphomen im Alter von 18-60 Jahren mit altersadaptiertem IPI =1 (alle) oder IPI =0 mit großen Tumormassen (Durchmesser ≥ 7,5 cm) – DSHNHL 2004-3 (UNFOLDER 21/14)
Unsere Kenn-Nr. 139/04

Sehr geehrter Herr Professor Hartmann!

Die Kommission hat sich in ihrer Sitzung am 30. September 2004 mit Ihrem o. g. Antrag befasst und kam zu folgender Entscheidung.
Nachdem Sie mit Schreiben vom 24.09.2004 auch die letzten Auflagen aus unserem Brief vom 31.08.2004 befriedigend beantwortet haben, sieht die Kommission bezüglich Ihres Prüfvorhabens keine berufsrechtlichen und berufsethischen Bedenken.

Sie erteilt Ihnen damit ein uneingeschränkt positives Votum.

Unter Bezugnahme auf § 2 des Statuts der Ethik-Kommission bei der Ärztekammer des Saarlandes bestehen gegen die Durchführung des beabsichtigten Forschungsvorhabens keine Bedenken.

Wir machen darauf aufmerksam, dass die Ethik-Kommission mit ihrer Stellungnahme lediglich eine Hilfestellung bei der Beurteilung ethischer und rechtlicher Gesichtspunkte eines geplanten Forschungsvorhabens gibt. Verantwortlich für die Planung und Durchführung bleibt der zuständige ärztliche Leiter des Forschungsvorhabens.

- 1 -

Die Ethik-Kommission bei der Ärztekammer des Saarlandes ist unter Beachtung der internationalen Richtlinien der ICH, GCP tätig, nach Landesrecht (Saarländisches Heilberufekammergesetz, § 5 Abs. 1) anerkannt und beim Bundesinstitut für Arzneimittel und Medizinprodukte gem. § 17 Abs. 7 des Medizinproduktegesetzes sowie beim Bundesamt für Strahlenschutz nach § 92 der Strahlenschutzverordnung und nach § 28g der Röntgenverordnung registriert.

Commerzbank Saarbrücken
Kto.-Nr. 53 89 200
BLZ 590 400 00

Dt. Apotheker- und Ärztebank Saarbrücken
Kto.-Nr. 0 001 926 209
BLZ 590 906 26

Postbank Saarbrücken
Kto.-Nr. 95 15 666
BLZ 590 100 66

Bank 1 Saar Saarbrücken
Kto.-Nr. 157 5007
BLZ 591 900 00

- 2 -

Bei Änderungen des Forschungsvorhabens vor oder während der Durchführung bedarf es nochmals eines entsprechenden Antrages vor der Änderung.

Wir machen ausdrücklich darauf aufmerksam, dass bei erkennbarer Trendwende mit negativem Ergebnis das Forschungsvorhaben abzubrechen ist.

Wir bitten, dem Probanden ein vollständiges Exemplar der Versicherungsbedingungen einschl. der Anschrift der Versicherung zur Verfügung zu stellen.

Wir wünschen Ihnen für die vorgesehene Aufgabe viel Erfolg und wären Ihnen zur gegebenen Zeit für die Übersendung eines Abschlußberichtes dankbar.

Mit freundlichen Grüßen

San.-Rat Dr. Ertz
stellv. Vorsitzender

22. 08. 2005

Ärztekammer
des Saarlandes
Körperschaft
des öffentlichen Rechts

Ethik-Kommission
Geschäftsstelle

Faktoreistraße 4
66111 Saarbrücken
Telefon-Durchwahl (06 81) 40 03-378
Telefax (06 81) 40 03-340
E-Mail: andrea.goergen@aeksaar.de
Internet: www.aerztekammer-saarland.de

Ärztekammer des Saarlandes · Postfach 10 02 62 · 66002 Saarbrücken
Ethik-Kommission

Herrn Professor
Dr. med. M. Pfreundschuh
DSHNHL – Studiensekretariat Homburg
Innere Medizin I
Universitätsklinikum des Saarlandes
66421 Homburg

Unsere Kenn-Nr.:	Ihr Schreiben vom:	Ihr Zeichen:	Datum:
139/04	06.07.2005		18. AUG. 2005

DSHNHL 2004-3 (Unfolder)
Unsere Kenn-Nr. 139/04

Sehr geehrter Herr Professor Pfreundschuh!

Wir kommen zurück auf das o.a. Schreiben und teilen Ihnen nach Prüfung der Unterlagen

- *Überarbeitetes Studienprotokoll „Unfolder", Version 3.2 vom 27.06.2005*
- *Graphik CHOEP vs. CHOP und R-CHOEP vs. R-CHOP*

mit, dass unsererseits keine Bedenken gegen Amendment Nr. 1 zum Studienprotokoll bestehen.

Mit freundlichen Grüßen

San.-Rat Dr. Ertz
stellv. Vorsitzender

Die Ethik-Kommission bei der Ärztekammer des Saarlandes ist unter Beachtung der internationalen Richtlinien der ICH, GCP-V und der 12. Novelle AMG tätig, nach Landesrecht (Saarländisches Heilberufekammergesetz, § 5 Abs. 1) anerkannt und beim Bundesinstitut für Arzneimittel und Medizinprodukte gem. § 17 Abs. 7 des Medizinproduktegesetzes sowie beim Bundesamt für Strahlenschutz nach § 92 der Strahlenschutzverordnung und nach § 28g der Röntgenverordnung registriert.

Commerzbank Saarbrücken
Kto.-Nr. 53 89 200
BLZ 590 400 00

Dt. Apotheker- und Ärztebank Saarbrücken
Kto.-Nr. 0 001 926 209
BLZ 590 906 26

Postbank Saarbrücken
Kto.-Nr. 95 15 666
BLZ 590 100 66

Bank 1 Saar Saarbrücken
Kto.-Nr. 157 5007
BLZ 591 900 00

Der Vorsitzende

Ärztekammer
des Saarlandes
Körperschaft
des öffentlichen Rechts

Ärztekammer des Saarlandes · Postfach 10 02 62 · 66002 Saarbrücken
Ethik-Kommission

Studiensekretariat der DSHNHL
Herrn Professor Dr. med. M. Pfreundschuh
Medizinische Klinik und Poliklinik
Herrn Professor Dr. med. J. Schubert
Innere Medizin I
Universitätsklinikum des Saarlandes
66421 Homburg

Ethik-Kommission
Geschäftsstelle

Faktoreistraße 4
66111 Saarbrücken
Telefon-Durchwahl (06 81) 40 03-378
Telefax (06 81) 40 03-340
E-Mail: andrea.goergen@aeksaar.de
Internet: www.aerztekammer-saarland.de

Unsere Kenn-Nr.:	Ihr Schreiben vom:	Ihr Zeichen:	Datum:
Prof. Schie./Gn			02. März 2006

Randomisierte Studie zum Vergleich einer Immuntherapie mit 6 Zyklen des monoklonalen anti-CD20-Antikörpers Rituximab in Kombination mit 6 Zyklen einer Chemotherapie mit CHOEP in 21-tägigen oder 14-tägigen Intervallen jeweils mit oder ohne Strahlentherapie von großen Tumormassen bei Patienten mit aggressiven CD20-positiven B-Zell-Lymphomen im Alter vom 18-60 Jahren mit altersadaptiertem IPI = 1 (alle) oder IPI = 0 mit großen Tumormassen (Druchmesser ≥ 7,5 cm) – DSHNHL 2004-3
Unsere Kenn-Nr. 139/04 ***(In jedem Schriftwechsel angeben!)***
Jetzige Vorlage: Unfolder-Änderungen der Protokoll-Version 3.2.2 (06.02.06) zu der Version 3.2.1 (07.10.05)

Sehr geehrter Herr Pfreundschuh,
sehr geehrter Herr Schubert!

Sie hatten auf Anregung einiger regionaler Ethik-Kommissionen Änderungen im Prüfprotokoll Version 3.2.1 vorgenommen und diese Änderungen in einer Übersicht dargestellt und in das Prüfprotokoll markiert eingearbeitet.

Die Nutzen-Risiko-Konstellation hat sich durch die vorgenommenen Änderungen nicht verschoben, das Primärvotum vom 02. Oktober 2004 gilt unverändert, es bestehen keine berufsrechtlichen und ethischen Bedenken.

Mit freundlichen Grüßen

Ihr H. Schieffer

San.-Rat Prof. Dr. Schieffer

Der Vorsitzende

Ärztekammer
des Saarlandes

Körperschaft
des öffentlichen Rechts

Ethik-Kommission
Geschäftsstelle

Faktoreistraße 4
66111 Saarbrücken
Telefon Durchwahl (06 81) 40 03 - 378
Telefax (06 81) 40 03 - 394
E-Mail: ethikkommission@aeksaar.de
Internet: www.aerztekammer-saarland.de

Ärztekammer des Saarlandes · Postfach 10 02 62 · 66002 Saarbrücken
Ethik-Kommission

Herrn Professor
Dr. med. M. Pfreundschuh
Direktor der Klinik für Innere Medizin I
Deutsche Studiengruppe
Hochmaligne Non-Hodgkin-Lymphome
Universitätsklinikum des Saarlandes
Kirrberger Straße
66421 Homburg

Unser Zeichen:	Ihr Schreiben vom:	Ihr Zeichen:	Datum:
Ha139/04	16.01.2013		25. Feb. 2013

DSHNHL 2004-3 (Unfolder)

Unsere Kenn-Nr. 139/04 ***bitte stets angeben!)***

Hier: Amendment 2

Sehr geehrter Herr Kollege Pfreundschuh,

Wir bestätigen den Erhalt Ihres o. a. Schreibens mit folgenden Dokumenten:

- *UNFOLDER Studienprotokoll (Version 4.0 v. 22.11.12)*

Die Unterlagen zu Amendment 2 wurden entsprechend § 6 unseres Statuts gemäß § 8, 5 GCP-V vom 09.08.2004 vom Vorsitzenden geprüft, es ist keine Beratung durch die gesamte Kommission erfolgt.

Wir teilen Ihnen mit, dass unsererseits keine Bedenken bestehen, da die Nutzen-Risiko-Relation durch das Protokoll Amendment 2 nicht in einer für die Beurteilung der ethischen Vertretbarkeit relevanten Weise verändert ist. Unser Primärvotum vom 02.10.2004 behält seine Gültigkeit.

Mit freundlichen Grüßen

Prof. Dr. G. Rettig-Stürmer
Vorsitzender

26. Feb. 2013

Die Ethik-Kommission bei der Ärztekammer des Saarlandes ist unter Beachtung der internationalen Richtlinien der ICH, GCP u. der 12. Novelle AMG tätig, nach Landesrecht (Saarländisches Heilberufekammergesetz, § 5 Abs. 1) anerkannt und beim Bundesinstitut für Arzneimittel und Medizinprodukte gem. § 22 des Medizinproduktegesetzes sowie beim Bundesamt für Strahlenschutz nach § 92 der Strahlenschutzverordnung und nach § 28g der Röntgenverordnung registriert.

Commerzbank Saarbrücken
Kto.-Nr. 53 89 200
BLZ 590 400 00

Dt. Apotheker- und Ärztebank Saarbrücken
Kto.-Nr. 0 001 926 209
BLZ 590 906 26

Postbank Saarbrücken
Kto.-Nr. 95 15 666
BLZ 590 100 66

Bank 1 Saar Saarbrücken
Kto.-Nr.157 5007
BLZ 591 900 00

14.8 Versicherung

GERLING
ALLGEMEINE VERSICHERUNGS-AG

Versicherungsschein
Probandenversicherung

Gerling Vertrieb Deutschland GmbH
Regionalzentrum Südwest
Postfach 15 02 01, 60062 Frankfurt

Ihr Gerling-Kundendienst
Theodor-Heuss-Allee 108, 60486 Frankfurt
Tel./Fax 069 7567-202 / 7567-267

Versicherungsnehmer/in
Universitätsklinikum des
Saarlandes
Innere Medizin I
Prof. Dr. M. Pfreundschuh
Kirrberger Str.
66424 Homburg

Bitte stets vollständig angeben!
Versicherungsschein-Nummer
70-005868405-2

Richten Sie bitte alle für uns bestimmten Anzeigen und Mitteilungen unter Angabe der Versicherungsschein-Nr. an die obenstehende betreuende Stelle

Beginn der Versicherung	Ablauf der Versicherung	
01.11.05, 12 Uhr	01.05.10, 12 Uhr	
Prämienzahlweise	Nächste Fälligkeit	Ausstellungstag
gemäß Vereinbarung	01.05.10	02.11.05
Jahresprämie	zuzüglich Versicherungssteuer und ggf. Teilzahlungszuschlag	

Versichertes Risiko

Gesundheitsschädigungen der versicherten Personen als Folge der vom Versicherungsnehmer durchgeführten Studie/Studien gemäß Risikobeschreibung Probandenversicherung für klinische Studie/Studien gemäß Arzneimittelgesetz

Bedingungen und Vereinbarungen

	Risikobeschreibung
PB 1005:07	Besondere Vereinbarungen für die Versicherung einer einzelnen klinischen Prüfung eines Arzneimittels nach dem Arzneimittelgesetz (AMG)
PB 570:06	Allgemeine Versicherungsbedingungen für klinische Prüfungen von Arzneimitteln (Probandenversicherung) - GKA PB 570:06 2002.2 -

Leistungen

01 Personenschäden (Gesundheitsschädigungen) für alle Versicherungsfälle der versicherten Studie/klinischen Prüfung		EUR	10.000.000,00
Begrenzung der unter 01 genannten Deckungssumme bei			
02 Personenschäden (Gesundheitsschädigungen) je versicherte Person/Proband	auf	EUR	500.000,00

G80212 - 05.00 **Bitte beachten Sie die Hinweise auf der Rückseite, insbesondere zum 14-tägigen Widerspruchsrech**

Probandenversicherung **Versicherungsschein** Blatt 2

Versicherungsschein-Nr. 70-005868405-2
Ausstellungstag 02.11.05

Prämienberechnung vom 01.11.05 bis 01.05.10

Berechnungsgrundlage		Jahresprämie	Erhebung/Erstattung
01 Anzahl Probanden			
Probanden	250		
Prämiensatz	135,00		
Mindestprämie EUR	30.800,00		
Einmalprämie EUR	33.750,00		EUR 33.750,00
Prämienerhebung			EUR 33.750,00

Prämienrechnung

Prämienerhebung für die Zeit vom 01.11.2005 bis 01.05.2010	EUR 33.750,00
zuzüglich 16 % Versicherungssteuer	EUR 5.400,00
Einlösungsbetrag	EUR 39.150,00

Die gegenseitigen Rechte und Pflichten regeln sich nach dem gestellten Antrag, den genannten Versicherungsbedingungen, den gesetzlichen Bestimmungen und den etwa schriftlich festgelegten besonderen Vereinbarungen.

Gerling-Konzern
Allgemeine Versicherungs-AG

Dr. Jörissen Zagel

G80118 - 07.05

Probandenversicherung **Versicherungsschein** Blatt 3

Versicherungsschein-Nr. 70-005868405-2
Ausstellungstag 02.11.05

Risikobeschreibung

Studien-Nr.: UNFOLDER 21/14-Studie DSHNHL 2004-3

Studien-Titel: "Randomisierte Studie zum Vergleich einer kombinierten Immunochemotherapie mit 6 Zyklen des monoklonalen anti-CD20-Antikörpers Rituximab in Kombination mit 6 Zyklen einer Chemotherapie mit CHOP (Cyclophosphamid, Doxorubicin, Vincristin und Prednison) in 21-tägigen Intervallen oder 14-tägigen Intervallen jeweils mit und ohne konsolidierende Strahlentherapie von großen Tumormassen und/oder Extranodalbefällen bei Patienten mit aggressiven CD20+ B-Zell-Lymphomen im Alter von 18-60 Jahren mit altersadaptiertem IPI=1 (alle) oder IPI=0 mit großen Tumormassen (Durchmesser>7.5 cm)"

Versicherungsschutz besteht für die klinische Prüfung gemäß Fassung der Patienteninformation in der Version vom 28.09.2005. Nachträgliche Änderungen / Amendments sind nicht automatisch vom Versicherungsschutz erfasst.

G80124

14.9 Definition – Stadium und Extranodalbefall

14.9.1 Stadieneinteilung

Die Stadieneinteilung erfolgt nach folgender modifizierten Fassung des Ann-Arbor Systems.

Die Regionen nach Ann-Arbor sind wie folgt definiert:

Region 1	zervikal, supraklavikulär, occipital, pre-aurikulär, nuchal, submandibulär	rechts
Region 2	zervikal, supraklavikulär, occipital, pre-aurikulär, nuchal, submandibulär	links
Region 3	infraklavikulär	rechts
Region 4	infraklavikulär	links
Region 5	axillär/ pektoral	rechts
Region 6	axillär/ pektoral	links
Region 7	mediastinal (Thymus eingeschlossen)	
Region 8	Lungenhilus	rechts
Region 9	Lungenhilus	links
Region 10	mesenterial	
Region 11	paraaortal (Milz- und Leberhilus eingeschlossen)	
Region 12	iliakal	rechts
Region 13	iliakal	links
Region 14	inguinal/femoral	rechts
Region 15	inguinal/femoral	links

Stadieneinteilung:[40]

Stadium I	N	nodaler Befall in einer einzigen Region
	E	Vorliegen eines einzigen extralymphatisch lokalisierten Herdes
Stadium II	N	nodaler Befall in zwei oder mehreren Regionen auf einer Seite des Zwerchfells
	N, E	Vorliegen von einem oder mehreren nodalen Befällen in Regionen und einem extralymphatisch lokalisierten Herd auf einer Seite des Zwerchfells
Stadium III	N	nodaler Befall in zwei oder mehreren Regionen auf beiden Seiten des Zwerchfells
	N, E	Vorliegen von einem oder mehreren nodalen Befällen in Regionen auf beiden Seiten des Zwerchfells und einem extralymphatisch lokalisierten Herd
Stadium IV	E	• ausschließlich diffuser Befall eines oder mehrerer extralymphatischer Herde (mehrere lokale Befälle in einer E-Befallslokalisation zählen auch als diffus)<br>• oder disseminierter E-Befall (mehr als ein E-Befall),<br>• oder mehrere extralymphatisch lokalisierte Herde, bei denen eine strahlentherapeutische Behandlung nicht möglich ist.<br>• Eine Beteiligung der Leber und/oder des Knochenmarks ist grundsätzlich Stadium IV,E.
	N, E	Stadium IV,E mit zusätzlichem Befall in lymphatischen Regionen

Paarige Organe zählen als 1 E-Befall (altersadjustierter IPI).

14.9.2 Extranodalbefall (E)

Beteiligung von extralymphatischem Gewebe, entweder durch direktes Einwachsen aus einem beteiligten Lymphknoten oder mit engem anatomischen Bezug.

Als jeweils ein E-Befall beim altersadjustierten IPI zählt: Knochenmark, Milz, Lunge, Leber, Knochen, Pleura, Perikard, ZNS, Magen, Dünndarm, Dickdarm und weitere E-Befälle (s. Code):

Ausnahmen:
- Haut- und Weichteilbefälle: diese zählen jeweils einzeln
- Skelett: Befälle ober- und unterhalb des Zwerchfells zählen als zwei Befälle

Code für extranodale Herde:

ORB	Orbita
NNH	Nasennebenhöhlen (Kiefer-, Stirn-, Siebbeinhöhle)
NHH	Nasenhaupthöhle
MR	Mundraum (Mundhöhle, Lippen, Rachen)
ZU	Zunge
SP	Speicheldrüsen (Ohr-, Unterkieferspeicheldrüse)
SD	Schilddrüse
BD	Brustdrüse
BF	Bauchfell
BSP	Bauchspeicheldrüse
N	Niere
NN	Nebenniere
HB	Harnblase (einschl. Harnleiter, Harnwege)
HOD	Hoden (einschl. Nebenhoden)
EST	Eierstock
GBM	Gebärmutter
H	Haut
WT	Weichteile (einschl. Muskulatur, Binde- u. Fettgewebe)
AZ	Aszites
WR	Waldeyer´scher Rachenring einschl. Tonsille
SON	Sonstiges, bitte Klartext angeben

14.9.3 Definition Allgemeinsymptome

Die Stadien I bis IV erhalten folgende Zusätze:

- Zusatz B, wenn ein oder mehrere Allgemeinsymptome vorliegen
- Zusatz A, falls diese fehlen

Allgemeinsymptome sind:

- nicht anderweitig erklärbares Fieber über 38° C
- nicht anderweitig erklärbarer Nachtschweiß (Wechsel der Nachtwäsche)
- nicht anderweitig erklärbarer Gewichtsverlust von mehr als 10 % des Körpergewichtes innerhalb von 6 Monaten.

14.10 Definition - Bulk-Befall

Ein Bulk-Befall liegt vor bei:

- massivem Lymphombefall eines Lymphknotens mit > 7,5 cm Durchmesser im größten Durchmesser oder einem Konglomerattumor mit > 7,5 cm im größten Durchmesser;
- bei einem Mediastinaltumor > 7,5 cm Durchmesser, wobei die Hili und das Perikard nicht in die Messung mit einzubeziehen sind.

Die Abmessungen sind im Staging-Bogen für jeden einzelnen Bulk-Befall genau zu dokumentieren. Dabei kann **ein** Bulk-Befall auch mehrere **benachbarte** Regionen umfassen (Konglomerattumor).

14.11 Definition – Allgemeinzustand – ECOG

Grad 0	völlige Leistungsfähigkeit, keine Symptome
Grad 1	ambulanter Patient mit Symptomen, fähig zu leichter Arbeit
Grad 2	Patient mit Symptomen, tagsüber weniger als 50% im Bett, versorgt sich selbst
Grad 3	Patient mit Symptomen, tagsüber mehr als 50% im Bett, bedarf teilweise fremder Hilfe
Grad 4	völlig bettlägerig und auf fremde Hilfe angewiesen

14.12 Internationaler Prognostischer Index

Der internationale prognostische Index (IPI) von [Shipp, 1993] enthält 5 prognostischen Faktoren. Dazu gehören:

Alter	≤ 60 Jahre	vs.	> 60 Jahre
LDH-Wert	≤ oberer Normwert	vs.	> oberer Normwert
Stadium	I,II	vs.	III,IV
Anzahl der Extranodalbefälle	≤ 1	vs.	>1
Performance Status	ECOG 0,1	vs.	ECOG 2-4

Der altersadjustierte IPI für die Patienten ≤ 60 Jahre enthält:

- LDH-Wert
- Stadium
- Performance Status.

14.13 Liste der Referenzzentren für Lymphknotenpathologie zur Studie DSHNHL 2004-3 (UNFOLDER)

**Prof. Dr. med. A. C. Feller**<br>Institut für Pathologie, Universitätsklinikum Schleswig-Holstein,<br>Campus Lübeck<br>Ratzeburger Allee 160<br>23538 Lübeck	Tel: 0451/500 2705<br>Fax: 0451/500 3328<br>E-Mail: feller@patho.uni-luebeck.de
**Prof. Dr. med. M.-L. Hansmann**<br>Senckenbergisches Institut für Pathologie,<br>Universität Frankfurt<br>Theodor-Stern-Kai 7<br>60596 Frankfurt	Tel: 069/6301 5364<br>Fax: 069/6301 5241<br>E-Mail: m.l.hansmann@em.uni-frankfurt.de
**Prof. Dr. med. W. Klapper**<br>Institut für Pathologie<br>Sektion Hämatopathologie und<br>Lymphknotenregister der Universität Kiel<br>Michaelisstraße 11<br>24105 Kiel	Tel: 0431/597 3399<br>Fax: 0431/597 3426<br>E-Mail: w.klapper@path.uni-kiel.de
**Prof. Dr. med. P. Möller**<br>Pathologisches Institut, Universität Ulm<br>Albert-Einstein-Allee 11<br>89081 Ulm	Tel: 0731/502 3321<br>Fax: 0731/502 3884<br>E-Mail: peter.moeller@medizin.uni-ulm.de
**Prof. Dr. med. A. Rosenwald**<br>Pathologisches Institut, Universität Würzburg<br>Josef-Schneider-Str. 2<br>97080 Würzburg	Tel: 0931/201 47424<br>Fax: 0931201 47440<br>E-Mail: rosenwald@mail.uni-wuerzburg.de
**Prof. Dr. med. H. Stein**<br>Pathodiagnostik Berlin<br>Komturstraße 58-62<br>12099 Berlin	Tel: 030/2360 842 10<br>Fax: 030/2360 842 19<br>E-Mail: h.stein@pathodiagnostik.de

14.14 Definition – NCI-CTC Toxizitätskriterien

Schlüssel V:Common Toxicity Criteria (CTC): Klassifikation von akuten Nebenwirkungen bei der Chemo- und Radiotherapie

C Definition gilt nur für Nebenwirkungen von Chemotherapie

R Definition gilt nur für Nebenwirkungen von Radiotherapie

■ Modifikation der Phase I/II Studiengruppe der AIO

◆ Modifikation der ARO

	**Toxizität / Grad**	**0 = „Keine“**	**1 = „Gering“/“Leicht“**	**2 = „Mäßig“/“Deutlich“**	**3 = “Stark“/“Ausgeprägt“**	**4 = “Lebensbedrohlich“**
**[1]**	**Laborwerte**	N	N = Normalbereich			
	Hämatologie					
01.01	Hämoglobin (g/100 ml)	≥ 11,0	10,0 – 10,9	8,0 – 9,9	6,5 – 7,9	< 6,5
01.02	Leukozyten (x 10^9/l)	≥ 4,0	3,0 – 3,9	2,0 – 2,9	1,0 – 1,9	< 1,0
01.03	Granulozyten (x 10^9/l)	≥ 2,0	1,5 – 1,9	1,0 – 1,4	0,5 – 0,9	< 0,5
01.04	Thrombozyten (x 10^9/l)	≥ 100,0	75,0 – 99,9	50,0 – 74,9	25,0 – 49,9	< 25
01.05	Lymphozyten (x 10^9/l)	≥ 2,0	1,5 – 1,9	1,0 – 1,4	0,5 – 0,9	< 0,5
	Blutgerinnung					
01.06	Fibrinogen	N	0,99 x N – 0,75 x N	0,74 x N – 0,50 x N	0,49 x N – 0,25 x N	≤ 0,24 x N
01.07	Prothrombinzeit	N	1,01 x N – 1,25 x N	1,26 x N – 1,50 x N	1,51 x N – 2,00 x N	> 2,00 x N
01.08	Partielle Thromboplastinzeit	N	1,01 x N – 1,66 x N	1,67 x N – 2,33 x N	2,34 x N – 3,00 x N	> 3,00 x N
	Niere / Blase					
01.09	Kreatinin	N	N – 1,5 x N	1,6 x N – 3,0 x N	3,1 x N – 6,0 x N	> 6,0 x N
01.10	Proteinurie (g/l)	keine	< 3	3-10	>10	Nephrotisches Syndrom
01.11	Harnstoff (mg %)	< 20	21-30	31-50	> 50	---
	Leber					
01.12	Bilirubin	N	---	N – 1,5 x N	>1,5 x N – 3,0 x N	> 3,0 x N
01.13	Transaminasen (SGOT/PT)	N	N – 2,5 x N	2,6 x N – 5,0 x N	5,1 x N – 20,0 x N	> 20,0 x N
01.14	Alkalische Phosphatese	N	N – 2,5 x N	2,6 x N – 5,0 x N	5,1 x N – 20,0 x N	> 20,0 x N
	Stoffwechsel					
01.15	Hyperglykämie (mg/dl)	< 116	116 – 160	161- 250	251 – 500	> 500 oder Ketoazidose
01.16	Hypoglykämie (mg/dl)	> 64	55 – 64	40 – 54	30 – 39	< 30 oder hypoglykä-mischer Schock
01.17	Amylase	N	N – 1,5 x N	1,6 x N – 2,0 x N	2,1 x N – 5,0 x N	> 5,0 x N
01.18	Hyperkalzämie (mmol/l)	< 2,65	2,65 – 2,87	2,88 – 3,12	3,13 – 3,37	> 3,37
01.19	Hypokalzämie (mmol/l)	> 2,1	2,1 – 1,95	1,94 – 1,75	1,74 – 1,51	≤ 1,50
01.20	Hypomagnesämie (mmol/l)	> 1,4	1,4 – 1,2	1,1 – 0,9	0,8 – 0,6	≤ 0,5
01.21	Hyponaträmie (mmol/l)	> 135	131 – 135	126 – 130	121 – 125	≤ 120

	Toxizität / Grad	0 = „Keine“	1 = „Gering“/“Leicht“	2 = „Mäßig“/“Deutlich“	3 = “Stark“/“Ausgeprägt“	4 = “Lebensbedrohlich“
01.22	Hypokalämie (mmol/l)	> 3,5	3,1 – 3,5	2,6 – 3,0	2,1 – 2,5	≤ 2,0
**[2]**	**Gastro-Intestinaltrakt**					
02.01	Übelkeit	Keine	Gering, normale Nahrungsaufnahme möglich	Mäßig, Nahrungsaufnahme vermindert	Stark, keine Nahrungs-aufnahme möglich	-
02.02	Erbrechen	Kein	Gering (1 mal /Tag)	Mäßig (2-5 mal / Tag)	Stark (6-10 mal /Tag)	Bedrohlich (> 10 mal / Tag) oder parenterale Ernährung
02.03	Diarrhoe	Keine	Gering vermehrt im Vergleich zu sonst (2-3 Stühle / Tag)	Mäßig vermehrt (4-6 Stühle/Tag) oder nächtliche Stühle oder mäßige Krämpfe	Stark vermehrt (7-9 Stühle/Tag) oder Inkontinenz oder schwere Krämpfe	Bedrohlich (≥10 Stühle/Tag) oder blutige Diarrhoen
02.04	Stomatitis	Keine	Geringes Wundsein, Erytheme oder schmerzlose Erosionen	Mäßig schmerzhafte Erytheme, Ödeme oder Erosionen; feste Nahrung möglich	Stark schmerzhafte Erytheme, Ödeme oder Ulzera, flüssige Nahrung nötig	Enterale oder parenterale Ernährung nötig
02.05	Ösophagitis Dysphagie	Keine	Geringes Wundsein, Erytheme oder schmerzlose Erosionen	Mäßig schmerzhafte Erytheme, Ödeme oder Erosionen oder mäßige Dysphagie; keine Analgetika nötig	Stark schmerzhafte Dysphagie, Ödeme oder Ulzera; keine feste Nahrungsaufnahme möglich oder Analgetika nötig	Kompletter Verschluß oder Perforation; enterale oder parenterale Ernährung
02.06	Gastritis/Ulkus	Keine	Gering; durch Antazida therapierbar	Mäßig; forcierte oder konservative Therapie nötig	Stark; therapieresistent, erfordert operatives Vorgehen	Perforation oder Blutung
02.07	Dünndarmobstruktion	Keine	-	Intermittierend, keine Therapie nötig	Nichtoperative Intervention nötig	Operation nötig
02.08	Intestinale Fistel	Keine	-	Vorhanden, keine Therapie nötig	Nichtoperative Intervention nötig	Operation nötig
02.09	Obstipation	Keine	Geringe Obstipation	Mäßige Obstipation	Starke Obstipation; beginnender Subileus	Ileus > 96 h
2.10C 2.10R	Schleimhäute / Mukositis (RTOG)	N	Geringes Erythem, Beläge oder Schmerz, keine Therapie nötig	Fleckige,serosanguinöse Mukositis oder Schmerzen ohne Narkotikabedarf	Konfluent fibrinöse Mukositis, Ulzeration oder Narkotika zur Schmerz-behandlung nötig	Nekrose, tiefe Ulzera oder Hämorrhagie; parenterale Ernährung

	Toxizität / Grad	0 = „Keine“	1 = „Gering“/“Leicht“	2 = „Mäßig“/“Deutlich“	3 = “Stark“/“Ausgeprägt“	4 = “Lebensbedrohlich“
2.11R	Speicheldrüsen (RTOG)	N	Geringe Mundtrockenheit oder Geschmacksstörung; zäher Speichel, normale Kost möglich	Mäßige Mundtrockenheit oder Geschmacksstörung; Speichel sehr zäh; feste bis breiige Nahrung möglich	Komplette Mundtrockenheit, kompletter Geschmacksverlust; flüssige Nahrung nötig	Akute Nekrose, tiefe Ulzera; parenterale Ernährung / PEG
**[3]**	**Herz/Kreislauf**					
03.01	Arrhythmie	Keine	Flüchtig, nicht therapiebedürftig	Wiederkehrend oder persistierend, nicht therapiebedürftig	Persistierend und therapiebedürftig	Ventrikuläre Tachykardie oder Fibrillation; Monitoring nötig
03.02	Funktion (N = ursprüngl. Volumen)	N	Abfall der linksventrikul. Ejektionsfraktion um < 20 % von N	Abfall der linksventrikulären Ejektionsfraktion um ≥ 20 % von N	Geringe kongestive Herzinsuffizienz, auf Therapie ansprechend	Erhebliche kongestive Herzinsuffizienz, therapierefraktär
03.03	Ischämie	Keine	Asymptomatisch; unspezifische T-Wellen-Abflachungen	Asymptomatisch; deutliche ST-u. T-Wellen-veränderung → Ischämie	Mäßige klinische Symptomatik:Angina pectoris ohne Infarktevidenz	Lebensbedrohliche klinische Symptomatik; akuter Infarkt
03.04	Perikard	N	Asymptomatischer Erguß, keine Intervention nötig	Perkariditissymptomatik: Reiben, Brustschmerz, EKG-Veränderungen	Symptomatischer Perikarderguß; Drainage oder spezifische Therapie nötig	Perikardtamponade; Drainage dringend nötig
03.05	Sonstiges	-	Gering	Mäßig	Ausgeprägt	Lebensbedrohlich
03.06	Hypertonie (D = diastol. Blutdruck in mmHg)	Keine	Kurzfristig Anstieg: RR > 20 (D) oder auf RR > 150 / 100	Wiederholter / persistierender Anstieg: RR > 20 (D) oder auf RR > 150 / 100	Ausgepägter / persistierender Anstieg; antihypertensive Therapie nötig	Lebensbedrohlicher Anstieg; hypertensive Krise
03.07	Hypotonie	Keine	Gering, nicht therapiebedürftig (vorübergehend Therapie möglich)	Mäßig, Flüssigkeitsersatz oder andere Therapie nötig; keine stationäre Therapie	Stark; stationäre Therapie nötig; damit Normalisierung innerhalb 48 h.	Stationäre Therapie nötig, nach 48 h. nicht normalisiert
03.08	Phlebitis/Thrombose /Embolie	Keine	--	Oberflächliche Thrombophlebitis	Tiefe Phlebothrombose	Infarkt (zerebral, hepatisch, pulmonal oder anderer) oder Lungenembolie
03.09	Ödeme	Keine	Nur am Abend	Ganztags, keine spezielle Therapie nötig	Ganztags, spezielle Therapie nötig	Generalisierte Anasarka

DSHNHL 2004-3 Version 4.0 vom 22.11.2012 3

	**Toxizität / Grad**	**0 = „Keine“**	**1 = „Gering“/“Leicht“**	**2 = „Mäßig“/“Deutlich“**	**3 = “Stark“/“Ausgeprägt“**	**4 = „Lebensbedrohlich“**
**[4]**	**Lunge/Atmungsorgane**					
04.01	Dyspnoe	Keine	Keine Symptome, pathologischer Lungenfunktionstest	Dyspnoe unter starker Belastung	Dyspnoe unter normaler Belastung	Ruhedyspnoe
04.02	Blutgase (in mmHg)	pO_2:>85 pCO_2:≤40	pO_2: 71- 85 oder pCO_2: 41 – 50	pO_2: 61 – 70 oder CO_2: 51 – 60	pO_2: 51 – 60 oder pCO_2: 61 – 70	pO_2: ≤ 50 oder pCO_2 > 70
04.03	Lungenfunktion	> 90 %	76 – 90 % des Ausgangswertes	51 – 75 % des Ausgangswertes	26 – 50 % des Ausgangswertes	≤ 25% des Ausgangswertes
04.04	Lungenfibrose	Keine	Röntgenzeichen ohne Symptome	-	Röntgenzeichen mit Symptomen	-
04.05	Lungenödem	Kein	Röntgenzeichen ohne Symptome	-	Röntgenzeichen; Diuretika nötig	Rasche Intubation nötig
04.06	Pneumonitis	Keine	Röntgenzeichen ohne Symptome	Geringe Symptome; Steroide nötig	Starke Symptomatik; Sauerstoff nötig	Assistierte Beatmung nötig
04.07	Pleuraerguß	Kein	Vorhanden	-	-	-
04.08	ARDS (Adult Respiratory Distress Syndrome)	Keines	Geringes	Mäßiges	Ausgeprägtes	Lebensbedrohlich
04.09	Husten	Kein	Gering; leichte Antitussiva	Mäßig; starke Antitussiva nötig	Stark; nicht kontrollierbarer Husten	-
04.10	Kehlkopf (RTOG)	N	Geringe oder intermittierende Heiserkeit, Reizhusten; geringes Schleimhauterythem; keine Therapie nötig	Ständig Heiserkeit, Reizhusten; Hals- Mund- und Ohrenschmerzen, fibrinöses Exsudat, mäßiges Stimmbandödem; leichte Antitussiva nötig	“Flüstersprache”; starke Schmerzen; konfluierendes fibrinöses Exsudat; ausgeprägtes Stimmbandödem; starke Analgetika und Antitussiva nötig	Massive Dyspnoe, Stridor oder Hämoptysen; Intubation oder Tracheostoma nötig
**[5]**	**Niere und Blase**					
05.01	Hämaturie	Keine	Nur mikroskopisch sichtbar	Makrohämaturie ohne Gerinnsel	Makrohämaturie mit Gerinnsel	Bedrohlich, Transfusion nötig
05.02	Hämorrhagische Zystitis	Keine	Nur mikroskopisch sichtbar	Blut makroskopisch sichtbar	Blasenspülung nötig	Zystektomie /Transfusion nötig
05.03	Inkontinenz	Keine	Streßinkontinenz (Niesen etc.)	Spontan, Kontrolle möglich	Unkontrolliert	-
05.04	Dysurie	Keine	Geringe Schmerzen oder Brennen; keine Therapie	Maßige Schmerzen oder Brennen; durch Medikamente kontrollierbar	Starke Schmerzen oder Brennen; durch Medikamente nicht kontrollierbar	-

DSHNHL 2004-3 Version 4.0 vom 22.11.2012 3

	Toxizität / Grad	0 = „Keine“	1 = „Gering“/“Leicht“	2 = „Mäßig“/“Deutlich“	3 =“Stark“/“Ausgeprägt“	4 = „Lebensbedrohlich“
05.05	Harnverhaltung	Keine	Restharn > 100 cm^3; gelegentlich Dysurie oder Katheter notwendig	Katheter immer zur Entleerung nötig	Operativer Eingriff (Transurethrale Resektion oder Dilatation) nötig	-
05.06	Vermehrt Harndrang	N	Gering vermehrter oder nächtlicher Harndrang, < 2 x N	Mäßig vermehrter Harndrang, ≥ 2 x N, aber ≤ 1 x / h	Stark vermehrter Harndrang, > 1 x / h, oder Katheterisierung nötig	-
05.07	Blasenkrämpfe	Keine	-	Vorhanden	-	-
05.08	Ureterobstruktion	Keine	Unilateral, kein Eingriff nötig	Bilateral, kein Eingriff nötig	Inkomplett bilateral, Operation (Shunt, Harn-leiterschiene, Nephrotomie) nötig	Komplette bilaterale Obstruktion
05.09	Fistelbildung	Keine	-	-	Vorhanden	-
**[6]**	**Nervensystem**					
06.01	Sensorium	N	Verlust der tiefen Sehnenreflexe; geringe Parästhesein	Mäßiger objektivierbarer sensibler Verlust; mäßig-gradige Parästhesien	Starker objektivierbarer sensibler Verlust oder Parästhesien mit Funktions-einbußen	-
06.02	Motorik	N	Geringe subjektive Schwäche, keine Funktionseinbußen	Mäßige objektive Schwäche, ohne signifikante Funktions-einbußen	Ausgeprägte objektive Schwäche mit schweren Funktionseinbußen	Paralyse
06.03	Bewußtsein	Klar, wach	Leichte Somnolenz oder agitierte Stimmungslage	Mäßige Somnolenz oder agitierte Stimmungslage	Starke Somnolenz, Agitiert-heit, Dysorientierung oder Halluzinationen	Koma, Anfälle oder toxische Psychose
06.04	Koordination	N	Geringe Dyskoordination oder Dysdiadochokinese	Mäßiger Intentionstremor, Dysmetrie, undeutliche Sprache oder Nystagmus	Ausgeprägte lokomotorische Ataxie	Zerebellare Nekrose
06.05	Gemütslage	N	Geringe Angst oder Depression	Mäßige Angstzustände oder Depression	Starke Angstzustände oder Depressionen	Selbstmordabsichten
06.06	Kopfschmerzen	Keine	Gering, kurzfristig	Mäßig bis stark, aber vorübergehend	Sehr stark und langfristig anhaltend	-
06.07	Verhaltensveränderungen	Keine	Änderung ohne negative Konsequenz für sich selbst oder die Familie	Negativer Einfluß auf sich selbst oder auf die Familie	Gefährdung für sich selbst oder andere (oder die Umwelt)	Psychotisches Verhalten

DSHNHL 2004-3 Version 4.0 vom 22.11.2012 3

	Toxizität / Grad	0 = „Keine“	1 = "Gering"/"Leicht"	2 = "Mäßig"/"Deutlich"	3 ="Stark"/"Ausgeprägt"	4 = "Lebensbedrohlich"
06.08<br><br>06.09	Schwindel/Vertigo<br><br>Geschmack	Kein<br><br>N	Gering vorhanden, kontrollierbar<br>Gering verändert, z. B. metallisch	Mäßig, schwer kontrollierbar<br>Deutlich verändert	Stark, unkontrollierbar, arbeitsunfähig<br>-	-<br><br>-
06.10	Schlafstörungen	Keine	Gering, gelegentlich Medikamente	Mäßig, kontrollierbar, häufig Medikamente	Schlafstörungen trotz Medikamenten	-
**[7]**	**Endokrines System**					
07.01	Libido	N	Gering herabgesetzt	Mäßig herabgesetzt und gestört	Stark gestört	
07.02	Amenorrhoe – Frau	Keine	Ja	-	-	-
07.03	Gynäkomastie - Mann	Keine	Geringe	Deutliche und schmerzhafte	-	-
07.04	Hitzewallungen	Keine	Gering oder < 1 x / Tag	Mäßiggradig und ≥ 1 x / Tag	Stark und häufig, sehr beeinträchtigend	-
07.05	Cushing-Syndrom	Kein	Gering erkennbar	Verstärkt bzw. deutlich erkennbar	-	-
**[8]**	**Sinnesorgane**					
08.01	Gehör/ Hörvermögen	N	Asymptomatischer Hörverlust, nur audiometrisch faßbar	Mäßige Symptomatik: Tinnitus; geringe Hypakusis bei Audiometrie	Stark beeinträchtigender Hörverlust, Korrektur mit Hörgerät (-hilfe) nötig	Nicht korrigierbare Ertaubung
08.06R	Otitis (RTOG)	Keine	Geringes Erythem, Otitis externa, Pruritus; keine Therapie	Mäßige (seröse) Otitis externa et media; lokale Therapie nötig	Starke serosanguinöse Otitis externa et media; intensive Therapie nötig	-
08.02	Auge / Sehvermögen	N	Gering vermindert	Mäßig vermindert	Symptomatischer subtotaler Sehverlust	Uni- / bilaterale Erblindung
08.03C<br>08.03R	Konjunktivitis / Keratitis (RTOG)		Geringes Erythem, Chemosis oder Konjunktivitis mit / ohne Sklerainjektion; starkes „Augentränen“; keine Steroide oder Antibiotika	Mäßiges Erythem, Chemosis oder Konjunktivitis mit/ohne Keratitis, Iritis mit Photophobie; Steroide oder Antibiotika nötig	Starke Keratitis mit Kornea-Ulzeration oder Sichttrübung; objektiver Visusverlust (= Sichttrübung); akutes Glaukom, Panophthalmie	-
08.04	„Trockenes Auge“	Nein	Gering; keine Therapie nötig	Mäßig; artefizielle Tränenflüssigkeit nötig	-	Enukleation nötig
08.05	Glaukom	Kein	-	-	Ja, vorhanden	-
08.07	Nase / Geruch	N	Gering verändert	Deutlich verändert	-	-

DSHNHL 2004-3 Version 4.0 vom 22.11.2012 3

	Toxizität / Grad	0 = „Keine“	1= "Gering"/"Leicht"	2 = "Mäßig"/"Deutlich"	3 ="Stark"/"Ausgeprägt"	4 = "Lebensbedrohlich"
[9]	**Haut/Allergie**					
09.01	Epidermis lokal (z. B. nach Injektionen)	N	Geringe Schmerzen und Schwellung	Mäßige Schmerzen und Schwellung mit Inflam-mation oder Phlebitis	Starke Schmerzen und Schwellung, Ulzerationen	Plastisch-chirurgische Therapiemaßnahmen nötig
09.02C	Epidermis systemisch (Gesamthaut betreffend)	N	Gestreute makuläre oder papulöse Eruption oder Erythem ohne Pruritus oder andere assoziierte Symptome	Dicht gestreute makuläre oder papulöse Eruption oder Erythem mit Pruritus oder anderen assoziierten Symptomen	Generalisierte makuläre papulöse oder vesikuläre Eruption mit starken assoziierten Symptomen	Generalisierte exfoliative oder ulzerierende Dermatitis
09.03	Allergie	Keine	Vorübergehend; Schüttelfrost und Fieber <38,0°C	Urtikaria, Schüttelfrost, Fieber von ≥ 38,0° C, leichter Bronchospasmus	Serumkrankheit, Bronchospasmus, parenterale Medikation nötig	Anaphylaxie
09.04	Haut/Unterhaut lokal (RTOG) (im Strahlenfeld)	N	Geringes Erythem , Epilation, trockene Desquamation,reduzierte Schweißsekretion	Mäßiges Erythem, vereinzelt feuchte Epitheliolyse (< 50%), mäßiges Ödem; lokale Therapie möglich	Ausgeprägtes Erythem, konfluierende, feuchte Epitheliolyse (≥ 50%), starkes Ödem; intensive lokale Therapie nötig	Tiefe Ulzera, Hämorrhagie oder Nekrose; operative Therapie nötig
[10]	**Allgemeinsymptome**					
10.01	Appetit	N	Gering vermindert	Kurzfristig ; < 1 Woche vermindert	Langfristig; ≥ 1 Woche vermindert	Völlige Appetitlosigkeit
10.02	Gewichtszunahme	< 5,0 %	5,0 - 9,9 %	10,0 - 19,9 %	≥ 20,0 %	-
10.03	Gewichtsabnahme	< 5,0 %	5,0 - 9,9 %	10,0 - 19,9 %	≥ 20,0 %	-
10.04	Blutungen (klinische)	Keine	Gering; keine Transfusion	Mäßig; 1-2 Transfusionen / Episode	stark; 3-4 Transfusionen / Episode	Massiv; > 4 Trans-fusionen / Episode
10.05	Alopezie	Keine	Minimal, nicht auffallend	Mässig fleckig; deutlich erkennbar	Komplett aber reversibel	Komplett und irreversibel
[11]	**Fieber/Infektion**					
11.01	Körpertemperatur	N	37,1 - 38,0° C	38,1 - 40° C	> 40,0° C für < 24 h	> 40,0° C für ≥ 24 h; Hypotension
11.02	Infektion	Keine	Gering, nicht therapiebedürftig	Mäßig, orale Antibiotika nötig	Stark, i.v. Antibiotika / Antimykotika	Lebensbedrohliche Sepsis
11.03	Schüttelfrost	Kein	Gering oder kurzfristig	Ausgeprägt und langanhaltend	-	-
11.04	Myalgie/Arthralgie	Keine	Gering, keine Beeinträchtigung	Mäßig, Bewegungseinschränkung	Arbeitsunfähig	-
11.05	Schweißtätigkeit	N	Gering und gelegentlich gesteigert	Häufig und naßgeschwitzt	-	-
12.xx	Weitere Befunde	N	„Gering“ / „Leicht“	„Mäßig“ / „Deutlich“	„sStark“ / „Ausgeprägt“	„Lebensbedrohlich“

DSHNHL 2004-3 Version 4.0 vom 22.11.2012 3

14.15 Dokumentationsbögen

14.15.1 Webbasiert

Um das Herunterladen des Protokolls aus dem Internet zu beschleunigen, wurden die Dokumentationsbögen herausgenommen.
Alle für die Randomisation eines Patienten notwendigen Unterlagen finden Sie auf der Homepage im Internet:

http://www.lymphome.de/Studien & Studiengruppen/Studienregister

Wählen Sie die gewünschte Studie aus, um auf die Seite mit den entsprechenden Unterlagen und Dokumentationsbögen zu gelangen.
Der Zugang zu den vollständigen Studienprotokollen und Dokumentationsbögen wird aus Gründen der Vertraulichkeit passwortgeschützt angeboten. Die Zugangsdaten werden Ihnen nach einer Online-Registrierung unter http://www.lymphome.de über die Zentrale des Kompetenznetzes Maligne Lymphome mitgeteilt.
Bei Rückfragen zur Registrierung wenden Sie sich bitte an die Zentrale des Kompetenznetzes Maligne Lymphome:

Zentrale des Kompetenznetz Maligne Lymphome

Tel.: 0221/478-7400 oder -7403
Fax: 0221/478-7406
E-Mail: lymphome@medizin.uni-koeln.de

14.15.2 Konventionell

Für Teilnehmer ohne Internetzugang besteht weiterhin die Möglichkeit, die Unterlagen über des Studiensekretariat der DSHNHL in Homburg/Saar anzufordern:

Studiensekretariat der DSHNHL
Prof. Dr. M. Pfreundschuh
Universitätsklinikum des Saarlandes
Innere Medizin I, Geb. 40
D-66421 Homburg/Saar

Tel.: 06841/16–23084
Fax: 06841/16–23004
E-Mail: dshnhl@uks.eu

Die Unterlagen zur:

- **Teilnahmeerklärung des Zentrums**
- **zur Aufklärung des Patienten**

finden sie weiterhin im Protokoll.
