# Supplementary material for: Radiation and Dose-densification of R-CHOP in Aggressive B-cell Lymphoma With Intermediate Prognosis: The UNFOLDER Study
Source: Hemasphere. 2023 Jul 5;7(7):e904. doi: 10.1097/HS9.0000000000000904 (PMC10325769; doi:10.1097/HS9.0000000000000904)
Supplement: Supplementary file 2 [file hs9-7-e904-s002.docx]

**Supplementary Appendix**

Supplement to: Thurner L, Ziepert M, Berdel C et al.

Radiation and Dose-Densification of R-CHOP in aggressive B-cell lymphoma with intermediate prognosis: the UNFOLDER study

| **Table of content** |  |  |
| --- | --- | --- |
|  |  |  |
| **Supplementary Tables** |  |  |
|  |  |  |
| **Supplementary Table S1** | List of UNFOLDER study investigators | **Page 3** |
| **Supplementary Table S2** | UNFOLDER Protocol Amendments | **Page 9** |
| **Supplementary Table S3** | Demographics for patients qualifying and not qualifying for radiotherapy | **Page 10** |
| **Supplementary Table S4** | Sites of extralymphatic involvements | **Page 11** |
| **Supplementary Table S5** | Primary pathology | **Page 12** |
| **Supplementary Table S6** | Causes of death | **Page 13** |
| **Supplementary Table S7** | Secondary neoplasia | **Page 14** |
|  |  |  |
| **Supplementary Figures** |  |  |
| **Supplementary Figure S1** | Total duration of CHOP chemotherapy, absolute dose of doxorubicin, and rituximab for patients qualifying for radiotherapy and not qualifying for radiotherapy | **Page 16** |
| **Supplementary Figure S2** | Event-free, progression-free and, overall survival according to therapy arms for patients qualifying for radiotherapy | **Page 17** |
| **Supplementary Figure S3** | Event-free, progression-free and, overall survival according to radiotherapy- or observation-arm in patients qualifying for radiotherapy with extralymphatic involvement | **Page 18** |
| **Supplementary Figure S4** | Progression-free and overall survival for patients qualifying for radiotherapy, who achieved a complete remission/uncertain complete remission after R-CHOP chemotherapy | **Page 19** |
| **Supplementary Figure S5** | Progression-free and overall survival for patients qualifying for radiotherapy as treated analysis | **Page 20** |
| **Supplementary Figure S6** | Event- free survival, progression-free, and overall survival according to radiotherapy- or observation-arm and R-CHOP-14 or R-CHOP21 arm for non-PMBCL patients qualifying for radiotherapy | **Page 21** |
| **Supplementary Figure S7** | Event- free, progression-free,  and overall survival for non-  PMBCL and aaIPI 1 patients  according to LDH > twice the  upper limit of normal [LDH <  twice the upper limit of normal  compared to LDH > twice the  upper limit of normal］ | **Page 22** |
| **Supplementary Figure S8** | Event- free, progression-free,  and overall survival for patients with bulky disease according to treatment arm | **Page 23** |
|  |  |  |
| **Study protocol** |  |  |
| **UNFOLDER study protocol** |  | **Page 24-seq.** |

Table S1**:** List of UNFOLDER study investigators

| Principal Investigator | Site location |
| --- | --- |
| Wolfgang Abenhardt | MOPS Elisenhof, Prielmayerstr. 1, 80335 München, GERMANY |
| Reinhard Andreesen | Klinikum der Universität Regensburg, Abteilung Hämatologie und Internistische Onkologie, Franz-Josef-Strauss-Allee 11, 93042 Regensburg, GERMANY |
| Wolfgang Bair | Schloßbergklinik Oberstaufen, Schlossstr. 27, 87534 Oberstaufen/Allgäu, GERMANY |
| Hans Becker | Hans-Susemihl-Krankenhaus Emden, Med. Klinik I, Bolardusstr. 20, 26721 Emden, GERMANY |
| Dirk Behringer | Augusta-Kranken-Anstalt gGmbH, Klinik f. Hämatologie und Onkologie, Bergstr. 26, 44791 Bochum, GERMANY |
| Martin Bentz | Städtisches Klinikum Karlsruhe, II. Med. Klinik, Moltkestr. 90, 76133 Karlsruhe, GERMANY |
| Wolfgang E. Berdel | Universitätsklinikum Münster, Med. Klinik und Poliklinik A, Hämatologie/Onkologie, Albert-Schweitzer-Str. 33, 48129 Münster, GERMANY |
| Lothar Bergmann | Klinikum d. Johann-Wolfgang-Goethe-Univ. Frankfurt, Med. Klinik III, Theodor-Stern-Kai 7, 60590 Frankfurt/Main, GERMANY |
| Harald Biersack | Universitätsklinikum Schleswig-Holstein (Campus Lübeck), Ratzeburger Allee 160, 23538 Lübeck, GERMANY |
| Stephan Bildat | Klinikum Kreis Herford, Med. Versorgungszentrum, Schwarzenmoorstr. 70, 32049 Herford, GERMANY |
| Hans Peter Böck | Gemeinschaftspraxis, Hämatologie u. Intern. Onkologie, Marktstr. 11, 63065 Offenbach, GERMANY |
| Peter Borchmann | Universitätsklinik Köln, Klinik I für Innere Medizin, Kerpener Str. 62, 50937 Köln, GERMANY |
| Jan Braess | Krankenhaus der Barmherzigen Brüder Regensburg, Prüfeninger Str. 86, 93049 Regensburg, GERMANY |
| Stefan Brettner | Kreiskrankenhaus Waldbröl, Dr.-Goldenbogen-Str. 10, 51545 Waldbröl, GERMANY |
| Maura Brugiatelli | Az. Ospedaliera Messina, Contrada Sperone, 98158 Messina, ITALY |
| Friedrich Burghardt | Evang. Krankenhaus Holzminden, Forster Weg 34, 37603 Holzminden |
| Martin Burk | Klinikum Stadt Hanau, Onkologie/Hämatologie, Leimenstr. 20, 63450 Hanau, GERMANY |
| Angelo Michele Carella | AOU San Martino Genova, Largo Rosanna Benzi 10, 16132 Genova, ITALY |
| Beate Dargel | Harz-Klinikum Wernigerode-Blankenburg GmbH, Med. Klinik, Ilsenburger str. 15, 38855 Wernigerode, GERMANY |
| Peter de Nully Brown | Rigshospitalet, Department of Hematology, Blegdamsvej 9, 2100 Kobenhavn, DENMARK |
| Matthias Demandt | MVZ Onkologie, Klinikum Straubing GmbH, St.-Elisabeth-Str. 23, 94315 Straubing, GERMANY |
| Yves Dencausse | Praxis f. Innere Medizin am KH Siloah, Wilferdinger Str. 67, 75179 Pforzheim, GERMANY |
| Judith Dierlamm | Universitätsklinikum Eppendorf, II. Med. Klinik und Poliklinik, Martinistr. 52, 20246 Hamburg, GERMANY |
| Josef Diers | St. Marienhospital Vechta, Innere Medizin, Hämatologie u. Intern. Onkologie, Marienstr. 6-8, 49377 Vechta, GERMANY |
| Hermann Dietzfelbinger | Privatklinik Dr. med. R. Schindlbeck, Seestr. 43, 82211 Herrsching, GERMANY |
| Ulrich Dührsen | Universitätsklinikum Essen, Klinik für Hämatologie, Hufelandstr. 55, 45122 Essen, GERMANY |
| Erik Engel | Hämatologisch Onkologische Praxis Altona, Mörkenstr. 43-47, 22767 Hamburg, GERMANY |
| Walburga Engel-Riedel | Lungenklinik Köln-Merheim, Ostmerheimer Str. 200, 51109 Köln, GERMANY |
| Henning Eschenburg | Gemeinschaftspraxis Dr. S. Duda/ Dr. Eschenburg/ Dr. S. Wilhelm, Am Wall 1, 18273 Güstrow, GERMANY |
| Massimo Federico | CHIMOMO Department, University of Modena and Reggio Emilia, 41100 Modena, ITALY |
| Thomas Fischer | Universitätsklinikum Magdeburg, Zentrum f. Innere Medizin, Klinik f. Hämatologie/Onkologie, Leipziger Str. 44, 39120 Magdeburg, GERMANY |
| Ludwig Fischer von Weikersthal | Klinikum St. Marien Amberg - MVZ, Mariahilfbergweg 7, 92224 Amberg, GERMANY |
| Helmut Forstbauer | Praxis Dr. med. Helmut Forstbauer, Schloßstr. 18, 53840 Troisdorf, GERMANY |
| Norbert Frickhofen | Dr.-Horst-Schmidt-Kliniken Wiesbaden, Innere Medizin III, 65199 Wiesbaden, GERMANY |
| Arnold Ganser | Medizinische Hochschule Hannover, Zentrum f. Innere Medizin, Carl-Neuberg-Str. 1, 30625 Hannover, GERMANY |
| Tobias Gaska | Brüderkrankenhaus St. Josef Paderborn, Klinik f. Hämatologie u. Onkologie, Husenerstr. 46, 33098 Paderborn, GERMANY |
| Gianfranco Giglio | Ospidale A. Cardarelli, Via L. Montalbo snc/Contrada Tappino86100 Campobasso, ITALY |
| Ulrich Graeven | Krankenhaus Maria-Hilf II Franziskushaus, Med. Klinik I, Viersener Str. 450, 41063 Mönchengladbach, GERMANY |
| Jochen Greiner | Diakonie Klinikum Stuttgart, Med. Klinik II, Rosenbergstr. 38, 70176 Stuttgart, GERMANY |
| Frank Griesinger | Pius-Hospital Oldenburg, Klinik für Internistische Onkologie, Georgstr. 12, 26121 Oldenburg, GERMANY |
| Corinna Hahn-Ast | Med. Univ. Poliklinik Bonn, Med. Klinik u. Poliklinik III, Venusberg-Campus 1, 53127 Bonn, GERMANY |
| Mathias Hänel | Krankenhaus Küchwald Chemnitz, Klinik für Innere Medizin III, Bürgerstr. 2, 09009 Chemnitz, GERMANY |
| Frank Hartmann | Klinikum Lippe-Lemgo GmbH, med. Klinik II, Rintelner Str. 85, 32657 Lemgo, GERMANY |
| Jörg Thomas Hartmann | Franziskus-Hospital Bielefeld, Kisker Str. 26, 33615 Bielefeld, GERMANY |
| Holger Hebart | Stauferklinik Schwäbisch Gmünd, Wetzgauer Str. 85, 73557 Mutlangen, GERMANY |
| Michael Heike | Klinikum Dortmund, Medizinische Klinik Mitte, Beurhausstr. 40, 44137 Dortmund, GERMANY |
| Gerhard Heil | Märkische Kliniken GmbH, Klinikum Lüdenscheid, Paulmannshöher Str. 14, 58515 Lüdenscheid, GERMANY |
| Tobias Heintges | Lukaskrankenhaus Neuss, Med. Klinik II, Preußenstr. 84, 41464 Neuss, GERMANY |
| Manfred Hensel | Gemeinschaftspraxis Dres. med. Brust/Schuster/Plöger/Hensel, Q5, 68161 Mannheim, GERMANY |
| Bernd Hertenstein | Klinikum Bremen-Mitte gGmbH, Hämatologie, St.-Jürgen-Str. 1, 28177 Bremen, GERMANY |
| Heinz-Gert Höffkes | Städt. Klinikum Fulda, Med. Klinik III, Pacelliallee 4, 36043 Fulda, GERMANY |
| Martin Hoffmann | Klinikum der Stadt Ludwigshafen, Med. Klinik A, Bremserstr. 79, 67063 Ludwigshafen, GERMANY |
| Jörg Hoffmann | St. Marienkrankenhaus Ludwigshafen, Salzburger Str. 15, 67067 Ludwigshafen, GERMANY |
| Hans-Jürgen Hurtz | GMP Rohrberg/Hurtz/Schmidt/Frank-Gleich, Niemeyerstr. 23, 06110 Halle, GERMANY |
| Elke Jäger | Krankenhaus Nordwest Frankfurt, II. Med. Klinik, Steinbacher Hohl 2-26, 60488 Frankfurt/Main, GERMANY |
| Jan Janssen | Onkologische Praxis Aurich Dres. Reichert/Janssen, Wallinghausenerstraße 8-10, 26603 Aurich, GERMANY |
| Bjarne Anker Jensen | Amtssygehuset i Herlev, Haematologisk afd. L, Herlev Hospital, 2730 Herlev, DENMARK |
| Christoph Kahl | Universität Rostock, Abteilung Hämatologie/Onkologie, Klinik u. Poliklinik für Innere Medizin, Ernst-Heydemann-Str. 6, 18057 Rostock, GERMANY |
| Christoph Kahl | Klinikum Magdeburg, Birkenallee 34, 39130 Magdeburg, GERMANY |
| Ulrich Kaiser | St. Bernward Krankenhaus, Med. Klinik II, Treibestr. 9, 31132 Hildesheim, GERMANY |
| Ulrich Keller | Klinikum rechts der Isar, III. Med. Klinik und Poliklinik, Ismaninger Str. 22, 81675 München, GERMANY |
| Barbara Kempf | Klinikum Landshut, Med. Klinik I, Robert-Koch-Str. 1, 84034 Landshut, GERMANY |
| Alexander Kiani | Klinikum Bayreuth GmbH, Med. Klinik IV, Preuschwitzerstr. 101, 95445 Bayreuth, GERMANY |
| Michael Kiehl | Klinikum Frankfurt (Oder), Abteilung Innere Medizin, Müllroser Chaussee 7, 15236 Frankfurt/Oder, GERMANY |
| Heinz Kirchen | Krankenhaus der Barmherzigen Brüder, I. Med. Abteilung, Nordallee 1, 54292 Trier, GERMANY |
| Bodo Klump | Paracelsus-Krankenhaus Ruit, Klinik für Allgemeine Innere Medizin, Gastroenterologie und Tumormedizin, Kreiskliniken Esslingen, Hedelfinger Str. 166, 73760 Ostfildern |
| Michael Kneba | Städtisches Krankenhaus Kiel, II. Med. Klinik und Poliklinik, Chemnitzstr. 33, 23116 Kiel, GERMANY |
| Yon-Dschun Ko | Johanniter Krankenhaus Bonn, Internistische Onkologie, Johanniterstr. 3-5, 53113 Bonn, GERMANY |
| Georg Köchling | Onkologie Schwarzwald-Alb, Onkologische Schwerpunktpraxis, Albert-Schweitzer-Str. 14, 78052 VS-Villingen, GERMANY |
| H. Köppler | Gemeinschaftspraxis Dres. Köppler/Heymanns/Weide/Thomalla/van Roye, Neversstr. 5, 56068 Koblenz, Germany |
| Detlev Kohl | Ammerland-Klinik GmbH, Lange Str. 38, 26655 Westerstede, GERMANY |
| Beate Krammer-Steiner | Klinikum Rostock Südstadt, Innere Medizin, Südring 81, 18059 Rostock, GERMANY |
| Jürgen Krauter | Städt. Klinikum Braunschweig, Med. Klinik, Celler Str. 38, 38114 Braunschweig, GERMANY |
| Ute Kreibich | Heinrich-Braun-KH/ Städtisches Klinikum Zwickau, Klinik für Innere Medizin II, Karl-Keil-Str. 35, 08009 Zwickau, GERMANY |
| Stephan Kremers | Caritas-Krankenhaus Lebach, Heeresstr. 49, 66822 Lebach, GERMANY |
| Ekkehard Ladda | Onkologische Schwerpunktpraxis, Nürnberger Str. 12, 92318 Neumarkt, GERMANY |
| Christof Lamberti | Klinikum Coburg GmbH, Med. Klinik V, Hämatologie, Intern. Onkologie, Ketschendorfer Str. 33, 96450 Coburg, GERMANY |
| Helmut Lambertz | Klinikum Garmisch-Partenkirchen, Auenstr. 6, 82467 Garmisch-Partenkirchen, GERMANY |
| Elisabeth Lange | Evangelisches Krankenhaus Hamm, Med. Klinik, Hämatologie/Onkologie, Werler Str. 110, 59063 Hamm, GERMANY |
| Eva Lengfelder | Universitätsmedizin Mannheim, III. Med. Universitätsklinik, Theodor-Kutzer-Ufer 1-3, 68167 Mannheim, GERMANY |
| Christian Lerchenmüller | Onkologische Schwerpunktpraxis, Steinfurter Str. 60 B, 48151 Münster, GERMANY |
| Christina Limmroth | Krankenhaus Holweide, Kliniken der Stadt Köln, Med. Klinik, Neufelderstr. 32, 51069 Köln, GERMANY |
| Hans-Walter Lindemann | St.-Josefs -Hospital Hagen, Hämatologie u. Internistische Onkologie, Bergstr. 56, 58095 Hagen, GERMANY |
| Wolf-Dieter Ludwig | Helios Klinikum Berlin Buch/Robert-Rössle-Klinik, Schwanebecker Chaussee 50, 13125 Berlin, GERMANY |
| Ludwig Lutz | Städt. Krankenhaus München-Harlaching, Sanatoriumsplatz 2, Klinik f. Hämatologie, Onkologie u. Palliativmedizin, 81545 München, GERMANY |
| Andreas Mackensen | Universitätsklinik Erlangen-Nürnberg, Maximiliansplatz 2, 91054 Erlangen |
| Rolf Mahlberg | KA Mutterhaus der Borromäerinnen, Med. Abteilung I, Feldstr. 16, 54290 Trier, GERMANY |
| Luisa Mantovani-Löffler | Klinikum "St. Georg", Delitzscherstr. 141, 04129 Leipzig, GERMANY |
| Reinhard Marks | Universitätsklinikum Freiburg, Abt. Innere Medizin I, Hugstetter Str. 55, 76106 Freiburg, GERMANY |
| Felix Marquard | Praxis Dr. Felix Marquard, Neumarkt 1D, 29221 Celle |
| Patrizio Mazza | Ospedale San Giuseppe Moscati, Ematologia, Via per Martina Franca, 74100 Taranto, ITALY |
| Gerald Meckenstock | St. Josef-Hospital Gelsenkirchen, Abt. für Onkologie u. Hämatologie, Rudolf-Bertram-Platz 1, 45699 Gelsenkirchen, GERMANY |
| Hans-Günther Mergenthaler | Bürgerhospital Stuttgart, Tunzhofer Str. 14-16, 70191 Stuttgart, GERMANY |
| Hans-Günther Mergenthaler | Katharinenhospital Stuttgart, Kriegsbergstr. 60, 70174 Stuttgart, GERMANY |
| Francesco Merli | Hematology Azienda USL-IRCCS di Reggio Emilia, 41100 Reggio Emilia, ITALY |
| Bernd Metzner | Klinikum Oldenburg gGmbH, Abt. Hämatologie/Onkologie, Rahel-Straus-Str. 10, 26133 Oldenburg, GERMANY |
| Jörg Mezger | St.-Vincentius-Krankenhäuser Karlsruhe, Med. Klinik A, Südendstr. 32, 76137 Karlsruhe, GERMANY |
| Fortunato Morabito | Azienda Ospedaliera di Cosenza, Via Felize Migliori 1, 87100 Cosenza, ITALY |
| Lothar Müller | Schwerpunktpraxis f. Hämatologie u. Intern. Onkologie, Annenstr. 11, 26789 Leer, GERMANY |
| Caterina Musolino | Policlinico Universitario "G. Martino" , Via Consolare Valeria, 98100 Messina, ITALY |
| Ralph Naumann | St. Marien-Krankenhaus, Kampenstr. 51, 57072 Siegen, GERMANY |
| Andreas Neubauer | Universitätsklinikum Marburg, Klinik für Innere Medizin, Baldingerstraße, 35033 Marburg, GERMANY |
| Godehard Obst | Praxis für Hämatologie und Internistische Onkologie, Hannovesche Str. 2, 30938 Burgwedel, GERMANY |
| Bettina Peuser | Onkolog. Praxis am Diakonissenhaus Leipzig, Fachärztin für Innere Medizin, Georg-Schwarz-Str. 53, 04179 Leipzig, GERMANY |
| Michael Pfreundschuh | Universitätsklinikum des Saarlandes, Innere Medizin I, 66424 Homburg, GERMANY |
| Grabiella Pinotti | Ospedale di circolo e Fondazione Macchi Varese, Viale Luigi Borri 57, 21100 Varese, ITALY |
| Hans-Jörg Pohl | Virngrund-Klinik Ellwangen, Innere Abteilung, Dalkinger Str. 8-12, 73479 Ellwangen |
| Kurt Possinger | Campus Charité Mitte, Medizinische Klinik, Schumannstr. 20/21, 10117 Berlin, GERMANY |
| Otto Prümmer | Klinikum Kempten-Oberallgäu gGmbH, Innere Medizin III, Hämatologie u. Onkologie, Memminger Str. 50-52, 87439 Kempten, GERMANY |
| Aruna Raghavachar | Helios Klinikum Wuppertal, Med. Klinik I, Heusnerstr. 40, 42283 Wuppertal, GERMANY |
| Andreas Rank | Zentralklinikum Augsburg, II. Med. Klinik, Stenglinstr. 2, 86156 Augsburg, GERMANY |
| Tobias Reiber | Praxis Dr. Reiber, Facharzt f. Innere Medizin, Hämatologie, Internist. Onkologie, Schreiberstr. 20, 79098 Freiburg, GERMANY |
| Hans Reinel | Leopoldina-Krankenhaus Schweinfurt, Med. Klinik II, Gustav-Adolf-Str. 8, 97422 Schweinfurt, GERMANY |
| Eva Römer | Klinikum Idar-Oberstein, Dr.-Ottmar-Kohler-Str. 2, 55743 Idar-Oberstein, GERMANY |
| Volker Runde | Wilhelm-Anton-Hospital, Klinik für Innere Medizin, Hämatologie/Intern. Onkologie, Vossheider Str. 214, 47574 Goch, GERMANY |
| Mario Russo | Osp. S. Vincenzo – Taormina, Contrada Sirina, 98039 Taormina, ITALY |
| Matthias Sandmann | Kliniken St. Antonius Wuppertal, Hämatologie/Onkologie, Carnaper Str. 48, 42283 Wuppertal, GERMANY |
| Thomas Schichtl | Med. Versorgungszentrum Weiden, Sölingerstr. 16, 92637 Weiden, GERMANY |
| Frank Schlegel | St.-Antonius-Hospital Eschweiler, Abteilung für Hämatologie u. Onkologie, Dechant-Deckers-Str. 8, 52249 Eschweiler, GERMANY |
| Christian A. Schmidt | Ernst-Moritz-Arndt-Universität, Med. Universitätsklinik C, Hämatologie und Onkologie, Sauerbruchstr., 17487 Greifswald, GERMANY |
| Christian Schmidt | Klinikum Großhadern, Med. Klinik und Poliklinik III, Marchioninistr. 15, 81366 München, GERMANY |
| Rudolf Schmits | Gemeinschaftspraxis Hämat. und Intern. Onkologie, Am Ludwigsberg 78, 66113 Saarbrücken, GERMANY |
| Clemens Schmitt | Charité, Campus Virchow-Klinikum, Med. Klinik, Hämatologie und Onkologie, Augustenburger Platz 1, 13353 Berlin, GERMANY |
| Norbert Schmitz | Asklepios Klinik St. Georg, Hämatologie, Onkologie, Stammzelltransplantationen, Lohmühlenstr. 5, 20099 Hamburg, GERMANY (former address). For current address please see affiliation number 34 |
| Stephan Schmitz | Gemeinschaftspraxis für Onkologie und Hämatologie Köln, Dres. Schmitz/Steinmetz/Gabor, Sachsenring 69, 50677 Köln, GERMANY |
| Jan Schröder | Gemeinschaftspraxis Dres. med. Schröder/Sieg, Kettwiger Str. 62, 45468 Mülheim/Ruhr, GERMANY |
| Wolfgang Schütte | Städt. Krankenhaus Martha-Maria Halle, Innere Medizin II, Röntgenstr. 1, 06120 Halle, GERMANY |
| Dieter Semsek | Onkologische Gemeinschaftspraxis Marschner, Zeiss, Kirste, Semsek, Wirthstr. 11c, 79110 Freiburg, GERMANY |
| Ofer Shpilberg | Rabin Medical Center, Beilinson Hospital, Hematology departement, Jabutinski 95, 49100 Petah-Tiqwa, ISRAEL |
| Martin Sökler | Universitätsklinikum Tübingen, Med. Klinik und Poliklinik, Otfried-Müller-Str. 10, 72076 Tübingen, GERMANY |
| Ulrike Söling | Gemeinschaftspraxis Dres. Siehl/Söling, Goethestr. 47, 34119 Kassel, GERMANY |
| Martina Stauch | Gemeinschaftspraxis Dr. med. Martina Stauch, Niederbronner Str. 2, 96317 Kronach, GERMANY |
| Hjalmar Steinhauer | Carl-Thiem-Klinikum Cottbus, Med. Klinik II, Thiemstr. 111, 03048 Cottbus, GERMANY |
| Eckhard Thiel | Universitätsklinikum Benjamin Franklin, Charité, Med. Klinik III, Hindenburgdamm 30, 12200 Berlin, GERMANY |
| Van Anh Tran Nguyen | Katharinen Hospital UNNA, Innere Klinik I/Gastroenterologie, Obere Husemannstr. 2, 59423 Unna, GERMANY |
| Ralf Ulrich Trappe | Evang. Diakonie-Krankenhaus gGmbH, Med. Klinik II, Gröpelinger Heerstr. 406-408, 28239 Bremen, GERMANY |
| Lorenz Trümper | Georg-August-Universität Göttingen, Hämatologie und Onkologie, Zentrum für Innere Medizin, Robert-Koch-Str. 40, 37075 Göttingen, GERMANY |
| Dirk Tummes | Hämatologische Praxis, Weberstr. 8, 52070 Aachen, GERMANY |
| Daniele Vallisa | Ematologia - OSP. Civile Piacenza, Via Taverna 49, 29100 Piacenza, ITALY |
| Ursula Vehling-Kaiser | Praxis Dr. med. Vehling-Kaiser, Landgasse 132-135, 84028 Landshut, GERMANY |
| Andreas Viardot | Med. Universitätsklinik Ulm, Innere Abteilung III, Albert-Einstein-Allee 23, 89081 Ulm, GERMANY |
| Alexander Wacker | Kreiskliniken Reutlingen, Klinikum am Steinenberg, Steinenbergstr. 31, 72764 Reutlingen, GERMANY |
| Wolfgang Weber | Praxis für Hämatologie/Intern. Onkologie, Melsunger Str. 11, 34576 Homberg Efze, GERMANY |
| Paul Weber | Krankenhaus Siloah Pforzheim, Klinik für Innere Medizin I, Wilferdinger Str. 67, 75179 Pforzheim |
| Georg Weißenborn | Praxis Dr. Georg Weißenborn, Kirchstr. 7, 27239 Twistringen, GERMANY |
| Swen Weßendorf | Städtische Kliniken, Onkologie/Hämatologie, Gastroenterologie und Infektiologie, Hirschlandstr.97, 73730 Esslingen, GERMANY |
| Mathias Witzens-Harig | Ruprecht-Karls-Universität Heidelberg, Med. Klinik, Abt. Innere Medizin, Im Neuenheimer Feld 410, 69120 Heidelberg, GERMANY |
| Hans-Heinrich Wöltjen | Klinikum Minden, Hans-Nolte-Str. 1, 32429 Minden, GERMANY |

Table S2: UNFOLDER Protocol Amendments

|  | Version | Date of version | Date of approval by the Ethics Committee of the Medical Council of Saarland | Date of activation | Changes |
| --- | --- | --- | --- | --- | --- |
| Initial version | 3.1 | 15 September 2004 | 02 October 2004 | - | - |
| Amendment 1 | 3.2 | 27 June 2005 | 18 August 2005 | - | - |
| Supplement | 3.2.1 | 28 September 2005 | 07 October 2005 | 02 January 2006  (start of recruitment) | Change of patient informed consent form |
| Supplement | 3.2.2 | 06 February 2006 | 02 March 2006 | 06 April 2006 | I.th. CNS prophylaxis also for patients with Burkitt and Burkitt-like lymphoma, addition of rate of secondary neoplasia as secondary endpoint, clarification and editorial changes |
| Amendment 2 | 4.0 | 22 November 2012 | 25 February 2013 | 08 May 2013 | Closure of the two treatment arms (R-CHOP-21, R-CHOP-14) without radiotherapy for patients with bulky disease and/or extranodal involvement, abandonement of i.th. CNS prophylaxis, introduction of prophylactic irradiation of contralateral testis in case of testicular lymphoma, clarification and editorial changes |

Table S3: Demographics for patients qualifying and not qualifying for radiotherapy

|  | qualifying for  radiotherapy | not qualifying for radiotherapy | UNFOLDER |
| --- | --- | --- | --- |
|  | (n=467) | (n=228) | (n=695) |
| Male  Female | 261 (56%)  206 (44%) | 142 (62%)  86 (38%) | 403 (58%)  292 (42%) |
| Age, median (range) | 44 (18, 60) | 50 (18, 60) | 47 (18, 60) |
| Serum lactate dehydrogenase > than upper limit of normal | 211 (45%) | 82 (36%) | 293 (42%) |
| Eastern Cooperative Oncology Group Performance status > 1 | 2 (0.4%) | 1 (0.4%) | 3 (0.4%) |
| Stage III/ IV | 165 (35%) | 131 (57%) | 296 (43%) |
| Age-adjusted International Prognostic Index  0*  1  2 | 94 (20%)  368 (79%)  5 (1%) | 17 (7%)  208 (91%)  3 (1%) | 111 (16%)  576 (83%)  8 (1%) |
| Stage I  II  III  IV | 122 (26%)  180 (39%)  47 (10%)  118 (25%) | 37 (16%)  60 (26%)  77 (34%)  54 (24%) | 159 (23%)  240 (35%)  124 (18%)  172 (25%) |
| Extralymphatic involvement. | 242 (52%) | 82 (36%) | 324 (47%) |
| Extralymphatic involvement > 1 | 88 (19%) | 25 (11%) | 113 (16%) |
| Bulk ≥ 7·5 cm | 357 (76%) | 39 (17%) | 396 (57%) |
| B symptoms** | 118 (25%) | 34 (15%) | 152 (22%) |
| Bone marrow involvement | 22 (5%) | 19 (8%) | 41 (6%) |
| Reference pathology available | 449 (96%) | 206 (90%) | 655 (94%) |
| DLBCL | 396 (88%) | 161 (78%) | 557 (85%) |
| PMBCL*** | 131 (29%) | 5 (2%) | 136 (21%) |
| Follicular lymphoma IIIb | 9 (2%) | 10 (5%) | 19 (3%) |
| Follicular lymphoma III° + DLBCL | 14 (3%) | 22 (11%) | 36 (6%) |
| Burkitt‘s lymphoma | 3 (1%) | 3 (1%) | 6 (1%) |
| Burkitt-like | 3 (1%) | 0 (0%) | 3 (0.5%) |
| Aggressive marginal zone lymphoma | 1 (0.2%) | 4 (2%) | 5 (1%) |
| Grey zone lymphoma | 3 (1%) | 0 (0%) | 3 (0·5%) |
| B-cell, NOS | 1 (0.2%) | 2 (1%) | 3 (0·5%) |
| B-cell, unclassified (techn. insufficient mat.) | 7 (2%) | 0 (0%) | 7 (1%) |
| Other, not B-cell | 12 (3%) | 4 (2%) | 16 (2%) |

*10 (4/6) IPI=0 without bulk **4 (3/1) missing values *** Subtype of DLBCL

Bone marrow is counted as extralymphatic; spleen and Waldeyers ring are counted as lymphathic

DLBCL: diffuse large B-cell lymphoma, PMBCL: primary mediastinal B-cell lymphoma, NOS: not otherwise specified

Table S4: Sites of extralymphatic involvements

| Localisation | qualifying for radiotherapy (n=467) | | | | not qualifying for radiotherapy (n=228) | |
| --- | --- | --- | --- | --- | --- | --- |
|  | R-CHOP-21  (n=81) | R-CHOP-14  (n=81) | R-CHOP-21 +radiotherapy  (n=155) | R-CHOP-14 +radiotherapy  (n=150) | R-CHOP-21  (n=114) | R-CHOP-14  (n=114) |
| Bone marrow | 3 (4%) | 5 (6%) | 8 (5%) | 6 (4%) | 13 (11%) | 6 (5%) |
| Lung | 2 (2%) | 3 (4%) | 9 (6%) | 8 (5%) | 4 (4%) | 4 (4%) |
| Liver | 0 (0%) | 1 (1%) | 1 (1%) | 3 (2%) | 2 (2%) | 2 (2%) |
| Skeletal* | 11 (14%) | 9 (11%) | 20 (13%) | 18 (12%) | 4 (4%) | 1 (1%) |
| Pleura | 2 (2%) | 5 (6%) | 5 (3%) | 3 (2%) | 0 (0%) | 1 (1%) |
| Pericard | 1 (1%) | 1 (1%) | 5 (3%) | 5 (3%) | 0 (0%) | 0 (0%) |
| CNS | 0 (0%) | 0 (0%) | 0 (0%) | 0 (0%) | 0 (0%) | 0 (0%) |
| Stomach | 5 (6%) | 2 (2%) | 8 (5%) | 4 (3%) | 1 (1%) | 4 (4%) |
| Small intestine | 5 (6%) | 0 (0%) | 5 (3%) | 5 (3%) | 4 (4%) | 11 (10%) |
| Colon | 1 (1%) | 0 (0%) | 2 (1%) | 3 (2%) | 4 (4%) | 2 (2%) |
| Orbita | 0 (0%) | 0 (0%) | 0 (0%) | 3 (2%) | 0 (0%) | 0 (0%) |
| Paranasal sinuses | 1 (1%) | 3 (4%) | 4 (3%) | 5 (3%) | 0 (0%) | 1 (1%) |
| Main nasal cavity | 2 (2%) | 1 (1%) | 2 (1%) | 3 (2%) | 1 (1%) | 0 (0%) |
| Mouth region | 4 (5%) | 2 (2%) | 4 (3%) | 5 (3%) | 1 (1%) | 0 (0%) |
| Tongue | 0 (0%) | 1 (1%) | 2 (1%) | 2 (1%) | 0 (0%) | 0 (0%) |
| Salivary glands | 1 (1%) | 1 (1%) | 1 (1%) | 4 (3%) | 1 (1%) | 0 (0%) |
| Thyroid gland | 2 (2%) | 3 (4%) | 2 (1%) | 3 (2%) | 2 (2%) | 4 (4%) |
| Mammary gland | 0 (0%) | 0 (0%) | 6 (4%) | 3 (2%) | 0 (0%) | 0 (0%) |
| Peritoneum | 0 (0%) | 2 (2%) | 1 (1%) | 1 (1%) | 1 (1%) | 0 (0%) |
| Pancreas | 0 (0%) | 1 (1%) | 1 (1%) | 1 (1%) | 1 (1%) | 0 (0%) |
| Kidney | 2 (2%) | 1 (1%) | 1 (1%) | 2 (1%) | 0 (0%) | 2 (2%) |
| Adrenal gland | 0 (0%) | 0 (0%) | 0 (0%) | 0 (0%) | 2 (2%) | 2 (2%) |
| Urinary bladder | 0 (0%) | 0 (0%) | 2 (1%) | 0 (0%) | 0 (0%) | 0 (0%) |
| Testes | 0 (0%) | 0 (0%) | 0 (0%) | 0 (0%) | 6 (5%) | 4 (4%) |
| Ovary | 0 (0%) | 0 (0%) | 1 (1%) | 1 (1%) | 5 (4%) | 1 (1%) |
| Uterus | 2 (2%) | 0 (0%) | 1 (1%) | 3 (2%) | 1 (1%) | 0 (0%) |
| Skin | 1 (1%) | 1 (1%) | 5** (3%) | 2 (1%) | 2 (2%) | 0 (0%) |
| Soft tissues | 9 (11%) | 15 (19%) | 22 (14%) | 17 (11%) | 5*** (4%) | 7 (6%) |
| Ascites | 0 (0%) | 0 (0%) | 0 (0%) | 0 (0%) | 1 (1%) | 0 (0%) |
| Other | 5**** (6%) | 2 (2%) | 4 (3%) | 4 (3%) | 2 (2%) | 1 (1%) |

*13 (1/3/5/2/1/1) with cranial and caudal skeletal involvement

**1 with two skin involvements

***1 with two soft tissues involvements

****1 with two other involvements

Table S5: Primary pathology

|  | qualifying for radiotherapy | not qualifying for radiotherapy | UNFOLDER |
| --- | --- | --- | --- |
|  | (n=467) | (n=228) | (n=695) |
| DLBCL* | 431 (92%) | 190 (83%) | 621 (89%) |
| Follicular lymphoma III°b | 5 (1%) | 8 (4%) | 13 (2%) |
| Follicular lymphoma III° + DLBCL | 13 (3%) | 20 (9%) | 33 (5%) |
| Burkitt‘s lymphoma | 0 (0%) | 1 (0.4%) | 1 (0·1%) |
| Burkitt-like | 0 (0%) | 1 (0.4%) | 1 (0·1%) |
| Aggressive marginal zone lymphoma | 1 (0·2%) | 2 (1%) | 3 (0·4%) |
| B-cell, NOS | 17 (4%) | 6 (3%) | 23 (3%) |

DLBCL: diffuse large B-cell lymphoma, NOS: not otherwise specified

* including primary mediastinal B-cell lymphoma (PMBCL).

Table S6: Causes of death

|  | qualifying for radiotherapy (n=467) | | | | not qualifying for radiotherapy (n=228) | |
| --- | --- | --- | --- | --- | --- | --- |
|  | R-CHOP-21  (n=81) | R-CHOP-14  (n=81) | R-CHOP-21 + radiotherapy  (n=155) | R-CHOP-14 + radiotherapy  (n=150) | R-CHOP-21  (n=114) | R-CHOP-14  (n=114) |
| Lymphoma related | 4 | 4 | 9 | 10 | 8 | 3 |
| Related to study treatment^#^ | 0 | 1 | 2 | 0 | 0 | 1 |
| Secondary neoplasia | 2 | 0 | 2 | 1 | 1 | 2 |
| Concomitant diseases | 0 | 1 | 1 | 0 | 1 | 2 |
| Other* | 1 | 1 | 0 | 0 | 0 | 3 |
| Unknown | 0 | 0 | 1 | 0 | 0 | 0 |
| Total (patients died) | 7 (9%) | 7 (9%) | 15 (10%) | 11 (7%) | 10 (9%) | 11 (10%) |

^#^ all deaths related to study treatment were related to R-CHOP chemotherapy

* sudden cardiac arrest, traffic accident, suicide, cardiogenic shock during percutaneous coronary intervention, liver failure

Table S7: Secondary neoplasia

|  | qualifying for radiotherapy (n=467) | | | | not qualifying for radiotherapy (n=228) | |
| --- | --- | --- | --- | --- | --- | --- |
|  | R-CHOP-21  (n=81) | R-CHOP-14  (n=81) | R-CHOP-21 + radiotherapy  (n=155) | R-CHOP-14 + radiotherapy  (n=150) | R-CHOP-21  (n=114) | R-CHOP-14  (n=114) |
| Breast cancer | 1 | 1 | - | 1 | 1 | - |
| Lung cancer | - | - | 1 | 1 | 2 | 2 |
| Head and neck cancer | 1 | - | 1 | - | - | 1 |
| Renal cell carcinoma | - | - | - | - | - | 1 |
| Prostate cancer | - | - | 1 | 1 | - | 1 |
| Rectal cancer | - | - | - | - | 1 | - |
| Gastric cancer | - | - | 1 | - | - | - |
| Cholangiocellular-Carcinoma | - | - | 1 | - | - | - |
| Neuroendocrine tumor of ileum  Neuroendocrine carcinoma of the gastro-intestinal tract | 1 | -  - | -  - | 1  - | -  - | -  - |
| MALT-lymphoma | - | - | 1 (ocular adnexis) | - | - | 1 (parotic gland) |
| Basal cell carcinoma | - | - | - | - | 1 | - |
| Malignant Melanoma | 1 | - | - | - | - | - |
| Multiple Myeloma | - | - | - | - | - | 1 |
| Rhabdomyosarcoma | - | - | 1 | - | - | - |
| Liposarcoma | - | - | - | - | - | 1 |
| Gastrointestinal stroma tumor | - | - | - | - | 1 | - |
| Hodgkin‘s Lymphoma | - | - | - | - | 1 | - |
| Acute myeloid leukemia | - | - | - | - | 1 | - |
| Total | 4 (5%) | 1 (1%) | 7 (4%) | 4 (3%) | 8 (7%) | 8 (7%) |


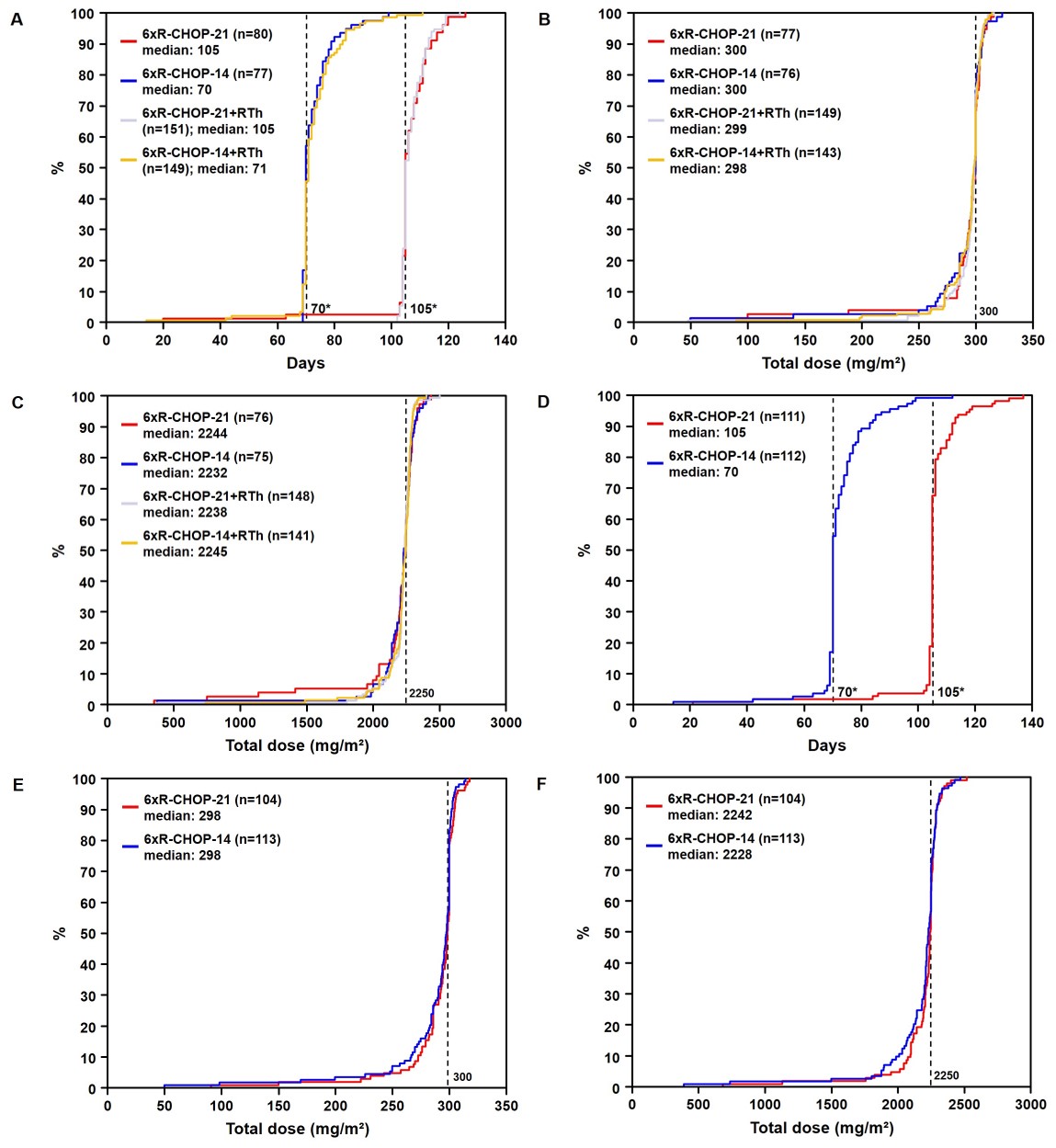
**Supplementary Figure S1: Total duration of CHOP chemotherapy, absolute dose of doxorubicin, and rituximab.** Graphs show total duration of CHOP chemotherapy in patients qualifying for radiotherapy (A), absolute dose of doxorubicin in patients qualifying for radiotherapy (B), absolute dose of rituximab in patients qualifying for radiotherapy C) and total duration of CHOP chemotherapy in patients not qualifying for radiotherapy (D), absolute dose of doxorubicin in patients not qualifying for radiotherapy (E), absolute dose of rituximab in patients not qualifying for radiotherapy (F).


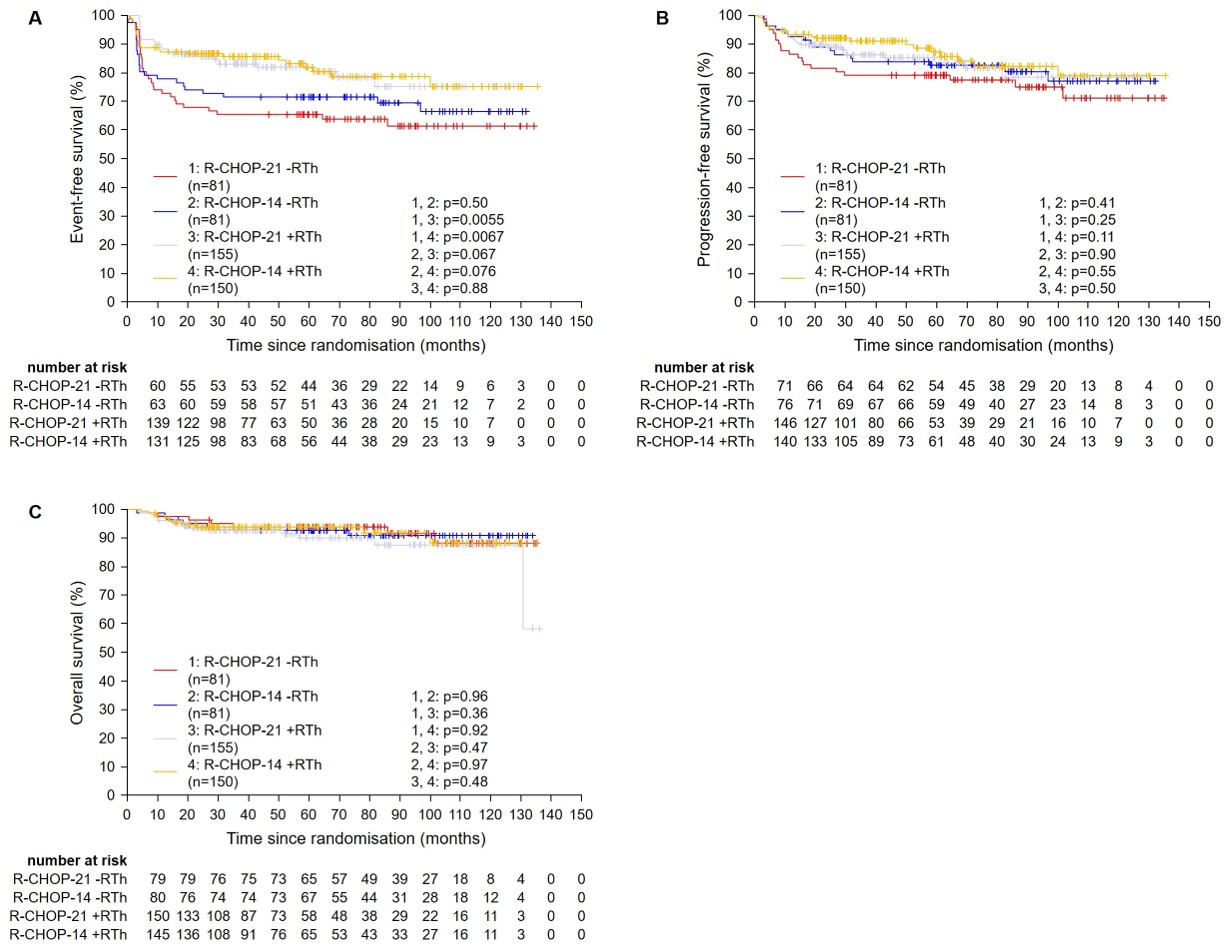
**Supplementary Figure S2. EFS, PFS, and OS according to therapy arms**. Graphs show EFS (A), PFS (B) and OS (C) in patients qualifying for radiotherapy according to therapy arms.


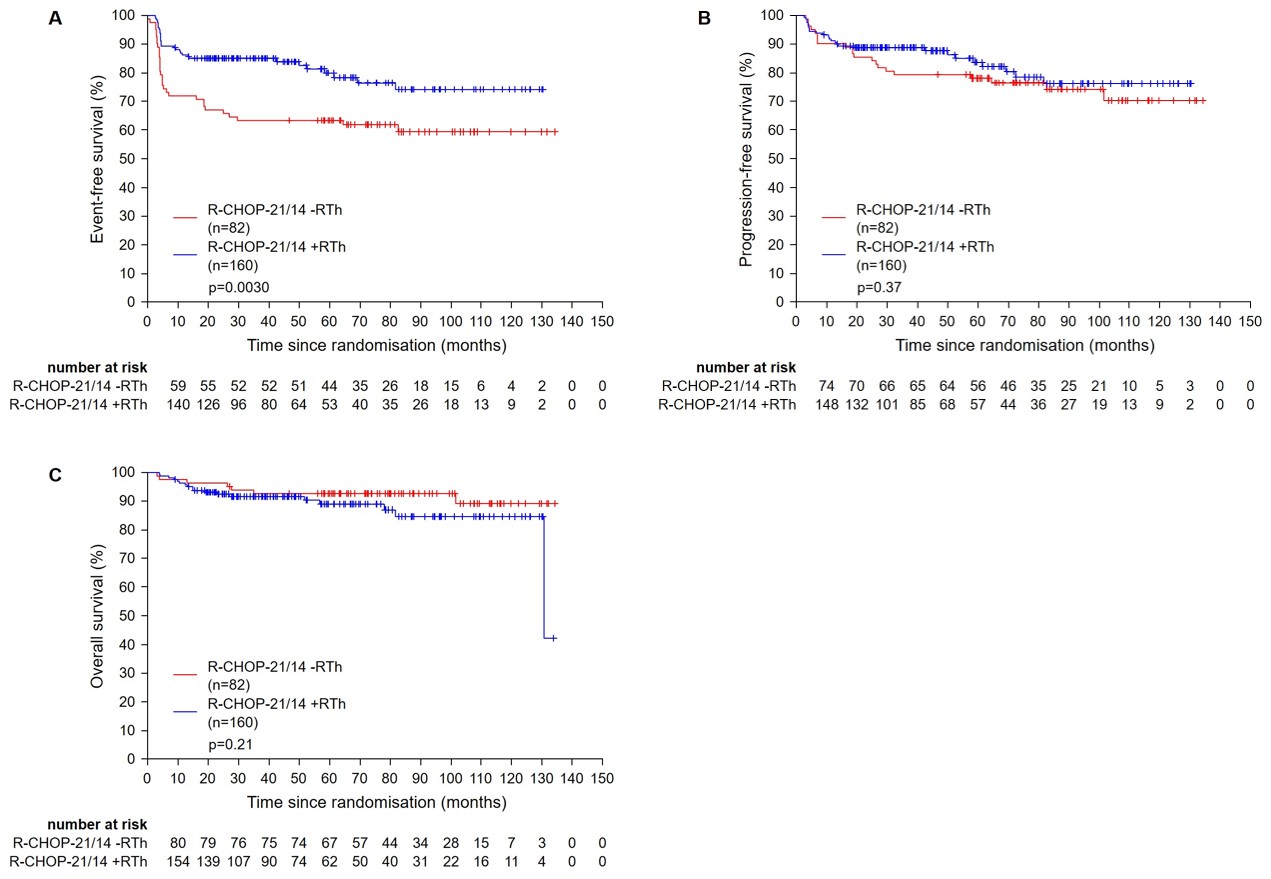


**Supplementary Figure S3. EFS, PFS, and OS in patients qualifying for radiotherapy with extralymphatic involvement.**

Graphs show EFS (A), PFS (B), and OS (C) according to radiotherapy - or observation-arm.


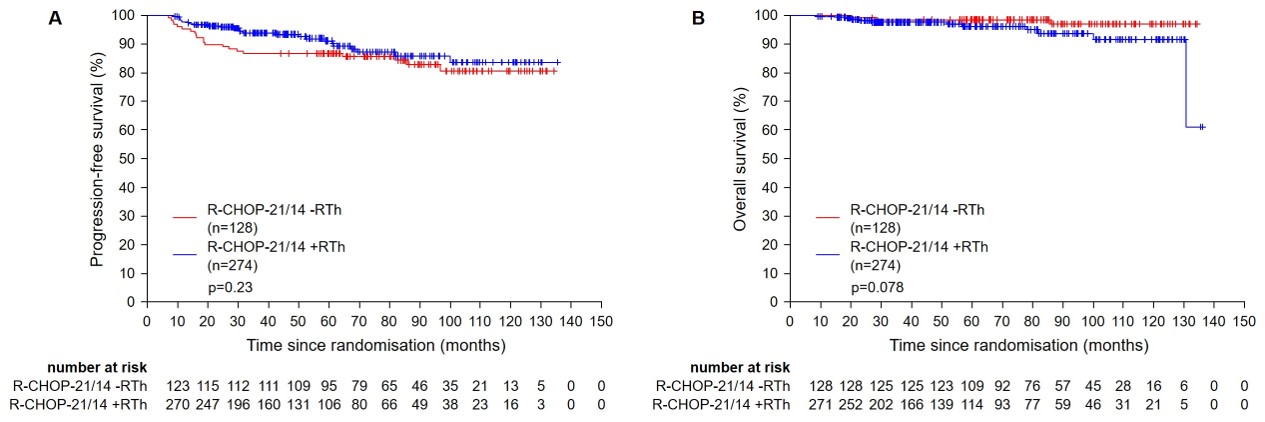


**Supplementary Figure S4. PFS and OS for patients qualifying for radiotherapy, who achieved a CR/CRu after R-CHOP chemotherapy.** Graphs show PFS (A) and OS (B).


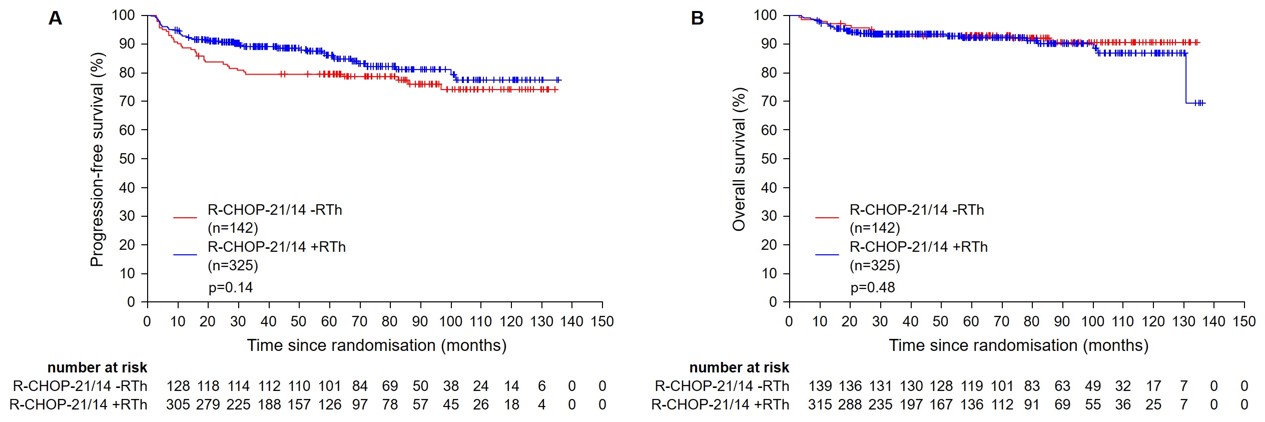


**Supplementary Figure S5. PFS and OS for patients qualifying for radiotherapy as treated analysis.** Graphs show PFS (A) and OS (B).


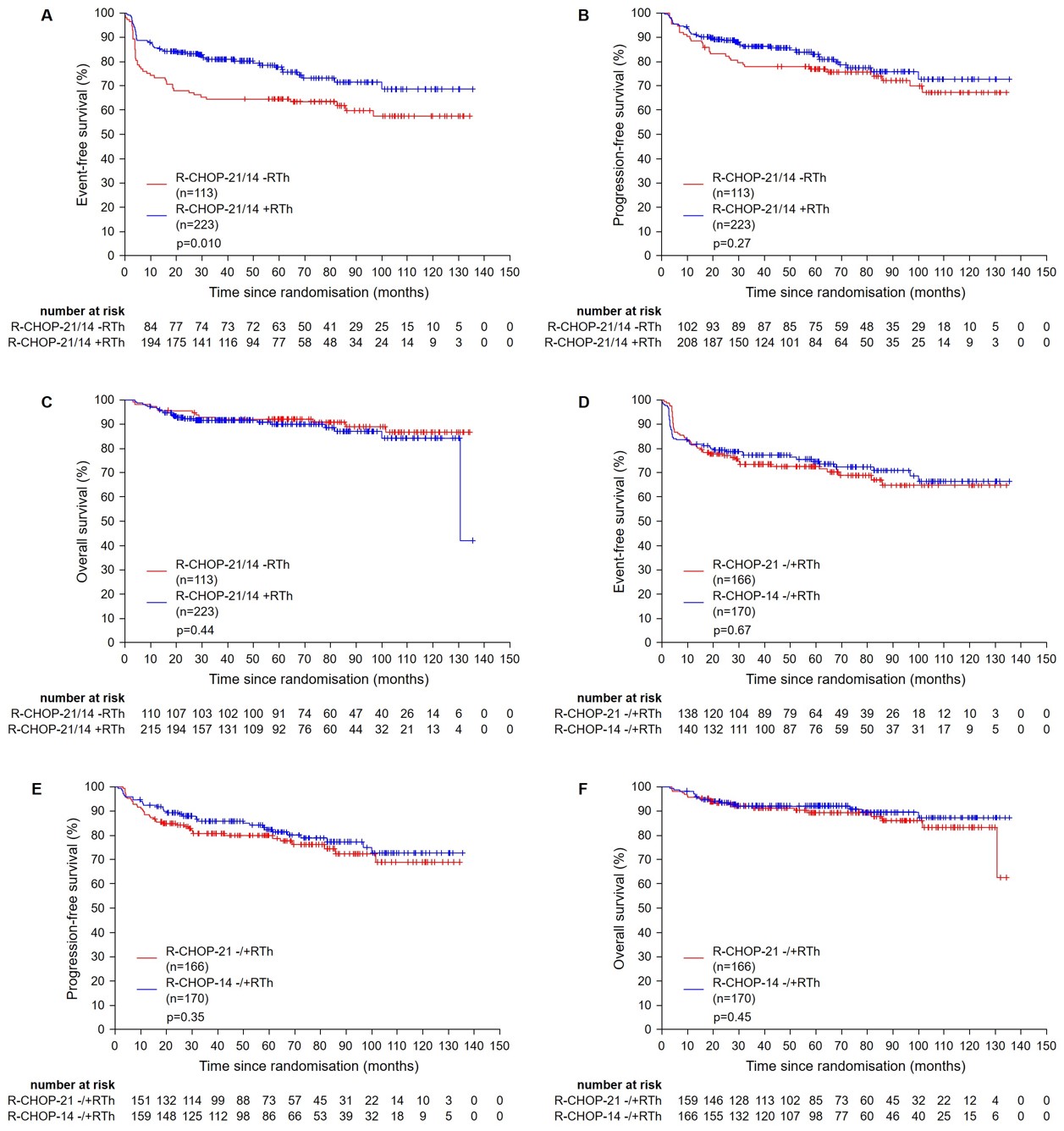


**Supplementary Figure S6. EFS, PFS, and OS according to radiotherapy- or observation-arm and R-CHOP-14 or R-CHOP21 arm** **for non- primary mediastinal B-cell lymphoma patients qualifying for radiotherapy.** Graphs show EFS (A), PFS (B), and OS (C) according to radiotherapy- or observation-arm and EFS (D), PFS €, and OS (F) according to R-CHOP-14 or R-CHOP21 arm.


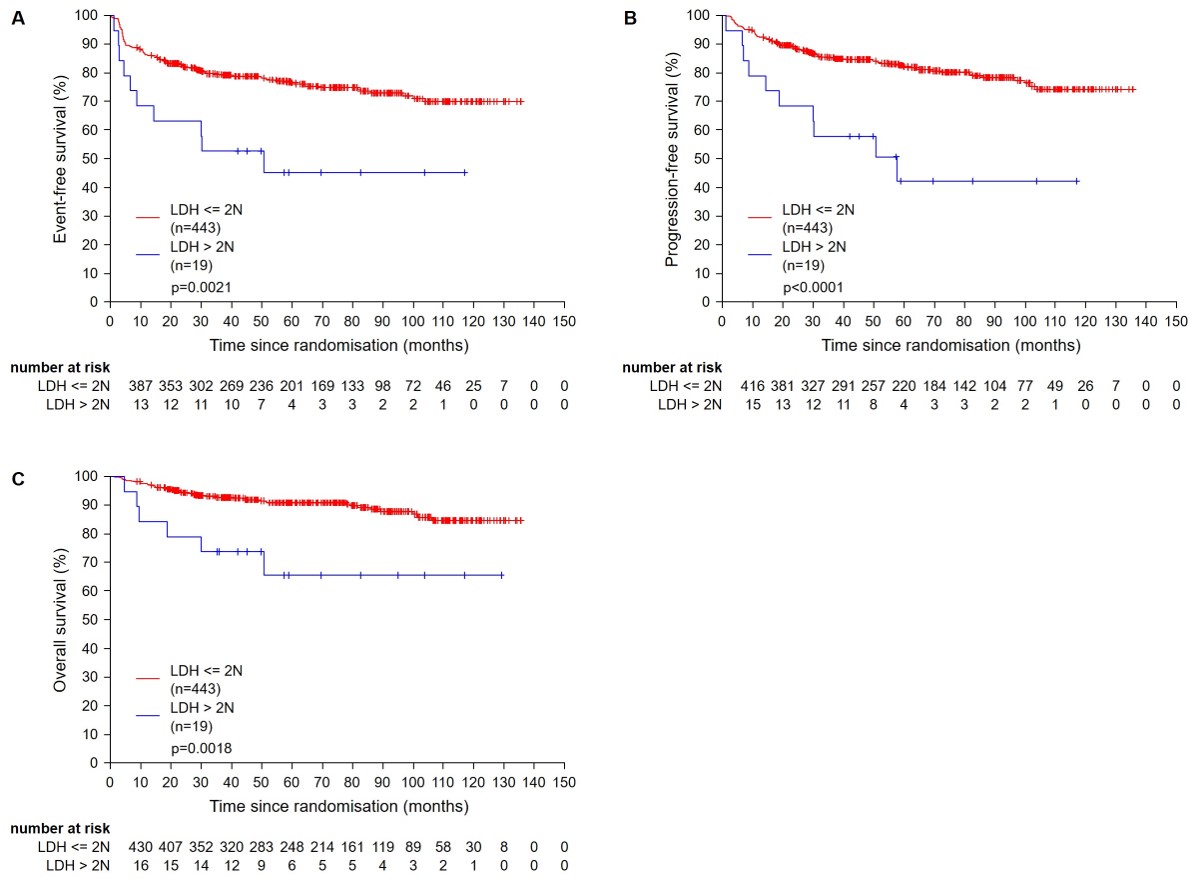


**Supplementary Figure S7: EFS, PFS, and OS for non- primary mediastinal B-cell lymphoma and aaIPI 1 patients according to LDH.** Graphs show EFS (A), PFS (B), and OS (C) according to LDH > twice the upper limit of normal [LDH <= twice the upper limit of normal compared to LDH > twice the upper limit of normal］.

**
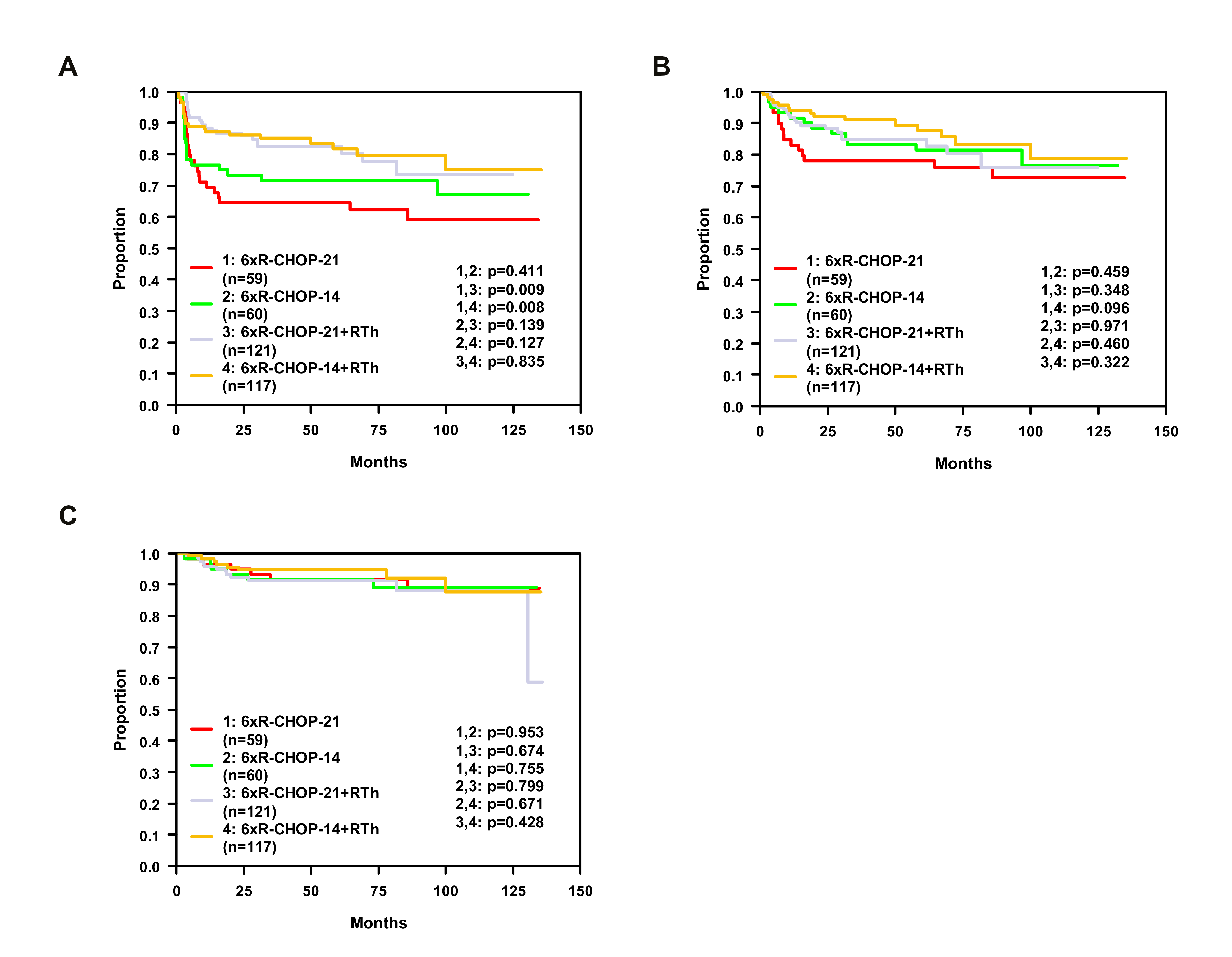
**

**Supplementary Figure S8: Event- free, progression-free, and overall survival for patients with bulky disease according to treatment arm.** Graphs show EFS (A), PFS (B), and OS (C) in patients qualifying for radiotherapy according to therapy arms.
